# Overlooked considerations in prescribing green and blue infrastructure solutions for urban environments

**DOI:** 10.1016/j.xinn.2025.101184

**Published:** 2025-11-19

**Authors:** Prashant Kumar, Karina Corada Perez, Akash Biswal, Hao Sun, Anubhav Kumar Dwivedi, Sarkawt Hama, Soheila Khalili, Ajit Ahlawat, Maria de Fatima Andrade, Ronaldo Adriano Alves, Emannuelly A. Amaral dos Santos, Maria Athanassiadou, Camilo Bastos Ribeiro, Prabin Bhusal, Miguel Luiz Bucalem, Bonnie G. Buchanan, Leticia Figueiredo Candido, Shi-Jie Cao, Amarilis Lucia Casteli Figueiredo Gallardo, Ruidong Chang, Amanda K. Chaves Ribeiro, Brian Considine, Regina Maura de Miranda, Letícia Aparecida de Paiva, Priyanka de Souza, Marco A. Franco, Edmilson D. Freitas, H. Christopher Frey, Marco F. Funari, Bruno Furieri, John Gallagher, Leandro Luiz Giatti, Marcos Jeronimo Goroski Rambalducci, Christos H. Halios, Felicity Harris, Leonardo Hoinaski, Colin Horton, Yuhan Huang, Laurence Jones, Robyn Jones, John Kandulu, Madhusudan Katti, Giuliano Maselli Locosselli, Augusto Akio Lucchezi Miyahara, Jorge Alberto Martins, Leila Droprinchinski Martins, Mauricio Cruz Mantoani, Roberta Consentino Kronka Mülfarth, Yasmin Kaore Lago Kitagawa, Willian Lemker Andreão, Jackson Lemons, Giulia Mariano Machado, Shelagh K. Malham, Meredith P. Martin, Maria Clara V.M. Starling, Aonghus McNabola, Otavio Medeiros Sobrinho, Eugene Mohareb, Erick G. Sperandio Nascimento, Thiago Nogueira, Gwilym Owen, Rajan Parajuli, Hari Prasad Pandey, Rizzieri Pedruzzi, Pedro José Pérez Martínez, Janaina Antonino Pinto, Jorge Armando Piscoya Santibañez, Shila Pokhrel, Paula Lelis Rabelo Albala, Neyval C. Reis, Anderson P. Rudke, Devendra Saroj, Yiming Sui, Veronica Soebarto, Yonatal Tefera, Taciana Toledo de Almeida Albuquerque, Bruna Lima Veras Maia, Fang Wang, Jannis Wenk, Robson Will, Carmel Williams, Hannah Sloan Wood, Qingyun Wu, Chang Xi, Russell Yates, Runming Yao

**Affiliations:** 1 Global Centre for Clean Air Research (GCARE), School of Engineering, Civil and Environmental Engineering, Faculty of Engineering and Physical Sciences, University of Surrey, Guildford GU2 7XH, UK; 2 Institute for Sustainability, University of Surrey, Guildford GU2 7XH, UK; 3 Sustainability Research Institute, University of East London, London E16 2RD, UK; 4 Department of Geoscience and Remote Sensing, Delft University of Technology (TU Delft), 2628 CN Delft, the Netherlands; 5 Leibniz Institute for Tropospheric Research, e.V. (TROPOS), 04318 Leipzig, Germany; 6 Institute of Astronomy, Geophysics and Atmospheric Sciences, Department of Atmospheric Sciences, University of São Paulo, São Paulo 05508-090, Brazil; 7 Department of Geography, State University of Londrina, Londrina 86057-970, Brazil; 8 Department of Sanitary and Environmental Engineering, School of Engineering, Federal University of Minas Gerais, Belo Horizonte 31270-901, Brazil; 9 Urban Climate Applications, Met Office, Exeter EX1 3PB, UK; 10 Department of Sanitary and Environmental Engineering, Federal University of Santa Catarina, Florianópolis 88040-070, Brazil; 11 Department of Forestry and Environmental Resources, North Carolina State University, Raleigh, NC 27695, USA; 12 Institute of Forestry, Tribhuvan University, Pokhara 33700, Nepal; 13 Polytechnic School, University of São Paulo, São Paulo 05508-010, Brazil; 14 Sustainable and Explainable Fintech (SAEF) Center, University of Surrey, Guildford GU2 7XH, UK; 15 Institute of Environmental Research from the State of São Paulo, São Paulo 01061-970, Brazil; 16 School of Architecture, Southeast University, 2 Sipailou, Nanjing 210096, China; 17 Smart and Sustainable Cities Program at the University Nove de Julho, São Paulo 01525-000, Brazil; 18 School of Architecture and Civil Engineering, The University of Adelaide, Adelaide, SA 5005, Australia; 19 Department of Civil, Structural & Environmental Engineering, Trinity College Dublin, The University of Dublin, Dublin D02 PN40, Ireland; 20 School of Arts, Sciences and Humanities, University of São Paulo, São Paulo 03828-000, Brazil; 21 Faculty of Engineering, Architecture and Urbanism, University of Campinas, Campinas 13083-970, Brazil; 22 Department of Urban and Regional Planning, University of Colorado Denver, Denver, CO 80202, USA; 23 CU Population Center, University of Colorado Boulder, Boulder, CO 80302, USA; 24 Department of Civil, Construction, and Environmental Engineering, North Carolina State University, Raleigh, NC 27606, USA; 25 Department of Environmental Engineering, Federal University of Espírito Santo, Vitória, ES 29075-910, Brazil; 26 Department of Environmental Health, School of Public Health, University of São Paulo, São Paulo 01246904, Brazil; 27 Federal University of Technology, Parana 80230-910, Brazil; 28 School of Built Environment, University of Reading, Reading RG6 6DF, UK; 29 Portsmouth City Council, Portsmouth PO1 2AL, UK; 30 Rugby Borough Council, Rugby CV21 2RR, UK; 31 Centre for Green Technology, School of Civil and Environmental Engineering, University of Technology Sydney, Sydney, NSW 2007, Australia; 32 UK Centre for Ecology & Hydrology, Deiniol Road, Bangor LL57 2UW, UK; 33 Liverpool Hope University, Department of Geography and Environmental Science, Liverpool L16 9JD, UK; 34 School of Psychology and Sport Science, Bangor University, Bangor LL57 2DG, UK; 35 College of Business, Government and Law, Flinders University, Bedford Park, SA 5042, Australia; 36 Center for Nuclear Energy in Agriculture, University of São Paulo, São Paulo 13416-000, Brazil; 37 Faculty of Architecture, Urbanism and Design, University of São Paulo, São Paulo 05508-080, Brazil; 38 Foundation for Scientific and Cultural Development, Federal University of Lavras, Lavras 37203-202, Brazil; 39 ArcelorMittal, Global Research and Development, Espirito Santo 29161-376, Brazil; 40 School of Ocean Sciences, Bangor University, Menai Bridge, Anglesey LL59 5AU, UK; 41 School of Engineering, RMIT University, Melbourne, VIC 3000, Australia; 42 Surrey Institute for People-Centred Artificial Intelligence, Faculty of Engineering and Physical Sciences, University of Surrey, Guildford GU2 7XH, UK; 43 Stricto Sensu Department, SENAI CIMATEC University, Salvador 41650-010, Brazil; 44 Resilience Unit, Cardiff Council, Cardiff CF10 4UW, UK; 45 University of Southern Queensland, Toowoomba, QLD 4350, Australia; 46 Ministry of Forests and Environment, Government of Nepal, Kathmandu 44600, Nepal; 47 Department of Sanitary and Environmental Engineering, Rio de Janeiro State University, Rio de Janeiro 20550-013, Brazil; 48 Department of Infrastructure and Environment, Faculty of Civil Engineering, Architecture and Urbanism, State University of Campinas, São Paulo 13083-889, Brazil; 49 School of Public Health, The University of Adelaide, Adelaide, SA 5005, Australia; 50 State Key Laboratory of Soil and Sustainable Agriculture, Institute of Soil Science, Chinese Academy of Sciences, Nanjing 210008, China; 51 University of Chinese Academy of Sciences, Beijing 100049, China; 52 Joint International Research Laboratory of Green Buildings and Built Environments (Ministry of Education), Chongqing University, Chongqing 400045, China; 53 Federal Institute of Hydrology (BfG), Department G - Qualitative Hydrology, Am Mainzer Tor 1, 56068 Koblenz, Germany; 54 Department of Chemical Engineering, University of Bath, Bath BA2 7AY, UK; 55 Independent Consultant, 2200 Copenhagen, Denmark; 56 Surrey County Council, Woodhatch, Reigate RH2 8EF, UK; 57 National Centre for International Research of Low-carbon and Green Buildings (Ministry of Science and Technology), Chongqing University, Chongqing 400045, China; 58 Faculty of Architecture and Urbanism, University of Brasília, Brasília 70904-900, Brazil; 59 Joint FAO/IAEA Centre of Nuclear Techniques in Food and Agriculture, International Atomic Energy Agency, Vienna 1400, Austria

**Keywords:** climate adaptation, urban resilience, nature-based solutions, multidimensional challenges, United Nations Sustainable Development Goals, GBI constraints

## Abstract

Green and blue infrastructure (GBI) is emerging as a key strategy for climate adaptation and urban resilience, yet its implementation often faces critical contextual barriers. This review initially screened over 29,000 publications, ultimately synthesizing more than 500 relevant studies supplemented by diverse expert input. The result is a novel integrative framework that connects previously siloed knowledge and consolidates 21 underexplored barriers across four key domains of GBI implementation: environmental, social, economic, and governance/policy. Environmental barriers include conflicts between GBI and renewable energy goals, specifically photovoltaics, unintended consequences of GBI (such as allergenic pollen production), urban ventilation disruption, and vulnerability of plant species to multiple urban stressors. Effective responses include thoughtful allocation and integration of photovoltaics and GBI, developing context-specific frameworks combining ecological knowledge with technological innovation, fostering cross-disciplinary collaboration across technical and social domains, science-based species selection and implementing multi-scalar strategies that enhance ecological connectivity. Social barriers encompass environmental injustice, cultural disconnection, limited public adoption, safety concerns, and esthetic preferences favoring manicured over ecologically functional landscapes. These challenges highlight the need for participatory design, culturally responsive planning, and inclusive resource allocation to strengthen community engagement and long-term stewardship. Economic barriers stem from biodiversity undervaluation, inadequate asset recognition in accounting frameworks, incomplete cost-benefit analyses, and limited private investment. Innovative financing tools such as green bonds and debt-for-nature swaps offer promising mechanisms for resilient financing, while standardized natural capital accounting frameworks can better capture GBI’s multifunctional value. Governance barriers include land scarcity, urban design limitations, policy fragmentation, and disconnects with other urban agendas such as walkability. Overcoming these requires institutional realignment, cross-sectoral collaboration, and integrated spatial planning. The review unifies these findings into 12 actionable recommendations to support holistic decision-making, emphasizing that effective GBI implementation demands context-specific strategies combining innovation, inclusive governance, and long-term stewardship to mainstream GBI in sustainable urban development.

## Public summary

• 21 unseen barriers to green/blue infrastructure (GBI) are identified, with key solutions to fix these issues.
• GBI environmental barriers include green energy clashes, unwanted side effects, and urban plant stress.
• GBI social barriers include cultural/equity issues, safety concerns, design tastes, and need community input.
• GBI financial barriers include undervaluation, investment gaps, and limited funding/benefit recognition.
• GBI governance barriers include land limits, traditional designs, policy gaps, and competing urban priorities.

## Graphical abstract

**Figure:**
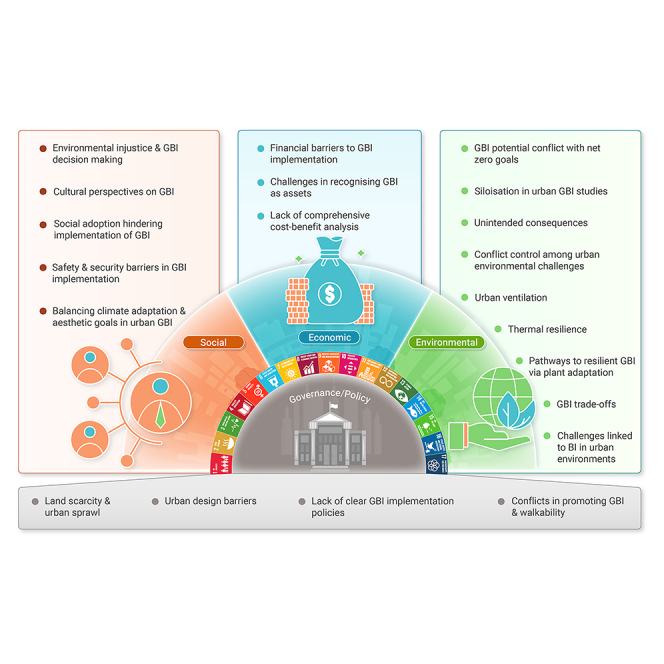

## Introduction

Cities now house ∼55% of the global population, projected to reach 68% by 2050 (Figure S1).^1^ Due to high population and building density, cities face serious environmental issues namely air pollution, heat island effects, floods,^2^ and droughts,^3^ all of which contribute to poor outcomes for human health and biodiversity. These challenges are often addressed separately in a reactive manner rather than holistically. To build resilient cities, implementing green and blue infrastructure (GBI), also called “nature-based solutions (NbSs)” is crucial.^4^^,^^5^ In this review, we adopt GBI as an umbrella term encompassing green infrastructure (GI) (e.g., parks, street trees, and gardens) and blue infrastructure (BI) (e.g., rivers, ponds, lakes, and natural wetlands), as well as hybrid infrastructure such as green walls, living green roofs, urban agriculture, rain garden, bioswales, permeable paving, and constructed wetlands, which integrate natural elements into or around gray infrastructure.^5^^,^^6^

GBI is increasingly recognized as a sustainable strategy to enrich urban resilience, reduce climate risks, and promote ecological sustainability while delivering diverse environmental, health, and economic benefits.^7^^,^^8^ For instance, in Barcelona, adhering to international exposure recommendations could prevent almost 2,000 premature deaths annually, primarily through increased physical activity and reduced levels of air pollution, traffic congestion, noise, and urban heat, along with improved access to green spaces.^9^ Similarly, exposure to greenspace could prevent up to 10% of childhood overweight and obesity cases.^10^ School greenery has been linked to a 20% reduction in mental health issues^11^ and a 6% improvement in children’s memory compared with those with less green surroundings.^12^ GBI can increase local property values by 5%–20% depending on proximity and quality of green space.^13^ Additionally, strategic urban planning incorporating GBI provides multiple benefits including reduced urban heat, lower cooling energy demand, decreased healthcare costs, and improved stormwater management.^14^^,^^15^

Globally, over 130 countries have embedded urban greening initiatives into their national Sustainable Development Goals (SDGs) commitments.^8^ These nature-based approaches complement technology-based solutions.^16^ In this context, the International Union for Conservation of Nature (IUCN) Global Standard for NbSs connects ecosystem-based approaches to urban greening, supporting SDG implementation.^17^ For example, Natural England’s Green Infrastructure Framework in the UK sets a 40% green cover for urban residential areas by 2035.^18^

Beyond ecological resilience, GBI fosters cultural vibrancy and environmental education. International frameworks, such as the United Nations (UN) SDGs and UNESCO’s initiatives highlight its role in cultural well-being and place-based learning.^19^ Urban GBI delivers diverse cultural ecosystem services (CESs) by fostering a sense of place, safeguarding heritage values, and enabling experiential learning in everyday landscapes.^20^ National and local initiatives, such as green school initiatives and culturally adaptive landscape planning, further reinforce how GBI enriches community identity^21^ and supports environmental education.^22^ However, inequities in access, limited community engagement and adoption, and culturally disconnected design practices reveal that GBI often struggles to resonate with diverse urban social realities. Embedding GBI in inclusive, locally grounded processes is therefore essential, as urban greening is gradually shifting from a purely ecological intervention toward a more socially embedded infrastructure for cultural resilience, social equity, and collective learning.^23^

As climate challenges intensify, GBI has become central to risk mitigation strategies and urban resilience frameworks. The UN SDGs call for increased investment in NbSs, with GBI supporting multiple goals: health and environment (SDG3: Good health and well-being; SDG15: Life on land), urban development (SDG11: Sustainable cities and communities), resource management (SDG2: Zero hunger; SDG6: Clean water and sanitation; SDG12: Responsible consumption and production), energy systems (SDG7: Affordable and clean energy), resilient infrastructure (SDG9: Industry, innovation and infrastructure), and climate response (SDG13: Climate action; SDG14: Life below water). This integrated approach demonstrates GBI’s synergistic advancement of sustainable development priorities (see Figure S2). Responding to these imperatives, the IUCN developed the Global Standard for NbSs to provide a framework for integrating ecological, social, and economic goals.^17^ In Europe, the EU Green Infrastructure Strategy^24^ and the EU Biodiversity Strategy for 2030^25^ advocate for coordinated, cross-sectoral greening policies to mitigate air quality, urban heat, flood risks, and biodiversity loss. The latter requires all European cities with populations over 20,000 to develop Urban Greening Plans by 2030. These strategies reflect a widespread policy shift toward evidence-based approaches and multifunctional GBI design. Nevertheless, existing planning practices and academic approaches remain fragmented, with limited integration of climate risk, equity, and co-benefits in GI decision-making. Robust, spatially explicit methods are urgently needed to optimize GBI placement for maximizing environmental and social outcomes^26^ as well as to address such conflicts.

A wide range of recent literature has synthesized GBI and NbSs from diverse disciplinary perspectives (Table 1). Table 1 summarizes reviews (2017–2025) highlighting their role in addressing interconnected urban challenges. These studies have predominantly focused on GBI’s capacity to mitigate environmental risks in urban areas, such as urban heat island (UHI), air pollution, flooding, and climate-induced stress.^4^^,^^5^^,^^6^^,^^62^ Some reviews explored optimal GBI placement strategies,^33^^,^^36^ environmental justice (EJ) implications,^34^ and planning instruments for public green space provision.^35^ A significant share of these studies focuses on GBI’s environmental and climate-regulating functions, including stormwater management^47^ and its contribution to hazard reduction and ecosystem service (ES) delivery.^41^^,^^52^ Few have emphasized the contribution of GI on human health,^44^ reducing thermal discomfort,^42^ and improving mental resilience,^43^ and reducing vulnerability to heat.^45^ Others assessed the ES delivered by specific GBIs, such as green roofs, street trees, or urban agriculture, and identified regional disparities in implementation, particularly the underrepresentation of developing countries in both practice and research.^39^^,^^40^ Recent reviews also advanced methodological frameworks to assess GBI effectiveness in reducing hydro-meteorological hazards,^63^^,^^64^ proposed standardized evaluation tools,^65^^,^^66^ and revealed gaps in monitoring, stakeholder engagement, and integration with gray infrastructure.^35^^,^^37^

**Table 1:** Summary of key relevant review papers from the past decade on GBI research

Author (year)	Key focus area of review	What was covered
Wang et al.^27^	understanding ecosystem services provided by urban GBI, using a bibliometric analysis to map research trends and knowledge clusters	identified major themes such as air quality improvement, biodiversity support, urban cooling, water management, and soil functions
Zarei and Shahab^28^	identifying the success factors and implementation challenges of NbSs in GI, and categorized barriers across institutional, social, economic, and technical dimensions	classified 21 underexplored barriers (e.g., governance gaps, cultural resistance, undervaluation of biodiversity, and financing limitations) from over 500 studies
Chau et al.^29^	understanding the barriers and challenges to implementing GI, with particular insights from Melbourne’s urban policy and planning context	recognized obstacles such as fragmented governance, insufficient funding, lack of technical expertise, and limited political prioritization
Seidu et al.^30^	understanding the integration of green and gray infrastructure systems in dense urban regions	presented institutional, technical, financial, and governance challenges, such as a lack of professional capacity, and outlined effective guidelines, including adopting hybrid design approaches and leveraging digital tools
Kim and Kim^31^	understanding the evolution of research on GI for urban flooding	highlighted technical hydrological performance studies toward socio-ecological frameworks, hybrid blue-green-gray systems, and multidisciplinary approaches to GI for flood resilience
Tao et al.^32^	integration of computational fluid dynamics and machine learning for urban GI	examined role of integrated computational fluid dynamics-machine learning approaches for urban GI design, specifically heat mitigation and air quality improvement
Dobrinić et al.^33^	use of computational learning for GI mapping optimization	reviewed various techniques of deep learning for GI mapping used in sustainable urban development
Kumar et al.^8^	barriers, significances, successful case studies, and greening initiatives opportunities in urban settings	emphasized the need for a holistic, inclusive, and cross-sectoral collaboration combined with a forward-looking approach to urban greening to build cities that are more resilient, sustainable, and equitable
Li et al.^34^	environmental justice in NbS implementation	identified key challenges and offered recommendations for NbS use in managing UHI, flooding, wildfire, COVID-19, and air pollution
Muñoz and Duarte^35^	urban planning tools to expand GI in public spaces	analyzed 126 global strategies leveraging GI to address extreme climate change events
Sobhaninia et al.^36^	optimal location of GI to mitigate UHI and manage stormwater	assessed 8 GI types, integrating environmental, social, and economic factors to support informed placement decisions
Khalili et al.^37^	methods for evaluating urban GI benefits	reviewed monitoring, remorse sensing, and modeling approaches assessing GI’s impact on heat regulation, human thermal experience, and air pollution; identified strengths, limitations, and key parameters of each method
Kumar et al.^5^	overheating in urban areas and role of GBGI	examined 51 types of GBGI to understand their effectiveness in reducing urban heating
Kumar et al.^4^	air pollution mitigation and GBGI	assessed the air quality benefits of 51 GBGI types across urban environments
Perera et al.^38^	GBI policy framework in 12 global cities	highlighted the emphasis on vegetation cover in policies and the need for GBI policies alignment at state and local government levels
Przestrzelska et al.^39^	GBI in rainwater management	revealed GBI’s limited applicability in diverse climates and research bias toward high-GDP countries
Tate et al.^40^	economic evaluation of GBI interventions	revealed a lack of stakeholders’ involvement and underrepresentation of studies from low-income and emerging economies
Debele et al.^41^	global role of NbSs in mitigating natural hazards	consolidated and analyzed NbS case studies worldwide; showing effectiveness in reducing natural hazard and climate changes
de Quadros and Mizgier^42^	GI strategies for pedestrian thermal comfort	identified street trees, green walls, and green spaces as effective cooling tools; green roofs had minimal pedestrian-level impact
Li and Lange^43^	GBI and stress resilience	explored links between urban landscapes with green cover (gardens, parks, wetlands, corridors, rivers, canals) and stress responses
Potter et al.^44^	health benefits of GBGI exposure	confirmed positive health outcomes from GBGI, although mechanisms remain insufficiently understood
Adnan et al.^45^	heat vulnerability and mitigation in Australia	highlighted the usefulness of GI and water-conscious urban planning in reducing heat-related risks
Evans et al.^46^	ES from urban agriculture and GI	demonstrated that community gardens, green spaces, parks, and allotments provide a wide array (16+) of ES
Jones et al.^6^	ES, trade-offs, and synergies among urban GI	provided a new typology of GI, and reviewed the literature to create a matrix of GI × ES delivery to inform planning and illustrate synergies and trade-offs for environmental and social outcomes
Almaaitah et al.^47^	GBI’s dual role in UHI mitigation and stormwater management	found strong evidence for stormwater benefits, with fewer studies focused on UHI mitigation effectiveness
Choi et al.^48^	co-benefits and trade-off for different GI types	found GI strategies primarily focused on climate adaptation, with limited attention to socio-cultural benefits
Kumar et al.^49^	monitoring methods for NbS performance against natural hazards	analyzed NbS monitoring methods and instruments to assess their effectiveness and challenges in addressing droughts, heatwaves, floods, landslides, storm surges, and coastal land loss
Kumar et al.^50^	modeling approaches for NbS efficiency	assessed hydrological and hazard modeling methods for evaluating NbSs, outlining benefits and data limitation. Highlighted the necessity to develop multi-scale process-based models to better assessment NbS benefits
Toxopeus and Polzin^51^	financing challenges and solutions for NbS (parks, trees, allotment gardens, and GBI)	identified key funding barriers and proposed strategies to improve benefit valuation and public-private investment balance
Veerkamp et al.^52^	GBI and ES delivery (local temperature regulation, stormwater management, waste processing, air pollution control, pollination services, and recreational and esthetic benefits)	emphasized gaps in ES and GBI coverage; most studies focused on temperature regulation and esthetics, often in parks or unspecified green spaces
Kumar et al.^53^	operationalizing NbS for hazard mitigation	analyzed the European policy frameworks applicable to hydro-meteorological hazards for NbS in policy and proposed NbS planning with focus on co-benefits and co-designed
Shah et al.^54^	frameworks and indicators for hydro-meteorological risk in NbS	suggested a framework for assessing vulnerability and risk within the scope of NbSs. Critiqued existing hazard and risk assessment indicators, calling for more inclusive NbS relevant metrics
Ying et al.^55^	strategic GI implementation	described GI as a multidisciplinary utility for delivering environmental and socioeconomic benefits simultaneously, with Europe and US leading in GI research
Debele et al.^56^	revised NbS concepts and classification of hydro-meteorological hazards	examined the impacts of hydro-meteorological risks (HMHs) in Europe and explores how NbSs can strengthen resilience, reduce adverse effects of HMHs, and support environmental sustainability
Ruan et al.^57^	understanding the positive and negative impacts of GI on the food-water-energy nexus	developed a framework to characterize the role of GI in sustaining food-water-energy nexus
Meng et al.^58^	quantification of the food-water-energy nexus in urban GBI	highlighted that most GBGI studies examine isolated benefits or life cycle impacts, while neglecting transboundary effects
Bellezoni et al.^59^	understanding how urban GBI influences the food, water, and energy nexus	established need for policies and research to shift from isolated to integrated approaches to fully connect GBI for sustainable urban futures
Venkataramanan et al.^60^	health and well-being outcomes of GI for water management	found limited evidence on human health impact; emphasized the need for community support and maintenance of GI
O’Brien et al.^61^	cultural ES of urban GI	classified well-being outcomes form 7 GI types into capability, experiences, and identities

Institutional constraints, competing uses of limited land, and fragmented policies can limit the implementation of GBI at scale. Gaps remain in research on ES,^61^ inclusive governance,^34^ and the socio-spatial distribution of benefits, particularly in developing countries.^40^ Methodological reviews further identify the need for improved modeling, monitoring, and evaluation tools.^37^^,^^50^ GBI implementation remains constrained by fragmented approaches and narrow technical focus, with deeper integration of cultural, social, and policy dimensions still lacking.^67^ Overlooked issues include competing land use, inadequate or absent financing mechanisms, fragmented NbS policies, and unintended environmental consequences such as pollen, biogenic volatile organic compounds (bVOCs), and trade-offs with other urban agendas, such as net zero energy goals or car-centric urban design. Moreover, extreme climatic events, such as heatwaves, intense rainfall, prolonged droughts, and severe storms, may themselves constitute substantial barriers to its implementation and sustained maintenance.^68^

This review shifts focus from GBI benefits to underexplored barriers impeding its urban implementation. The comprehensive cross-disciplinary synthesis bridges fragmented knowledge by integrating perspectives from urban planning, ecology, climate science, economics, and social equity research. It creates a cohesive framework that analyses critical interconnections with energy efficiency, walkability, climate resilience, EJ, and competing land uses, concluding with actionable recommendations for more resilient and socially embedded GBI strategies. While some barriers (e.g., bVOCs, EJ, governance fragmentation) are well documented in the literature, they are typically examined in isolation and remain weakly embedded in planning standards, valuation methods, and governance processes. The term “overlooked” reflects this gap between existing knowledge and systematic application, positioning this review as a crucial integrative contribution that connects disparate research domains with practical implementation.

The overarching goal of this integrative review is to synthesize critical dimensions of GBI implementation that remain underexplored in the current literature. Specifically, the review develops a unified framework examining: (1) environmental barriers, including technical and system integration and strategic alignment challenges (GBI net zero conflicts, research siloization), environmental and ecological performance limitations (unintended consequences such as bVOC emissions), and climate management complexities (ventilation impacts, thermal resilience, plant adaptation, GBI trade-off, and BI); (2) social barriers, encompassing environmental injustice, cultural disconnection, adoption challenges, safety concerns, and esthetic controversy; (3) economic barriers, involving financial undervaluation of biodiversity, asset recognition issues in accounting frameworks, cost-benefit analysis (CBA) limitations, and investment barriers; and (4) governance/policy barriers, comprising land and space constraints, urban design barriers, policy fragmentation, integration challenges with other urban systems, and regulatory gaps. By bridging these previously siloed knowledge domains, this review provides key conclusions and actionable recommendations to support more holistic and effective decision-making in GBI implementation.

## Scope, methods, and outline

The scope of this review is confined to underexplored barriers that hinder the implementation of GBI in urban areas. Monitoring and modeling, health impact assessment, and multi-benefit analyses methodologies of GBI interventions lie outside this review’s scope. For comprehensive coverage of these aspects, readers are directed to the key resources summarized in Table 1.

**Figure 1:**
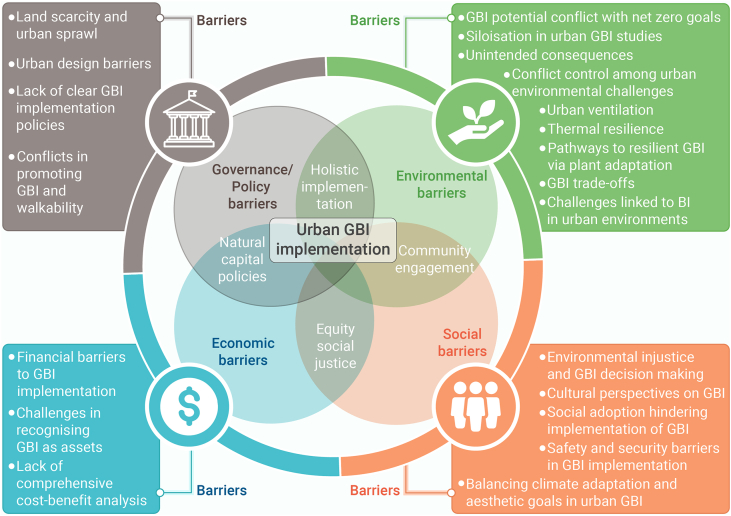
The topic areas covered in this review demonstrate a sustainability framework encompassing environmental, social, economic, and governance barriers hindering GBI implementation

Barriers were identified and collated through a series of co-design workshops, involving numerous international experts in the field,^69^ with a wider writing team involved in reviewing each topic, and informed by prior research.^70^ The co-design process for barrier identification involved a multi-stage approach: (1) initial identification through a large interdisciplinary workshop, (2) independent refinement by a smaller multidisciplinary expert group workshop, and (3) finalization via full-author iteration to ensure a balanced, non-redundant list of overlooked barriers. Further details are provided in section S1. As illustrated in Figure 1, barriers were organized using a sustainability framework,^71^ across four domains: environmental, social, economic, and the cross-cutting theme of governance and policy barriers that impede GBI implementation.

Literature search in Web of Science and Scopus databases was conducted, using structured search terms covering the challenge, and relevant to each topic section. The search term used included “Challenge AND Implementation AND (‘Green infrastructur’ OR ‘Blue infrastructure’) AND Urban.” In addition, combinations of terms such as “Barrier OR Constraint AND (Green infrastructure OR Blue infrastructure) AND Implementation AND Urban and Obstacle AND Adoption AND (‘Nature-based solutions’ OR ‘Ecosystem services’) AND City” were also tested to ensure thematic breadth. Separate searches were conducted for each barrier domain (environmental, social, economic, governance/policy), with domain-specific keywords (e.g., “air quality,” “heat stress,” “public acceptance,” “financing,” “policy integration”) combined with the core GBI terms, yielding ∼29,000 results (Figures S2 and S3). Figure S2 illustrates the distribution of GBI publications across different SDGs, aligned SDG11 and SDG13 showing the highest share of publications. Figure S3 shows the year-wise publication trends. First duplicates were removed. Title and abstract screening excluded irrelevant publications: non-urban settings, purely technical hydrological studies without GBI focus, and unrelated engineering fields. Full-text screening applied inclusion criteria: urban relevance, explicit barrier discussion, English language, and peer-reviewed status. A total of 577 studies met criteria and formed the synthesis evidence base, supplemented by authors’ expertise and cross-referencing.

Following the introduction and methods, core sections analyze GBI implementation barriers: environmental, social, economic, and governance/policy. The last section presents conclusions, recommendations, and research gaps.

## Environmental barriers

**Figure 2:**
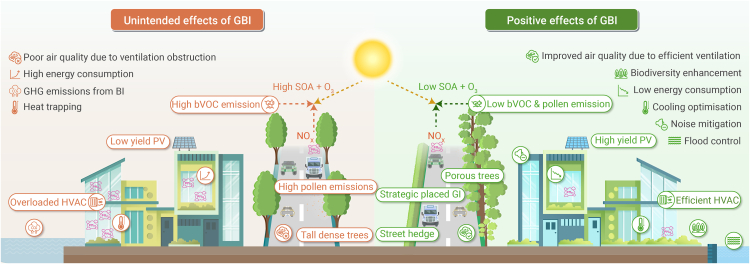
Contrasting potential outcomes of GBI at streets and household scale in an urban setting: unintended negative effects (left, red font) versus positive environmental benefits from strategic implementation (right, green font)

GBI has both positive and negative impact for mitigation of environmental problems at households and street scale as shown in Figure 2. To effectively address these contradictory impacts and optimize GBI functionality, nine environmental barriers (see GBI potential conflict with net zero goals to challenges linked to BI in urban environments) were identified that currently undermine successful implementation in urban contexts (Table 2). Table S1 provides a detailed summary of the case studies discussed below.

**Table 2:** Summary of environmental barriers, challenges, and potential solutions

Environmental barriers	Challenges	Overcoming challenges
Conflicts between GBI and net zero goals^a^	• PV competes for space with existing GBI • tree shading affects PV performance • PV and GBI net zero goals and other environmental benefits are poorly studied	• green roof and BI can be used as alternatives for PV placement • GBI can reduce cooling energy demand, reducing some PV needs • use modeling tools to evaluate carbon emission and sequestration potential for both GBI and PV
Siloization in urban GBI research and planning^b^	• decision-makers focus on single-issue problems rather than multifunctional solutions • fragmentation of green spaces impacts biodiversity, local climate, energy consumption, and well-being • green corridors studied mainly for biodiversity impacts, neglecting other benefits	• cross-disciplinary approaches and inclusive systems-based analysis • ecological production function based modeling assessment is suitable to identify shortfalls for mitigation actions • ES framework provides context for comprehensive assessment
GBI trade-offs^h^	• GBI benefits vary across spatiotemporal scales • a lack of integrated tools to holistically assess ecosystem service	• identify effective links between GI types using real-time monitoring • integrated modeling and remote sensing can address the trade-offs efficiently
Unintended consequences^c^	• certain vegetation emits bVOCs, leading to SA and ozone (O_3_) • wind-pollinated species increase respiratory issues with allergenic pollen • dense vegetation can disrupt airflow, trapping pollutants • GBI contributes to GHG emissions, reducing sequestration potential	• select species with low bVOC emissions, minimal allergenic traits, strong pollutant deposition • location specific design with hedgerows as alternatives to tall canopies in street canyons • periodic hydrological and soil management to limit GHG emissions
Plant adaptation and resilience in urban environments Pathway to resilient GBI via plant adaptation^g^	• urban environments push species beyond their realized niches • literature on plant stress tolerance in urban environments is limited • decision-making relies primarily on practitioners' expert judgment	• select plants based on phenotypic plasticity for resilience to stress such as heat and drought • introduction of species from surrounding biomes that match urban niches • integrate scientific data with tools support evidence-based practitioner decisions
BI integration challenges Challenges linked to BI in urban environments^i^	• BI presents dual character as benefits and risks • space constraints and urbanization restrict implementation of blue spaces • climate and geography limit BI options in arid or elevated regions	• design BI away from populous areas to avoid nighttime heat and maximize benefits • implement participatory planning and community involvement to address the space issue • restore rivers and daylight buried streams to reintegrate waterways
Achieving synergistic control^d^	• large street trees are beneficial to reduce UHI and noise but could restrict pollutant dispersion • high GI evapotranspiration is beneficial to UHI mitigation but worsens water shortage in dry seasons • BI is effective in UHI mitigation and water management but could release GHG and pollutant gases	• multifunctional planning that considers the needs of various urban challenges for synergistic management
Urban ventilation and air quality impacts^e^	• GBI can alter city breathability by increasing resistance to airflow, which restricts the dispersion of air pollution • poorly planned GI may obstruct building heating, ventilation, and air conditioning inlets, worsening IAQ	• integrate microclimate models to mitigate UHI while minimizing airflow blocking • use machine learning methods to understand the nexus between GBI, city, and building ventilation
Thermal resilience and microclimate^f^	• research focuses on cooling effects rather than adaptation dynamics • most studies examine GBI effects mainly on general populations, neglecting vulnerable groups • surface temperature alone inadequately represents microclimate conditions	• integration of environmental factors with human comfort considerations • development of city-scale overheating risk warning systems with focused strategies for vulnerable groups • adaptation of digital twins, remote sensing, and AI for analyzing microclimate heat mitigation strategy

Each barrier is discussed in detail in the following subsections, outlining key issues and mitigation strategies.

a See GBI potential conflict with net zero goals (Figure S1; Table S2).

b See siloization in urban GBI studies (Figure S5; Table S3).

c See unintended consequences (Figure S6; Table S4).

d See conflict control among urban environmental challenges (Figure S8).

e See urban ventilation (Table S5).

f See thermal resilience (Table S7).

g See pathways to resilient GBI via plant adaptation (Table S8).

h See GBI trade-offs.

i See challenges linked to BI in urban environments.

### GBI potential conflict with net zero goals

While net zero and GBI initiatives share many common goals, including climate resilience efforts, renewable electricity generation can compromise GBI initiatives (Table S2). Declining costs of solar photovoltaic (PV) systems have accelerated their global adoption as the fastest growing renewable energy source.^72^ Despite their benefits, PV installations may compete with existing land uses, including wetlands, parks, and forests, which can result in biodiversity and habitat loss.^73^^,^^74^^,^^75^ For example, in Australia, homeowners with rooftop PVs are advised by local solar companies to reduce tree shade to maximize energy output.^76^ PV performance is indeed affected by tree shade, which varies with species, height, and crown width.^77^ Such studies are often used to justify tree pruning or removal when shading PV panels, with 30 US states permitting vegetation removal^78^ despite trees’ carbon absorption^79^ and greenhouse gas (GHG) reduction benefits.^80^ A German study showed that trees only reduce solar roof radiation by 1% after 20 years of growth,^81^ although some argue PV systems provide greater carbon reduction than trees.^82^

Achieving a truly sustainable development rather than merely net zero development requires more than prioritizing PV over GI based solely on GHG reduction considerations.^83^ The multiple benefits of a GI (Table 1) need to be considered when balancing against the benefits of PV (Figure S4). Therefore, the questions are: how can we achieve a net zero energy development that is also green? How can urban planners, city councils, and homeowners decide which GI elements to implement, including which tree species to plant and where, while maximizing solar energy production?

GBI reduces urban heat and provides cooling effects, thereby lowering building energy demand. Simulations in representative neighborhoods show that tree shading can reduce annual cooling needs by 2% under today’s climate and by 5% in projected 2050 conditions,^84^ which may also reduce the required size of solar panels. In Montreal, a case study for a building demonstrates that urban trees alone can mitigate 17% of carbon emissions over a period of 60 years, without even considering on-site electricity generation.^85^

Strategic integration of GI with on-site solar power requires careful planning of tree species selection, placement, and PV positioning. In England, for example, solar farms are predominantly sited in human-modified landscapes, including in urban settings, instead of in sensitive ecological areas and designated conservation sites, indicating environmentally considerate placement.^86^ A framework integrating rooftop PV with GI under current and future climate scenarios was developed using urban modeling, energy simulation, and carbon sequestration analysis.^84^ Building-integrated photovoltaics with green roofs and facades offer mutual benefits: vegetation cools panels, improving efficiency and optimizing space, although performance depends on panel-plant distance, installation conditions, species selection, and microclimate.^87^ In tropical climates, plants combined with polycrystalline PV modules increased efficiency by about 2% at a 15° inclination.^88^ Similarly, systems using sedum plants delivered a 1.6% gain when panels were mounted at 1.01 m with a 3° south-facing inclination.^89^ Green roofs increase solar installations yields by 5%–15% through reduced albedo.^90^ GI-PV integration depends on context, requiring tailored solutions for local climate, vegetation, and building conditions.

Floating PV systems installed on human-made water bodies such as reservoirs and irrigation ponds offer opportunities for urban BI while maximizing land efficiency.^91^ For example, Singapore has implemented urban floating solar farms, capable of offsetting more than 4,000 tons of CO_2_ annually^92^ demonstrating the potential for large-scale deployment within dense city environments. However, research on their integration into urban design remains scarce, and studies on their impacts on water quality and aquatic ecology are limited despite growing implementation.^93^

To achieve net zero developments, GBI, particularly trees, are often perceived as detrimental to the PV systems. However, solar developments can be achieved through thoughtful planning without sacrificing urban landscapes. The vegetation can provide cooling and even improve PV efficiency. Incorporating PV into ecological restoration of urban landscapes also offers promising opportunities, although further research is needed. Decision-makers should embrace technological innovation to maximize the co-benefits of PV and GBI integration.

### Siloization in urban GBI studies

Scientific silos (or knowledge compartmentalization) relate to the organization of scientists into discrete communities with minimal interaction. Siloization can slow the propagation of scientific information and impede understanding of inter-relationships of complex systems.^94^^,^^95^ The siloization of GBI research has been broadly contextualized as the tendency to respond to single-issue problems rather than exploring the multifunctional solutions provided by ES of GI.^6^ Urban green corridors (GCs), defined as linear landscape features that allow biological migration and energy exchange between large patches of ecological source areas,^96^^,^^97^ provide an illustrative example. GCs are commonly proposed as a means to improve ecological connectivity,^96^ counteracting the effect of green space fragmentation, which is occurring alongside the loss of these spaces during urbanization.^98^ This trend is associated with development patterns, related to the intensification of infrastructure^99^ as well as morphological decisions that are dependent on extensive transportation networks.^100^ The resultant habitat fragmentation has well-established implications related to biodiversity loss, associated with the loss of habitat, migration routes, and species connectivity.^101^ However, this type of fragmentation also adversely influences local climates (by increasing UHIs),^102^ energy consumption patterns,^103^ landscape esthetic quality (as revealed by negative correlation between fragmentation index and esthetic quality metrics),^104^ human well-being (life satisfaction increases positively with vegetation and less soil sealing),^105^ and blue landscape availability.^106^ GCs can help mitigate all of these impacts.

Currently, much of the work on GCs has explored the important biodiversity impacts of connecting larger green spaces,^96^ often excluding analysis of other impacts mentioned above. A bibliometric analysis map (Figure S5) shows that the main body of scientific research is focused on the relationships between GCs, biodiversity improvement, and habitats for flora and fauna. Life quality including recreation and accessibility issues was less studied. However, there are many thematic categories that are far less examined in the context of GC studies, e.g., carbon storage and thermal exposure reduction,^5^ where GCs have been identified as one of the underrepresented GBI elements in heat mitigation studies. Moreover, a close examination of the interconnections between the thematic categories (i.e., the strength of their links) indicates that most of them were studied in silos (section S2): only five studies have examined more than three solutions (Table S3).

All five studies were published after 2018; two were conducted in China (Beijing^107^ and Nanjing^108^), two in Cusco, Peru,^109^^,^^110^ and one in Cagliari, Italy.^111^ Three deployed some type of landscape analysis utilizing remote sensing datasets (e.g., Land Use maps, Land Cover maps, Land Surface Temperature), while two studies were based on literature and online tools.^109^^,^^110^ In two studies,^108^^,^^111^ energy flow patterns and species along GCs were examined using least-cost path analysis and InVEST software to characterize wildlife movement resistance. The first of these five studies tackled siloization through ES concept application, assessing water storage/purification, local climate regulation, and esthetics before and after construction of a network of artificial lakes, wetlands, and parks along Beijing’s Yongding River.^107^ Ecological production functions identified shortfalls in ES production using mathematical models^112^ to connect ecosystems, stressors, and management actions. Coupling green and built infrastructure can achieve desired multifunctionality, where inadequate ecosystem function exists. These studies demonstrate how cross-disciplinary approaches allow systems-based analysis of GC enhancement measures, revealing complementary or unintended consequences.

More integrative approaches to GI impacts are essential, especially for GCs. Single metric performance assessment undermines their manifold benefits; ES frameworks provide the required context for comprehensive evaluation.

### Unintended consequences

GBI is gaining prominence for addressing urban challenges such as air pollution, heat, and biodiversity loss; however, poorly designed GBI interventions can cause unintended negative impacts.^4^^,^^113^ Certain vegetation types emit bVOCs, leading to salicylic acid (SA) and O_3_ formation when interacting with urban pollutants, such as NO_*x*_,^113^ while allergenic pollen can trigger respiratory illnesses.^114^^,^^115^ Dense vegetation can also disrupt airflow (see urban ventilation)^116^^,^^117^ and some green systems may emit GHGs such as methane (CH_4_) or nighttime CO_2_, lowering the sequestration potential (Table S4).^118^^,^^119^

bVOC emissions vary by species and stress, with high emitters such as *Populus tremula* increasing O_3_ risks, while low emitters such as *Acer campestre* may help reduce it when combined with high deposition traits.^120^^,^^121^ Stressors, such as heat, drought, and pruning can boost emissions by three to five times,^113^^,^^122^ and since reactive sesquiterpenes drive SA formation,^123^ choosing and maintaining low-emitting species is imperative.^4^^,^^124^

Urban GI, particularly wind-pollinated species, increase respiratory issues, with urban residents facing up to 20% higher allergy rates than rural populations.^114^^,^^125^ Air pollutants increase pollen allergenicity,^126^ for example, *Betula pendula* produces more potent allergens under polluted conditions.^127^ Climate change intensifies pollen seasons with emissions increasing by 200%.^128^ Urban planners in cities such as Sacramento, USA, London, UK, and Christchurch, New Zealand, favor often male dioecious trees (e.g., male-deodar cedars) to avoid seed or fruit litter, creating allergenic, monodominant canopies.^115^^,^^129^^,^^130^^,^^131^ These patterns increase public exposure, particularly for vulnerable groups, while synergistic effects between pollen and pollution amplify health impacts.^114^^,^^127^ Despite GBI’s promotion, allergen-related health risks are often ignored, exposing gaps in species selection and risk assessment.^115^

As discussed in urban ventilation, planting layout matters: at the ground level in street canyons, tall and dense tree canopies can obstruct vertical ventilation, leading to elevated levels of air pollutants.^116^^,^^117^^,^^132^^,^^133^ However, vegetation with optimized porosity and leaf area density can enhance mixing and filtration, particularly in open-road settings or with well-designed barriers.^117^ Symmetrical rows can hinder airflow, while staggered or one-sided designs may better support noise reduction,^134^ ventilation, and pollutant capture.^116^ Yet, urban planning often neglects these design nuances, increasing air quality risks in dense, high-traffic zones.^133^

As highlighted in pathways to resilient GBI via plant adaptation, water management and poorly designed systems can cause significant GHG emissions, especially in urban wetlands and irrigated green spaces.^118^^,^^119^ CH_4_ fluxes in constructed wetlands can be up to 10 times higher than in natural ones, and CO_2_ accumulation is common in poorly ventilated areas, particularly at night.^135^^,^^136^ Nutrient runoff further exacerbates emissions, yet these disservices are often overlooked in GBI assessments.^117^^,^^137^ Additionally, urban vegetation’s actual carbon sequestration is limited by plant species and size, site, and maintenance factors, which may offset expected benefits.^138^^,^^139^^,^^140^

Despite potential unintended impacts, GBI remains a powerful tool for urban resilience when informed by environmental evidence and local context (Figure S6).^120^ A key mitigation strategy is selecting species with low bVOC emissions, minimal allergenic traits, and strong pollutant deposition capacity. Tools such as OPALS (Ogren Plant Allergy Scale) and regional emission inventories support the selection of insect-pollinated, female, or low-allergenic species near sensitive populations, while also boosting biodiversity by providing habitat and resources for pollinators.^115^^,^^125^^,^^127^ Small, rough, and complex leaves further enhance pollutant capture without hindering airflow.^116^^,^^120^

Site-specific vegetation design is also critical. In street canyons, hedgerows offer an alternative to tall canopies, improving airflow and pollutant trapping.^116^^,^^133^ In open areas, wide, dense and optimal porous barriers are effective to balance filtration and air movement.^120^^,^^141^ Modeling tools can be used here to support GI design decisions under varying conditions in urban environments.^133^

Hydrological and soil management is equally important. Rain gardens, bioswales, and wetlands should avoid prolonged saturation to limit CH_4_ emissions from anaerobic activity.^118^^,^^119^ Over-irrigation and nutrient-rich runoff can be reduced with well-drained soils, plants adapted to wet-dry cycles, and reduced fertilization. Modeling helps optimize performance across environmental scenarios,^37^^,^^49^ enabling GBI to achieve environmental and health goals while minimizing trade-offs.

As further discussed in conflict control among urban environmental challenges, these unintended impacts often manifest as conflicts among urban environmental challenges. Moreover, GBI trade-offs represents broader trade-offs and planning barriers that hinder integrated urban solutions.

### Conflict control among urban environmental challenges

GI implementation in dense, and socially complex urban areas is often hindered by fragmented planning processes and mono-functional design strategies that focus on single objectives while neglecting the complexity and interdependency of urban systems. This can lead to trade-offs, where interventions addressing one challenge may fail to solve or even exacerbate others.^5^^,^^142^^,^^143^^,^^144^ As discussed in siloization in urban GBI studies and supported by various studies, this underscores the need for integrated, multifunctional planning that considers strategies for synergistic management.

Recent GBI research has shifted from biodiversity focus to applied strategies addressing climate adaptation, stormwater, air quality, UHI, public health, and EJ (see Figures S3 and S7). Figure S8 visualizes the multifaceted impacts of GBI on key urban challenges. The major barrier to achieving synergistic control arises from the mixed or even conflicting outcomes that GBI interventions can produce on specific urban challenges. This is particularly evident in the control of UHI, air pollution, and water management. GI can effectively mitigate UHI effect^145^^,^^146^^,^^147^^,^^148^^,^^149^ and reduce anthropogenic noise,^150^ with the scale of intervention playing a critical role. Large tree canopies and leaf area densities are particularly effective in achieving significant UHI and noise reductions. For example, a case study from Montreal, Canada, found that the noise level decreased with an increase in the mean volume of tree crowns and canopy.^151^ However, these features often conflict with the requirements for efficient air ventilation and pollutant dispersion in street canyons (unintended consequences and urban ventilation), leading to deteriorated air quality at roadsides.^152^^,^^153^^,^^154^ Further, while plants with high evapotranspiration rates are beneficial to UHI mitigation, they also require substantial irrigation^155^ and result in significant water losses, particularly during dry seasons.^156^^,^^157^ Similarly, BI is effective in UHI mitigation and water management,^52^ but could also cause air quality issues due to degradation of organic matter, particularly when high sediment accumulation is present.^158^^,^^159^^,^^160^

The above control conflicts among urban challenges are rooted in their different requirements. While UHI and noise mitigation require large tree canopies and high leaf area densities, effective pollutant dispersion in street canyons typically requires open space. However, studies have shown that GI could also act as porous barriers to stop the dispersion of traffic emissions to roadside pedestrian breathing zones when designed appropriately.^161^^,^^162^ Recent computational fluid dynamics analyses showed that the uneven distribution of trees in street canyons facilitate horizontal pollutant transport, offering a novel strategy for redirecting pollution hotspots away from densely populated areas.^163^ Such synergistic design considerations can help preserve the UHI and noise mitigation benefits of GI while minimizing, or even reversing, its negative impacts on pollutant dispersion.

To resolve the conflicts between vegetation evapotranspiration for UHI mitigation and water scarcity, strategies should prioritize shading and high-albedo effects over evapotranspiration. Studies have reported that the combined solar and surface radiation can cause 10°C–20°C hotter than ambient air temperature.^164^ Therefore, selecting native and drought-tolerant species that offer ample canopy cover and effective solar reflectance can provide UHI mitigation benefits while minimizing irrigation demands. Finally, the unintended GHG and pollutant emissions of BI during UHI mitigation can be effectively solved through stricter regulatory measures aimed at controlling eutrophication and reducing organic matter inputs.^165^

The synergistic management of various urban challenges using GBI requires systemic changes in planning, evaluation, and governance. Conflicts arising between key functions, such as large shade for UHI mitigation versus open space for pollutant dispersion, highlight the significance of integrated and holistic design strategies. Key principles for addressing these challenges include species selection and planting design for balanced/synergistic UHI and air pollution control, prioritization of shade and albedo over evapotranspiration for UHI mitigation, and effective sediment management in BI systems.

### Urban ventilation

As highlighted in unintended consequences, GBI interacts with natural and mechanical processes influencing indoor-outdoor air exchange in the built environment.^4^^,^^5^^,^^166^^,^^167^ Given the dynamic spatiotemporal variations that occur in cities, the GBI and urban form interactions, can impact the performance of natural ventilation or HVAC (heating, ventilation, and air conditioning) systems, which may also indirectly affect GBI health as it influences microclimatic conditions. This has been evidenced in different context and scales: from an individual building,^168^^,^^169^ to essential urban infrastructure including underground transport tunnels,^170^ and in city-scale ventilation (Tables S5 and S6).^171^ The bi-directional relationship between these factors have collectively received limited attention due to the complexity of GBI-GI interactions.

HVAC systems in particular provide a vital service to infrastructure across cities^172^ despite requiring substantial energy, and associated operational carbon emissions, providing thermal comfort and good indoor air quality (IAQ).^172^^,^^173^ At the same time they dispel heat externally and contribute to the UHI effect, in turn creating a detrimental loop between the HVAC system’s cooling load^174^ and UHI,^175^ which is further exacerbated by hotter climatic conditions.^176^ In dense high-rise urban environments, the dispersion of emissions relies heavily on wind-driven ventilation, described as the “breathability” of a city. This relates to the resistance to air flow created by the features and design of the urban geometry.^177^ The addition of GBI can alter city breathability where GI can increase the resistance to airflow while BI can create the opposite effect (see conflict control among urban environmental challenges).

Buildings also “breathe,” inhaling via HVAC system inlets, open windows and doors, and exhaling via exhausts and other outlets. Where the ventilation of city streets has been positively or negatively impacted by the presence of GBI, this in turn impacts on building ventilation via increases or decreases in IAQ,^169^ energy consumption,^174^ and humidity levels.^178^ Buildings with higher energy performances, sufficient air tightness, and efficient HVAC systems could see greater energy demands associated with increased ventilation rates to maintain acceptable IAQ.^179^ Poorly planned GI obstructing the mechanical ventilation system’s fresh air intake could lead to worsening IAQ if the supply of outdoor air is restricted. GI such as green walls and avenue planted trees have also been shown to restrict the vertical dispersion of air pollution.^152^^,^^180^ Both studies have highlighted the potential negative impact on street level air quality, yet most exposure to air pollution takes place predominantly indoors, and ventilation of buildings often originates from roof-level fresh air intakes. Therefore, GBI has the potential to improve IAQ and reduce heat and humidity loads along the source-receptor pathway of the HVAC intake. This consideration is, however, consistently overlooked in building and city design.

Understanding the GBI-urban ventilation nexus can overcome potential challenges posed by GBI implementation. Where negative impacts are highlighted from GBI designs, local solutions can be created to address insufficient urban ventilation. City ventilation can be substantially altered by modifying air flow patterns using urban design features,^181^ machine learning methods optimizing new building designs in urban areas,^182^ and GI.^4^ Moreover, machine and deep learning methods using mapping techniques have a role in implementing optimal GBI solutions^33^ and could be integrated with urban microclimate models to ensure UHI mitigation and minimize airflow restrictions.^183^ Previous investigations have also highlighted that the design, orientation, and location of building fresh air intakes can be significantly impacted by street level emissions altering IAQ.^184^^,^^185^ Green roofs significantly minimize NO_*x*_, CO_2_,^186^ O_3_,^187^^,^^188^ and particulate matter concentrations^189^ at roof-level, reducing pollutants entering building air intakes. HVAC filters on a Portland, USA, green roof removed up to 14% of incoming ozone depending on humidity conditions, although with higher microbial and VOC loadings than white roof filters.^187^ Similarly, a lightweight extensive green roof on a school building in New Belgrade, Serbia, reduced ambient PM_10_, PM_2.5_, and PM_1_ by up to 22.5%, 28%, and 31.8%, respectively, compared with a reference roof.^189^ Future work should develop GBI frameworks integrating building and city ventilation concepts.

The GBI-urban ventilation nexus, particularly involving HVAC systems, remains overlooked in urban design and management. GBI design needs to be considered within its wider context to maximize co-benefits and avoid unintended negative impacts.

### Thermal resilience

GBI is widely recognized for improving urban microclimates and has emerged as a vital strategy for improving cities’ thermal stress tolerance against heat exposure risks driven by rising climate change and urbanization. The key categories driving the enhancement of thermal resilience are social, ecological, and technological domains.^190^ Although many studies focused on individual aspects, lack of clarity regarding the interaction of humans with the urban environment during designing thermally comfortable adaptation spaces and developing strategies to improve thermal resilience.^191^

Existing studies have shown that the synergistic effect of GBI could lower surface and ambient air temperature reducing the UHI effect (Tables S6 and S7), which in turn enhances thermal comfort and reduces heat risk. Thermal adaptation in humans mainly involves three processes: physiological, psychological, and behavioral adaptations.^192^ Thermal adaptation plays a key role in users’ comfort perception in GBI environments,^193^ while research predominantly focuses on cooling effects rather than adaptation dynamics in complex urban settings. Few studies have explored how physiological parameters respond to changing thermal conditions in GBI environments.^194^ In practice, the Paris Oasis Schoolyard Programme transformed schoolyards into urban cool islands accessible to communities during heatwave days.^195^

Greening designs, plant species selection, and water body distribution may differentially impact overheating risks for vulnerable populations, particularly older people^196^ and children.^197^ Despite strong evidence of heat-related vulnerability in these demographics, current research predominantly examines GBI effects on general populations.^193^^,^^198^^,^^199^

To analyze the influence of GBI on thermal environments and human comfort, data are collected through three principal approaches: subjective questionnaires, direct environmental/physiological parameter measurements, and remote sensing techniques (Table S7). While questionnaires and direct measurements provide valuable insights, they often yield limited spatial information, undermining comprehensive analysis.^200^ Remote sensing offers an advantage by providing spatial distribution data of GBI across multiple large areas^201^ including surface temperatures through inversion techniques.^202^ However, surface temperature alone inadequately represents complete microclimate conditions. Comprehensive assessment of GBI’s thermal impact requires additional parameters such as humidity and wind speed, which remain challenging to obtain simultaneously through remote sensing.^201^^,^^202^^,^^203^

To overcome urban thermal resilience challenges, future GBI research and planning must shift from measuring cooling effectiveness to understanding and quantifying human thermal adaptation. A comprehensive strategy should integrate environmental factors with human comfort considerations to enhance adaptive capacity and resilience. Data collection from field studies on outdoor thermal comfort needs to extend to a wider range of demographic groups, including older people and children. Digital twins, remote sensing, and artificial intelligence can leverage integrated GBI datasets to model thermal environments and user needs, supporting sustainable urban planning through data-driven insights.^203^^,^^204^ Developing city-scale overheating risk warning and mapping systems, for example, human-centric digital twins^205^ that incorporate GBI information would enable proactive adaptation strategies, helping urban residents, particularly vulnerable populations, to avoid dangerous heat exposures while maximizing the effectiveness of implemented GBI solutions.

Future research on thermal resilience through GBI should advance three key areas: comprehensive human-urban interaction across diverse demographic groups, refined methods for evaluating heat exposure in GBI environments, and emerging AI-powered technologies for comprehensive environmental assessment and intervention solutions to mitigate urban heat risks.

### Pathways to resilient GBI via plant adaptation

GBI solutions aim to adapt cities to climate change and protect citizens from physical and mental health impairments.^4^^,^^206^ However, the GBI tools comprise living organisms subject to physiological and metabolic limitations from climate extremes.^207^^,^^208^ The potential demise of GBI interventions is often overlooked in urban adaptation, which tends to ignore the fact that the same plants, animals, and other organisms are already showing signs of decline in natural ecosystems worldwide.^209^^,^^210^^,^^211^^,^^212^^,^^213^^,^^214^

Throughout evolutionary history, organisms have been selected to cope with, survive, and reproduce under heatwaves, droughts, floods, and other sources of acute and chronic stress.^215^^,^^216^ These adaptations define a species’ realized niche—the range of environmental conditions where they naturally occur.^217^ While many organisms can thrive under a broader set of conditions—their fundamental niche^218^—all species have tolerance limits beyond which they cannot reproduce or survive. Urban environments frequently push species beyond their realized niches, yet the global success of cosmopolitan species in cities worldwide demonstrates species’ remarkable resilience to diverse urban conditions.^219^^,^^220^^,^^221^

As the cornerstone of GBI, plants are the focus of this section.^218^ Plants, as sessile organisms unable to seek shelter from environmental stress, rely on adaptive strategies.^222^ The fitness of plants in urban environments depends on morphological and functional phenotypic plasticity and adaptability, influencing their resilience to stress.^223^^,^^224^ Despite the importance of species suitability for GBI success, literature on plant stress tolerance in urban environments remains limited.^225^ Consequently, decision-making continues to rely primarily on practitioners’ empirical knowledge,^226^ a valuable but insufficient approach for promoting GBI biodiversity and resilience. There is also a risk that GBI species selection for urban planting comes from a “recipe book” of species long used in urban settings, but which may no longer be suitable under rapidly changing climatic conditions.

Understanding plant morphological and physiological responses to urban conditions under climate change is essential for selecting species that effectively provide ES. Phenotypic plasticity—one genotype-driven ability to produce various phenotypic variations in reaction to environmental conditions^227^^,^^228^—offers significant advantages, allowing plants to fine-tune their form and function to cope with local urban challenges.^229^ The plastic responses include thickened leaf cuticles, reduced leaf area and stomatal density, facultative shifts in photosynthetic metabolism, and hydraulic system adjustments that help plants cope with heat, drought, and high light intensity.^230^^,^^231^^,^^232^ Some species display remarkable stress tolerance, such as *Tipuana tipu* (Leguminosae) trees in São Paulo, Brazil, which increased photosynthetic and growth rates during one of the city’s worst recent droughts.^219^ While adaptation represents another pathway, it typically requires multiple generations under selective pressure and is therefore less likely to occur within time frames relevant to urban environments,^233^ except for species with short life cycles that already adapted to urban conditions.^234^^,^^235^^,^^236^^,^^237^^,^^238^^,^^239^ Urban plantings are typically undertaken with standard stock material and there is little or no scope for natural genetic variation to influence future generations of plants.

Species migration represents a third pathway for organisms responding to stress.^240^ Natural plant migration is slow, spanning decades or centuries, making it unlikely to keep pace with rapid urban environmental changes.^241^ In urban contexts, humans act as migration agents by introducing species through GBI implementation. While plant selection has historically been dictated by cultural preferences, market interests, and landscape design trends, emerging approaches now focus on introducing species from surrounding biomes that correspond ecologically to urban niches—a more efficient strategy for enhancing GBI resilience.^242^

Phenotypic plasticity, adaptation, and migration represent the three primary mechanisms by which species establish and occupy new niches (Table S8)—essential processes that must be incorporated into GBI planning and management to achieve both immediate benefits and sustained resilience.^243^ This integration remains particularly challenging in cities located within highly biodiverse regions where species knowledge by urban decision-makers is limited. However, practical shortcuts can accelerate appropriate species selection for urban GBI niches. These include leveraging basic scientific information (species descriptions and identification keys) alongside ecological studies of successional processes and plant strategies, providing invaluable insights into species’ capacity to tolerate current and future urban climate conditions.^244^ Without utilizing at least one of these three pathways, species may face local extinction through landscape transformation, undermining GBI effectiveness.^243^^,^^245^

### GBI trade-offs

GBI implementation often involves trade-offs (Figure S8) and additional barriers, multi-scalar, technical, and institutional, that prevent synergistic planning.

GBI benefits occur across spatiotemporal scales that often misalign with targeted environmental challenges.^26^^,^^246^ Spatially, GBI is commonly deployed in fragmented urban areas, while essential ecological processes function at broader scales.^247^^,^^248^ This spatial mismatch, coupled with weak integration into city-wide planning, limits effectiveness.^249^ For instance, scattered green roofs or pocket parks provide local cooling but may not mitigate UHIs (see thermal resilience), and GBI in downstream flood zones can be ineffective without upstream coordination.^250^^,^^251^ Temporally, GBI often delivers ES slower than urban crises unfold. Static assessments overlook temporal dynamics, compromising long-term evaluations.^252^ Some benefits arise quickly (e.g., air quality, noise, cooling) than others (e.g., biodiversity).^117^^,^^253^ Planning rarely accounts for time needed for full functionality (e.g., tree maturity, soil development), hindering co-benefit integration and political support. This calls for long-term, multi-scalar planning and vision.^246^

A lack of integrated tools to holistically assess ES synergies and trade-offs remains a key gap.^254^ GBI planning often relies on siloed (see siloization in urban GBI studies), mono-functional approaches, lacking the interoperability to evaluate interconnected processes and trade-offs (e.g., hydrology, urban climate, pollutant dispersion, and thermal comfort).^255^^,^^256^ This is compounded in cities with scarce or incompatible data. Even studies emphasizing multifunctionality tend to map priority areas for GBI interventions without accurately accounting for local needs.^257^^,^^258^^,^^259^ For instance, selecting a GBI with broad co-benefits to target multifunctionality usually relies on generalized criteria and suitability analysis, lacking context-specific design.^257^ This reliance on non-local assumptions may reduce relevance.^254^ Recent work illustrates the value of context-specific design. Open-air urban farming roofs in Shenzhen, China, could produce up to 7.44 kg of vegetables per m^2^ annually and reduce upstream energy and water footprints by a factor of 4.5.^260^ This example highlights both the potential and the trade-offs of farming roofs, which depend on local conditions, resources, and governances. Without integrative metrics, GBI decisions are fragmented, hampering planning, and increasing unintended trade-offs.

A key response to scale mismatches is multi-scalar environmental planning aligning GBI interventions from the block and neighborhood levels to watershed and metropolitan scales, enhancing ecological connectivity and integrated risk management. GBI networks should combine regional strategies (e.g., greenbelts, GCs) with local solutions (e.g., rain gardens, street trees).^261^^,^^262^ A blend of scales and configurations is needed to optimize co-benefits.^26^^,^^262^ Addressing spatial fragmentation requires cross-sectoral and multilevel governance agreements.^263^ Further research is needed to identify effective links between GBI types, supporting governance mechanisms, and resulting multifunctionality benefits.

Emerging planning frameworks use spatial multi-criteria methods to incorporate urban ES into spatial decision-making, combining environmental and social layers to rank priority intervention areas in cities and integrating equity considerations.^256^^,^^257^^,^^264^ These approaches map spatially explicit trade-offs at multiple scales and generate priority areas based on high-resolution geospatial analysis. However, such decision-making approaches are only as good as the data used to underpin them.

Technology and modeling strategies can partially overcome data or information limitations. Affordable sensing technologies and IoT platforms (e.g., air quality monitors, soil moisture sensors, urban rain gauges) enable real-time GI monitoring, supporting adaptive management.^161^^,^^265^^,^^266^^,^^267^ Biomonitoring with bioindicators (e.g., lichens, leaves, and microorganisms) can help infer pollutant loads and ecological health.^268^^,^^269^^,^^270^ Tools such as InVEST quantify services (e.g., thermal regulation, runoff retention, carbon sequestration, noise mitigation) under various GBI scenarios.^271^^,^^272^^,^^273^^,^^274^ Finally, integrating these with remote sensing, GIS, and environmental models, will support better GBI siting and selection.^67^^,^^256^^,^^275^^,^^276^

Addressing GBI implementation barriers demands resolving spatial-temporal scale mismatches through multilevel planning, integrated modeling, and high-resolution spatial data, including social and demographic data, to align localized interventions with broader ecological processes and long-term urban resilience objectives.

### Challenges linked to BI in urban environments

Urban BI encompasses both natural and artificial water-related elements within city boundaries, such as canals, lakes, ponds, rivers, wetlands, seas, and constructed drainage features that mimic natural hydrological processes.^5^^,^^6^^,^^277^^,^^278^ These water bodies support human well-being, flood mitigation, cooling, and recreational and cultural benefits in cities.^279^^,^^280^^,^^281^ Existing literature on BI challenges and solutions remains limited, especially regarding coastal systems. Space constraints in densely built areas often restrict the implementation and expansion of BI to a greater extent than most GI.^282^ Management challenges for BI include balancing flood risks, erosion control, and maintaining attractive, accessible spaces for urban dwellers.^278^ Climate and geography often shape BI options; cities in arid regions tend to make use of smaller water features since water is scarce, often pumped from elsewhere, and evaporation rates are high. Cities on elevated terrain will tend to have smaller water bodies compared with urban centers in low lying catchments where large rivers and lakes are more common. Cities with river catchments crossing national borders face additional political complexities beyond typical GI challenges in terms of managing water (high and low) flows and water quality. Cities relying on groundwater face additional complexities as these systems often cross jurisdictions and have unclear boundaries.^26^

BI often presents a dual character as both a benefit and a risk. While rivers and seas enhance well-being through access and visual connection,^283^ they also increase flood risks.^284^ Steep walls and high flood defenses designed to reduce risk will also reduce direct accessibility for the public, along with the visual connection of the public with these natural features. In tropical areas, there can also be health risks from vector-transmitted illnesses, such as malaria, or dengue, where insect vectors use any pooled water for breeding, from plant pots up to reservoirs. Solutions must balance protection with public accessibility to maximize multifunctional benefits.

A range of solutions can improve the implementation of BI in urban settings. Stronger governance and better policy integration are necessary to prioritize BI benefits, requiring national, and international coordination to manage water flows and water quality.^285^ At a local scale, participatory planning and close engagement with residents are often critical for successful long-term implementation of local BI projects, fostering local capacity, appreciation, and stewardship,^277^ such as caring for a stretch of canal or urban stream.^286^

While sharing knowledge on best management practices, upskilling personnel, and improving understanding among stakeholders^287^^,^^288^^,^^289^ are common solutions across multiple types of GBI, these need to be specific to management of water and BI since the technical challenges are much greater around issues such as managing risk of disease transmission, or alleviating flood risk. Sharing lessons learned and data from pilot and demonstration projects is also recommended.^290^

BI planning must consider local context and can leverage historical and cultural connections to water, as demonstrated by the revitalization of traditional urban irrigation canals in Spain.^291^ River restoration enhances existing BI by reintegrating waterways into urban environments through techniques such as “daylighting” buried streams, creating self-sustaining systems that deliver ecological benefits, flood control, recreation, and social value.^292^^,^^293^ In space-constrained coastal cities, offshore NbS and hybrid approaches combining GBGI enhance resilience and multifunctionality, as exemplified by vegetated water retention basins that support biodiversity.^294^^,^^295^^,^^296^

Designing blue spaces with an emphasis on multifunctionality can maximize benefits, such as ecological connectivity, public space, and recreation.^278^ Features such as fountains, and play areas with water sources create cooling and social hubs in urban spaces.^297^ Improving physical and esthetic access to blue spaces, such as bathing platforms and waterfront walkways, along with better water quality, significantly boosts public engagement and the benefits that city dwellers experience from BI.^298^^,^^299^

In summary, addressing these issues for BI requires coordinated governance across scales, community engagement, and technical expertise sensitive to local contexts. Strategic approaches should include multifunctional design, natural water system restoration, and integration with other infrastructure types.

## Social barriers

**Figure 3:**
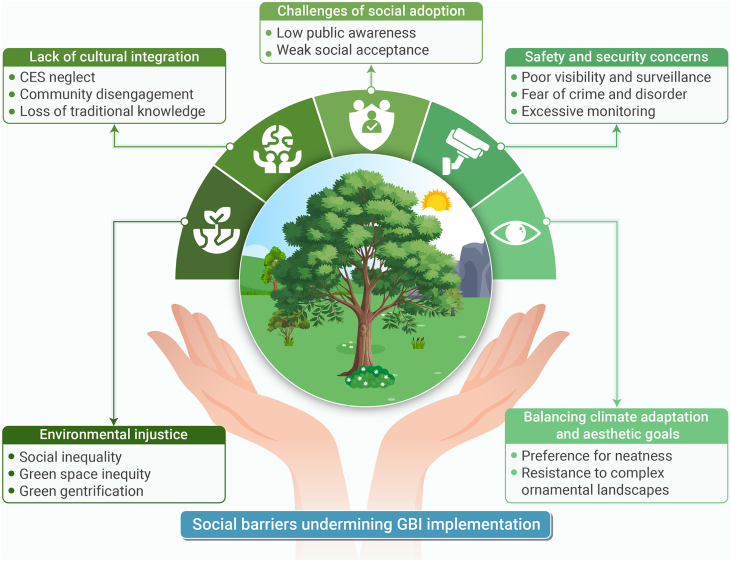
Five less-discussed social barriers that hinder effective urban GBI implementation These barriers include environmental injustice, cultural disconnect, social adoption challenges, safety concerns, and esthetic conflicts. Without inclusive and adaptive planning, they can undermine long-term GBI outcomes.

This section synthesizes five key social barriers: environmental injustice, cultural misalignment, low public adoption, safety concerns, and esthetic resistance (Figure 3). A consolidated summary of their core challenges and corresponding strategies is presented in Table 3 and a summary of discussed case studies in Table S1.

**Table 3:** Summary of social, economic, and governance barriers to GBI implementation, along with corresponding potential solutions

Barriers	Challenges	Overcoming challenges
**Social barriers**		

Environmental injustice^a^	• historical segregation and systemic inequalities • unequal distribution of green spaces and ecosystem benefits • green gentrification displacing vulnerable populations • exclusion of marginalized groups from decision-making	• integrate justice frameworks into GBI planning and design • prioritize investments in underserved communities • enable early and meaningful community participation • link local actions to broader multi-scalar governance
Cultural disconnection^b^	• under-recognition of the CESs • exclusion of local and traditional knowledge • poorly contextualized GBI undermining heritage • ignoring culturally specific landscape preferences	• integrate CESs into GBI frameworks • embed local and traditional knowledge into planning • apply heritage-sensitive and culturally adaptive design • foster co-creative public engagement
Public adoption^c^	• low public awareness of GBI benefits • misalignment between expert planning and community needs • lack of early participation weakening stewardship • disconnection between technocratic approaches and local realities	• embed continuous community participation from early stages • design GBI to enhance perceived safety and usability • link GBI benefits visibly to daily life • use environmental education to build trust and ownership
Safety concerns^d^	• vegetation creating surveillance blind spots • obstructed visibility at entry points • poorly monitored parks linked to antisocial behavior • crime stigma reducing GBI use • over-securitization limiting access	• apply crime prevention through environmental design (CPTED) principles • use smart lighting, CCTV, and IoT monitoring discreetly • promote participatory design and local stewardship • balance safety needs with social inclusivity
Esthetic tensions^e^	• public preference for ornamental over functional landscapes • functional GBI perceived as messy or neglected • policies favor visual esthetics over ecological resilience	• apply “cues to care” strategies to improve esthetic acceptance • foster esthetic literacy and public engagement • integrate multifunctional and adaptive design approaches

**Economic barriers**		

Financial undervaluation^f^	• biodiversity undervalued • private funding limited • cost-effectiveness is hard to demonstrate • weak environmental, social, and governance (ESG) metrics	• use green/blue bonds, debt-for-nature swaps • strengthen ESG with AI and satellite data • build resilient financial systems
Asset recognition issues^g^	• existing accounting standards only consider built infrastructures as assets • local governments face financing limits • co-benefits of natural assets are undervalued	• adopt environmental-economic accounting and ISO 55000 standards • integrate GBI into policies • create biodiversity and water credits • reform asset accounting
Lack of comprehensive cost-benefit analysis^h^	• ecosystem services, biodiversity, and well-being are often excluded • high upfront costs • data gaps and inconsistency in valuation methods • weak ESG metrics and lack of standardized evaluation methods	• use real-world proxies and life cycle assessments • develop inclusive, standardized cost-benefit analysis • collect localized, context-specific, stakeholder-driven data in both costs and benefits

**Governance/policy barriers**		

Land use and space constraints^i^	• high land competition in dense cities • rising land values reduce feasibility • fragmented green networks • reliance on gray infrastructure systems	• promote adaptive, space-efficient GBI • prioritize micro-scale interventions (e.g., green roofs, permeable pavements) • strengthen land use regulations
Urban design barrier^j^	• conflicting demands among land uses • poor integration of ecological functions • weak coordination across spatial scales	• apply holistic and 3D spatial planning • design multifunctional and compatible GBI • use policy incentives to support implementation
Policy fragmentation and regulatory gaps^k^	• lack of clear, binding GBI legislation • institutional silos and poor coordination • inconsistent environmental standards	• implement legal and regulatory reforms • enhance interjurisdictional coordination • support capacity building and training
Integration challenges^l^	• GBI not embedded in walkability or transport systems • disjointed planning undermines synergies • poor alignment with social and ecological priorities	• foster multi-scale and cross-sector planning • strengthen participatory governance • deploy spatial analysis tools (e.g., GIS) for integration

Each barrier listed is further elaborated in the relevant sections of this review, where the source and context of the challenges are discussed in detail.

a See environmental injustice and GBI decision-making (Table S9).

b See cultural perspectives on GBI (Table S10).

c See social adoption hindering implementation of GBI.

d See safety and security barriers in GBI implementation (Table S11).

e See balancing climate adaptation and aesthetic goals in urban GBI (Table S12).

f See financial barriers to GBI implementation.

g See challenges in recognizing GBI as assets.

h See lack of comprehensive cost-benefit analysis.

i See land scarcity and urban sprawl.

j See urban design barriers.

k See lack of clear GBI implementation policies.

l See conflicts in promoting GBI and walkability.

### Environmental injustice and GBI decision-making

Cities frequently exhibit significant social inequalities in wealth and access to resources, including uneven distribution of green spaces and the ES they provide.^300^^,^^301^^,^^302^ Urban populations may be shaped by complex histories that include colonization, migration, and segregation along lines of race, ethnicity, religion, and caste, among others. These are further compounded by contemporary stressors that include increasing wealth inequality, the influx of refugees, and climate-related disasters. Consequently, the environmental trade-offs of green spaces (or lack thereof) in cities are not borne or enjoyed equally by everyone, resulting in systematic patterns of environmental injustice in most cities.^303^^,^^304^^,^^305^ Historically marginalized groups disproportionately experience greater exposure to environmental hazards, including industrial and vehicular emissions and water contamination, noise pollution, rising heat risks,^306^ floods,^307^ and green space shortages.^308^^,^^309^ Moreover, GBI projects in low-income communities often raise real estate prices, thereby pricing long-term residents out of their neighborhoods, a paradoxical phenomenon described as “green gentrification.”^310^^,^^311^^,^^312^^,^^313^ Consequently, the communities and urban areas with the greatest need for GBI investments are often the last to benefit. Equity in GBI implementation remains a key concern, as vulnerable communities often lack access to high-quality infrastructure.^314^ Addressing this imbalance requires inclusive strategies, community co-creation, and investment in underserved neighborhoods. The implementation of GBI rarely integrates these equity dimensions, often limited by data and governance constraints.^315^

Persistent environmental disparities are rooted in historic patterns of racial segregation in the US, including the practice of “redlining” created by the Home Owners Loan Corporation to demarcate parts of a city into zones based on perceived loan default risk, with Black and Brown communities relegated to the least desirable red zone.^316^^,^^317^ Redlining was officially enacted in hundreds of cities across the US from the 1930s to the 1960s, when the policy officially ended. Decades later, urban ecologists continue to find significant differences in tree canopy cover,^318^ impervious surface area,^319^ building density, air quality, summer temperatures,^320^^,^^321^ and biodiversity,^322^^,^^323^^,^^324^ with attendant disparities in public health outcomes.^325^

Cities in Europe and elsewhere have their own specific histories of unequal access for migrants and low-income communities, often with the similar result of low-income areas lacking adequate GBI,^326^^,^^327^ and therefore experiencing UHI,^328^ higher air pollution, lower biodiversity^329^^,^^330^^,^^331^^,^^332^^,^^333^ and poorer health outcomes over time.^334^

In the Global South, extreme urban inequality can be found in informal settlements shaped by self-construction and social struggle from marginalized groups, seeking the right to housing and the city.^335^ Over 1 billion people lived in urban informal settlements in the world in 2020, and, if urban poverty trends persist, these areas will continue to expand.^336^ In these excluded spaces, resource optimization is essential, yet they are rarely prioritized in urban green policies.^337^

On the one hand, green policies can generate tension by promoting gentrification, and on the other, the growing trend of increasing socioeconomic disadvantage in urban areas, especially in emerging economies, reproduces urban territories of informality where GBI is difficult to implement due to spatial constraints in dense cities and vulnerability, marginalization, and the lack of basic infrastructure. In the scientific literature, most articles focus on green gentrification and racial inequalities in the implementation of GBI, particularly in cities within the Global North. Table S9 presents a selection of relevant studies on this topic.

GBI projects have the potential to address these environmental legacies of historic segregation and current marginalization, but only if planned and implemented in ways that prioritize the needs and voices of these communities.^338^ At a minimum (Figure S9), participatory approaches and democratic governance are essential for ensuring that marginalized groups can participate in planning and governance to shape their neighborhoods.^339^ Such approaches should also recognize that barriers to social adaptation and structural access may differ across communities and require context-specific solutions.^340^ Some studies show that minority communities also report substantial benefit from local greenspace, and do not always feel marginalized, but only where they feel a sense of belonging.^341^ Top-down policy that aims to build GBI in under-resourced areas must include EJ criteria in project design as well as opportunities for meaningful participation in decision-making processes from the target communities. Finally, GBI initiatives should integrate EJ frameworks in both research and practice if we are to effectively disrupt the cycle of continued marginalization for these communities around the world.^342^^,^^343^ This approach also requires a multi-scalar perspective linking local decision-making to broader governance mechanisms at city, regional, and national levels.

Environmental injustice perpetuates unequal access to GBI, frequently excluding marginalized communities from its benefits and decision-making processes. Without equity-driven planning, GBI implementation exacerbates existing risks and disparities. Integrating justice frameworks and ensuring inclusive participation is crucial to guarantee that GBI simultaneously advances sustainability and social equity.

### Cultural perspectives on GBI

Although GBI is increasingly promoted, its cultural dimensions remain systematically under-addressed in both planning and implementation. A key issue is the persistent under-recognition of CESs, with limited attention to intangible values such as heritage, identity, and education.^118^^,^^344^^,^^345^ Community and cultural engagement is often weak, as top-down planning processes frequently overlook local socio-cultural perspectives, leading to limited public acceptance.^346^^,^^347^ Institutional procedures frequently privilege technical or esthetic criteria over place-specific cultural values, and indigenous knowledge systems, which offer valuable insights into sustainable land management, are also rarely incorporated into formal GBI strategies.^348^

In addition to these gaps, poorly contextualized interventions, such as inappropriate vertical greening, insensitive vegetation management, or the neglect of traditional spatial arrangements and layout principles can undermine both cultural heritage and microclimatic performance.^349^^,^^350^^,^^351^ Moreover, culturally specific landscape preferences are often ignored, resulting in GBI designs that fail to resonate with the diverse social, esthetic, and spiritual meanings attributed to green spaces.^352^^,^^353^ Together, these shortcomings reveal that neglecting cultural perspectives not only weakens public resonance but also undermines the long-term legitimacy of GBI. Table S10 summarizes case studies and reviews that reflect these challenges, revealing persistent gaps. Addressing these challenges requires a more inclusive, culturally grounded, and community-responsive approach to GBI.

Traditional and religious cultural contexts have demonstrated the capacity to enhance both ecological performance and cultural resonance. The Lingering garden in Suzhou, China, illustrates how culturally embedded landscapes can function as effective GBI.^354^ Through the integration of water elements, dense plantings, and shading structures, these classical gardens passively regulate microclimates by enhancing thermal comfort and humidity, particularly in shaded or water-adjacent areas.^349^ Similarly, the sacred Mughal garden of the Taj Mahal and the minimalist Zen garden of Ryoan-ji reflect how religious traditions have historically embedded GBI elements within spaces of ritual, reflection, and ecological value.^352^ These precedents demonstrate that microclimates and cultural narratives are co-produced through spatial codes and symbolic cues. However, such practices remain largely confined to small spatial scales. Scaling them up to the neighborhood or urban level is often hindered by mismatches with modern urban layouts, rigid planning systems, and the complexity of cultural meanings.^295^

Poorly contextualized GBI interventions can undermine the cultural heritage they aim to support. For example, installing vertical greening on historical facades may increase moisture retention and cause masonry cracking, accelerating material deterioration.^350^ In heritage sites, inadequate vegetation management may negatively affect microclimatic conditions, visitor experience, and the preservation of delicate historic structures.^351^ In Malaysian heritage cities, adaptive reuse of historic buildings has sometimes caused cultural and structural harm when added greenery ignores original layouts or materials.^355^ Collectively, these cases show that insufficient contextual sensitivity threatens both tangible heritage (materials, form) and intangible heritage (symbolism, identity).

Insufficient cultural sensitivity and weak integration of local values are key barriers to GBI success. When interventions neglect traditional knowledge, land practices, or community identity, they are often perceived as externally imposed, reducing public engagement and long-term stewardship. In Peru’s Tumbes Basin, for example, indigenous fog-harvesting terraces have been excluded from formal planning processes, marginalizing culturally significant land management practices.^348^ Christchurch’s Wigram basin demonstrated poor recognition of cultural benefits due to limited indigenous consultation.^356^ In Moscow’s Gorky Park, while recreational use thrives, educational and heritage functions remain underrepresented.^357^ These cases illustrate the consequences of undervalued CESs, sidelined traditional ecological knowledge, and heritage-insensitive programming.

To overcome cultural limitations in current GBI implementation, a 3-fold strategy can align design intent with place-specific cultural systems. First, integrating traditional ecological knowledge, such as local water systems, symbolic plantings, and spatial practices helps align GBI with place-specific values and deepens cultural relevance.^358^ Second, heritage-compatible assessment should be embedded from concept to maintenance using tailored frameworks, such as vegetation risk indices or heritage-compatible green design principles minimizing physical and symbolic damage.^359^ Third, public engagement can be strengthened through participatory processes that involve communities from early planning stages, particularly by embedding spaces for cultural rituals, storytelling, and place-based learning.^360^ These measures facilitate convert cultural sensitivity from aspiration into verifiable practices.

### Social adoption hindering implementation of GBI

Although urban GBI is widely promoted for its climate and ecological benefits,^361^^,^^362^ its implementation is often hindered by limited social acceptance and engagement. The durability of GBI depends on whether residents and stakeholders perceive interventions as safe, useful, fair, and worth caring for over time. Factors such as public perceptions, cultural relevance, safety concerns, and historical injustices influence how residents interact with these interventions.^363^^,^^364^ Social adoption depends on four interlinked dimensions: perceived benefits and risks (e.g., safety, usability), procedural fairness (inclusion and voice), distributional fairness (who gains/losses), and capacity for ongoing stewardship. Without adequate social alignment, even ecologically sound projects risk being underutilized or rejected.

Empirical research underscores the social value of GBI. Inclusive, accessible parks promote interaction, cohesion, and intercultural understanding.^365^ Green space design that encourages informal interactions such as bench conversations or children’s play, foster trust and belongingness.^366^ In Beijing, centralized, high-quality green spaces were more effective in enhancing residents’ community attachment than fragmented or inaccessible ones.^367^ These findings indicate that design choices facilitating everyday sociability, clear access, and inclusive use strengthen place-based attachment and social adoption.

Despite growing insights, social barriers to GBI adoption persist due to entrenched perceptions and institutional norms.^368^ While promoted for its multifunctionality, GBI often reflects planner-led priorities that are misaligned with local values and everyday practices.^363^ Planners may prioritize cost-effectiveness or technical feasibility, while residents resist interventions that disrupt cultural ties or land uses.^70^ This misalignment can weaken trust, leading to skepticism and reduced willingness to engage with or maintain interventions. Additionally, historical patterns of environmental injustice contribute to unequal access to urban nature, generating skepticism among marginalized groups regarding new GI interventions.^369^ These dynamics reflect both distributional inequities (who gets what where), recognition voids (respecting local’s values and norms) and procedural gaps (who decides how), which together weaken social license to operate. By contrast, where communities feel they belong in a place, the use of, and benefits from, local green spaces are plentiful.^341^

GBI interventions can trigger green gentrification (see environmental injustice and GBI decision-making), where environmental improvements displace vulnerable populations through increased property values.^370^ Governance structures lacking inclusive participation^339^ exacerbate these effects by limiting community’s inputs.^371^ This transforms GBI into a technocratic solution detached from community realities.^372^ Mitigating these risks requires anti-displacement measures (affordable housing commitments, community land trusts, benefit-sharing agreements, local opportunities) to prevent environmental gains from causing social losses. From a social adoption perspective, gentrification pressures erode trust and diminish stewardship participation.

A key barrier to GBI adoption is the misalignment between planners’ intentions and local communities’ values. Projects often prioritize ecological or esthetic goals but overlook concerns about safety, usability, or cultural relevance.^347^ In marginalized areas, residents may view dense vegetation or secluded spaces as unsafe (see safety and security barriers in GBI implementation).^373^^,^^374^ Perceived safety depends on lighting, sightlines, visibility of guardianship, and maintenance quality. Neglect in these areas quickly undermines adoption of GBI.

Limited awareness about GBI functions restricts informed engagement,^70^ while psychological barriers (low perceived self-efficacy, uncertainty about usage rules) suppress participation even when physical access exists. Without inclusive processes, communities remain disconnected from projects, weakening long-term stewardship.^70^^,^^361^ Additionally, green spaces shaped by dominant cultural norms may neglect the needs of women, elderlies, or ethnic minorities, making them feel unwelcome.^375^ These factors can cause the technocratic implementation to be disconnected from the social realities of GBI it seeks to serve.^372^

Enhancing social adoption of GBI requires embedding meaningful community engagement into planning and implementation.^339^ Participatory approaches such as co-design, mapping, and citizen science increase interventions’ legitimacy, relevance, and local ownership.^364^^,^^376^ Effective engagement is early (agenda-setting, not just consultation), iterative (multiple feedback loops), and accessible (multilingual materials, varied meeting times, paid participation where appropriate). Citizen science initiatives offer unique opportunities to capture place-based knowledge and skills, empower residents, and generate contextually rich data that inform GBI decision-making. Previous research on elderly’s perceptions suggest accessibility, safety, and usability, emphasizing the significance of green space availability and condition.^377^ Moreover, urban greenery’s contribution to supporting healthy aging reflections from elderly people highlights the significance of engaging them in recall assessments.^378^ These practices enhance procedural justice and build shared ownership beyond the project phase. Similar perception-base assessments are needed at local level with different backgrounds of the people in a society such as women, children, marginalized communities, etc., for the fairness of GBI projects.

Ensuring equitable access to GBI is critical for fostering public trust and addressing spatial and social inequalities. Prioritizing GBI in underserved areas can address injustices and build community trust.^379^^,^^380^ Linking these initiatives with local employment, education, or recreation generates co-benefits and strengthens public support.^381^ Communication campaigns, school programs, and targeted outreach raise awareness of GBI’s multifunctional benefits, from cooling to mental well-being.^382^

Institutional reform must support integrated, socially responsive planning through clear maintenance accountability, ring-fenced dedicated budgets for community partnerships, and metrics tracking inclusions (participation diversity, perceived safety, equitable usage, realized recognition). Cross-sectoral collaboration and adaptive governance can bridge ecological, technical, and social goals.^362^^,^^383^^,^^384^ Formalizing agreements with park friend groups and schools converts episodic engagement into stable care networks, embedding GBI within the urban fabric for its wider social adoption.

While the ecological and infrastructural benefits of GBI are well established, its successful implementation depends on overcoming social adoption barriers. Misaligned priorities, lack of engagement, inequitable access, and institutional fragmentation hinder public support. Addressing these challenges requires inclusive planning, equity-focused investments, effective communication, and integrated governance. A socially grounded approach to GBI is key to building resilient, just, and livable urban futures.

### Safety and security barriers in GBI implementation

Despite growing policy support, GBI implementation often faces barriers linked to safety and security concerns (Table S11). GBI can be perceived as both a resilience asset and a security concern.^385^^,^^386^ Poorly designed or maintained green areas may enable concealment or illegal activities where surveillance is limited.^387^^,^^388^ In Latin America and Sub-Saharan African regions, over half of urban planners have altered or canceled projects due to crime concerns.^389^ Similarly, a 2023 UK survey revealed 44% of urban residents avoid local green spaces due to safety concerns, which increased to 63% in disadvantaged areas.^18^ In Malmö, Sweden, residents’ concerns about poorly lit parks prompted redesign with improved lighting and surveillance,^390^ while London similarly redesigned small parks and paths to address drug use and antisocial behavior in unsupervised areas.^391^

Safety challenges occur across multiple spatial scales. On streets, dense vegetation can obstruct views and create blind spots, reducing passive surveillance.^386^^,^^387^ In housing areas, green fences may block visibility and undermine defensible space principles.^392^^,^^393^ In underserved neighborhoods, poorly monitored parks often attract antisocial behavior.^394^^,^^395^ Unmonitored greenways and gardens can invite antisocial behavior and vandalism despite fostering community engagement.^396^ In high-crime informal settlements, GBI faces resistance due to security concerns,^312^^,^^397^ while paradoxically areas under constant surveillance may restrict access for underprivileged groups (Table S11).^13^

Recent studies incorporate safety directly into GBI planning through multi-scale approaches that support climate adaptation while enhancing real and perceived safety.^4^^,^^5^^,^^398^ These frameworks emphasize integrated planning, inter-agency cooperation, and the evaluation of indicators for security and well-being across social groups.^397^ Multifunctionality indices and ES maps offer instruments to design socially safe.^255^^,^^399^

Crime prevention through environmental design (CPTED) complements these approaches by emphasizing natural surveillance, territorial reinforcement, and access control.^392^^,^^400^ Interventions such as motion-activated lighting and scheduled patrols improve perceived safety in isolated greenways. Technology enhances safety through smart lighting, CCTV, and IoT-based monitoring that preserve the landscape’s visual character.^401^^,^^402^

Community participation in planning, maintenance, and oversight fosters ownership and reduces vandalism through informal social control.^403^ Transforming neglected spaces into green spaces has been associated with reduced criminal activity and improved perceived safety.^404^ Effective GBI design should be informed by local risk assessments, integrated CPTED principles, and promote community involvement to develop inclusive resilient GBI.

Safety and security remain critical yet underexplored dimensions in GBI implementation (see Table S11). Effective GBI design should: (1) be informed by local risk assessments to reflect area-specific needs, (2) integrate CPTED principles and monitoring technologies to enhance both real and perceived safety while preserving ecological value, and (3) promote community participation to strengthen social oversight and reduce misuse. Addressing these factors is essential for developing inclusive, resilient, and socially sustainable GBI systems.

### Balancing climate adaptation and aesthetic goals in urban GBI

A tension between ecological functionality and prevailing esthetic preferences often challenges the implementation of GBI. While climate-resilient GBI prioritizes heterogeneous, function-driven, and ecologically complex design, urban planning and public perception have tended to favor tidy, ornamental landscapes.^405^^,^^406^ Historically, urban esthetics have emphasized beautification and recreation, prioritizing lawns and formal gardens.^406^^,^^407^ Increased awareness of climate change is driving a shift toward multifunctional landscapes guided by ecological principles and the provision of ES.^408^^,^^409^ For example, in 2019, King’s College Cambridge, UK, transformed its historic 1772 lawn into a wildflower meadow, reflecting a shift from formal turf to multifunctional landscapes.^410^ Nevertheless, modernist esthetic norms continue to shape public expectations and institutional practices, often hindering the adoption of climate-adaptive, structurally diverse, and ecologically robust designs.^405^^,^^406^ This misalignment between ecological needs and esthetic conventions limits GBI’s transformative potential.

Past scholarly works highlight the multifunctionality of GBI, focusing on its ecological, social, and esthetic co-benefits.^399^^,^^411^ However, recent evidence shows that esthetic priorities often precede ecological function in urban greening projects. For instance, street trees are often selected for their ornamental qualities rather than their ability to withstand drought or urban heat.^412^ Although native or climate-resilient species are ecologically valuable, they are perceived as unkempt or undesirable, provoking resistance from communities and policymakers.^413^ Esthetic preferences in urban landscapes are shaped by underlying socio-cultural norms and institutional frameworks.

Despite growing recognition of GBI’s multifunctionality, challenges remain in aligning ecological resilience with public expectations of beauty. A key conflict lies between ecological design principles, such as heterogeneity and native vegetation, and societal preferences for neatness and ornamental species.^414^^,^^415^ Functional GBI may appear disordered or untamed, often misinterpreted as neglect, especially in cultural contexts where tidy, manicured landscapes indicate care, safety, and social order.^405^^,^^406^

In addition, the lack of design elements that signal human intention and care can undermine public support for GBI projects (Table S12). This underlines the importance of integrating esthetic legibility into functional landscapes by carefully applying design principles grounded in social and cultural understanding.^407^

Institutional and governance frameworks often reinforce perceptual barriers; planning regulations, zoning codes, and funding mechanisms typically prioritize immediate visual appeal and public acceptance over long-term ecological performance and resilience.^415^^,^^416^ In such contexts, climate-adaptive infrastructure is frequently marginalized, particularly without interdisciplinary collaboration.^417^^,^^418^ Maintenance practices worsen this issue, as they are typically designed for conventional green spaces rather than complex, dynamic ecosystems requiring distinct expertise.^313^

Public interpretation of landscapes is strongly influenced by visible indicators of care, termed “cues to care.”^406^ These cues include trimmed edges, pathways, and signage that convey deliberate stewardship.^407^ Strategically applied, cues can bridge the gap between ecological functionality and public appeal. Integrating intentional design into the scientific design process, referred to as the “design-in-science” approach, is critical for translating ecological theory into practice.^419^ This framework underscores the importance of shaping spatial patterns by both ecological functions and their social meaning and visual clarity (Table S12).

Esthetics in climate-resilient urban design should apply integrative design principles from landscape ecology to guide planning, focusing on patch diversity, ecological corridors, and flows.^409^ Embracing “careful messiness” is essential; it reframes wildness as intentional, promoting esthetic literacy and ecological understanding among planners and the public.^406^ Urban planning norms must evolve by integrating adaptive principles into zoning regulations and development codes, thereby incentivizing multifunctional GBI.^409^ Public education fosters public acceptance; targeted awareness campaigns and participatory design processes can help reconcile community preference with ecological priorities.^368^^,^^406^^,^^417^ Finally, creating resilient and meaningful landscapes requires transdisciplinary collaboration, bringing together ecologists, landscape architects, urban planners, policymakers, and local communities.^409^^,^^418^

Many urban GBI projects still prioritize esthetics over climate resilience to contribute meaningfully to climate action. Public perception, policy, and limited funding often make it more challenging to adopt climate-resilient approaches. To truly future-proof cities and communities, GBI must integrate multifunctional, climate-adaptive designs that integrate both ecological and esthetic values. This will require a concerted effort that includes public education, participatory planning, policy reform, and the establishment of sustainable financing mechanisms to support resilient urban environments.

## Economic barriers

**Figure 4:**
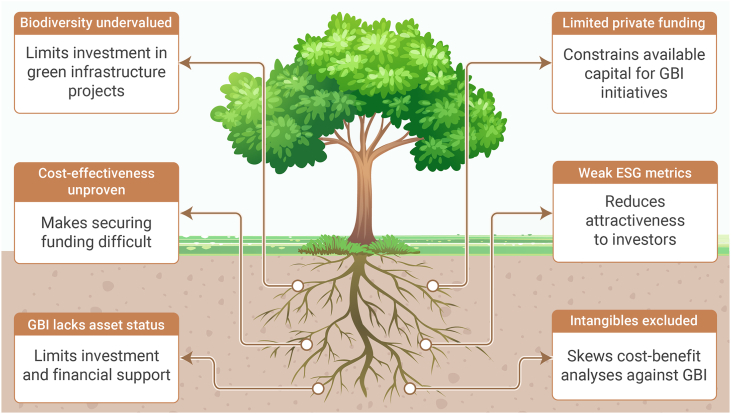
Schematic representation of the six financial challenges obstructing urban GBI implementation The illustration highlights how deeply embedded financial barriers can silently inhibit the growth and success of GBI initiatives. These barriers are further explored in the relevant sections of this review, where their origins, implications, and context-specific dynamics are discussed in detail.

This section identifies five financial and institutional challenges that limit GBI implementation: financial undervaluation of biodiversity, limited private investment, weak environment, social, and governance (ESG) metrics, and the lack of recognition of natural systems as formal assets. These issues reduce funding opportunities, lower investor confidence, and restrict policy support. Figure 4 outlines these key barriers and how they are interconnected. Each challenge is provided a detailed discussion in the following sections and summarized in Table 3, and a summary of discussed case studies is presented in Table S1.

### Financial barriers to GBI implementation

Over half of global GDP (or $44 trillion) depends on healthy biodiversity.^420^^,^^421^^,^^422^ Yet, the current economic system consistently undervalues biodiversity and natural capital.^423^ Traditional structures treat natural capital like other assets—financial, intellectual, and human—rather than recognizing the economy operates within, not alongside, nature.^423^ The financial system is described as the “economy’s circulatory system” and the large banking institutions as “the heart.”^424^^,^^425^ Today’s financial system differs greatly from earlier generations, mainly due to technological innovation.

Financial and policy decisions over the past century have driven biodiversity loss and now threaten financial stability. Despite its systematic risk, the biodiversity and finance agenda remain underdeveloped due to gaps in appropriate regulation, data, methodologies, and understanding. The finance ecosystem includes not only traditional institutions (banks, insurers, asset managers), central banks, multilateral development banks, accounting firms, and regulators, but also the growing FinTech sector. While regulators are beginning to address systemic risks, such as biodiversity loss, there is an urgency to understand how future biodiversity decline will impact businesses and how these risks may be priced.^420^

A GBI financial barrier is securing public and/or private financing.^51^ There is also a vast global disparity between public (86%) and private financing (14%).^421^ Private investors are crucial in tackling GBI-related sustainability challenges.^426^ Comparing cost-benefits with traditional solutions remains difficult, and the limited awareness of GBI financial benefits restricts funding. A clear understanding of valuation and risk mitigation is crucial. The biodiversity funding gap is estimated at $722–$967 billion.^427^ Compared with climate change finance, the biodiversity and finance agenda is underdeveloped, although key opportunities have been identified by UN and WWF TNFD and the WEF Future of Nature and Business Report.^423^^,^^428^

Technological innovation, especially AI, is transforming finance and has enormous potential to improve the objectivity of ESG, primarily for communication with regulators and investors.^429^ Alternative data, such as earth observation (EO) and satellite tracking, are accelerating AI adoption. Finance companies purchase vast quantities of financial and alternative data so that their funds can maximize performance. Managing these data is complex due to balancing manager performance, data proliferation, and sustainable financial modeling. There is also a lack of consensus on ESG measurement. Without better ESG metrics, biodiversity loss estimates, GBI, and related impacts risk being inaccurate, widening the gap between projected and actual financial losses. There needs to be more resilient and consistent ESG standards, especially with the new International Sustainability Standards Board. The application of satellite/EO data and state-of-the-art AI models (e.g., computer vision, generative AI, foundation models) is enhancing environmental monitoring. Improved monitoring enables stakeholders to access more precise ESG metrics, supporting resilient GBI valuation models and better risk management.

Debt capital markets are becoming an increasingly popular source to fund GBI solutions. The debt-for-nature swap reduces a country’s debt burden while allocating funds for conservation.^430^^,^^431^ In this mechanism, a country repurchases its debt at a discount and issues new debt on improved terms, using proceeds for environmental projects.^430^^,^^431^ Originating in 1987, the swap has gained traction in large-scale projects in Ecuador (2023), Gabon (2023), Barbados (2022), Belize (2021), and Seychelles (2015/2017).

Green bonds offer a potential solution to GBI issues, with proceeds tied to climate and environmental projects (e.g., green buildings, renewable energy).^432^^,^^433^ The European Investment Bank in 2007 issued the first green bond for financing renewable energy efficiency projects.^433^ Green bonds signal credible commitment through third-party certification and reputational risk for non-compliance.^433^ Investors typically respond positively to green bond announcements.^432^

Blue bonds, aimed at sustainable ocean economies, require careful analysis of ecological impact. However, before issuing blue bonds, issuers and investors need to make sure they can synthesize the environmental and social impact on the ocean and marine ecosystems. In 2021, Credit Suisse arranged $364 million in blue bonds in Belize, funding coastal protection and cutting debt by 12% of GDP.^434^

Financial solutions are pivotal in supporting GBI.^435^ Capital access and risk mitigation need to be prioritized. By 2030, a nature-based transition could produce US$10 trillion in business and generate 395 million jobs by 2030.^436^ Achieving this requires scaling investment to $536 billion annually by 2050.^421^ A strong financial ecosystem (Figure S10) is essential. Sustainability aligns finance with net zero, while addressing inequality and inclusion. *Explainability* ensures that systems behave as expected and builds transparent, trusted evidence for stakeholders. If the financial solutions are trustworthy, this will inspire confidence in individuals and organizations. Finally, resilient financial solutions must anticipate risks and support informed, adaptive decisions.

### Challenges in recognizing GBI as assets

Although the economic value of GBI can be quantified by comparing the benefits against costs,^437^ integrating GBI into policy priorities remains challenging. A key obstacle is that policymakers, planners, and local governments’ asset managers often treat GBI as a liability rather than a formal asset. For example, while one study estimated a major London park’s true value at £108 million, many councils still record parks at a nominal £1.^438^

Current accounting standards in the US and Australia fail to recognize the natural components of GBI (e.g., trees, soil, vegetation, and water) as formal assets. Under US financial rules, only human-engineered GBI (e.g., constructed stormwater systems) qualifies as an asset, while living systems are effectively assigned zero value despite their proven environmental, social, and health benefits.^439^ This artificial divide overlooks the hybrid nature of GBI: for example, parks combine built infrastructures (playgrounds, pipes) with ecological features (wetlands, trees),^67^ yet accounting practices capture only the former. Further, actuarial practices recognize risks such as tree-related property damage, while ignoring benefits such as flood mitigation.^440^

The Australian Accounting Standards Board^441^ classifies plants as assets only if they are agricultural or bearing crops, valuing them solely by acquisition cost. Water and non-commercial vegetation—even when critical for carbon sequestration—are excluded. Remote sensing and modeling advancements have improved carbon stock quantification, yet frameworks such as the National Carbon Accounting System focus narrowly on emissions rather than asset valuation.^442^

Globally, GBI’s asset recognition remains inconsistent. While the UK integrates GBI into planning policies, challenges persist, such as undervaluing GBI components (e.g., sustainable drainage systems) and gaps in performance metrics.^66^^,^^443^ Meanwhile, private developers exploit the market appeal of green proximity without contributing to GBI upkeep, highlighting a disconnect between perceived value and institutional accounting.^444^

A core barrier to GBI investment lies in its exclusion from institutional asset frameworks. Local governments, which hold the property rights and are chiefly responsible for managing urban GBIs, face significant budgetary and regulatory constraints because GBI rarely generates direct revenue under current accounting standards. The lack of formal recognition of GBI as assets in local accounting rules means that access to innovative financing (e.g., loans backed by tangible revenue streams) is limited.

The demonstrable benefits of GBI demand institutional realignment and formal recognition. Over a decade ago, Barbier^445^ advocated for ecosystems such as wetlands to be valued as natural assets, given their provision of goods, services, and cultural benefits. Echoing this, Vardon et al.^446^ called for governments to prioritize natural capital by integrating environmental accounting into core decision-making, bridging economic and ecological data.

Notable advancements are being made. Roghani et al.^447^ argued that GBI performance must be evaluated holistically, balancing costs against all primary and secondary benefits, aligning with ISO 55000’s asset management principles.^448^ Practical initiatives, such as EU’s Natural Capital Accounting initiative, the IPWEA’s guidance for Australian local governments,^449^ and the System of Environmental-Economic Accounting (SEEA), in ecosystem accounting^450^ are advancing standardized assessment methods for GBI.

Market-based mechanisms offer further promise. Carbon markets exemplify how public-private investment can monetize environmental assets, creating revenue streams for loans and reinvestment.^451^ Emerging markets for biodiversity or water quality credits—although currently underdeveloped—could follow a similar path. Initiatives such as Australia’s Nature Repair Market signal progress,^452^ by credibly monetizing co-benefits through market mechanisms, GBI can shift from being undervalued ecological infrastructure to recognized financial assets. This shift could unlock mainstream investment, empower local governments to act as accredited sellers, and establish sustainable revenue streams that justify GBI’s formal inclusion in institutional accounting frameworks, enabling access to innovative financing for GBI managers.

Despite its proven environmental, social, and economic benefits, GBI remains systematically undervalued in policy and practice. Institutional frameworks in many countries—constrained by rigid accounting standards—continue to classify GBI as a liability rather than an asset, with only its human-engineered components meeting traditional asset criteria. To address this imbalance, GBI must be redefined as natural capital assets, supported by valuation frameworks that holistically account for both costs and multifunctional benefits. Emerging approaches, such as ISO 55000-aligned asset management, Environmental-Economic Accounting, nature-positive markets, and hybrid green-gray designs, demonstrate viable pathways to institutionalize GBI’s value. Critical steps include revising accounting standards to recognize ecological assets and the benefits they provide, developing tools to quantify GBI performance across environmental, economic, and social dimensions, and leveraging carbon and biodiversity credits to monetize ES. The urgency of climate adaptation and biodiversity loss demands that cities treat GBI as foundational infrastructure, not an optional amenity.

### Lack of comprehensive CBA

CBA is commonly employed to assess the economic viability of any GBI intervention before making investment and implementation decisions.^453^ It translates ecological values into financial metrics and may shape GBI innovation.^454^^,^^455^ Lacking comprehensive CBA understates GBI sustainability and effectiveness.^453^ Traditional CBAs overlook indirect benefits and externalities in GBI,^453^^,^^456^^,^^457^ leading to underinvestment.^456^^,^^458^

Methodological gaps present significant challenges in GBI evaluation. The difficulty in monetizing intangible benefits (ecological and social values) hinders the full cost-benefit analysis.^459^^,^^460^ Specifically, comprehensive CBA of cultural heritage, biodiversity, equity, and well-being remains challenging in terms of putting monetary values of such services,^459^^,^^460^^,^^461^^,^^462^ while benefits such as stormwater control and energy savings from green roofs are more straightforward to quantify.^460^ GBI benefits with direct or indirect impacts on human health would class as intermediate complexity to value in monetary terms, but are increasingly included for regulating ES functions that GBI provide, such as air pollution removal,^463^^,^^464^ cooling,^376^^,^^465^ and noise mitigation.^150^ The absence of adequate valuation techniques and inconsistent methodologies^457^ further complicates full-scale assessments of GBI’s ecosystem functions.

Economic barriers also impede GBI implementation. High upfront costs and uncertain returns discourage stakeholder investment.^466^ Studies show that green roofs, water squares, and other GBI often fail to achieve favorable benefit-cost ratios within typical 30-year project spans, losing investor and policymaker interest.^457^^,^^467^ Green roofs, in particular, are economically viable only with subsidies due to high construction and maintenance costs, often yielding zero or negative net present value.^468^ Even if savings from avoided may not offset the high initial costs when other co-benefits are excluded.^469^

**Figure 5:**
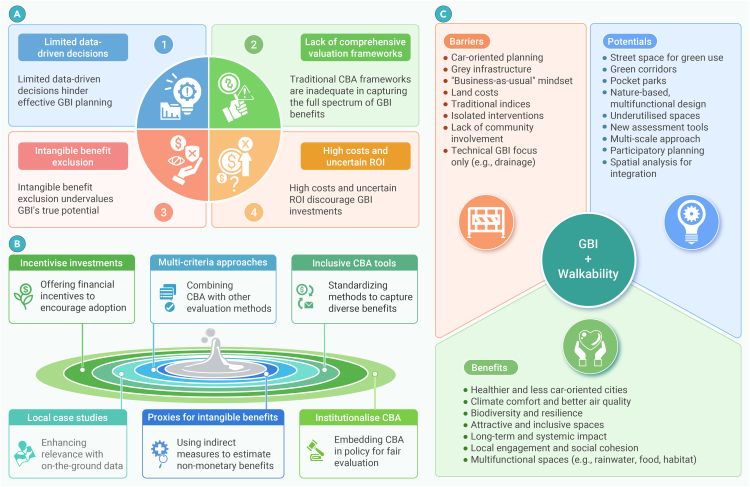
Schematic representation of key CBA and walkability challenges and corresponding strategies (A) Each numbered icon in this figure illustrates the core challenges in CBA that hinder the GBI implementation. (B) Targeted strategies, each linked to one or more of the challenges listed in (A), that help overcome CBA challenges in GBI implementation and drive more informed, fair investment decisions (see lack of comprehensive cost-benefit analysis. (C) Barriers, potentials, and benefits of integrating GBI and walkability strategies in urban design (see conflicts in promoting GBI and walkability).

Contextual variability poses additional analytical challenges in CBA of GBI. Data scarcity and variations by geography, neighborhood, or GBI type complicate analysis due to benefits' dynamic spatial and temporal variability (Figure 5A).^457^^,^^470^^,^^471^ CBAs are sensitive to uncertainties, model assumptions, and data availability,^470^ especially for impacts such as mental well-being or noise reduction.^460^^,^^472^ Context-specific performance variability^473^ and poor empirical evidence^454^ further limit comprehensive evaluation of GBI’s values and services.

These methodological and contextual gaps demonstrate the need to institutionalize CBA in GBI planning. Undervalued intangible benefits suggest less recognition of ES and social cohesion as GBI.^461^^,^^474^ The insufficient consideration of improved air and water quality, biodiversity, ecosystem resilience, noise reduction, and increased property values^454^ results in underestimation of GBI’s total value, deterring further investments.^475^ Table 4 summarizes case studies highlighting these challenges, revealing persistent gaps in CBA that hinder GBI implementation, along with key recommendations to overcome these challenges.

**Table 4:** Summary of the most relevant studies highlighting CBA hindering GBI implementation along with the key recommendations to overcome these challenges

CBA challenges	Case study/review	Key findings	Key recommendations	Reference
Challenges in monetizing intangible benefits	Ayutthaya Island, Thailand	valuing ES is difficult, limiting recognition of mitigation benefits	develop indicators reflecting various ecosystem service to enhance valuation	Vojinovic et al.^461^
	43 US, D.C., and Canada	challenges in quantifying intangible benefits due to GI’s complex structure	collect detailed data to better understand and value intangible benefits	Kim and Song^462^
	Systematic review (79 articles)	difficulty in monetizing benefits such as well-being and noise reduction; often excluded from economic evaluations	develop consistent methodologies to account for intangible benefits	Teotónio et al.^460^
	literature review (129 studies)	challenges in monetizing intangibles such as quality of life, well-being, and biodiversity	utilize multi-criteria analysis to quantify these benefits for integration into CBAs	Manso et al.^459^
Exclusion of co-benefits and externalities	systematic review (114 observations)	many CBAs overlook indirect benefits and environmental externalities underrepresenting GBI	combine CBA with complementary methods such as multi-criteria analysis, cost-effectiveness analysis, and qualitative assessments to better capture co-benefits	Chelli et al.^453^
	Sheffield, England, and other European cities	traditional valuations often miss co-benefits such as health, equity, and ecosystem resilience	adopt holistic frameworks and increase public sector engagement to support investments that reflect all benefits	Wild et al.^458^
	case study: Modesto, CA	existing CBAs may exclude urban forestry co-benefits such as species variability and ecological functions	promote research on species-level ecological performance to better incorporate of benefits into economic models	McPherson^476^
Lack of comprehensive data and standardized methods	Bruges, Belgium	difficulty in estimating exact values due to combined use, non-use, and investment values	develop a clear handbook to guide users in generating and understanding benefit transfer values	Vandermeulen et al.^477^
	Sint Maarten Island, Caribbean	challenges in monetizing co-benefits and uncertainties due to data availability and local issues	include a broader range of co-benefits to improve data collection and integration	Alves et al.^456^
	Barcelona and Badalona, Spain	lack of reliable data and standardized methods for hydrological performance of GI	use approaches such as Monte Carlo simulations to address uncertainties	Locatelli et al.^470^
	rapid evidence assessment (1,700 documents)	lack of reliable data and standardized methods for assessing NbS impacts	develop new methods beyond traditional CBAs to improve data collection and integration	Raymond et al.^472^
	book review (Europe, North America, China)	lack of comprehensive data and standardized methods for evaluating NbS effectiveness	systematically analyze data, including CBAs, and identify causal mechanisms to improve data collection and standardization	Kabisch et al.^478^
	London, UK	lack of comprehensive data and standardized methods for evaluating physical and social outputs of community gardens	use simplified CBA methodologies to improve accessibility and standardization	Schoen et al.^455^
CBA uncertainty	Oslo, Norway	CBAs are sensitive to data availability, uncertainties, and model assumptions	validate results using real-world data sources, such as insurance data, to improve accuracy	Wilbers et al.^457^
	Jung-gu, Seoul, South Korea	uncertain assumptions and regional factors reduce reliability of CBA	undertake additional region-specific studies that consider environmental, socioeconomic, and physical factors to improve decision-making	Shin and Kim^454^
	Madrid, Spain	critique of CBAs due to uncertain assumptions, discounting and lack of communicating output uncertainties	consider both monetary and biophysical values in decision-making to reduce uncertainty	Babi Almenar et al.^479^
	Newcastle, UK	difficulty in monetarily valuing multiple benefits of GBI	use tools such as CIRIA BeST to structure assessments for quantifying and monetizing each benefit	O'Donnell et al.^480^
High costs and uncertain return on investment (ROI)	Tanyard Branch Watershed, Athens, GA	green roofs have a higher net present value (NPV) compared with conventional roofing, indicating higher costs and uncertain ROI	gain more experience and establish long-term warranties to justify investments	Carter and Keeler^467^
	Grand Rapids, MI	high costs and uncertain ROI for green roofs; negative NPVs unless part of LEED-certified buildings	incorporate green roofs into LEED-certified buildings to leverage additional benefits such as rent premiums	Nordman et al.^468^
	Genoa, Italy	high installation and maintenance costs lead to uncertain ROI, especially for intensive green roofs	recommend tax incentives to improve ROI	Perini and Rosasco^475^
	Southern France and Rotterdam, the Netherlands	benefits from avoided damages are often insufficient to cover costs, leading to high costs and uncertain ROI	adapt public funding rules to assess for cross-sectoral assistances of NbS	Le Coent et al.^469^
	systematic review (116 articles)	elevated costs and uncertain ROI stem from insufficient knowledge about expenses, benefits, and impacts	develop guided examples of cost calculation, depreciation, and discounting to aid in creating credible business cases	Van Oijstaeijen et al.^226^
	Kuala Lumpur and Johor Bahru, Malaysia	high costs and uncertain ROI of green roofs, particularly for local authorities make the economic worth unclear	integrate intensive green roofs on flat rooftops to reduce costs and improve ROI	Shazmin Shareena and Nur Amira^481^

Integrated strategies can address challenges and promote wider GBI investment (Figure 5B). Real-world case studies as proxies, such as value changes and insurance data, help quantify the benefits of urban greening and flood mitigation.^482^^,^^483^ These approaches reduce uncertainty and help build more inclusive valuation frameworks. Studies suggest the need for long-term, context-specific data (e.g., real estate, insurance, and hospital records) to assess flood and health benefits.^484^ Incorporating local case studies and stakeholder input improves data relevance and reflects real-world conditions.^455^^,^^485^ Financial concerns, particularly high upfront costs and uncertain return on investment, can be mitigated by integrating co-benefits comprehensively through life cycle assessments and offering targeted incentives, such as tax relief or subsidies.^226^ Similarly, institutionalizing CBA by integrating the CBA framework into the planning processes could result in a fair and comprehensive manner of evaluating NbSs.^457^ Improved modeling approaches, which are better able to capture context-specific GBI performance, coupled with health assessments and economic valuation of the associated benefits, could address some of the valuation challenges,^464^ but are still underutilized and not fully standardized. For instance, a comprehensive CBA of street trees in Adelaide, Australia, demonstrated a 1.6 benefit-cost ratio across various neighborhoods, providing valuable evidence to support urban greening policies.^486^

While CBA holds potential to guide innovation and investment in GBI, its current application remains limited and incomplete. To account for the whole economic profitability of GBI, a standard CBA method must be developed incorporating more inclusive, transparent, and context-aware tools that can capture the full spectrum of ecological, social, and economic values, ensuring that NbSs are not just observed but valued and implemented for their full economic potential.

## Governance/policy barriers

**Figure 6:**
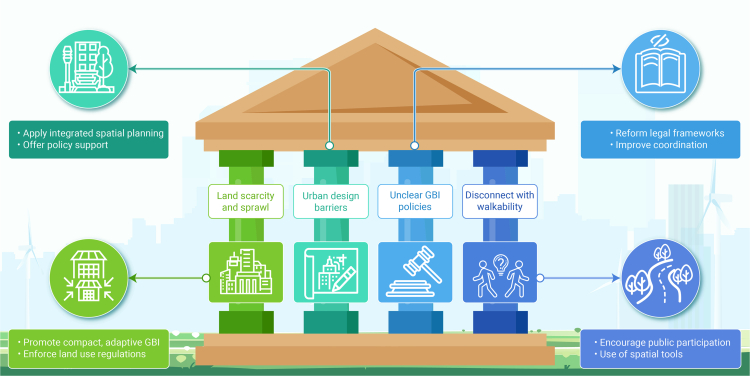
Governance and policy barriers to GBI implementation and corresponding solutions Each pillar highlights a critical challenge—land scarcity and urban sprawl, urban design limitations, unclear policies, and disconnect with walkability—alongside strategic interventions required to overcome each barrier.

This section identifies four barriers to GBI implementation: land scarcity and urban sprawl, urban design limitations, unclear GBI policies, and the disconnect with walkability. Governance frameworks are essential for scaling and sustaining GBI, yet progress is hindered by fragmented legislation, weak political support, and outdated planning practices. Figure 6 presents the barriers in a layered format, linking each to broader governance shortcomings and corresponding strategies—ranging from spatial planning improvements to enhanced coordination. Challenges and corresponding solutions are explored in the subsections and summarized in Table 3, and a summary of discussed case studies is presented in Table S1.

### Land scarcity and urban sprawl

Land scarcity and urban sprawl present interconnected challenges for implementing GBI in cities. Intense competition among residential, commercial, and infrastructural uses in dense urban areas makes space for GBI financially and spatially limited.^487^^,^^488^ This constraint is especially acute in rapidly growing metropolises such as São Paulo, where new GBI creation can be considerably limited by land use disputes.^489^ Rising land values further restrict the feasibility of large-scale GBI projects, as development priorities favor profit-driven densification over public benefit.^490^^,^^491^ Simultaneously, urban sprawl, characterized by low-density expansion consumes peri-urban lands that could otherwise host greenbelts or ecological corridors. It fragments natural ecosystems, reduces biodiversity, and increases car dependency and emissions.^487^^,^^492^^,^^493^ Bucharest exemplifies how unchecked sprawl directly converts natural land and disrupts ecological continuity, while also inflating the cost of sustainable infrastructure, leading to reliance on traditional gray systems instead of integrated approaches such as water-sensitive urban design.^487^^,^^494^^,^^495^

Both challenges contribute to fragmented, underperforming green networks. In dense cities, limited space constrains the dimensions and connectivity of parks and ecological corridors, while making retrofitting technically and financially challenging.^496^^,^^497^ In sprawled areas, although land may be more abundant, GBI is often poorly maintained, spatially isolated, or inaccessible, diminishing the ability of GBI to deliver critical ES, such as flood and noise mitigation and recreational opportunities.

To effectively address these constraints, cities must adopt space-efficient solutions and strategic approaches. Micro-scale interventions such as green walls and roofs can be incorporated into existing buildings^4^^,^^5^ without requiring additional land,^498^^,^^499^^,^^500^^,^^501^^,^^502^ while supporting multiple functions including heat mitigation, biodiversity, reduction of energy use, and air and noise pollution.^503^^,^^504^^,^^505^ Similarly, permeable surfaces help water infiltration, reducing stormwater runoff, and recharging groundwater.^506^ Street trees and pocket parks maximize urban space, providing shade, cooling, and green areas that prevent the UHI effect.^507^^,^^508^^,^^509^ By strategically integrating these GBI elements into the existing urban fabric, cities can achieve sustainability benefits without contributing to further sprawl,^510^^,^^511^^,^^512^ although regular maintenance is essential for maintaining effectiveness.

### Urban design barriers

GBI faces distinct challenges across technical, economic, social, and policy dimensions. These include: (1) technical integration challenges: balancing architectural demands and costs with ecological needs, particularly in innovative solutions such as rooftop greening; (2) functional conflicts: competition between ecological benefits and practical functionality in limited urban space; (3) multi-scale coordination challenges: fragmentation of regional ecological networks by property divisions; (4) design maintenance disconnect: insufficient consideration of maintenance requirements, leading to functional degradation and becoming “one-time projects”; (5) policy and technological inertia: traditional frameworks and established technical configurations that resist innovation and cross-sectoral collaboration (Table 5).

**Table 5:** Key urban design challenges hindering effective GBI implementation and potential technical and strategic solutions

Challenges	Solutions	References
Spatial integration challenges	• vertical greening systems • optimize structural load distribution • incorporating hydroponic or modular planters • rooftop agriculture with lightweight substrates and automated irrigation systems	Xi et al.^513^; Su et al.^514^; Blair and Johnson^515^
Functional conflicts	• bio-retention areas integrated into parking lots • permeable pavements paired with underground retention systems • bio-retention facilities replace conventional drainage channels	Cortinovis et al.^516^; Ronchi et al.^261^; Pogliani et al.^517^
Multi-scale coordination challenges	• map ecological corridors • align regional networks with site-specific development • ensuring GBI integration across urban scales	Pauleit et al.^518^; Xi et al.^519^; Xi et al.^520^; Xi et al.^521^; Kanniah^522^
Maintenance deficiencies	• drought-resistant plant communities and modular • easily replaceable components	Tabatabaee et al.^523^; Rosa et al.^524^; Barbosa et al.^525^
Policy and technological lag	• GBI design by embedding resilience thinking • tax abatements and regulatory updates • interdisciplinary collaboration	D'Amato and Korhonen^526^; Fuenfschilling et al.^527^; Davies and Lafortezza^362^; Tapia and Reith^528^; Tapia et al.^529^; Ahmed and de Oliveira^530^

While expanding GBI areas can help reduce temperatures,^513^ high building coverage in high-density urban areas often limits its implementation.^514^ Additionally, urban expansion’s encroachment on natural GBI poses a severe problem.^515^ Against this backdrop, there is a need to combine multifunctionality and regional coordination for GBI deployment,^516^ requiring a paradigm shift from traditional design approaches to an ecological framework. Traditional planning models are strictly based on land use classifications (residential, industrial, or commercial) and rarely consider whether land is suitable for specific functions or its consistency within a broader territorial context.^261^^,^^517^

Given GBI’s critical role in ecological service systems, connectivity and multifunctionality have become core design principles.^518^ However, current GBI development and design research remains fragmented, focusing on micro-scale elements such as street trees,^519^^,^^520^ community green spaces,^521^ or city-level tree canopy coverage,^522^ with limited consideration of multi-scale connectivity. Furthermore, while many studies aim to enhance GBI’s potential through life cycle risk assessment models^523^ and CBA,^524^^,^^525^ life cycle aspects such as maintenance are often overlooked during actual design and implementation.

Current policies and technical preferences prioritize conventional engineered solutions,^526^ while established technical configurations have become institutionalized and rigid over time,^527^ further influencing decision-making by urban designers and policymakers to favor entrenched methods.^362^ Insufficient cross-sectoral coordination and inadequate public participation mechanisms further constrain GBI implementation. However, studies show that integrated planning approaches combining technical solutions with community co-governance are gradually improving feasibility.^528^^,^^529^ While many cities recognize GBI’s importance in local regulations, developers are often unaware of these guidelines, particularly in low- and middle-income countries (LMICs).^530^ For example, Ekostaden Augustenborg in Malmö, Sweden, transformed a declining 1950s housing district through integrated redevelopment that combined nature-based stormwater systems and green roofs with community co-design and governance.^531^

Integrating GBI into urban design requires a holistic approach that reimagines space, technology, and governance (Table 5). Spatial constraints can be addressed through three-dimensional design,^532^^,^^533^ such as optimizing structural load distribution while incorporating hydroponic or modular planters to reduce heat islands and improve air quality.^534^^,^^535^ Rooftop agriculture can be designed using lightweight substrates and automated irrigation systems to maximize productivity without compromizing building integrity.^536^ Functional compatibility demands synergistic design, where permeable pavements are paired with underground retention systems to manage peak flows and prevent flooding, while bio-retention facilities replace conventional drainage channels to enhance biodiversity and water quality.^537^ Multi-scale coordination relies on GIS-driven planning tools to map ecological corridors and align regional networks with site-specific development, ensuring seamless integration across urban scales.^538^ Maintenance challenges are mitigated through low-maintenance technologies, such as drought-resistant plant communities and modular, easily replaceable components, while community-based stewardship programs foster accountability and long-term sustainability. Policy frameworks must evolve to incentivize GBI through tax abatements and regulatory updates, while interdisciplinary collaboration ensures that ecological, social, and economic objectives are balanced.^8^ By embedding resilience thinking into design, GBI transitions from an ancillary feature to a foundational element of urban systems, creating adaptive, equitable, and ecologically functional cities.

In order to overcome the challenges of GBI in urban design, it is necessary to take multidimensional measures.^247^ The use of data-driven design and cross-disciplinary technological integration is crucial,^529^ while also focusing on policy innovation and institutional safeguards. Establishing specialized agencies and cross-departmental cooperation mechanisms can help strengthen supervision and reform. In terms of talent cultivation, both cross-disciplinary talent training and vocational staff training can enhance the professional quality and the collaborative abilities of relevant personnel. Comprehensive participation mechanisms and capacity building and education programs can be used to engage community residents.^8^ By implementing these structural and institutional approaches in conjunction with practical technical measures, GBI can be transformed into a core framework for urban resilient development, ultimately promoting sustainable urban transformation.

### Lack of clear GBI implementation policies

The lack of clear policies for GBI implementation is a widely discussed barrier in the literature and generally exhibits a high degree of transversality, inherent to the nature of policymaking itself. This barrier does not always appear in the scientific literature as a topic with clearly defined boundaries. Rather, it can be addressed as an institutional and governance barrier,^70^ encompassing critical themes such as leadership, strategic vision, political commitment, inter-agency cooperation, legislative frameworks, environmental policies, and conflicting policy actions. Alternatively, it may be framed within a broader socio-political understanding of barriers, capturing intrinsic societal elements that ultimately shape the reluctant behavioral patterns of policymakers in the gray-green transition.^480^ Within this socio-political perspective, the lack of clear GBI policies appears even more transversal, expanding to include additional factors, such as negative experiences, responsibility, capacity, knowledge, organizational culture, financial constraints, administrative bureaucracy, lack of clear standards and guidelines, and resistance to change.^539^^,^^540^^,^^541^^,^^542^ Considering the broad scope of this barrier, its challenges will be grouped into five key topics: leadership, tradition, legislation, knowledge, and priority conflicts.

The lack of leadership, clear vision, and political will constitutes a major barrier to the implementation of GBI, particularly at lower levels of public administration where GBI is often absent from urban planning agendas.^143^^,^^543^^,^^544^ Weak leadership is further reflected in institutional fragmentation, ineffective communication, lack of commitment, ambiguous role definitions, and the isolation of managers across different sectors.^372^^,^^544^^,^^545^ Without a long-term vision, managers are unlikely to support initiatives whose results extend beyond their administrative term.

Economic interests historically associated with gray infrastructure valued for its visual prominence, perceived contribution to economic growth, and potential for job creation continue to pose a significant political barrier to the adoption of GBI.^480^ Since GBI projects mature slowly and lack immediate electoral visibility, they are less politically attractive.^480^^,^^546^

The absence of specific legislation and interjurisdictional authority limits GBI implementation.^70^^,^^480^^,^^540^^,^^547^ Environmental challenges that cross administrative boundaries demand federal regulation. Current legal frameworks often mandate conventional materials, reinforcing gray infrastructure solutions.^70^^,^^548^ The limited integration of scientific knowledge into policymaking often results in unrealistic expectations for GBI projects. Climate change and urban environmental shifts are often inadequately considered, reducing GBI efficiency and adoption continuity.^480^^,^^545^ Gaps in understanding the societal benefits of GBI and inadequate training, particularly in engineering fields, exacerbate the problem.

Limited financial resources, especially in LMICs, hinder GBI policy development, as these initiatives often struggle to compete with gray infrastructure in CBAs.^70^^,^^372^^,^^480^ Without public recognition of GBI’s value, political pressure is minimal, perpetuating a cycle of invisibility and exclusion from the political agenda.

Overcoming the challenges to GBI implementation policies requires addressing two fundamental and interdependent aspects. First, the transition from gray-to-green infrastructure will be slow and gradual, demanding continuous relearning by academics, politicians, and managers. As this transformation unfolds over a longer timescale than electoral cycles, strong and comprehensive environmental policies across all levels of public administration are essential.^549^ Over time, as the benefits of GBI become better valued, political and economic interests traditionally tied to gray infrastructure are expected to shift toward greener solutions.^550^ Melbourne’s long-running gray-to-green initiative is a striking example of a slow, strategic shift from gray to GI, supported by enduring policy frameworks across local government.^551^

Second, human resource training and knowledge production are critical.^552^^,^^553^ GBI solutions are relatively new compared with conventional structures and remain unfamiliar to many managers and technicians. Professionals trained under traditional urban engineering paradigms often resist innovative, systemic approaches and lack technical training, didactic guides, regulatory support, and confidence in GBI models. To enable the gray-to-green transition, curricular reforms are necessary to equip future professionals with the skills needed to implement GBI solutions.^554^^,^^555^

The effective implementation of GBI is hampered by the lack of clear, cohesive policies, rooted in both governance challenges and broader socio-political dynamics. Key obstacles include inadequate leadership, vision, and political will; economic interests favoring gray infrastructure; outdated legislation and fragmented regulatory authority; and poor integration of scientific knowledge into policymaking. Financial constraints and competing priorities, especially in LMICs, further marginalized GBI, creating a cycle of invisibility due to limited public demand. Overcoming these barriers requires strengthening leadership, reforming legislation, promoting knowledge dissemination, and increasing public awareness to establish GBI as a political and societal priority.

### Conflicts in promoting GBI and walkability

Urban GBI is a multipurpose strategy to address urgent societal problems by offering ESs that improve public health and quality of life and thus can increase walkability. However, despite these benefits, recent research has shown that GBI and walkability have been poorly integrated, revealing a paradox that demands deeper reflection on the barriers hindering their synergy.^556^ A multi-scale approach is essential, combining urban design with social and environmental needs. Car-centric policies, gray solutions, prioritization of conventional infrastructure investments, and “business as usual” planning need to be revisited to avoid obstructing integration. In addition, GBI projects must be developed from a multipurpose perspective, including enhancing pedestrian attraction.

Walkability contributes significantly to urban quality and public health,^557^^,^^558^ being defined as the effectiveness in promoting and facilitating walkways by ensuring comfort and safety, linking people to diverse destinations, and creating visually engaging routes.^559^^,^^560^ This relationship is exemplified by a study from Michigan, USA, where walkability is associated with reduced obesity rates and improved mental health outcomes.^561^

Features that support well-being relate to the provision of walkable, community, and vegetated areas, emphasizing the importance of integrating urban planning, biodiversity, and ES.^562^ Thus, the production of CESs arises from the interrelations between people and the landscape, with urban green areas understood as cohesive systems that offer comprehensive human experiences.^67^^,^^563^

Promoting walkability decreases sedentary behavior and encourages physical activity, thereby reducing incidence of various health conditions such as obesity, diabetes, and anxiety.^564^^,^^565^ The pedestrian pathways quality depends on multiple aspects of the built environment, including sidewalk width, presence of trees, safety, mixed land use, active frontages, lighting, etc.^560^^,^^566^ Walkable areas tend to be less car dependent, which leads to improved air quality, reduced noise, decreased urban heat, and an increased offer of many other ES. The implementation of GBI contributes to increasing urban greenery and can significantly improve walkability. In addition, these strategies support resilience.^567^^,^^568^^,^^569^

However, despite multiple benefits, recent research indicates a poor correlation between GBI and walkability.^556^^,^^570^^,^^571^ Factors include car-oriented planning, the prioritization of gray infrastructure, and land costs in denser areas. Engineering projects for drainage, heat islands, brownfield revitalization, and green areas that prioritize conventional solutions are examples of barriers. Also, many walkability assessment indices do not properly incorporate solutions focused on GBI, overlooking broader benefits of a walkable environment.^556^ Finally, a lack of public participation and local contextualization limit success.^568^^,^^572^

In terms of streetscape design, a diverse array of GBI strategies can be adopted, ranging from creating GCs that provide shade and thermal comfort to technical solutions that absorb stormwater. For a more effective implementation, it is essential to consider multiple scales of walkability. Alignment between GBI and site potential at the macro-scale is necessary prior to micro-scale interventions, ensuring that impacts extend site-specific actions. In denser areas, promoting linear and pocket parks, or reappropriating smaller spaces such as parking spots, can create attractive areas for pedestrians, recognizing that their long-term benefits outweigh the initial costs.^573^

Spatial analysis tools, including GIS, can be used to identify vegetated spaces and evaluate their potential to provide ES.^574^ These areas can be transformed into pedestrian networks integrated with GBI,^568^^,^^575^ offering benefits such as improved air quality.^5^^,^^37^ Moreover, new methodologies for public policymaking and engaging communities are necessary, particularly those addressing climate change and biodiversity in walkability initiatives.^572^ In this context, GBI emerged as a strong ally, offering potential to mitigate and adapt the impacts of extreme weather while increasing biodiversity and other urban benefits.^576^

By working across macro and micro scales, interventions can foster more walkable green spaces,^247^ enhancing public health and social interaction while also contributing to broader ES benefits such as enhancing habitats, reducing urban heat, improving air quality, mitigating floods, capturing rainwater, and promoting food production.

Although GBI and walkability are often recognized as complementary components of sustainable urban development, their relationship is complex. The observed disconnection between greenness and walkability highlights the need to move beyond merely co-locating green spaces and pedestrian infrastructure (Figure 5C). Instead, a more integrated, multi-scalar approach is required, incorporating innovative analysis, policymaking, and active public participation. Further research is essential to deepen understanding and strengthen the synergies between GBI and walkability, ensuring that their combined potential can be implemented in future urban environments.

## Conclusion and recommendations

This review presents a comprehensive cross-disciplinary synthesis of underexplored barriers and emerging challenges in GBI implementation within urban environments. By integrating previously fragmented perspectives from urban design, climate science, economics, and social equity research into a cohesive framework, the study bridges disparate knowledge domains and situates GBI within broader urban system dynamics to identify actionable strategies for resilient adoption.

### Environmental barriers

• Potential conflicts between solar energy production and GBI can be resolved through integrated planning approaches that harmonize climate resilience and carbon neutrality goals without sacrificing valuable urban landscapes.
• The fragmentation of GBI research into disciplinary silos prevents full realization of its multifunctional benefits. Interdisciplinary studies integrating physical measurements with human adaptation factors are critical to properly capture GBI’s multifunctionality in urban environments.
• Potential negative impacts of GBI, including effects on air quality, allergen production, GHG emissions, and water/soil dynamics, require thorough consideration during planning stages and continuous monitoring throughout implementation.
• Implementation requires careful consideration of interactions between building systems, ventilation, and climate management across local and wider urban scales to maximize co-benefits and prevent unintended consequences.
• Comprehensive assessment of GBI thermal resilience potential requires integrating physical temperature measurements with subjective human adaptation factors, leveraging technologies such as remote sensing, digital twins, and AI to improve heat risk mapping across demographics.
• Long-term GBI robustness requires science-based plant selection informed by ecological and evolutionary research on species’ adaptability to harsh urban environments and changing climate conditions, creating low-maintenance, long-lived solutions.
• Multi-scalar environmental planning must align interventions across block, neighborhood, watershed, and metropolitan scales, to overcome spatial mismatches between fragmented deployments and broader ecological processes.
• Urban BI requires context-sensitive restoration approaches that balance inherent risks (e.g., flooding and water-borne diseases) with potential benefits to urban character, ESs, and community well-being.

### Social barriers

• Justice frameworks must be embedded throughout planning, design, and governance processes, acknowledging historic inequalities while ensuring inclusive approaches that prevent green gentrification and actively disrupt cycles of environmental marginalization.
• Planning must incorporate traditional ecological knowledge and heritage values beyond tokenistic engagement, as interventions that resonate with community identity enhance both ecological performance and long-term stewardship of GBI.
• Successful implementation requires addressing social adoption barriers through genuine community engagement that aligns technical and ecological priorities with local values, reforming fragmented institutional structures, and ensuring equitable access.
• Safety concerns must be systematically addressed through integrated approaches combining CPTED principles, smart technologies, and community participation to ensure all residents feel secure and welcome.
• The tension between ecological functionality and conventional esthetics can be addressed through design elements that signal human care, institutional reforms that value resilience over immediate visual appeal, and approaches that transform public understanding of “beautiful” landscapes.

### Economic barriers

• Scaling GBI investment requires developing innovative financing mechanisms (eco-bonds, conservation-linked debt) and leveraging new technologies for credible ESG metrics that attract private capital while properly valuing nature’s contribution to economic prosperity.
• Recognizing GBI’s true value requires institutional realignment through standardized capital accounting frameworks such as SEEA, performance-based asset management approaches aligned with ISO 55000, and credible market mechanisms that monetize ES.
• Implementation requires standardized cost-benefit methodologies incorporating real-world proxies, long-term case studies, and life cycle assessments that capture previously overlooked intangible benefits, enabling decision-makers to quantify cultural, health, and biodiversity values despite high upfront costs.

### Governance barriers

• Urban land constraints can be addressed by maximizing existing infrastructure through strategic integration of micro-scale interventions such as green roofs, walls, permeable surfaces, and pocket parks that deliver multiple benefits without requiring land acquisition or contributing to further sprawl.
• Advancing GBI in urban design requires data-driven three-dimensional spatial design strategies, interdisciplinary integration of ecological and gray infrastructure, innovative policies, and active community engagement to elevate GBI from isolated interventions to a foundational element of resilient urban development.
• Implementation demands both strong environmental frameworks across governance levels and reformed educational curricula to equip professionals with systemic thinking skills that build resilience into urban governance structures and outlast electoral cycles.
• Bridging the GBI-walkability disconnect requires multi-scale integration strategies that combine GC networks with streetscape interventions, supported by GIS-powered spatial analysis and inclusive governance mechanisms, to fragmented urban landscapes into interconnected systems.

Twelve integrated recommendations derived from 21 barriers were developed. Each recommendation is mapped to its specific barrier(s) in the list below, with comprehensive linkage details provided in Table S13.

#### Strengthen interdisciplinary collaboration to overcome siloed GBI approaches

Cross-sectoral cooperation among ecologists, planners, engineers, and climate and air pollution scientists can shift the focus from single-issue solutions to multifunctional strategies. Employing ecological production functions and ES frameworks enables holistic planning, enhancing biodiversity, mitigating urban heat, minimizing air pollution, and optimizing energy usage in buildings (see siloization in urban GBI studies and conflict control among urban environmental challenges). This recommendation is aimed at municipal policymakers and planners, fostering inter-agency and cross-sectoral collaboration.

#### Prioritize science-driven species selection and adaptive design to minimize environmental threats

For example, to avoid air quality disbenefits, selection of low-allergenic and insect-pollinated species using tools such as OPALS, can reduce respiratory health impacts in vulnerable groups with higher allergy susceptibility, while choosing plants with minimal bVOC emissions prevents secondary air pollution. Incorporating optimal canopy structures and staggered arrangements maintains street level ventilation and prevents pollution trapping (see unintended consequences and pathways to resilient GBI via plant adaptation). This recommendation is directed at urban ecologists, planners, and public health professionals, encouraging species choice and adaptive design to reduce pollution and allergen risks.

#### Harness microclimate modeling and AI for indoor-outdoor ventilation needs and GBI design

To integrate GBI into urban ventilation strategies, there is a need for strengthening collaboration among planners, architects, ecologists, and health experts. Using urban microclimate models and integrating machine learning with GI mapping can help develop context-specific solutions that preserve airflow, protect HVAC inlets, and prevent heat and pollutant entrapment, while ensuring that vegetation is spatially configured for maximum benefit (see urban ventilation and GBI trade-offs). This recommendation highlights the role of urban planners and ecologists in applying microclimate modeling and AI for evidence-based airflow, heat regulation, and pollution management.

#### Integrate thermal adaptation and vulnerability information into GBI planning

Shift the focus from generalized cooling benefits to nuanced human thermal adaptation, particularly for vulnerable populations. Remote sensing, digital twins, and heat exposure evaluation can identify urban overheating risks to develop targeted interventions that align with both physiological response patterns and microclimate (see thermal resilience and environmental injustice and GBI decision-making”). This recommendation calls on urban planners, public health agencies, and adaptation teams to embed vulnerability mapping into planning to better target interventions for sensitive populations**.**

#### Integrate social dimensions explicitly into GBI research frameworks and performance evaluation

Future studies should systematically incorporate metrics for social acceptance, cultural relevance, safety perception, and equity outcomes when assessing GBI effectiveness, moving beyond purely ecological or technical indicators (see cultural perspectives on GBI, social adoption hindering implementation of GBI, and balancing climate adaptation and esthetic goals in urban GBI). This recommendation is aimed at urban researchers, social scientists, and local governments, embedding equity, cultural acceptance, and community priorities in GBI performance evaluation to embed equity, and cultural and social acceptance.

#### Promote cross-disciplinary collaboration to align GBI design with community realities

GBI planning must co-develop ecological functions and social values by involving social scientists, community groups, and community knowledge holders during initial planning and design phases (see social adoption hindering implementation of GBI and safety and security barriers in GBI implementation). This recommendation emphasizes collaboration between planners, social scientists, local governments, and community groups to co-develop designs that balance ecological benefits with lived social values.

#### Develop context-sensitive policies that balance ecological resilience, public safety, and esthetic expectations

Urban policies should guide GBI implementation to address real and perceived safety concerns, respect diverse cultural esthetics, and ensure equitable access, avoiding one-size-fits-all greening solutions (see safety and security barriers in GBI implementation, balancing climate adaptation and esthetic goals in urban GBI, and land scarcity and urban sprawl). This recommendation is directed at landscape architects and urban designers, ensuring the balance between ecological function and esthetics to guide GBI implementation to align with esthetics and equity.

#### Strengthen the financial ecosystem through standardized ESG metrics and monitoring

Prioritize the development and global adoption of resilient, standardized ESG metrics and risk valuation frameworks, leveraging emerging technologies such as AI, EO, and satellite data. Reliable and comparable data will strengthen GBI valuation, support better risk assessment, and attract sustainable investments (see financial barriers to GBI implementation and challenges in recognizing GBI as assets). This recommendation calls on investors, financial institutions, and local governments to adopt robust and standardized ESG metrics, providing reliable data for valuation, risk assessment, and sustainable investment.

#### Scale innovative financing mechanisms for GBI deployment

Expand the use of green/blue bonds, and debt-for-nature swaps by creating clear, accessible frameworks for environmental and social impact evaluation. Third-party certifications and structured de-risking mechanisms will help build investor confidence and close the biodiversity financing gap (see lack of comprehensive cost-benefit analysis and conflicts in promoting GBI and walkability). This recommendation urges financial institutions and developers to expand access to GBI through frameworks that evaluate and communicate environmental and social impacts.

#### Align financial regulation and incentives with biodiversity and natural capital goals

Incorporate ecological risks into finance regulations and monetary and fiscal policies. Coupled with incentives such as tax benefits and blended finance models, this will encourage larger and more stable investments in GBI (see financial barriers to GBI implementation, challenges in recognizing GBI as assets, and lack of comprehensive cost-benefit analysis). This recommendation highlights the need for policymakers, local governments, and investors to align fiscal and regulatory instruments with biodiversity protection and natural capital objectives.

#### Prioritize micro-scale, three-dimensional GBI to optimize urban land use

Support micro-scale, three-dimensional GBI, including rooftop greenery, vertical gardens, and pocket parks to promote ecological diversity and livability in space-constrained urban areas. Ensure integration into city sustainability plans with long-term maintenance strategies (see land scarcity and urban sprawl). This recommendation is aimed at urban planners, architects, and developers, promoting compact three-dimensional solutions such as green roofs, vertical gardens, and pocket parks.

#### Embed GBI in urban planning through adaptive governance and policy incentives

Position GBI as a core element of urban planning rather than a supplementary feature. Implement integrated spatial planning and foster collaboration across sectors and disciplines. Adopt adaptive governance structures that offer clear incentives for ecological design. Ensure GBI aligns with economic, social, and environmental objectives to support resilient, inclusive cities (see urban design barriers, lack of clear GBI implementation policies, and conflicts in promoting GBI and walkability). This recommendation emphasizes the role of local governments, policymakers, and planners in embedding GBI as core infrastructure through adaptive governance and policy frameworks that integrate economic, social, and environmental priorities.

This synthesized framework advances GBI implementation by connecting theory with practice, enabling a shift from standardized contextually responsive models that reflect local conditions across environmental, social, economic, and governance dimensions. Interdisciplinary collaboration is essential to align diverse stakeholder priorities, supported by long-term monitoring to assess performance and trade-offs. Adaptive governance must reflect complex urban dynamics. The future of GBI lies in science-driven, justice-oriented approaches, transitioning from isolated, esthetic interventions to multifunctional, context-sensitive systems. As climate pressures grow, integration with PVs and data-driven monitoring will enhance energy resilience and urban cooling. Equity, inclusion, and participatory governance must underpin implementation, while embedding natural capital in policy and finance is key to scaling investment and achieving climate-resilient, inclusive urban futures.

## Funding and acknowledgments

This work is carried out under the framework of UKRI (EPSRC, NERC, AHRC) funded RECLAIM Network Plus (EP/W034034/1; EP/W033984/1) and GP4Streets (UKRI1281) projects. P.K. and co-authors acknowledge the support received through the UKRI-funded GreenCities (NE/X002799/1, NE/X002772/1, 10.13039/501100001807FAPESP grant nos. 2016/18438-0, 2019/08783-0, 2022/02365-5, 2022/04619-4, 2024/01097-2, 2024/23425-1, and 25/03337-3), GREENIN Micro Network Plus (APP55977), DEFRAG (NE/W002892/1) and UGPN-funded (UGPN-NBS and GREENICON) projects. All the co-authors contributed equally, and their names are listed in alphabetical order following the core writing team. The funders had no role in study design, data collection and analysis, decision to publish, or preparation of the manuscript.

## Author contributions

Conceptualization, P.K., methods, P.K., K.C.P., A.B., H.S., and A.K.D.; supervision, P.K.; project administration, P.K.; funding, P.K.; data analysis (figures, tables), P.K., K.C.P., A.B., H.S., and A.K.D.; data extraction, K.C.P., A.B., H.S., and A.K.D.; writing – original draft, P.K., K.C.P., A.B., H.S., A.K.D., S.H., and S.K.; writing – review & editing, P.K., K.C.P., A.B., H.S., A.K.D., S.H., S.K., A.A., M.d.F.A., R.A.A., E.A.A.d.S., M.A., C.B.R., P.B., M.L.B., B.G.B., L.F.C., S.-J.C., A.L.C.F.G., R.C., A.K.C.R., B.C., R.M.d.M., L.A.d.P., P.d.S., M.A.F., E.D.F., M.F.F., B.F., J.G., L.L.G., M.J.G.R., C.H.H., WF.H., L.H., C.H., Y.H., L.J., R.J., J.K., M.K., G.M.L., A.A.L.M., J.A.M., L.D.M., M.C.M., R.C.K.M., Y.K.L.K., W.L.A., J.L., G.M.M., S.K.M., M.P.M., M.C.V.M.S., A.Mc., O.M.S., E.M., E.G.S.N., T.N., G.O., R.P., H.P.P., R.P., P.J.P.M., J.A.P., S.P., J.A.P.S., P.L.R.A., N.C.R., A.P.R., D.S., Y.S., V.S., Y.T., T.T.d.A.A., B.L.V.M., F.W., J.W., C.W., H.S.W., Q.W., R.W., C.X., R.Y., and R.Y. The authors’ names appear in alphabetical order after the core writing team. All authors commented on the draft manuscript and assisted in the conceptual development of the text, tables, figures, and the overall cohesiveness and proofreading of the paper.

## Declaration of interests

The authors declare no competing interests.

## Lead contact website

https://www.surrey.ac.uk/people/prashant-kumar and www.surrey.ac.uk/gcare.

## Supplemental information

Document S1. Figures S1–S10, Tables S1–S13, and sections S1–S3

Document S2. Article plus supplemental information
