# Supplementary material for: Overlooked considerations in prescribing green and blue infrastructure solutions for urban environments
Source: Innovation (Camb). 2025 Nov 19;7(5):101184. doi: 10.1016/j.xinn.2025.101184 (PMC13148010; doi:10.1016/j.xinn.2025.101184)
Supplement: Document S1. Figures S1–S10, Tables S1–S13, and sections S1–S3 [file mmc1.pdf]

The Innovation, Volume 7

Supplemental Information

Overlooked considerations in prescribing green and blue infrastructure solutions for urban environments

Prashant Kumar, Karina Corada Perez, Akash Biswal, Hao Sun, Anubhav Kumar Dwivedi, Sarkawt Hama, Soheila Khalili, Ajit Ahlawat, Maria de Fatima Andrade, Ronaldo Adriano Alves, Emannuelly A. Amaral dos Santos, Maria Athanassiadou, Camilo Bastos Ribeiro, Prabin Bhusal, Miguel Luiz Bucalem, Bonnie G. Buchanan, Leticia Figueiredo Candido, Shi-Jie Cao, Amarilis Lucia Casteli Figueiredo Gallardo, Ruidong Chang, Amanda K. Chaves Ribeiro, Brian Considine, Regina Maura de Miranda, Letícia Aparecida de Paiva, Priyanka de Souza, Marco A. Franco, Edmilson D. Freitas, H. Christopher Frey, Marco F. Funari, Bruno Furieri, John Gallagher, Leandro Luiz Giatti, Marcos Jeronimo Goroski Rambalducci, Christos H. Halios, Felicity Harris, Leonardo Hoinaski, Colin Horton, Yuhan Huang, Laurence Jones, Robyn Jones, John Kandulu, Madhusudan Katti, Giuliano Maselli Locosselli, Augusto Akio Lucchezi Miyahara, Jorge Alberto Martins, Leila Droprinchinski Martins, Mauricio Cruz Mantoani, Roberta Consentino Kronka Mülfarth, Yasmin Kaore Lago Kitagawa, Willian Lemker Andreão, Jackson Lemons, Giulia Mariano Machado, Shelagh K. Malham, Meredith P. Martin, Maria Clara V.M. Starling, Aonghus McNabola, Otavio Medeiros Sobrinho, Eugene Mohareb, Erick G. Sperandio Nascimento, Thiago Nogueira, Gwilym Owen, Rajan Parajuli, Hari Prasad Pandey, Rizzieri Pedruzzi, Pedro José Pérez Martínez, Janaina Antonino Pinto, Jorge Armando Piscoya Santibañez, Shila Pokhrel, Paula Lelis Rabelo Albala, Neyval C. Reis, Anderson P. Rudke, Devendra Saroj, Yiming Sui, Veronica Soebarto, Yonatal Tefera, Taciana Toledo de Almeida Albuquerque, Bruna Lima Veras Maia, Fang Wang, Jannis Wenk, Robson Will, Carmel Williams, Hannah Sloan Wood, Qingyun Wu, Chang Xi, Russell Yates, and Runming Yao

Supplementary Information (SI)

for

Overlooked Considerations in Prescribing Green and Blue Infrastructure Solutions for Urban Environments

Prashant Kumar,[1,2,16,19,*] Karina Corada Perez,[3] Akash Biswal,[1] Hao Sun,[1] Anubhav Kumar Dwivedi,[1] Sarkawt Hama,[1] Soheila Khalili,[1,2] Ajit Ahlawat,[4,5] Maria de Fatima Andrade,[6] Ronaldo Adriano Alves,[7] Emannuelly A. Amaral dos Santos,[8] Maria Athanassiadou,[9] Camilo Bastos Ribeiro,[10] Prabin Bhusal,[11,12] Miguel Luiz Bucalem,[13] Bonnie G. Buchanan,[14] Leticia Figueiredo Candido,[15] Shi-Jie Cao,[1,16] Amarilis Lucia Casteli Figueiredo Gallardo,[13,17] Ruidong Chang,[18] Amanda K. Chaves Ribeiro,[8] Brian Considine,[19,41] Regina Maura de Miranda,[20] Letícia Aparecida de Paiva,[21] Priyanka de Souza,[22,23] Marco A. Franco,[6] Edmilson D. Freitas,[6] H. Christopher Frey,[24] Marco F. Funari,[1,2] Bruno Furieri,[25] John Gallagher,[19] Leandro Luiz Giatti,[26] Marcos Jeronimo Goroski Rambalducci,[27] Christos H. Halios,[28] Felicity Haris,[29] Leonardo Hoinaski,[10] Colin Horton,[30] Yuhan Huang,[31] Laurence Jones,[32,33] Robyn Jones,[34] John Kandulu,[35] Madhusudan Katti,[11] Giuliano Maselli Locosselli,[15,36] Augusto Akio Lucchezi Miyahara,[15] Jorge Alberto Martins,[27] Leila Droprinchinski Martins,[27] Mauricio Cruz Mantoani,[6] Roberta Consentino Kronka Mülfarth,[37] Yasmin Kaore Lago Kitagawa,[38] Willian Lemker Andreão,[39] Jackson Lemons,[11] Giulia Mariano Machado,[26] Shelagh K Malham,[40] Meredith P. Martin,[11] Maria Clara V. M. Starling,[8] Aonghus McNabola,[19,41] Otavio Medeiros Sobrinho,[8] Eugene Mohareb,[28] Erick G. Sperandio Nascimento,[1,42,43] Thiago Nogueira,[26] Gwilym Owen,[44] Rajan Parajuli,[11] Hari Prasad Pandey,[45,46] Rizzieri Pedruzzi,[47] Pedro José Pérez Martínez,[21] Janaina Antonino Pinto,[48] Jorge Armando Piscoya Santibañez,[6] Shila Pokhrel,[11,46] Paula Lelis Rabelo Albala,[37,59] Neyval C. Reis,[25] Anderson P. Rudke,[8] Devendra Saroj,[1,2] Yiming Sui,[28] Veronica Soebarto,[18] Yonatal Tefera,[49] Taciana Toledo de Almeida Albuquerque,[8] Bruna Lima Veras Maia,[8] Fang Wang,[50,51,52] Jannis Wenk,[53,54] Robson Will,[10] Carmel Williams,[49] Hannah Sloan Wood,[55] Qingyun Wu,[31] Chang Xi,[16] Russell Yates,[56] Runming Yao,[27,57,58]

[1]Global Centre for Clean Air Research (GCARE), School of Engineering, Civil and Environmental Engineering, Faculty of Engineering and Physical Sciences, University of

---

[*]Correspondence: p.kumar@surrey.ac.uk (P. A.)

Surrey, Guildford GU2 7XH, United Kingdom
[2]Institute for Sustainability, University of Surrey, Guildford GU2 7XH, United Kingdom
[3]Sustainability Research Institute, University of East London, London E16 2RD, United Kingdom
[4]Department of Geoscience and Remote Sensing, Delft University of Technology (TU Delft), Delft 2628 CN, The Netherlands
[5]Leibniz Institute for Tropospheric Research, e.V. (TROPOS), Leipzig 04318, Germany
[6]Institute of Astronomy, Geophysics and Atmospheric Sciences, Department of Atmospheric Sciences, University of São Paulo, São Paulo 05508-090, Brazil
[7] Department of Geography, State University of Londrina, Londrina 86057-970, Brazil
[8]Department of Sanitary and Environmental Engineering, School of Engineering, Federal University of Minas Gerais, Belo Horizonte 31270-901, Brazil
[9]Urban Climate Applications, Met Office, Exeter EX1 3PB, United Kingdom
[10]Department of Sanitary and Environmental Engineering, Federal University of Santa Catarina, Florianópolis 88040-070, Brazil
[11]Department of Forestry and Environmental Resources, North Carolina State University, Raleigh, North Carolina 27695, United States
[12]Institute of Forestry, Tribhuvan University, Pokhara 33700, Nepal
[13]Polytechnic School, University of São Paulo, São Paulo 05508-010, Brazil
[14]Sustainable and Explainable Fintech (SAEF) Center, University of Surrey, Guildford GU2 7XH, United Kingdom
[15]Institute of Environmental Research from the State of São Paulo, São Paulo 01061-970, Brazil
[16]School of Architecture, Southeast University, 2 Sipailou, Nanjing 210096, China
[17]Smart and Sustainable Cities Program at the University Nove de Julho, São Paulo 01525-000, Brazil
[18]School of Architecture and Civil Engineering, The University of Adelaide, Adelaide SA 5005, Australia
[19]Department of Civil, Structural & Environmental Engineering, Trinity College Dublin, the University of Dublin, Dublin D02 PN40, Ireland
[20]School of Arts, Sciences and Humanities, University of São Paulo, São Paulo 03828-000 Brazil

[21]Faculty of Engineering, Architecture and Urbanism, University of Campinas, Campinas13083-970, Brazil
[22]Department of Urban and Regional Planning, University of Colorado Denver, Denver CO 80202, United States
[23]CU Population Center, University of Colorado Boulder, Boulder CO 80302, United States
[24]Department of Civil, Construction, and Environmental Engineering, North Carolina State University, Raleigh NC 27606, United States
[25]Department of Environmental Engineering, Federal University of Espírito Santo, Vitória 29075-910, ES, Brazil
[26]Department of Environmental Health, School of Public Health, University of São Paulo, São Paulo 01246904, Brazil
[27]Federal University of Technology, Parana 80230-910, Brazil
[28]School of Built Environment, University of Reading, Reading RG6 6DF, United Kingdom
[29]Portsmouth City Council, Portsmouth PO1 2AL, United Kingdom
[30]Rugby Borough Council, Rugby CV21 2RR, United Kingdom
[31]Centre for Green Technology, School of Civil and Environmental Engineering, University of Technology Sydney, NSW 2007, Australia
[32]UK Centre for Ecology & Hydrology, Deiniol Road, Bangor LL57 2UW, United Kingdom
[33]Liverpool Hope University, Department of Geography and Environmental Science, Liverpool L16 9JD, United Kingdom
[34]School of Psychology and Sport Science, Bangor University, Bangor LL57 2DG, United Kingdom
[35]College of Business, Government and Law, Flinders University, Bedford Park, South Australia 5042, Australia
[36]Center for Nuclear Energy in Agriculture, University of São Paulo, São Paulo 13416-000, Brazil
[37]Faculty of Architecture, Urbanism and Design, University of São Paulo, São Paulo 05508-080, Brazil
[38]Foundation for Scientific and Cultural Development, Federal University of Lavras, Lavras 37203-202, Brazil
[39]ArcelorMittal, Global Research and Development, Espirito Santo 29161-376, Brazil, Brazil

[40]School of Ocean Sciences, Bangor University, Menai Bridge, Anglesey LL59 5AU, United Kingdom
[41]School of Engineering, RMIT University, Melbourne VIC 3000, Australia
[42]Surrey Institute for People-Centred Artificial Intelligence, Faculty of Engineering and Physical Sciences, University of Surrey, Guildford GU2 7XH, United Kingdom
[43]Stricto Sensu Department, SENAI CIMATEC University, Salvador 41650-010, Brazil
[44]Resilience Unit, Cardiff Council, Cardiff CF10 4UW, United Kingdom
[45]University of Southern Queensland, Toowoomba, Queensland 4350, Australia
[46]Ministry of Forests and Environment, Government of Nepal, Kathmandu 44600, Nepal
[47]Department of Sanitary and Environmental Engineering, Rio de Janeiro State University. Rio de Janeiro 20550-013, Brazil
[48]Department of Infrastructure and Environment, Faculty of Civil Engineering, Architecture and Urbanism, State University of Campinas, São Paulo 13083-889, Brazil
[49]School of Public Health, The University of Adelaide, Adelaide SA 5005, Australia
[50]State Key Laboratory of Soil and Sustainable Agriculture, Institute of Soil Science, Chinese Academy of Sciences, Nanjing 210008, China
[51]University of Chinese Academy of Sciences, Beijing 100049, China
[52]Joint FAO/IAEA Centre of Nuclear Techniques in Food and Agriculture, International Atomic Energy Agency, Vienna 1400, Austria
[53]Federal Institute of Hydrology (BfG), Department G - Qualitative Hydrology, Am Mainzer Tor 1, Koblenz 56068, Germany
[54]Department of Chemical Engineering, University of Bath, BA2 7AY, United Kingdom
[55]Independent Consultant, Copenhagen 2200, Denmark
[56]Surrey County Council, Woodhatch, Reigate RH2 8EF, United Kingdom
[57]Joint International Research Laboratory of Green Buildings and Built Environments (Ministry of Education), Chongqing University, Chongqing 400045, China
[58]National Centre for International Research of Low-carbon and Green Buildings (Ministry of Science and Technology), Chongqing University, Chongqing 400045, China
[59]Faculty of Architecture and Urbanism, University of Brasília, Brasília 70904-900, Brazil

This document includes:

- Figures S1-S10
- Tables S1-S13
- Sections S1-S3

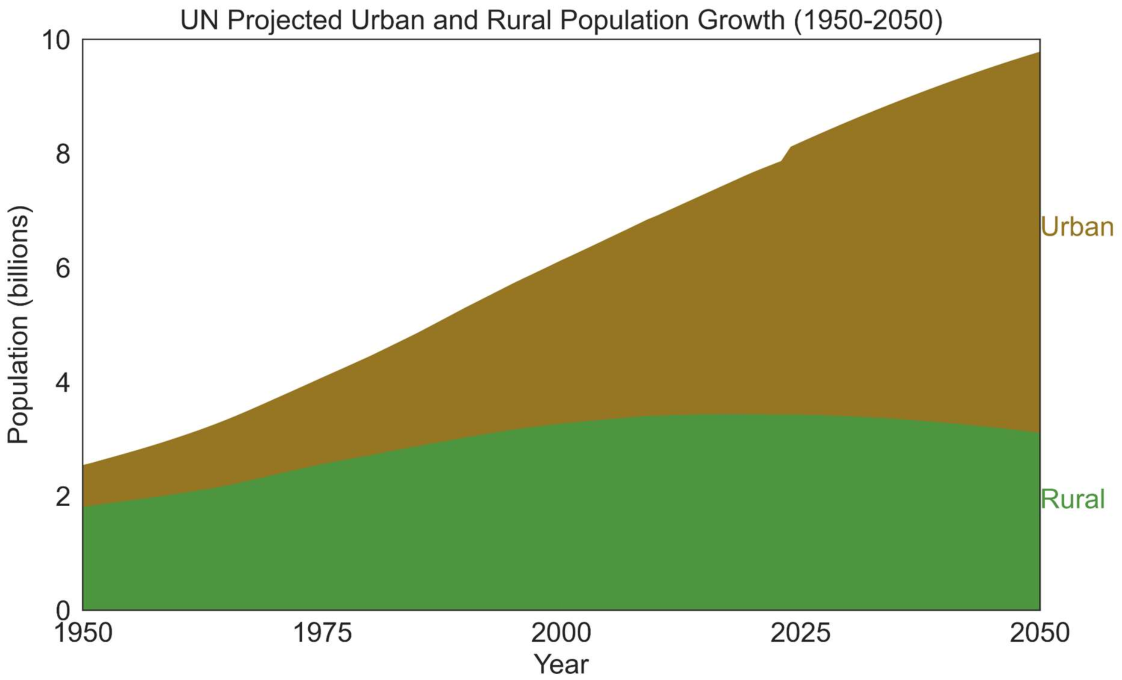

Figure S1. UN projected urban and rural population growth from 1950-2050 (Data Source: https://ourworldindata.org/urbanization).

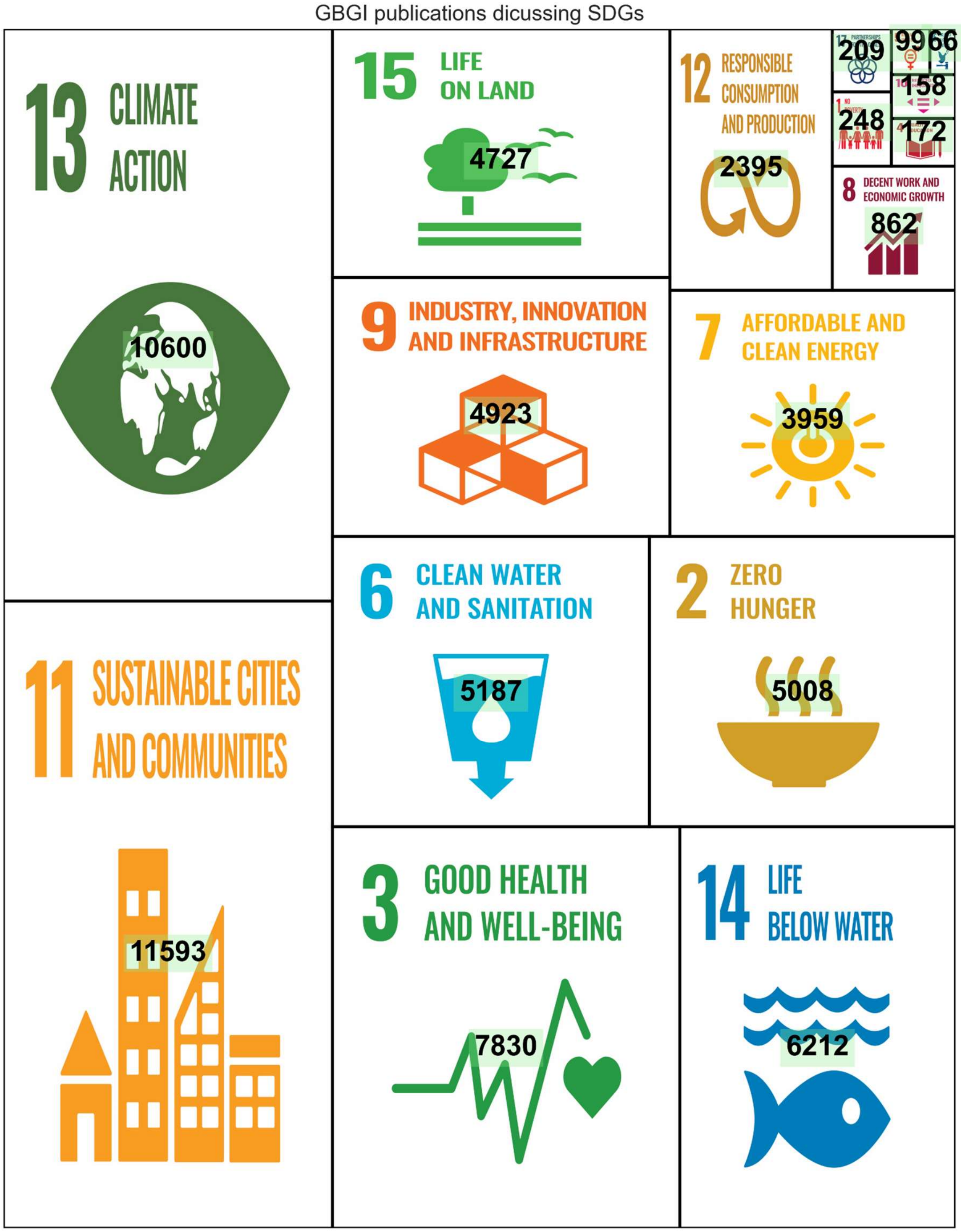

Figure S2. Treemap visualisation of publication records obtained after the Web of Science portal (Clarivate) using the search query: Green Infrastructure (All Fields) OR Nature-based Solutions (All Fields) AND "Air Pollution" (Keyword Plus®) AND "Climate Change" (Keyword Plus®) AND "Temperature" (Keyword Plus®) AND "Flooding" (Keyword Plus®). The data reflects the distribution of research focused on the intersection of urban environmental challenges and nature-based mitigation strategies. The size of box is an indication for the number of publications linked with the SDGs and the text inside the light green box shows the number of matching publications.

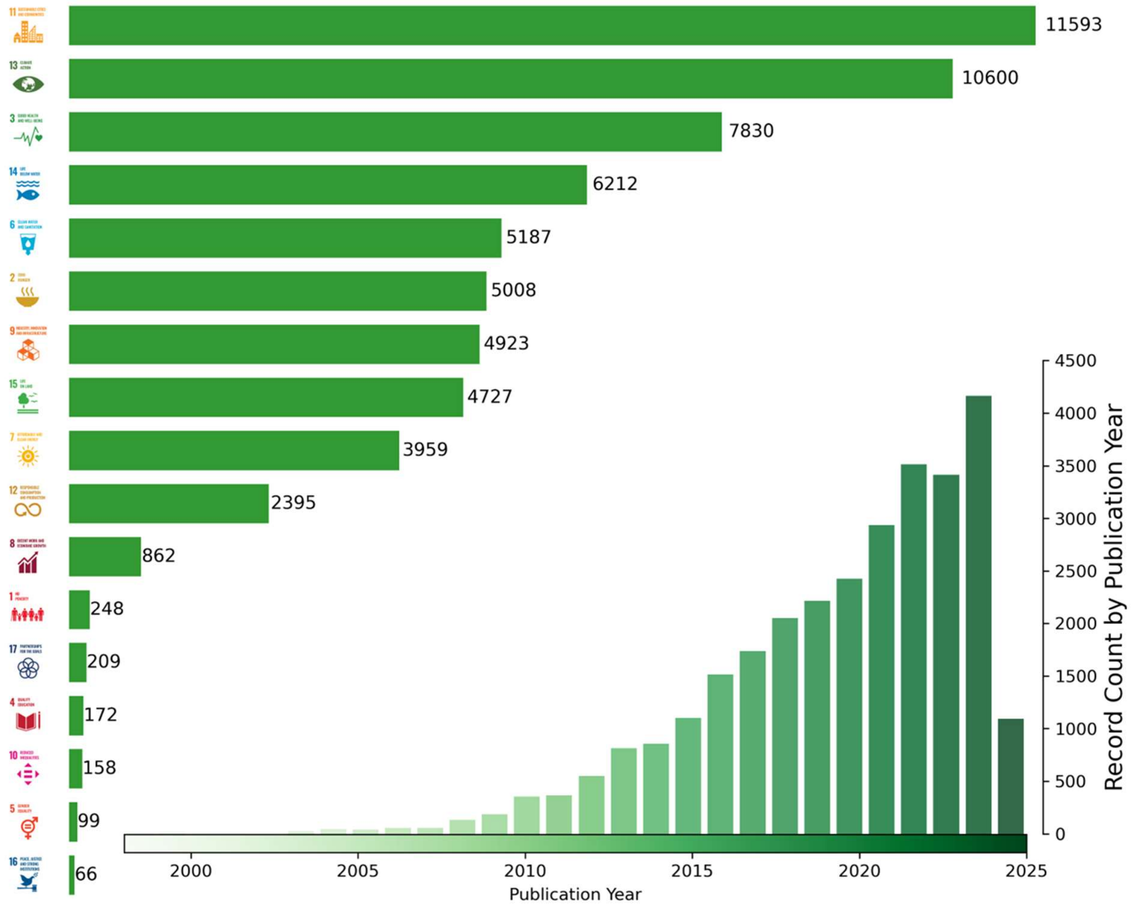

Figure S3. The horizontal bars represent the total number of publications associated with each SDG, and the vertical bar chart in the bottom shows the total volume of publications discussing the GBGI papers. The publication records obtained from the Web of Science portal (Clarivate) using the search query: Green Infrastructure (All Fields) OR Nature-based Solutions (All Fields) AND "Air Pollution" (Keyword Plus®) AND "Climate Change" (Keyword Plus®) AND "Temperature" (Keyword Plus®) AND "Flooding" (Keyword Plus®).

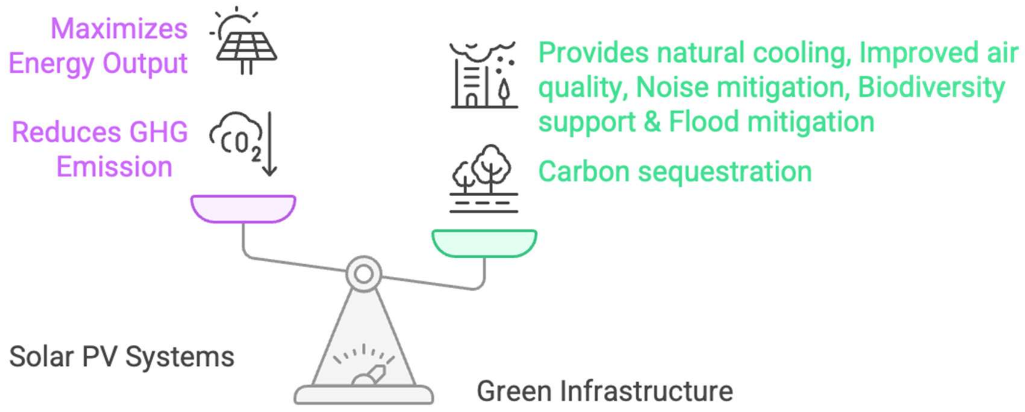

Figure S4. Balance model comparing solar PV Systems and GI. PV offers energy maximisation and GHG reduction, while GI provides natural cooling, improved air quality, noise mitigation, biodiversity support, and flood mitigation, in addition to carbon sequestration. GI delivers broader ecosystem services, though it may also compete with PV systems for available space.

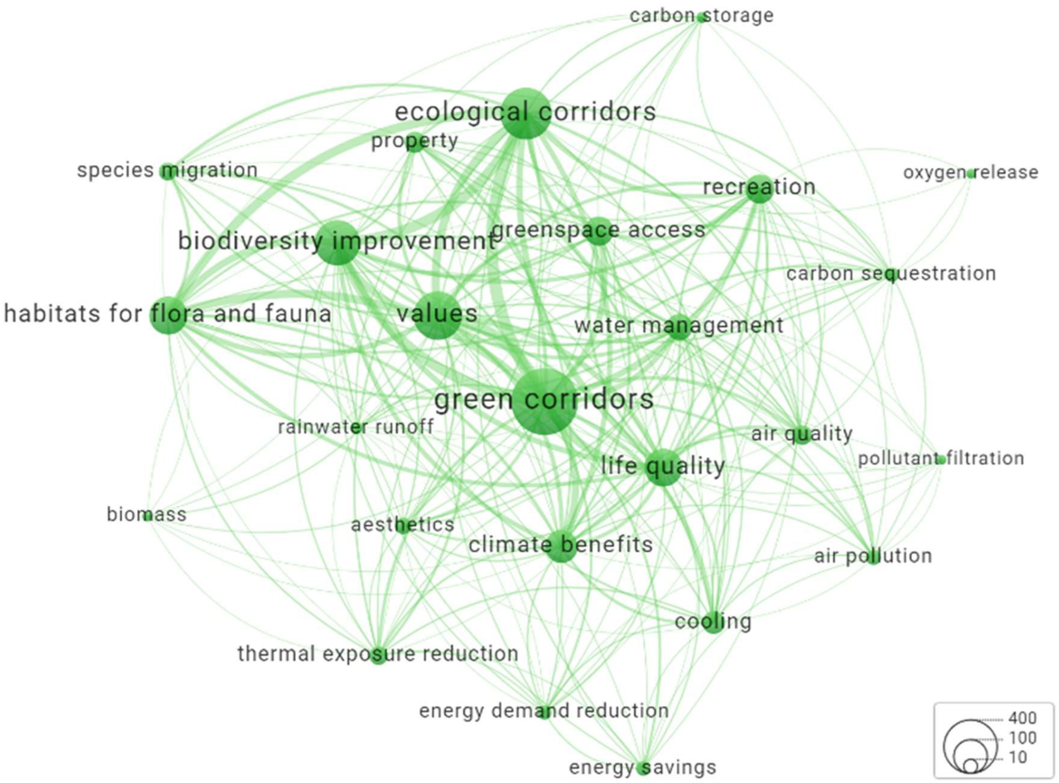

Figure S5. Results of a bibliometric analysis: thematic map obtained from 751 publications screened for the following keywords: climate benefits, thermal exposure reduction, cooling, biodiversity improvement, species migration, habitats for flora and fauna, greenspace access, energy savings, energy demand reduction, water management, rainwater runoff, air pollution, air quality, pollutant filtration, oxygen Release, biomass, carbon storage, carbon sequestration, recreation, aesthetics, life quality, property, values.

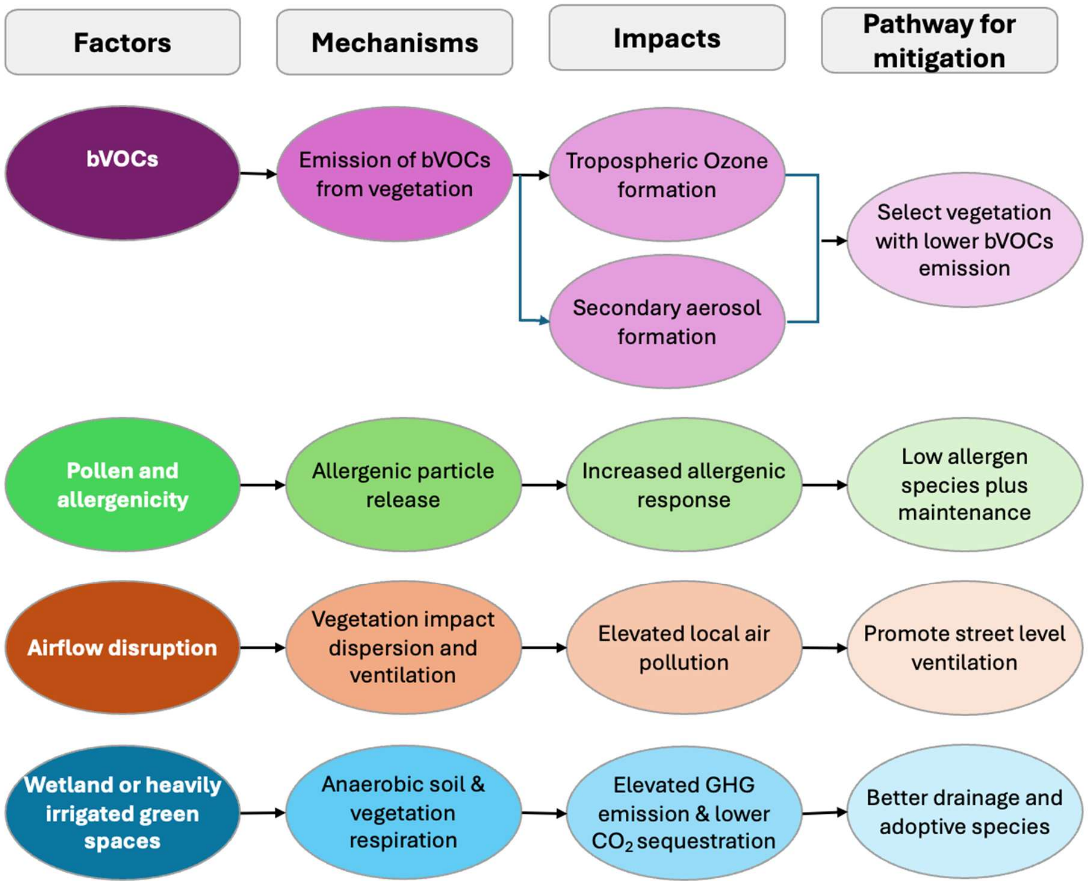

Figure S6. Conceptual diagram illustrating unintended consequences hindering the implementation of GBI. The image shows how factors like bVOC emissions, allergenic pollen, airflow disruption, and GHG release can lead to environmental and health impacts, along with strategies to mitigate each.

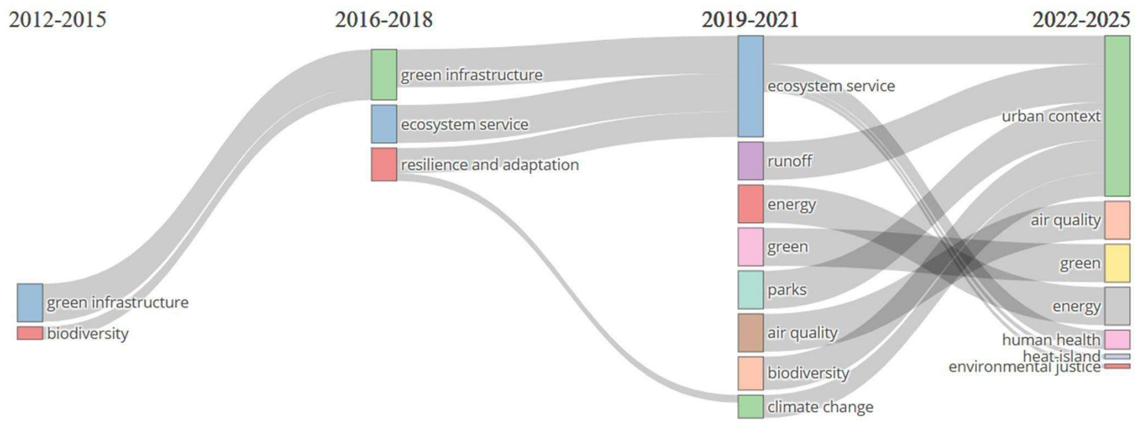

Figure S7. Thematic Evolution of GBI Research from 2012 to 2025. (Search string: "green infrastructure" OR "blue infrastructure" OR "green-blue infrastructure" OR "nature-based solutions" AND ("mitigate" OR "adapt" OR "address") AND ("environmental challenges" OR "environmental problems" OR "environmental issues" OR "urban resilience"). Database: Scopus and Web of Science).

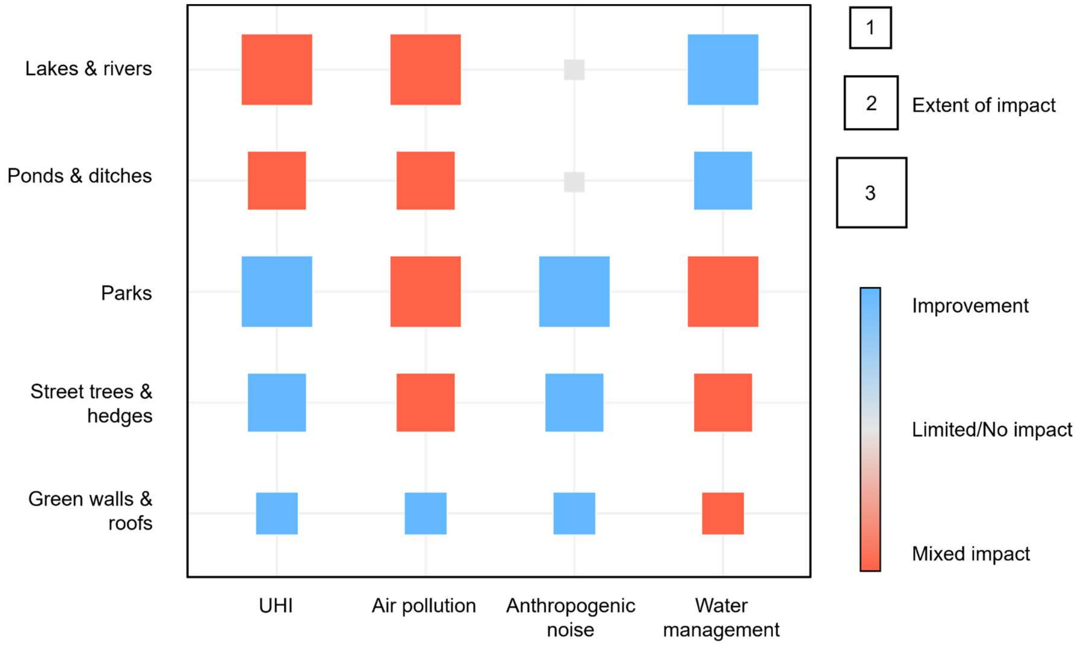

Figure S8. A visual matrix illustrating the diverse impacts of different GBI types on key urban challenges. Square colour and size indicate the type and relative magnitude of influence that each GBI type has on a given urban challenge, based on the authors' synthesis of literature. Detailed corresponding information can be found in Table S5.

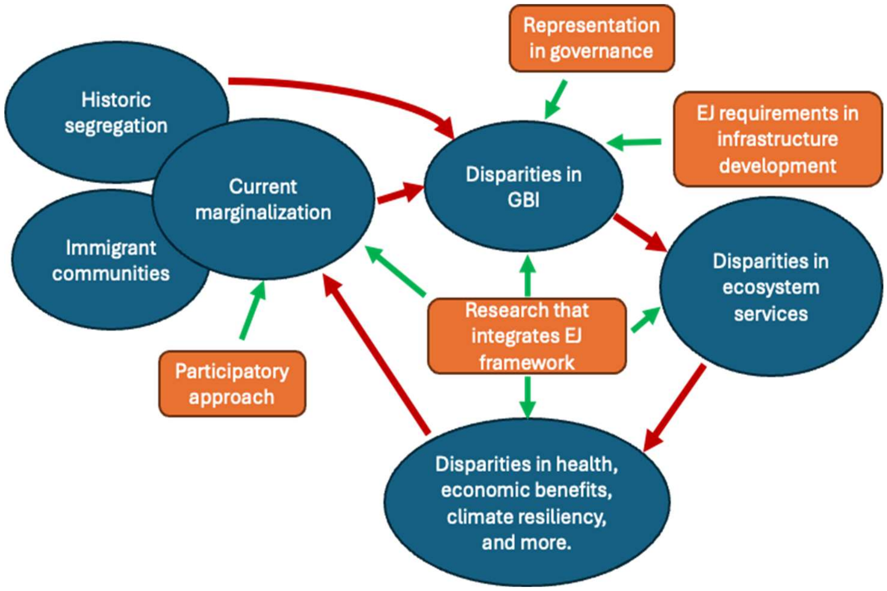

Figure S9. Framework linking socio-economic marginalisation with disparities in ecosystem services and well-being (blue circles and red arrows), with policy interventions points highlighted by orange box.

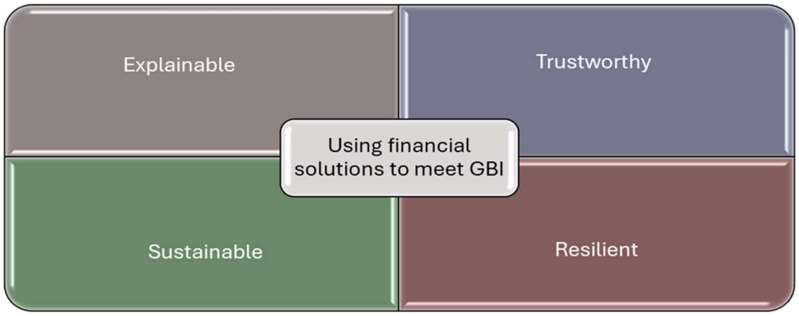

Figure S10. Building a strong financial ecosystem to support Green and Blue Infrastructure. The framework illustrates the four foundational pillars essential for mobilizing financial solutions that effectively support the implementation and scaling of GBI.

S1. Co-design process for barrier identification

The identification and selection of the barriers presented in this study were the result of a rigorous, multi-stage participatory process designed to leverage diverse expertise and build consensus. The three-step process involved: (1) Initial Identification via a large interdisciplinary RECLAIM Network Plus workshop, with over 60 participants, held in-person at the University of Surrey, Guildford, UK, on 30 January 2025 (RECLAIM 2025). (2) Independent Refinement by a multidisciplinary expert group in-person meeting of 20 participants at North Carolina State University (NCSU), Raleigh, NC, USA, on 26 March 2025. (3) Finalisation through an iteration involving all authors named in this manuscript to ensure a balanced, non-redundant list of 'overlooked' barriers.

The process began with a large, interdisciplinary RECLAIM Network Plus workshop (RECLAIM 2025), which generated an initial longlist of potential barriers through structured panel discussions and breakout sessions. This workshop included experts from councils, local government, policy makers, research funders, and collaborators from academic and non-academic sectors such as charity organisations and businesses. To refine this extensive long-list, a second, more focused workshop was convened with approximately 20 multidisciplinary experts at NCSU, the majority of whom had not been involved in the generation of the long-list, and provided a second independent expert opinion. This group - comprising engineers, social scientists, computer scientists, mathematicians, ecologists, urban planners, environmental policy, and climate scientists - collectively screened and prioritised the topics to agree upon a coherent, shorter list.

To ensure inclusivity and final consensus, this refined list was then circulated to the entire author team, including members who could not attend the previous workshops. This third stage was crucial for eliminating repetition, balancing the coverage of topics, identifying and matching their expertise, and verifying that each barrier met the core criterion of being 'overlooked' in current planning and policy.

This entire collaborative effort was co-ordinated by the lead author through one-to-one discussions, and a shared open-access working draft online manuscript outline. This platform allowed all contributors to openly comment, critique, and support suggestions in real-time, creating a transparent and iterative workflow that directly shaped the final selection and framing of the barriers presented herein.

S2. Overcoming siloisation in urban GBI studies

The bibliometric analysis was produced with 753 English language publications from the Scopus online database, using the keywords “Green Corridors” OR “Ecological Corridors”. Publications were then screened for the presence of the above predefined keywords. The screening involved searching across titles, abstracts, author keywords, and index keywords for specific terms associated with each of the predefined keywords. Variations in wording and conceptually equivalent expressions were also considered indicative of keyword presence and the resulting keyword list was then used to replace the original author keywords in the dataset. This standardised keyword dataset was subsequently imported into VOSviewer to generate a co-occurrence map of the relationships between “Green Corridors”, “Ecological Corridors” and the other thematic categories as shown in Figure S5.

A search within the results was conducted for keywords in the following eight ecosystem service categories: 1. climate benefits; thermal exposure reduction; cooling 2. biodiversity improvement; species migration; habitats for flora and fauna 3. greenspace access 4. energy savings; energy demand reduction 5. water management; rainwater runoff 6. air pollution; air quality; pollutant filtration; oxygen release 7. biomass; carbon storage; carbon sequestration 8. recreation; aesthetics; life quality; property; values. Keywords from four or more of the above categories appeared in 12 studies which were downloaded and examined. Seven studies were excluded, and the remaining five studies are presented in Table S2.

S3. Strategies to mitigate environmental challenges

To explore how scholarly research has addressed fragmented approaches to GBI implementation - particularly in relation to the mitigation, adaptation, and management of environmental challenges - a comprehensive literature search was conducted using the Scopus (n = 207) and Web of Science (n = 305) databases. The following search string was applied: ("green infrastructure" OR "blue infrastructure" OR "green-blue infrastructure" OR "nature-based solutions") AND ("mitigate" OR "adapt" OR "address") AND ("environmental challenges" OR "environmental problems" OR "environmental issues" OR "urban resilience"). After the removal of duplicate records, a total of 335 unique articles were kept for bibliometric and thematic analysis. Bibliometric analyses, including thematic mapping and thematic evolution, were performed using the Bibliometrix R package (Aria and Cuccurullo, 2017) by applying the Biblioshiny interface. Thematic maps were based on keyword co-occurrence networks, analysed through clustering algorithms and plotted according to their centrality and density. Thematic evolution was examined across four time slices corresponding to 3-year periods (2012–2015, 2016–2018, 2019–2021, and 2022–2025) to enable an analysis of the development and growth of research within this topic over time. Over the past decade, research on GBI has undergone a conceptual transformation (Figure S7). This visual representation maps how scholarly interest has progressed in response to environmental challenges.

Table S1. Deeper analysis of case studies, detailing GBGI type, challenges, trade-off, failures and references with corss-link to relevant paper sections for further context.

GBGI types used in case study	Challenges addressed/ faced	Trade-offs	Failure (partial or full)	Reference: author (year)	Link to manuscript section
Urban trees	Demonstrated carbon mitigation potential: urban trees offset ~17% of carbon emissions over 60 years	Long-term carbon benefits but limited integration with renewable generation options	Partial: effective over long timescales, but does not address immediate emission reduction needs	Grossi et al. (2023)	GBI conflicts with Net Zero
Urban trees	Trees reduce solar roof radiation by ~1% after 20 years of growth	Trade-off between tree canopy benefits vs. rooftop PV efficiency; some argue PV delivers greater carbon reduction than trees	Partial: tree benefits remain, but PV may outperform in emission reduction	Reitberger et al. (2025)	GBI conflicts with Net Zero
Green corridors	Multi-functionality (water storage/purification, local climate regulation, aesthetics) and biodiversity & energy flow (species connectivity, energy patterns via least-cost path and InVest)	Balancing ecological restoration and multifunctional ES delivery with infrastructure expansion and connectivity	Partial: improvements achieved in ES and connectivity, but gaps in ecological resilience and ecosystem functions remained	Wong et al., (2018); Tao et al., (2022)	Siloisation in urban GBI studies
Green corridors	Well-being & accessibility: studies highlighted recreation and life quality benefits using literature and online tools	Trade-off between limited quantitative ecological data and qualitative life-quality outcomes	Partial: broadened focus beyond biodiversity, but methods less robust	Larrea et al. (2024); Mancilla et al. (2024)	Siloisation in urban GBI studies

Green corridor	Biodiversity & energy flow: analysed species movement resistance and energy flow with InVest + least-cost path	Balancing urban development with ecological connectivity	Partial: useful for planning, but still siloed in selected themes	Isola et al. (2024)	Siloisation in urban GBI studies
Wetlands	Designed for nitrogen removal and water regulation but can increase CH_4 and CO_2 emissions	Wetlands designed to reduce nitrogen loads can have low carbon impacts and affect wildlife.	Partial: Poorly implemented wetland restoration programs can result in the loss or perceived loss of community use rights	Li et al. (2024); Yuan et al. (2025)	Unintended consequences
Urban trees	Noise reduction: increased tree crown volume and canopy linked to decreased noise levels	Focused on acoustic benefits, less attention to other ecosystem services	Partial: case-specific evidence, broader transferability uncertain	Zhao et al. (2021)	Conflict control among urban environmental challenges
Green roof	Seasonal impact of green roofs on outdoor fresh air intakes via HVAC filters around 14% reduction in ozone	Ozone removal benefit but higher microbial and VOC fluxes transfer from green roof to HVAC system	Partial: green roof has benefits for ozone removal but impact of PM ingress on HVAC filter loading not addressed	Ramasubramanian et al. (2021)	Urban ventilation
Green roof	Demonstrated ambient PM reductions - 31.8% after 1 year of growth	Enhances PM removal but the impact on a HVAC system fresh air intake is not clear	Partial: effective at PM removal on rooftop but limited to local scale	Kostadinović et al. (2023)	Urban ventilation
Schoolyard greening	Heatwave resilience: transforming schoolyards into urban cool islands, accessible to communities during extreme heat	Trade-off between limited space use and multifunctional adaptation benefits	Partial: success in pilot schools, broader city-wide impacts still scaling	Climate-ADAPT (2022)	Thermal resilience

Urban trees	Stress tolerance during severe drought: tree showed increased photosynthetic activity and growth	Focus on single species; broader applicability to other urban tree species uncertain	Partial: demonstrates strong resilience, but not generalisable across all GBI species	Locosselli et al. (2024)	Pathways to Resilient GBI via Plant Adaptation
Rooftop farming	Food-water-energy nexus: open-air roofs produced up to 7.44 kg vegetables/m² annually and reduced upstream energy & water footprints by a factor of 4.5	Trade-off between maximizing food production and rooftop space available for other GBGI or PV systems	Partial: strong potential, but scalability and long-term viability depend on local context	Yuan et al. (2025)	GBI trade-offs
Urban trees	Redlining and racial segregation created lasting disparities in canopy, impervious cover, air quality, heat, biodiversity, and health	Unequal GBGI distribution across racial groups	Partial: inequities persist post-redlining	Gibbons (2023); Benz & Burney (2021)	Environmental injustice and GBI decision making
Classical Chinese gardens	Demonstrates cultural-ecological integration; regulates microclimate	Strong cultural embedment but limited scalability to urban level	Partial: effective at garden scale, limited in urban upscaling	Xiong et al. (2020)	Cultural perspectives on GBI
Urban parks	Promoted stronger community attachment compared to fragmented/inaccessible spaces	Centralisation boosts social cohesion but risks unequal access for distant residents	Partial: effective for attached users, limited inclusivity across city	Zhu et al. (2017)	Social adoption hindering implementation of GBI
Small urban parks and greenways	Safety concerns over drug use and antisocial behaviour led to redesign	Improved surveillance may reduce natural character	Partial: redesign improved safety but altered original intent	Rigolon & Christensen (2019)	Safety and security barriers in GBI implementation
Urban meadows	Shift from ornamental lawns to multifunctional, climate-adaptive landscapes	Enhances biodiversity and resilience but challenges traditional aesthetic norms	Partial: ecological benefits achieved, but resistance from	Marshall et al. (2023)	Balancing climate adaptation and aesthetic goals in urban GBI

			conventional aesthetic expectations		
Green/blue bonds and debt-for-nature swaps	Difficulty securing finance; biodiversity funding gap persists	Credible certification ensures investor trust, but depends on global financial markets and risk pricing	Partial: successful in some countries (e.g., Seychelles, Belize, Ecuador) but large biodiversity finance gap persists	Flammer (2020, 2021); Schweinberger (2023)	Financial barriers to GBI implementation
Parks, urban vegetation, and wetlands	Quantified environmental value but excluded from formal accounting; often treated as liabilities	Developers profit from green proximity, but local councils lack revenue for upkeep; hybrid grey-green benefits undercounted	Partial: undervaluation limits financing options and institutional support	Matsler (2019); Kennedy et al. (2023)	Challenges in recognising GBI as assets
Green roofs	Faced high upfront construction costs, uncertain long-term return on investment, and maintenance challenges	Economically viable only with subsidies due to high construction and maintenance costs. Even with subsidies, many projects yield zero or negative net present values (NPV)	Partial failure in cases without sustained subsidies or incentive-based programs; risk of abandonment when costs outweigh benefits	Nordman et al. (2018)	Lack of comprehensive cost-benefit analysis (CBA)
Green roofs, walls, permeable surfaces,, pocket parks	Land scarcity and sprawl limit space; rising land values prioritise densification; peri-urban expansion fragments ecosystems	Space-efficient retrofits provide ES but need maintenance; peri-urban GBI often isolated	Partial: microscale effective but limited in scale and connectivity	Tache et al. (2024); Bressane et al. (2024)	Land scarcity and urban sprawl

Rooftop farms, permeable pavements, bio-retention, modular planters	Technical conflicts, poor multi-scale coordination, policy inertia, and weak maintenance planning	3D and multifunctional GBI enhance ES but face cost and governance trade-offs	Partial: pilots (e.g., Malmö) succeed but grey bias dominates	Wang et al. (2025); Kramer et al. (2024)	Urban design barriers
Parks, corridors, hybrid green-grey systems	Lack of clear policies; weak leadership, fragmented governance, competing grey priorities	Long-term benefits but low short-term political visibility; grey favoured	Partial: gradual shifts (e.g., Melbourne Grey-to-Green) but slow elsewhere	Abdallah et al. (2025)	Lack of clear GBI implementation policies
Green corridors, linear parks, stormwater-linked streetscapes	Poor GBI–walkability integration; car-centric planning, grey engineering, land costs	Walkability and ES gains need space reallocation and upfront cost	Partial: co-location common but true integration rare	Wedyan et al. (2025); Petersen et al. (2024)	Conflicts in promoting GBI and walkability

Table S2. Summary of key review papers on solar PV and green infrastructure integration (2010-2025). Literature addressing conflicts, synergies, impacts, and frameworks for balancing renewable energy with conservation of GI.

Author (year)	Key focus area of review	Main findings
Pandey et al. (2025a)	How agrivoltaic systems integrate agriculture and solar energy production to enhance land-use efficiency, profitability, and environmental sustainability.	Agrivoltaic systems enhance water efficiency (150-300%), improve land use (200%), and increase revenue (15x), despite higher installation costs (5-40%), offering competitive returns (payback <10 years) and revenue diversification, especially in arid regions.
Reitberger et al. (2025)	Reduction of solar radiation on the roof as a result of tree growth.	Tree growth over 20 years only reduced solar radiation on the roof by 1%.
Skandalos & Karamanis (2025)	An integrated approach to rooftop PV and green infrastructure under present and future European climate scenarios.	NbS solutions such as tree planting, green roofs, and green walls offer significant carbon sequestration benefits that complement those provided by solar PV installations. Additionally, tree shading contributes to annual reductions in cooling demand.
Asa'a et al. (2024)	Agrivoltaic systems that combine solar energy generation with crop cultivation for optimized land use.	Agrivoltaics increases land use efficiency, creates beneficial microclimates, and shows crop-specific shade responses with PV design optimization being critical for balancing energy and agricultural production.
Dunlap et al. (2024)	Impact of intensive solar development in California.	To maximise solar energy produced, intensive solar development can have negative impacts on nature and green spaces.
Oliveira et al. (2024)	Ecological impacts of floating photovoltaics (FPV) on aquatic organisms across different taxonomic groups, identifying research trends, knowledge gaps, and potential effects on aquatic biodiversity.	FPV generally reduces algal growth and primary production, with effects increasing at higher coverage. Impacts on aquatic organisms are complex, including altered community structures and physiological changes, but research remains limited despite rapid industry expansion.
Essak & Ghosh (2023)	Floating photovoltaics (FPV) as a growing renewable energy technology, analysing its benefits over ground-mounted systems, ecological impacts, applications across different water bodies, and global implementation potential.	FPV offers 3-12.5% higher efficiency than ground-mounted systems, reduces water evaporation, addresses land scarcity, and shows strong growth potential. However, impacts on water quality and ecology remain understudied and require further research.
Grossi et al. (2023)	Potential of trees to absorb carbon emissions in Montreal.	Trees can reduce carbon emissions of a building with BIPV by 17% even before accounting for the on-site electricity generation.

Liu et al. (2023)	Integration of solar energy technologies and NBS to achieve carbon neutrality.	Incorporating NBS is important to maximise resilient benefits. Solar energy technologies alone are not enough to achieve carbon neutrality.
Wang et al. (2023a)	Integration of photovoltaic systems with green roofs and facades to create sustainable, energy-efficient buildings with multiple environmental benefits.	The combination of building-integrated PV with GI provides mutual benefits, with vegetation cooling photovoltaic panels to improve efficiency (1-3%) while optimizing space usage, though performance depends on installation distance, plant species, and microclimate.
Zhang et al. (2023a)	Benefits and potential environmental impacts of implementing PV technologies.	Power generation with PV reduces pollution although also has potential negative impacts, e.g. toxic chemicals in PV materials production and can negatively impact vulnerable lands and ecological functions.
Kruitwagen et al. (2021)	A comprehensive global database of photovoltaic installations across commercial, industrial, and utility scales.	The data indicate that the majority of solar PV facilities are located on croplands, arid regions, and grasslands.
Dhar et al. (2020)	Environmental challenges linked to solar and wind energy development, along with mitigation strategies and potential reclamation practices.	Wind and solar energy installations can adversely affect land, native vegetation, and biodiversity; however, these impacts can be significantly reduced by incorporating reclamation into the planning phase and applying best management practices.
Zhong & Tong (2020)	Factors that affect the performance of PV systems and proposed a new spatial optimization method to maximise PV performance.	Different PV panel orientations and alignment scenarios can be optimised to maximise energy production.
Hernandez et al. (2014)	USSE development has direct and indirect effects on biodiversity, land-use change, soils, water resources, and human health.	Several USSE characteristics and development strategies have low environmental impacts and therefore opportunities exist to minimize impacts, including having proper inventory and monetizing the real value of natural capital and ES of land.
Anders et al. (2010)	Laws in the United States at the state level on access to direct sunlight.	Removing vegetation is allowed by law in the US if the vegetation comprises the performance of rooftop solar PV.

Table S3. Summary of papers examining four or more ecosystem service categories associated with Urban Green Corridors.

Author (Year)	Aim	Methods	Conclusion	ES Studied
Wong et al. (2018)	Examine the impact of a GC in multiple ES. Beijing, China.	Ecological Production Functions methodology assessed the impact of the GC before and after its construction.	During landscape design ecosystem functions need to be considered in order to avoid shortfalls. Multi-functionality can be obtained by pairing green and built infrastructure.	Regulation of the local climate, water storage and purification, aesthetics.
Tao et al. (2022)	Establish an evaluation framework aiming to ecological restoration and protection at multiple scales . Nanjing, China.	ESs quantified by InVEST. An Ordered Weighted Averaging model simulated areas that were selected for their ecological conservation importance. Circuit theory identified ecological sources, corridors, barriers and pinch points at the city and main urban scales.	Many GC and ecological barriers identified at the small and urban scales, indicating that studying large-scale ecological networks at city scale only cannot fully reflect the challenges involved.	Habitat improvement carbon storage, nitrogen retention, and water conservation.
Larrea et al. (2024); Mancilla et al. (2024)	Design of a GC that aims to ecosystem restoration, improvement of green spaces, connectivity and revaluing of the urban landscape. Cusco, Peru.	Non-experimental approach: literature analysis, landscape analysis with climate consultant, planimetry, 3D survey.	A case study of botanical gardens including biofilters, terraces and guinea pigs farms contributes to water quality, energy generation and habitat for flora and fauna.	Energy generation, recreational areas, water purification, flora and fauna habitat.
Isola et al. (2024)	A methodology to approach UGI, as a network of areas connected by urban GCs. Cagliari, Italy.	a. Spatial taxonomy of GI with Ess (InVEST model); b. GCs identified with Least-Cost Path analysis c. Linear Regression between the two.	Results allow the quantitative profile of GI that contributes as a structural reference for urban policies.	Recreation opportunities; water retainment capacity; carbon sequestration and storage; habitat quality; cooling capacity.

Table S4. Summary of studies outlining unintended consequences of GBI implementation. The table provides an overview of selected studies that examine unintended consequences associated with the implementation of GBI. Each entry includes the reference, a brief description of the study and the specific unintended consequence, GHG, bVOC, pollen emission, allergenicity and airflow disruption.

Author (Year)	Description	Unintended consequence addressed
Nowak and Crane (2002)	The article quantifies carbon storage but warns that poorly managed vegetation may also emit CO_2, reducing net sequestration gains.	GHG
Pinto et al. (2010)	Review of O_3 effects on bVOC emissions, highlighting positive feedback that enhance SOA formation.	bVOC
Cariñanos and Casares-Porcel (2011)	This study addresses how specific urban planting strategies can unintentionally increase allergy incidence due to the use of highly allergenic species.	Pollen and allergenicity
Pugh et al. (2012)	This study demonstrates that certain green GI arrangements can trap air pollutants instead of dispersing them, worsening local air quality.	Airflow disruption
Calfapietra et al. (2013)	Examines how biogenic VOC emissions from urban trees contribute to ozone formation, emphasizing differences among species and associated feedback mechanisms.	bVOC
Nowak et al. (2013)	Assessed the life-cycle carbon impact of urban trees.	GHG
Janhäll (2015)	The review discusses how tall or improperly placed vegetation can reduce air dilution and lead to pollutant buildup in urban microenvironments.	Airflow disruption
Chen et al. (2016)	This study evaluated different species to improve air quality at an urban level.	Airflow disruption
Livesley et al. (2016)	This study shows that urban green areas with high vegetation density may enhance nocturnal CO_2 accumulation, especially in areas with limited airflow.	GHG
Säumel et al. (2016)	This study shows the maintenance activities such as pruning, irrigation, and waste management can generate CO_2 emissions that partially offset sequestration benefits.	GHG
Velasco et al. (2016)	The study found that the capacity of urban greenery in (sub)tropical areas to sequester CO_2 is relatively constrained. In Mexico City it acted as a small sink, while in Singapore it was a source.	GHG
Abhijith et al. (2017)	This review highlights how certain GI designs can unintentionally increase pollutant concentrations by disrupting airflow in street canyons.	Airflow disruption
Sierra-Heredia et al. (2018)	The study explores the distribution and health risks of Canadian aeroallergens, while assessing how climate dynamics, air pollution, and urban vegetation changes contribute.	Pollen and allergenicity
Eisenman et al. (2019)	This study shows that urban tree planting programs often fail to adequately consider the allergenicity of	Pollen and allergenicity

	selected species.	
Sawers (2019)	Policy-oriented review highlighting the importance of species selection to minimize bVOC contributions to air pollution.	bVOC
Barwise and Kumar (2020)	This review emphasizes how poor selection of plant species for vegetative barriers may fail to reduce or even exacerbate air pollution levels.	Airflow disruption, pollen and allergenicity, GHG, bVOC
Salvador et al. (2020)	Field research in a subtropical forest reveals that bVOC oxidation products play a major role in ozone and secondary organic aerosol (SOA) formation.	bVOC
Gu et al. (2021)	Urban greening within LA may raise bVOCs, offsetting air quality gains. Using low-VOC plants is advised.	bVOC
Sousa-Silva et al. (2021)	This study finds that an insufficient understanding of tree pollen allergenicity can lead to planting choices that unintentionally increase health risks.	Pollen and allergenicity
Browning et al. (2022)	This study shows the positive health outcomes from GBGI exposure but noted that allergenic pollen release represents a significant challenge that can diminish these benefits for portions of the population.	Pollen and allergenicity
Cao et al. (2022)	Long-term modelling in China indicating biomass-driven increases in bVOC emissions intensify summertime O_3 and SOA.	bVOC
Fellini et al. (2022)	Wind tunnel tests show that dense tree canopies may reduce air ventilation, unintentionally trapping pollutants at pedestrian level.	Airflow disruption
Zhang and Steiner, (2022)	The study provides evidence that climate change will alter both the timing and volume of airborne pollen emissions by century's end.	GHG, Pollen and allergenicity
Stawoska et al. (2023)	The study found that birch pollen from polluted areas had higher Bet v1 allergen levels and altered protein structures, potentially increasing allergenicity, despite minimal effects on plant physiology.	Pollen and allergenicity
Li et al. (2024a)	This bibliometric review reveals that constructed wetlands, if poorly designed, may act as unintended sources of methane and carbon dioxide.	GHG
Maison et al. (2024)	Street-level models in Paris show higher pollution and exposure than regional models, especially for NO_2 and BC.	Airflow disruption
Yuan et al. (2025)	This review shows how plants in wetlands influence greenhouse gas emissions, depending on species, diversity, and harvesting.	GHG

Table S5. Summary of key papers addressing the synergistic relationship between GBI and urban ventilation from 2010-2025. The table outlines the effect GBI and urban ventilation (UV) has on urban heat island (UHI), air quality (AQ), building energy performance (BEP), humidity (H), natural ventilation (NV), HVAC systems and thermal comfort (TC).

**Author (Year)**	**Description**	**UV**	**GI**	**AQ**	**HVAC**	**UHI**	**GBI**	**NV**	**H**	**BEP**	**TC**
Amorim et al. (2013)	Evaluation of the impact GI has on pollution dispersal emitted by road traffic in an urban setting using numerical models.	√	√	√							
Asere and Blumberga (2020)	Analysis of how indoor air quality, building efficiency, and HVAC energy use are interrelated.				√					√	
Asim et al. (2022)	Review of the parameters that inform the sustainable performance of HVAC systems.			√						√	√
Badach et al. (2022)	GIS and numerical modeling used to assess how structural changes influence ventilation pathways.	√		√			√				
Beier et al. (2022)	Microclimate modelling of the influence GBI has on UHI.					√	√				
Belda et al. (2021)	Numerical model examining real world urban case study, and the influence GI has upon particulate concentrations, surface and air temperatures.	√	√	√		√					
Buccolieri et al. (2010)	Modelling urban ventilation to test the 'breathability' of cities.	√									
Considine et al. (2022)	Development of rooftop air filtration technology to reduce particulate load under diverse climates.	√		√	√						
Considine et al. (2023)	Street canyon model analysing the concentration rate of vehicular particulate emissions entering the outdoor fresh air intake of a HVAC system located at rooftop level.	√									
Irga et al. (2024)	Integrating green walls into tunnel ventilation stacks to reduce the potential increase in ambient particulate emissions.	√	√	√							
Kostadinović et al. (2023)	On-site study of green roof performance in passive air pollutant control.	√									
Liu et al. (2021)	Review of green roof effects on hydrology, thermal regulation, and air quality.		√	√		√					

Morgan et al. (2017)	Deployment of passive air filtration to reduce ambient particulate matter entering ventilation systems.		√		√						
Palusci & Cecere, (2022)	Developed a workflow and framework for improving urban ventilation in existing urban areas to mitigate UHI and air pollution.	√									
Park et al. (2024)	Analysis of GI as a mitigation measure for UHI in a humid region.	√	√			√			√		
Ramasubramanian et al. (2019)	Field study investigating the reduction of O_3 present in the outdoor fresh air ventilation intake at the rooftop level where a green roof is present.	√				√					
Ramasubramanian et al. (2021)	Field study comparing the concentrations of bVOC, microbial composition and O_3 present on the filters of a HVAC system for a green and white roof.		√	√	√						
Salmond et al. (2013)	Field study of the influence GI has on the vertical and horizontal gradients of pollutants in a street canyon.			√	√						
Shi et al. (2022)	Investigated how ventilation corridors and GBI influence UHI across diverse urban climates.	√				√	√				
Tang et al. (2023)	Developed theoretical references for urban and GBI planning based on wind-driven ventilation and pollutant dispersion in street canyons.	√									
Vasudevan et al. (2024)	Adjustable wind deflectors used to promote pollution removal from urban street canyons.	√		√							
Wahba et al. (2018)	Retrofitting GI to a building's roof and walls to enhance the HVAC system's energy performance.		√	√	√					√	
Xiong et al. (2024)	Proposed a natural ventilation model for energy-efficient buildings, integrating multiple environmental variables to assess thermal comfort and indoor air quality.			√				√		√	
Zhang et al. (2020)	GHG emissions associated with air condition systems at a city scale.	√		√	√					√	
Zhang et al. (2023b)	Parametric investigation into how GI planted upstream of a building impacts the natural cross ventilation rates.				√			√			
Zhao et al. (2024)	Machine learning model to predict local temperatures and wind velocities with the addition of new buildings and HVAC heat rejection in high density urban areas.	√									

Table S6. Potential conflicts to achieving synergistic control of urban challenges through GBI.

GBI types	Potential disservices	Case studies and meta-analysis reviews
Large BI (e.g., lakes and rivers)	Nighttime UHI intensification (e.g., due to increased humidity and heat release from water masses)	(Chen et al., 2024; Fricke et al., 2024; Hu and Li, 2020; Theeuwes et al., 2013; Triyuly et al., 2021)
	Greenhouse gas emissions from sediment accumulation (e.g., N_2O and CH_4 emissions)	(Woszczyk and Schubert, 2021; Yin et al., 2025; Zhang et al., 2021)
Small BI (e.g., ponds and ditches)	Nighttime UHI intensification (e.g., due to increased humidity and heat release from water sources)	(Yao et al., 2023; Fricke et al., 2024)
	Greenhouse gas emissions from sediment accumulation (e.g., CH_4 and N_2O emissions)	(Bauduin et al., 2024; Holgerson and Raymond, 2016; Rosentreter et al., 2021; van Bergen et al., 2019)
Large GI (e.g., Parks)	Biogenic emissions (bVOCs and pollen from vegetation, contributing to elevated O_3 and PM concentrations)	(Ahn et al., 2022; Bao et al., 2023; Churkina et al., 2017; Ma et al., 2022; Maison et al., 2024)
Street trees and hedges	Obstruction of pollutant dispersion (e.g., dense tree canopies reducing street-level ventilation in urban canyons)	(Buccolieri et al., 2018; Li et al., 2023; Salmond et al., 2013; Gromke and Ruck, 2007; Guo et al., 2023; Řezníček et al., 2025)
All types of GI	Over-Irrigation Leading to Water Scarcity (e.g., excessive water consumption by turfgrass during drought seasons)	(Cheung et al., 2022; Litvak et al., 2017; Pincetl et al., 2019; Saher and Ott, 2025; Van Mechelen et al., 2015; Wilfong et al., 2025)

Table S7. Overview of data collection methods (measurements, questionnaires, and simulations) and objectives pursued by GBI implementation (cooling/heating effect assessment and thermal comfort evaluation).

Author (Year)	Data collection method					Objective	
	Measurements			Subjective questionnaires	Simulation	Cooling/ heating effect	Thermal comfort
	Environmental parameters	Physiological parameters	Remote sensing				
Dashti et al. (2024)	√			√		√	√
Fei et al. (2023a)	√			√		√	√
Fei et al. (2024)	√				√	√	
Li et al. (2020)	√				√	√	√
Pritipadmaja et al. (2023)			√			√	
Manavvi and Milosevic (2025)	√					√	√
Jiang et al. (2020)					√	√	
Tan et al. (2021)			√			√	
Fei et al. (2022)	√			√	√	√	√
Ming et al. (2025)	√				√	√	
Cao et al. (2022)	√				√	√	
Behzad & Guilandoust, (2024)					√	√	√
Islam et al. (2024)			√			√	
Yang et al. (2020)			√			√	
Fei et al. (2023b)	√			√		√	√
Sun et al. (2020)			√			√	
Sun et al. (2021)					√	√	√
Wang et al. (2024)	√			√		√	√
Huang et al. (2024b)			√			√	
Balany et al. (2022)			√			√	
Lehnert et al. (2021)	√				√	√	√
Huang et al. (2024a)	√	√		√		√	√

Table S8. Summary of vegetation responses to climate change in cities. The table presents an overview of four main responses of plants to stress and how they take place in cities according to recent case-studies and reviews.

Responses to climate change	Reported pathways	Author (Year)
Phenotypic plasticity	● Phenotypic plasticity confers resilience to plant species growing in cities.<br>● Possible plasticity to enhance the knowledge of tree sensitivity and adaptive capacity to climate change.<br>● Leaf-level characteristics exhibited higher variability across different species and urban locations.<br>● Heat and drought tolerant species adjust assimilation rate to maintain growth rate.<br>● Air pollution modulates morphological and physiological traits, affecting photosynthesis differently depending on the plant species.<br>● Artificial light at night negatively impacts the photosynthetic parameters and the pigment content.	Das et al. (2024); Esperon-Rodriguez et al. (2020); Géron et al. (2024); Hanley et al. (2021); Locosselli et al. (2024); Martínez-Villa et al. (2024); Matsumoto et al. (2022); Papadopoulou et al. (2023); Sotillo et al. (2024); Wang et al. (2023); Wei et al. (2023)
Adaptation	● Urban environments may impose selective pressures that drive adaptation in short-lived plant species.<br>● Phenotypic plasticity may enhance the adaptive potential of specific plant type in cities.<br>● Genetic differentiation among urban and rural populations of the same species indicates adaptive responses to urban selective pressures.<br>● Phenotypic plasticity can increase the adaptive capacity of plant species in urban settings.	de Barros Ruas et al. (2022); Fukano et al. (2020); Fukano et al. (2023a); Fukano et al. (2023b), Lambert et al. (2021); Silva Luz et al. (2024); Sotillo et al. (2024); Woudstra et al. (2024)
Assisted migration	● Changes in environmental conditions due to urbanization processes support the use of natural features from nearby ecosystems to enhance the resilience of GBI in urban areas.	Miyahara et al. (2022)
Extinction	● Environmental changes due to urbanization processes can make it difficult to native species survive and reproduce, leading to local extinctions.<br>● GBI management, eutrophication, aridity, and landscape fragmentation are key local drivers of extinction for species with low phenotypic plasticity.	Hahs et al. (2009); Sotillo et al. (2024)

Table S9. Summary of key environmental injustice and GBI decision making challenges and research findings North and South Global countries. The table provides an overview of selected studies that focus on green gentrification and racial inequalities in the implementation of GBI

Place studied	Key focus	What was covered	Author (Year)
Australia	Relationship between the development of urban green areas, the price increase, and the formation of gentrification.	Housing mobility and gentrification dynamics.	Caprioli et al. (2023)
Brazil–Curitiba and São Paulo	Remote sensing is used to examine the temperature variation, identifying heat islands.	Green infrastructures and neighbourhood income level.	Ribeiro et al. (2023)
China	Employs an array of social-environmental benefits to evaluate GI's contributions to human well-being.	Mitigation of the UHI effect, recreational functions, enhanced landscape connectivity, and efficient stormwater management. Well-being and socio-economic indexes.	Xiong et al. (2024)
Colombia	Colombian GBI implementation experience	Recognize the significant influence of grassroots activism and civil society in defending urban commons, and recommend a critical review of green-blue infrastructure and river rehabilitation projects in Latin America.	Pradilla & Hack (2024)
Europe (3 cities) and the US (4 cities)	Explores the role that green gentrification plays in exacerbating racial tensions within historically marginalized urban communities, benefiting via enhancements like parks, gardens, waterfront restoration, and urban greenways.	Examines the complex relationship between historical environmental and racial injustices and current racial green inequities produced by the green city agenda.	Lewartowska (2024)
Italy–Rome and Poland–Gdansk	NbS can enhance air quality and improve societal well-being, but it is often not implemented equally, resulting in environmental injustice patterns.	The study supports concerns for fair NbS distribution, pointing to the potential exacerbation of socioeconomic divides, which heighten the exposure of disadvantaged communities to climate impacts.	Azadgar et al. (2025)
Portugal–Porto	Explores green infrastructure and its effects on urban heat island in Porto, Portugal, by monitoring the distribution of air spatial temperature and humidity.	Evaluation of vulnerability to heat-related health risks using heat risk index, integrating land surface temperature, land cover, and demographic data through remote sensing.	Lopes et al. (2025)
US	Conducted coding and analysis of 122 GBI formal	Intersectional urban challenges of social injustice and inequity,	Grabowski et al.

	plans from 20 US cities to explore how equity and justice are addressed within the areas of visions, processes, and distributions.	climate change, aging and expensive infrastructure, and socio-economic change.	(2023)
US–Boston	The study of green climate-resilient infrastructure–driven planning highlights a nuanced experience of gentrification and displacement affecting community belonging.	Underrecognized placemaking practices and alternative approaches to tackling socio-climate vulnerability both shape the dynamics of climate justice and injustice.	Shokry et al. (2025)
US–New York	Climate-driven hazards considering disproportionately affect low-income communities and communities of colour.	Co-production between government stakeholders and researchers to evaluate future situations over NYC for 2100 in response to climate hazards.	Dutta et al. (2025)

Table S10. Summary of key cultural challenges and research insights in GBI. This table presents a synthesis of cultural challenges identified in the planning, implementation, and adoption of GBI, drawn from diverse case studies and literature reviews. It highlights key findings from the selected studies along with actionable recommendations.

Cultural challenge	Case study/review	Key findings	Recommendations	Author (Year)
Under-recognition of cultural benefits	Review of 14,344 papers on CES using CiteSpace	CES underrepresented vs. ecological functions; limited focus on heritage, identity, education	Integrate CES via clear frameworks and wider spatial scales	Li et al. (2024b)
	Review of 153 interdisciplinary studies	Biophysical goals dominate; cultural identity and heritage overlooked	Use interdisciplinary and Indigenous-informed planning	McNabb et al. (2024a)
	Gorky Park, Russia	CES present but heritage and education uses underrepresented	Add cultural programming, educational and heritage-sensitive elements	Dushkova et al. (2025)
	Review (U.S, China, Canada)	Non-monetary values like place and learning are often missed	Incorporate CES such as sense of place and education into GBI	Elmqvist et al. (2015)
Limited community and cultural engagement	Semarang, Indonesia	Top-down BGI ignores local culture; weak awareness and uptake	Apply inclusive, bottom-up planning	Drosou et al. (2019)
	Wigram Basin, New Zealand	Weak perception of socio-cultural value due to limited engagement.	Include cultural experts, use co-design, plan explicitly for cultural value.	McNabb et al. (2024b)
	Helsinki, Finland	Technocratic planning sparked opposition due to overlooked values	Use early value mapping to include local cultural dimensions	Kati & Jari (2016)
	Narrative review (U.S. GI practices)	Lack of genuine participation excludes underrepresented groups and reinforces power imbalances	Start with participatory planning to empower communities and reflect local cultural values	Lovell & Taylor (2013)
	Conceptual review and framework proposal	Existing BGI frameworks overlook community values, practices, and engagement	Create tailored social resilience frameworks with place-based values and co-designed indicators	Campbell et al. (2024)
Lack of	Tumbes Basin, Peru	Indigenous agro-ecological GBI ignored in	Decolonize GI via Indigenous systems and	Tomateo (2021)

integration of Indigenous knowledge		formal planning	flexible typologies	
Cultural heritage and traditional landscapes	Jiangnan Furniture Museum, China	Optimized courtyard design improves microclimate and reduces seasonal energy loads	Combine vernacular spatial logic with passive strategies for cultural and environmental performance	Xu et al. (2018)
	Suzhou gardens (China)	Traditional gardens regulate climate and preserve identity	Apply water–vegetation–architecture logic in modern parks	Jiadai & Ibrahim, (2025)
	Antwerp, Belgium	Vertical greening (VG) applied to protected buildings raises concerns over material damage and lack of guidance	Provide context-specific strategies and heritage- compatible VG guidelines	Kale et al. (2023)
	Roman Theatre of Piane di Falerone, Italy	Removing vegetation solely based on preservation risks can worsen thermal comfort and reduce site usability	Balance vegetation removal with thermal comfort using tools like the Vegetation Hazard Impact Index (VHI) and microclimate modelling	Fabbri et al. (2025)
	Malaysian heritage cities (e.g., Sekeping, Penang & Kuala Lumpur)	Green reuse supports identity and performance, but GBI lacks heritage-specific criteria	Create a “Green Adaptive Reuse Index” linking GBI with heritage conservation	Alauddin et al. (2022)
‘Green gentrification’ and cultural displacement	Washington, DC	Long-term residents see GI as tied to displacement	Early community engagement, listening sessions, trust-building	Gearin et al. (2023)
	Six-city comparison (Barcelona, Halle, Lodz, New York, Oslo, Stockholm)	Overlooking justice leads to displacement, especially among marginalized groups	Apply EJ filters and participatory processes to address institutional and perceptual barriers	Kronenberg et al. (2021)
Culturally specific landscape preferences	Taj Mahal (India) & Ryoan-ji Garden (Japan)	Religious beliefs shape distinct spatial aesthetics rooted in Islamic and Zen traditions	GBI design should respect cultural interpretations of nature and avoid universalized models	Minnema (2019)
	Berlin, Germany	Green space meets quantity but not immigrant/elderly needs	Design based on culturally diverse usage and programming	Kabisch and Haase (2014)

129 **Table S11.** Summary of security barriers and solutions in GBI implementation across scales.

Scale	Safety/Security Concern	Description	Mitigation Strategy	Author (Year)
Street-level	Surveillance Blind Spots	Vegetated areas obstruct visibility, enabling illicit activities	Design with sightlines, low vegetation, smart lighting and cameras	Marzbali et al. (2012); He et al. (2025)
Household-level	Entry Point Obstruction	Vegetated fences/hedges obscure view of entry points	Transparent fencing, motion lighting, neighbourhood watch	Piroozfar et al. (2019); Marzbali et al. (2012)
Pocket Parks	Unregulated Gathering Spaces	Small parks may attract loitering or anti-social behaviour	Open layouts, visibility from homes, community programming	Kabisch et al. (2015); Nordh et al. (2009)
Informal Areas	Crime-Associated Stigma	GBI avoided for fear it may harbour crime in underserved areas	Participatory design, community policing	Pauleit et al. (2019); Kondo et al. (2016)
Transit Greens	Safety during Low-Use Periods	Isolated greenways/transit paths increase night-time vulnerability	Active lighting, regular patrols, clear escape routes	Bennetts et al. (2017)
All Scales	Over-Securitization	Excessive monitoring limits accessibility and inclusion	Balanced governance, inclusive and transparent planning	Wolch et al. (2014)

130

Table S12. Design trade-offs and integration strategies between climate adaptation and aesthetics in GBI.

Design Element	Climate Adaptation Function	Aesthetic Preference	Design Challenge	Creative Integration Strategy	Author (Year)
**Vegetation Type**	Native and drought-tolerant species enhance resilience and biodiversity	Ornamental and manicured plants are often preferred visually	Native species are perceived as messy or unmanaged	Combine native plants with structured layouts, signage, and seasonal color	Gillner et al. (2015); Lindemann-Matthies and Brieger, (2016); Livesley et al. (2016); Sjöman et al. (2012); Talal et al. (2021)
**Spatial Structure**	Heterogeneous layered landscapes buffer flood and reduce heat	Open, symmetrical spaces perceived as tidy and safe	Complex or irregular layout may be viewed as chaotic or unsafe	Frame ecological complexity with structured elements like paths or borders	Gillner et al. (2015); Ignatieva et al. (2017); Livesley et al. (2016)
**Water Feature**	Bioswales, retention ponds, and wetlands absorb runoff and improve water quality	Decorative fountains and still water bodies are preferred	Functional water features may appear unattractive or muddy	Integrate stormwater infrastructure with artistic and accessible design	Li and Nassauer, (2020); Meerow & Newell, (2017)
**Maintenance Approach**	Low-input systems mimic natural processes and reduce costs	High-maintenance landscapes signal care and order	Reduced upkeep can lead to perceptions of neglect	Promote "messy but cared for" aesthetic with signs and community involvement	Haase et al. (2014); Ignatieva et al. (2017); Li and Nassauer, (2020)
**Public Perception**	Resilient designing enhances long-term ecological health and equity	Residents often favour short-term beauty or tradition	Lack of awareness can reduce support for adaptive landscapes	Engage communities in co-designing, educating, and storytelling	Frantzeskaki et al. (2019); Hoyle et al. (2017); Raymond et al. (2017)
**Planning and Policy**	Integrated planning aligns spatial design with climate goals	Beautifying and greening goals are often fragmented	Ecological functions remain underfunded or undervalued	Support cross-sector collaboration and multifunctional policy tools	Kabisch et al. (2016); Langemeyer and Baró, (2021)

Table S13. Mapping of the twelve recommendations in Section 8 to the 21 identified barriers across environmental, social, economic, and governance domains. Each recommendation is linked to the specific barrier(s) and manuscript section(s) it addresses, enhancing clarity and strengthening the connection between challenges and proposed solutions.

**Recommendation (as in manuscript)**	**Barrier(s) addressed**	**Section(s)**
1. Strengthen interdisciplinary collaboration	Siloisation; Conflict control among urban challenges	3.2; 3.4
2. Prioritise science-driven species selection and adaptive design	Unintended consequences; Plant adaptation challenges; Biodiversity undervaluation	3.3; 3.7
3. Harness microclimate modelling and AI for ventilation-sensitive design	Urban ventilation; Trade-offs in microclimate and air quality	3.5; 3.8
4. Integrate thermal adaptation and vulnerability information	Thermal resilience; Environmental justice	3.6; 4.1
5. Integrate social dimensions into research frameworks	Cultural disconnection; Adoption challenges; Aesthetic controversies	4.2; 4.3; 4.5
6. Promote cross-disciplinary collaboration with communities	Social adoption; Safety concerns	4.3; 4.4
7. Develop context-sensitive policies	Safety concerns; Aesthetic controversies; Land and& space constraints	4.4; 4.5; 6.1
8. Strengthen financial ecosystem with ESG metrics	Economic undervaluation; Cost–benefit limitations	5.1; 5.2
9. Scale innovative financing mechanisms	Investment barriers; Regulatory gaps	5.3; 6.4
10. Align financial regulation and incentives	Integration challenges with other urban systems; Economic undervaluation; Investment barriers	5.1; 5.2; 5.3
11. Prioritise micro-scale, 3D GBI	Land and& space constraints; Trade-offs in land use	6.1
12. Embed GBI in urban planning through adaptive governance	Policy fragmentation; Integration challenges; Regulatory gaps	6.2; 6.3; 6.4

References

Abdallah A. K., Ismail L. S. and Alkaabi A. M. (2025). Green Careers: Educating for the Future of Sustainability. In Legal Frameworks and Educational Strategies for Sustainable Development, (IGI Global), 337-366.

Abhijith, K. V., Kumar, P., Gallagher, J., McNabola, A., Baldauf, R., Pilla, F., Broderick, B., Di Sabatino, S., & Pulvirenti, B. (2017). Air pollution abatement performances of green infrastructure in open road and built-up street canyon environments–A review. Atmospheric Environment, 162, 71–86.

Ahn, J.-W., Dinh, T.-V., Park, S.-Y., Choi, I.-Y., Park, C.-R., & Son, Y.-S. (2022). Characteristics of biogenic volatile organic compounds emitted from major species of street trees and urban forests. Atmospheric Pollution Research, 13(7), 101470.

AIPH (2025). Green City Case Study: Melbourne, Australia: Grey to Green. International Association of Horticultural Producers. Available online: https://aiph.org/green-city-case-studies/melbourne-australia/ (Accessed on August 28, 2025).

Alauddin, K., Mohd Nusa, F. N., Baharuddin, M. N., Abdul Rashid, M. S., & Remeli, R. (2022). The application of green adaptive reuse of historical buildings in UNESCO cities. Planning Malaysia, 20.

Amorim J.H., Rodrigues, V., Tavares, R., Valente, J., & Borrego, C. (2013). CFD modelling of the aerodynamic effect of trees on urban air pollution dispersion. Science of The Total Environment 461-462, 541-551.

Anders, S., Day, T., & Kuduk, C. A. (2010). "Hey, Your Tree Is Shading My Solar Panels": California's Solar Shade Control Act. Journal of Sustainable Real Estate, 2, 361-381.

Asa'a, S., Reher, T., Rongé, J., Diels, J., Poortmans, J., Radhakrishnan, H. S., van der Heide, A., Van de Poel, B., & Daenen, M. (2024). A multidisciplinary view on agrivoltaics: Future of energy and agriculture. Renewable and Sustainable Energy Reviews, 200, 114515.

Asere, L., Blumberga, A. (2020). Does energy efficiency-indoor air quality dilemma have an impact on the gross domestic product? J. Environ. Manage. 262, 110270.

Asim, N., Badiei, M., Mohammad, M., Razali, H., Rajabi, A., Chin Haw, L., & Jameelah Ghazali, M. (2022). Sustainability of Heating, Ventilation and Air-Conditioning (HVAC) Systems in Buildings—An Overview. International Journal of Environmental Research and Public Health, 19, 1016.

Azadgar, A., Luciani, G., & Nyka, L. (2025). Spatial allocation of nature-based solutions in the form of public green infrastructure in relation to the socio-economic district profile–a GIS-based comparative study of Gdańsk and Rome. Land Use Policy, 150.

Badach, J., Szczepański, J., Bonenberg, W., Gębicki, J., & Nyka, L. (2022). Developing the Urban Blue-Green Infrastructure as a Tool for Urban Air Quality Management. Sustainability, 14, 9688.

Balany, F., Muttil, N., Muthukumaran, S., Wong, M. S., & Ng, A. W. (2022). Studying the effect of blue-green infrastructure on microclimate and human thermal comfort in Melbourne's central business district. Sustainability, 14, 9057.

Bao, X., Zhou, W., Xu, L., & Zheng, Z. (2023). A meta-analysis on plant volatile organic compound emissions of different plant species and responses to environmental stress. Environmental Pollution, 318, 120886.

Barwise, Y., & Kumar, P. (2020). Designing vegetation barriers for urban air pollution abatement: A practical review for appropriate plant species selection. Npj Climate and Atmospheric Science, 3, 12.

Bauduin, T., Gypens, N., & Borges, A. V. (2024). Seasonal and spatial variations of greenhouse gas (CO2, CH4 and N2O) emissions from urban ponds in Brussels. Water research, 253, 121257.

Behzad, Z., & Guilandoust, A. (2024). Enhancing outdoor thermal comfort in a historic site in a hot dry climate (Case study: Naghsh-e-Jahan Square, Isfahan). Sustainable Cities and Society, 102, 105209.

Beier, M., Gerstendörfer, J., Mendzigall, K., Pavlik, D., Trute, P., von Tils, R. (2022). Climate Impact and Model Approaches of Blue-Green Infrastructure Measures for Neighborhood Planning. Sustainability 14, 6861.

Belda, M., Resler, J., Geletič, J., Krč, P., Maronga, B., Sühring, M., Kurppa, M., Kanani-Sühring, F., Fuka, V., Eben, K., Benešová, N., Auvinen, M. (2021). Sensitivity analysis of the PALM model system 6.0 in the urban environment. Geosci. Model Dev. 14, 4443–4464.

Bennetts, H., Soebarto, V., Oakley, S., & Babie, P. (2017). Feeling safe and comfortable in the urban environment. Journal of Urbanism: International Research on Placemaking and Urban Sustainability, 10, 401-421.

Benz S. A., & Burney, J. A. (2021). Widespread Race and Class Disparities in Surface Urban Heat Extremes Across the United States. Earth's Future 9:1–14.

Bressane A., Loureiro A. I. S., Medeiros L. C. d. C., et al. (2024). Overcoming Barriers to Managing Urban Green Spaces in Metropolitan Areas: Prospects from a Case Study in an Emerging Economy. Sustainability 16:7019

Browning, M. H., Rigolon, A., & McAnirlin, O. (2022). Where greenspace matters most: A systematic review of urbanicity, greenspace, and physical health. Landscape and Urban Planning, 217, 104233.

Buccolieri, R., Jeanjean, A. P., Gatto, E., & Leigh, R. J. (2018). The impact of trees on street ventilation, NOx and PM2. 5 concentrations across heights in Marylebone Rd street canyon, central London. Sustainable Cities and Society, 41, 227-241.

Buccolieri, R., Sandberg, M., & Di Sabatino, S. (2010). City breathability and its link to pollutant concentration distribution within urban-like geometries. Atmospheric Environment, 44, 1894-1903.

Calfapietra, C., Fares, S., Manes, F., Morani, A., Sgrigna, G., & Loreto, F. (2013). Role of Biogenic Volatile Organic Compounds (BVOC) emitted by urban trees on ozone concentration in cities: A review. Environmental pollution, 183, 71-80.

Campbell, A., Chanse, V., & Schindler, M. (2024). Developing a conceptual framework for characterizing and measuring social resilience in blue-green infrastructure (BGI). Sustainability, 16, 3847.

Cao, S., Wang, Y., Ni, Z., & Xia, B. (2022). Effects of blue-green infrastructures on the microclimate in an urban residential area under hot weather. Frontiers in Sustainable Cities, 4, 824779.

Caprioli, C., Bottero, M., & De Angelis, E. (2023). Combining an agent-based model, hedonic pricing and multicriteria analysis to model green gentrification dynamics. Computers, Environment and Urban Systems, 102.

Cariñanos, P., & Casares-Porcel, M. (2011). Urban Choigreen zones and related pollen allergy: A review. Some guidelines for designing spaces with low allergy impact. Landscape and urban planning, 101, 205-214.

Chen, L., Liu, C., Zou, R., Yang, M., & Zhang, Z. (2016). Experimental examination of effectiveness of vegetation as bio-filter of particulate matters in the urban environment. Environmental Pollution, 208, 198-208.

Chen, X., Wang, H., & Yang, J. (2024). Effect of green blue spaces on the urban thermal environment: A field study in Hong Kong. Urban Climate, 55, 101912.

Cheung, P. K., Nice, K. A., & Livesley, S. J. (2022). Irrigating urban green space for cooling benefits: The mechanisms and management considerations. Environmental Research: Climate, 1(1), 015001.

Churkina, G., Kuik, F., Bonn, B., Lauer, A., Grote, R., Tomiak, K., & Butler, T. M. (2017). Effect of VOC Emissions from Vegetation on Air Quality in Berlin during a Heatwave. Environmental Science & Technology, 51(11), 6120–6130.

Climate-ADAPT (2022). Paris Oasis Schoolyard Programme, France. European Environment

Cohen-Shacham, E., Walters, G., Janzen, C., & Maginnis, S. (2016). Nature-based solutions to address global societal challenges. IUCN: Gland, Switzerland, 97(2016), 2036.

Considine, B., Gallagher, J., Kumar, P., & McNabola, A. (2023). The impact of street level particulate emissions on the energy performance of roof level building ventilation systems. Journal of Wind Engineering and Industrial Aerodynamics, 233, 105310.

Considine, B., McNabola, A., Kumar, P., & Gallagher, J. (2022). A numerical analysis of particulate matter control technology integrated with HVAC system inlet design and implications on energy consumption. Building and Environment, 211, 108726.

Das, S., Ossola, A., & Beaumont, L. J. (2024). Records of urban occurrences expand estimates of the climate niches in tree species. Global Ecology and Biogeography, 33, e13809.

Dashti, A., Mohammadsharifi, N., Shokuhi, M., & Matzarakis, A. (2024). A comprehensive study on wintertime outdoor thermal comfort of blue-green infrastructure in an arid climate: A case of Isfahan, Iran. Sustainable Cities and Society, 113, 105658.

de Barros Ruas, R., Costa, L. M. S., & Bered, F. (2022). Urbanization driving changes in plant species and communities–A global view. Global Ecology and Conservation, 38, e02243.

Dhar, A., Naeth, M.A., Jennings, P.D., Gamal El-Din, M. (2020). Perspectives on environmental impacts and a land reclamation strategy for solar and wind energy systems. Science of The Total Environment, 718, 134602.

Drosou, N., Soetanto, R., Hermawan, F., Chmutina, K., Bosher, L., & Hatmoko, J. U. D. (2019). Key Factors Influencing Wider Adoption of Blue–Green Infrastructure in Developing Cities. Water, 11, 1234.

Dunlap, A.A., Sovacool, B.K., & Novakovic, B. (2024). "Our town is dying:" Exploring utility-scale and rooftop solar energy injustices in Southeastern California. Geoforum 156, 104120.

Dushkova, D., Taherkhani, M., Konstantinova, A., Vasenev, V. I., & Dovletyarova, E. A. (2025). Understanding Factors Affecting the Use of Urban Parks Through the Lens of Ecosystem Services and Blue–Green Infrastructure: The Case of Gorky Park, Moscow, Russia. Land, 14, 237.

Dutta, M., Herreros-Cantis, P., McPhearson, T., Mustafa, A., Palmer, M. I., Tosca, M., Ventrella, J., & Cook, E. M. (2025). New York City 2100: Environmental justice implications of future scenarios for addressing extreme heat. Landscape and Urban Planning, 254.

Eisenman, T. S., Jariwala, S. P., & Lovasi, G. S. (2019). Urban trees and asthma: a call for epidemiological research. The Lancet Respiratory Medicine, 7, e19-e20.

Elmqvist, T., Setälä, H., Handel, S., Van Der Ploeg, S., Aronson, J., Blignaut, J., Gómez-Baggethun, E., Nowak, D., Kronenberg, J., & De Groot, R. (2015). Benefits of restoring ecosystem services in urban areas. Current Opinion in Environmental Sustainability, 14, 101–108.

Esperon-Rodriguez, M., Rymer, P. D., Power, S. A., Challis, A., Marchin, R. M., & Tjoelker, M. G. (2020). Functional adaptations and trait plasticity of urban trees along a climatic gradient. Urban Forestry & Urban Greening, 54, 126771.

Essak, L., & Ghosh, A. (2022). Floating photovoltaics: A review. Clean Technologies, 4, 752-769.

Fabbri, K., Catalano, M., & Ugolini, A. (2025). Vegetation in Archaeological Areas: Risks, Opportunities, and Guidelines to Preserve or Remove: An Italian Case Study. Sustainability, 17, 2712.

Fei, F., Wang, L., Wang, Y., Yao, W., Fukuda, H., Xiao, Y., Tian, L. & Ji, T. (2023a). A new method for evaluating the synergistic effect of urban water body and vegetation in the summer outdoor thermal environment. Journal of Cleaner Production, 414, 137680.

Fei, F., Wang, Y., Wang, L., Fukuda, H., Yao, W., Zhou, Y. & Dong, X. (2023b). Mechanisms of urban blue-green infrastructure on winter microclimate using artificial neural network. Energy and Buildings, 293, 113188.

Fei, F., Wang, Y., Yao, W., Gao, W. & Wang, L. (2022). Coupling mechanism of water and greenery on summer thermal environment of waterfront space in China's cold regions. Building and Environment, 214, 108912.

Fei, F., Xiao, Y., Wang, L., Wang, Y., Fukuda, H., Yao, W., Yu, H., & Dong, Q. (2024). A novel approach for quantifying the influence intensity of urban water and greenery resources on microclimate for efficient utilization. Sustainable Cities and Society, 112, 105597.

Fellini, S., Marro, M., Del Ponte, A. V., Barulli, M., Soulhac, L., Ridolfi, L., & Salizzoni, P. (2022). High resolution wind-tunnel investigation about the effect of street trees on pollutant concentration and street canyon ventilation. Building and Environment, 226, 109763.

Flammer C. (2020). Green bonds: effectiveness and implications for public policy. Environmental and Energy Policy and the Economy 1:95-128.

Flammer C. (2021). Corporate green bonds. Journal of Financial Economics 142:499-516.

Frantzeskaki, N., McPhearson, T., Collier, M. J., Kendal, D., Bulkeley, H., Dumitru, A., Walsh, C., Noble, K., Van Wyk, E., Ordóñez, C., Oke, C., & Pintér, L. (2019). Nature-based solutions for urban climate change adaptation: linking science, policy, and practice communities for evidence-based decision-making. BioScience, 69, 455-466.

Fricke, L., Legg, R., & Kabisch, N. (2024). Impact of blue spaces on urban microclimate in different climate zones, daytimes and seasons–a systematic review. Urban Forestry & Urban Greening, 128528.

Fukano, Y., Guo, W., Uchida, K., & Tachiki, Y. (2020). Contemporary adaptive divergence of plant competitive traits in urban and rural populations and its implication for weed management. Journal of Ecology, 108, 2521-2530.

Fukano, Y., Uchida, K., & Tachiki, Y. (2023a). Urban-rural gradients: how landscape changes drive adaptive evolution of plant competitive traits. Evolutionary Ecology, 37, 215-232.

Fukano, Y., Yamori, W., Misu, H., Sato, M. P., Shirasawa, K., Tachiki, Y., & Uchida, K. (2023b). From green to red: Urban heat stress drives leaf color evolution. Science Advances, 9, 42.

Gearin, E., Dunson, K., & Hampton, M. (2023). Greened out: mitigating the impacts of eco-gentrification through community dialogue. Architecture_MPS, 25, 1-14.

Géron, C., Lembrechts, J. J., Fameree, M., Taddei, V., Nijs, I., & Monty, A. (2024). Phenotypic plasticity as the main driver of alien plant trait variation in urban versus rural microclimate for the model species Veronica persica. Oecologia, 205, 643-654.

Gibbons J. (2023). Examining the long-term influence of New Deal era redlining on contemporary gentrification. Urban Studies 60:2816-2834.

Gillner, S., Vogt, J., Tharang, A., Dettmann, S., & Roloff, A. (2015). Role of street trees in mitigating effects of heat and drought at highly sealed urban sites. Landscape and Urban Planning, 143, 33-42.

Grabowski, Z. J., McPhearson, T., & Pickett, S. T. A. (2023). Transforming US urban green infrastructure planning to address equity. Landscape and Urban Planning, 229. https://doi.org/10.1016/j.landurbplan.2022.104591

Gromke, C., & Ruck, B. (2007). Influence of trees on the dispersion of pollutants in an urban street canyon experimental investigation of the flow and concentration field. Atmospheric Environment, 41, 3287-3302.

Grossi, F., Ge, H., Zmeureanu, R., & Baba, F. (2023). Feasibility of planting trees around buildings as a nature-based solution of carbon sequestration An LCA approach using two case studies. Buildings, 13, 41.

Gu, S., Guenther, A., & Faiola, C. (2021). Effects of anthropogenic and biogenic volatile organic compounds on Los Angeles air quality. Environmental Science & Technology, 55, 12191-12201.

Guo, Y., Xiao, Q., Ling, C., Teng, M., Wang, P., Xiao, Z., Wu, C. (2023). The right tree for the right street canyons: An approach of tree species selection for mitigating air pollution. Building and Environment 245, 110886.
Haase, D., Larondelle, N., Andersson, E., Artmann, M., Borgström, S., Breuste, J., Gomez-Baggethun, E., Gren, A., Hamstead, Z., Hansen, R., Kabisch, N., Kremer, P., Langemeyer, J., Lorance Rall, E., McPhearson, T., Pauleit, S., Qureshi, S., Schwarz, N., Voigt, A., Wurster D., & Elmqvist. T. (2014). A quantitative review of urban ecosystem service assessments: Concepts, models, and implementation. Ambio, 43, 413–433.
Hahs, A. K., McDonnell, M. J., McCarthy, M. A., Vesk, P. A., Corlett, R. T., Norton, B. A., Clemants, S. E., Duncan, R. P., Thompson, K., Schwartz, M. W., & Williams, N. S. (2009). A global synthesis of plant extinction rates in urban areas. Ecology Letters, 12, 1165-1173.
Hanley, P. A., Arndt, S. K., Livesley, S. J., & Szota, C. (2021). Relating the climate envelopes of urban tree species to their drought and thermal tolerance. Science of The Total Environment, 753, 142012.
He, Q., Wu, L., Lee, C. S., Zhu, C., Bai, W., Guo, W., & Ye, X. (2025). Greener the safer? Effects of urban green space on community safety and perception of safety using satellite and street view imagery data. Journal of Criminal Justice, 97, 102372.
Hernandez, R.R., Easter, S.B., Murphy-Mariscal, M.L., Maestre, F.T., Tavassoli, M., Allen, E.B., Barrows, C.W., Belnap, J., Ochoa-Hueso, R., Ravia, S., & Allen, M.F. (2014). Environmental impacts of utility-scale solar energy. Renewable and Sustainable Energy Review, 29, 766–779.
Holgerson, M. A., & Raymond, P. A. (2016). Large contribution to inland water CO2 and CH4 emissions from very small ponds. Nature Geoscience, 9(3), 222–226.
Hoyle, H., Hitchmough, J., & Jorgensen, A. (2017). Attractive, climate-adapted and sustainable? Public perception of non-native planting in the designed urban landscape. Landscape and Urban Planning, 164, 49-63.
Hu, L., & Li, Q. (2020). Greenspace, bluespace, and their interactive influence on urban thermal environments. Environmental Research Letters, 15, 034041.
Huang, B., Zhao, Y., Yang, J., Wang, W., Guo, T., Luo, X., & Du, M. (2024a). Thermal Comfort and Restorative Benefits of Waterfront Green Spaces for College Students in Hot and Humid Regions. Sustainability, 16, 8924.
Huang, S., Xiao, X., Tian, T., & Che, Y. (2024b). Seasonal influences on preferences for urban blue-green spaces: Integrating land surface temperature into the assessment of cultural ecosystem service value. Sustainable Cities and Society, 102, 105237.
Ignatieva, M., Eriksson, F., Eriksson, T., Berg, P., & Hedblom, M. (2017). The lawn as a social and cultural phenomenon in Sweden. Urban Forestry & Urban Greening, 21, 213–223.
Irga, P., Bailes, S., Matheson, S., & Torpy, F. (2024). Incorporation of Green Infrastructure on Road Tunnel Ventilation Stacks: Potential Ambient Air Quality Improvement, Journal of Living Architecture, 10, 1-15.
Islam, M. R., Talukdar, S., Rihan, M., & Rahman, A. (2024). Evaluating cooling effect of blue-green infrastructure on urban thermal environment in a metropolitan city: Using geospatial and machine learning techniques. Sustainable Cities and Society, 113, 105666.
Isola, F., Lai, S., Leone, F., Zoppi, C. (2024). Urban Green Infrastructure and Ecosystem Service Supply: A Study Concerning the Functional Urban Area of Cagliari, Italy. Sustainability, 16(19), 8628.
Janhäll, S. (2015). Review on urban vegetation and particle air pollution – Deposition and dispersion. Atmospheric Environment, 105, 130–137.

Jiadai, T., & Ibrahim, W. Y. B. W. (2025). Visualization microclimate scenarios of Classical Chinese Garden in Suzhou, China. Journal of Infrastructure, Policy and Development, 9(1), 10772.
Jiang, Y., Jiang, S., & Shi, T. (2020). Comparative study on the cooling effects of green space patterns in waterfront build-up blocks: An experience from Shanghai. International journal of environmental research and public health, 17, 8684.
Kabisch, N., & Haase, D. (2014). Green justice or just green? Provision of urban green spaces in Berlin, Germany. Landscape and Urban Planning, 122, 129–139.
Kabisch, N., Qureshi, S., & Haase, D. (2015). Human–environment interactions in urban green spaces A systematic review. Environmental Impact Assessment Review, 50, 25–34.
Kabisch, N., Strohbach, M., Haase, D., & Kronenberg, J. (2016). Urban green space availability in European cities. Ecological Indicators, 70, 586–596.
Kale, E., De Groeve, M., Pinnel, L., Erkan, Y., Hacigüzeller, P., Orr, S. A., & De Kock, T. (2023). Mapping Vertical Greening on Urban Built Heritage Exposed to Environmental Stressors–A Case Study in Antwerp, Belgium. Sustainability, 15, 12987.
Kati, V., & Jari, N. (2016). Bottom-up thinking Identifying socio-cultural values of ecosystem services in local blue–green infrastructure planning in Helsinki, Finland. Land use policy, 50, 537-547.
Kennedy A., Gutterman, S., Pilgrim, B., Ranjan, A., Serre, D., Wason, S., & Climate Risk Task Force (2023). The climate change adaptation gap: An actuarial perspective.
Kondo, M., Hohl, B., Han, S., & Branas, C. (2016). Effects of greening and community reuse of vacant lots on crime. Urban studies, 53, 3279-3295.
Kostadinović, D., Jovanović, M., Bakić, V., Stepanić, N. (2023). Mitigation of urban particulate pollution using lightweight green roof system. Energy Build. 293, 113203.
Kramer J., Silverton S. and Späth P. (2024). Urban governance arrangements for sustainability and justice–linking theory with experience. Urban Transformations 6:6.
Kronenberg, J., Andersson, E., Barton, D. N., Borgström, S. T., Langemeyer, J., Björklund, T., Haase, D., Kennedy, C., Koprowska, K., Łaszkiewicz, E., McPhearson, T., Stange, E. E., & Wolff, M. (2021). The thorny path toward greening: Unintended consequences, trade-offs, and constraints in green and blue infrastructure planning, implementation, and management. Ecology and Society, 26, 36.
Kruitwagen, L., Story, K.T., Friedrich, J., Byers, L., Skillman, S., & Hepburn, C. (2021). A global inventory of photovoltaic solar energy generating units. Nature, 598, 604-611.
Lambert, M. R., Brans, K. I., Des Roches, S., Donihue, C. M., & Diamond, S. E. (2021). Adaptive evolution in cities: progress and misconceptions. Trends in Ecology & Evolution, 36, 239-257.
Langemeyer, J., & Baró, F. (2021). Nature-based solutions as nodes of green-blue infrastructure networks: A cross-scale, co-creation approach. Nature-Based Solutions, 1, 100006.
Larrea, V., Pelaez, F., Esenarro, D. (2024). Design of a Green Corridor and the Revitalization of the Huatanay River, City of Cuzco, Peru 2024. Urban Science, 8, 185 2024.
Lehnert, M., Tokar, V., Jurek, M., & Geletič, J. (2021). Summer thermal comfort in Czech cities: measured effects of blue and green features in city centres. International Journal of Biometeorology, 65, 1277-1289.
Lewartowska, E., Anguelovski, I., Oscilowicz, E., Triguero-Mas, M., Cole, H., Shokry, G., Pérez-del-Pulgar, C., & Connolly, J. J. T. (2024). RACIAL INEQUITY IN GREEN INFRASTRUCTURE AND GENTRIFICATION: Challenging Compounded

Environmental Racisms in the Green City. International Journal of Urban and Regional Research, 48(2), 294–322.
Li Z., Kong L., Hu L., et al. (2024). Greenhouse gas emissions from constructed wetlands: A bibliometric analysis and mini-review. Science of the Total Environment 906:167582.
Li, J., & Nassauer, J. I. (2020). Cues to care: A systematic analytical review. Landscape and Urban Planning, 201, 103821.
Li, J., & Nassauer, J. I. (2021). Technology in support of nature-based solutions requires understanding everyday experiences. Ecology and Society, 26,35.
Li, J., Xu, H., Ren, M., Duan, J., You, W., & Zhou, Y. (2024b). Knowledge Mapping of Cultural Ecosystem Services Applied on Blue-Green Infrastructure A Scientometric Review with CiteSpace. Forests, 15, 1736.
Li, Z., Kong, L., Hu, L., Wei, J., Zhang, X., Guo, W., & Shi, W. (2024a). Greenhouse gas emissions from constructed wetlands: A bibliometric analysis and mini-review. Science of the Total Environment, 906, 167582.
Li, Z., Zhang, H., Juan, Y. H., Lee, Y. T., Wen, C. Y., & Yang, A. S. (2023). Effects of urban tree planting on thermal comfort and air quality in the street canyon in a subtropical climate. Sustainable Cities and Society, 91, 104334.
Lindemann-Matthies, P., & Brieger, H. (2016). Does urban gardening increase aesthetic quality of urban areas? A case study from Germany. Urban forestry & urban greening, 17, 33-41.
Litvak, E., Manago, K. F., Hogue, T. S., & Pataki, D. E. (2017). Evapotranspiration of urban landscapes in Los Angeles, California at the municipal scale. Water Resources Research, 53, 4236-4252.
Liu, H., Kong, F., Yin, H., Middel, A., Zheng, X., Huang, J., Xu, H., Wang, D., Wen, Z. (2021). Impacts of green roofs on water, temperature, and air quality: A bibliometric review. Build. Environ. 196, 107794.
Liu, Y., Skandalos, N., Braslina, L., Kapsalis, V., & Karamanis, D. (2023). Integrating Solar Energy and Nature-Based Solutions for Climate-Neutral Urban Environments. Solar, 3, 382–415
Livesley, S. J., McPherson, E. G., & Calfapietra, C. (2016). The Urban Forest and Ecosystem Services: Impacts on Urban Water, Heat, and Pollution Cycles at the Tree, Street, and City Scale. Journal of Environmental Quality, 45, 119–124.
Locosselli, G. M., Cintra, B. B. L., Ferreira, L. S., Da Silva-Luz, C. L., Miyahara, A. A. L., Brienen, R. J., Gloor, E., Boom, A., Grandis, A., & Buckeridge, M. S. (2024). Stress-tolerant trees for resilient cities: Tree-ring analysis reveals species suitable for a future climate. Urban Climate, 55, 101964.
Lopes, H. S., Vidal, D. G., Cherif, N., Silva, L., & Remoaldo, P. C. (2025). Green infrastructure and its influence on urban heat island, heat risk, and air pollution: A case study of Porto (Portugal). Journal of Environmental Management, 376.
Lovell, S. T., & Taylor, J. R. (2013). Supplying urban ecosystem services through multifunctional green infrastructure in the United States. Landscape Ecology, 28, 1447–1463.
Ma, M., Gao, Y., Ding, A., Su, H., Liao, H., Wang, S., Wang, X., Zhao, B., Zhang, S., Fu, P., Guenther, A.B., Wang, M., Li, S., Chu, B., Yao, X., Gao, H., 2022. Development and Assessment of a High-Resolution Biogenic Emission Inventory from Urban Green Spaces in China. Environ. Sci. Technol. 56, 175–184.
Maison, A., Lugon, L., Park, S.-J., Baudic, A., Cantrell, C., Couvidat, F., d'Anna, B., Karl, T., Kuenen, J. J. P., Lannuque, V., Marais, E. A., Mellouki, A., Petetin, H., Sartelet, K., Tournadre, B., Valari, M., Zaveri, R. A., & Gros, V. (2024). Significant impact of urban

tree biogenic emissions on air quality estimated by a bottom-up inventory and chemistry transport modeling. Atmospheric Chemistry and Physics, 24, 6011–6046.

Manavvi, S., & Milosevic, D. (2025). Chasing cool: Unveiling the influence of green-blue features on outdoor thermal environment in Roorkee (India). Building and Environment, 267, 112238.

Mancilla, D., Robledo, S., Esenarro, D., Raymundo, V., Vega, V. (2024). Green Corridors and Social Connectivity with a Sustainable Approach in the City of Cuzco in Peru. Urban Science, 8, 79.

Marshall C. A., Wilkinson M. T., Hadfield P. M., et al. (2023). Urban wildflower meadow planting for biodiversity, climate and society: An evaluation at King's College, Cambridge. Ecological Solutions and Evidence 4:e12243.

Martínez-Villa, J. A., Paquette, A., Feeley, K. J., Morales-Morales, P. A., Messier, C., & Durán, S. M. (2024). Changes in morphological and physiological traits of urban trees in response to elevated temperatures within an Urban Heat Island. Tree physiology, 44, 145.

Marzbali, M. H., Abdullah, A., Razak, N. A., & Tilaki, M. J. M. (2012). Validating crime prevention through environmental design construct through checklist using structural equation modelling. International Journal of Law, Crime and Justice, 40, 82-99.

Matsler A. M. (2019). Making ‘green’fit in a ‘grey’accounting system: The institutional knowledge system challenges of valuing urban nature as infrastructural assets. Environmental Science & Policy 99:160-168.

Matsumoto, M., Kiyomizu, T., Yamagishi, S., Kinoshita, T., Kumpitsch, L., Kume, A., Hanba, Y.T. (2022). Responses of photosynthesis and long-term water use efficiency to ambient air pollution in urban roadside trees. Urban Ecosystems, 25, 1029-1042.

McNabb, T., Charters, F. J., Challies, E., & Dionisio, R. (2024a). Unlocking urban blue-green infrastructure: an interdisciplinary literature review analysing co-benefits and synergies between bio-physical and socio-cultural outcomes. Blue-Green Systems, 6, 217-231.

McNabb, T., Charters, F., Dionisio, R., & Challies, E. (2024b). Design and implementation of blue-green infrastructure for socio-cultural benefits at community scales: The case of Wigram Basin in Ōtautahi Christchurch. Nature-Based Solutions, 6, 100192.

Meerow, S., & Newell, J. P. (2017). Spatial planning for multifunctional green infrastructure: Growing resilience in Detroit. Landscape and Urban Planning, 159, 62–75.

Ming, T., Hu, Y., Shi, T., Li, Y., Hu, S., Yang, D., Lv, B., Peng, C., & Chen, Y. (2025). Effect of Blue–Green Infrastructure in mitigating microenvironmental heat islands: Field- and simulation-based insights. Atmosphere, 16, 134.

Minnema, L. (2019). Cross-Cultural Comparisons between the Mughal Tomb Garden of Taj Mahal in Agra (India) and the Dry Landscape Garden of the Ryoan-Ji Zen Monastery in Kyoto (Japan). Worldviews: Global Religions, Culture, and Ecology, 23, 197–229.

Miyahara, A. A. L., Wild, T., Sandre, A. A., Pellegrino, P. R. M., da Silva Filho, C. A., Buckeridge, M. S., & Locosselli, G. M. (2022). Developing and classifying urban biomes as a basis for nature-based solutions. Urban Climate, 45, 101251.

Morgan, D. T., Daly, T., Gallagher, J., & McNabola, A. (2017). Reducing energy consumption and increasing filter life in HVAC systems using an aspiration efficiency reducer: Long-term performance assessment at full-scale. Journal of Building Engineering, 12, 267-274.

Nordh, H. Hartig, T., Hagerhall, C.M., & fry, G.(2009). Components of small urban parks that predict the possibility for restoration. Urban Forestry & Urban Greening, 8, 225–235.Piroozfar, P., Farr, E. R., Aboagye-Nimo, E., & Osei-Berchie, J. (2019). Crime prevention in urban spaces through environmental design: A critical UK perspective. Cities, 95, 102411.

Nordman E. E., Isely E., Isely P., et al. (2018). Benefit-cost analysis of stormwater green infrastructure practices for Grand Rapids, Michigan, USA. Journal of Cleaner Production 200:501-510.

Nowak, D. J., & Crane, D. E. (2002). Carbon storage and sequestration by urban trees in the USA. Environmental Pollution, 116, 381–389.

Nowak, D.J., Greenfield, E.J., Hoehn, R.E., Lapoint, E. (2013). Carbon storage and sequestration by trees in urban and community areas of the United States. Environmental Pollution, 178, 229–236.

Oliveira, P. M. B., Almeida, R. M., & Cardoso, S. J. (2024). Effects of floating photovoltaics on aquatic organisms: a review. Hydrobiologia, 1-16.

Palusci, O. & Cecere, C. (2022). Urban Ventilation in the Compact City: A Critical Review and a Multidisciplinary Methodology for Improving Sustainability and Resilience in Urban Areas. Sustainability, 14, 3948.

Pandey, G., Lyden, S., Franklin, E., Millar, B., & Harrison, M. T. (2025a). A systematic review of agrivoltaics on productivity, profitability, and environmental co-benefits. Sustainable Production and Consumption, 56, 13-36.

Papadopoulou, S., Stefi, A. L., Christodoulakis, N. S., Gkikas, D., & Rhizopoulou, S. (2023). Structural and Physiological Traits of Compound Leaves of Ceratonia siliqua Trees Grown in Urban and Suburban Ambient Conditions. Plants, 12, 514.

Park, B. J., Lee, D. K., Yun, S. H., Kim, E. S., Lee, J. H., & Kim, S. H. (2024). Assessing the impact of green infrastructure on thermal comfort in relation to humidity: A case study in Korea. Urban Forestry & Urban Greening, 95, 128305.

Pauleit, S., Ambrose-Oji, B., Andersson, E., Anton, B., Buijs, A., Haase, D., Elands, B., Hansen, R., Kowarik, I., Kronenberg, J., Mattijssen, T., Stahl Olafsson, A., Rall, E., Van Der Jagt, A. P. N., & Konijnendijk Van Den Bosch, C. (2019). Advancing urban green infrastructure in Europe: Outcomes and reflections from the GREEN SURGE project. Urban Forestry & Urban Greening, 40, 4–16.

Petersen C. J., Russel D. J., Jensen A., et al. (2024). Walkable maps and policy innovation for nature: a novel methodology for understanding policy learning. International Journal of Qualitative Methods 23:16094069241254006.

Pincetl, S., Gillespie, T.W., Pataki, D.E., Porse, E., Jia, S., Kidera, E., Nobles, N., Rodriguez, J., & Choi, D. (2019). Evaluating the effects of turf-replacement programs in Los Angeles. Landscape and Urban Planning, 185, 210–221.

Pinto, D. M., Blande, J. D., Souza, S. R., Nerg, A. M., & Holopainen, J. K. (2010). Plant volatile organic compounds (VOCs) in ozone (O3) polluted atmospheres: the ecological effects. J Chem Ecol, 36, 22-34.

Piroozfar, P., Farr, E. R., Aboagye-Nimo, E., & Osei-Berchie, J. (2019). Crime prevention in urban spaces through environmental design: A critical UK perspective. Cities, 95, 102411.

Pradilla, G., & Hack, J. (2024). An urban rivers renaissance? Stream restoration and green–blue infrastructure in Latin America – Insights from urban planning in Colombia. Urban Ecosystems. https://doi.org/10.1007/s11252-024-01571-9

Pritipadmaja, Garg, R. D. & Sharma, A. K. (2023). Assessing the cooling effect of blue-green spaces: implications for Urban Heat Island mitigation. Water, 15, 2983.

Pugh, T. A. M., MacKenzie, A. R., Whyatt, J. D., & Hewitt, C. N. (2012). Effectiveness of green infrastructure for improvement of air quality in urban street canyons. Environmental Science & Technology, 46, 7692–7699.

Ramasubramanian, P., Luhung, I., Lim, S. B. Y., Schuster, S. C., Starry, O., and Gall, E. T. (2021). Impact of Green and White Roofs on Air Handler Filters and Indoor Ventilation Air. Build. Environ., 197, p. 107860.

Ramasubramanian, P., Starry, O., Rosenstiel, T., Gall, E.T. (2019). Pilot study on the impact of green roofs on ozone levels near building ventilation air supply. Build. Environ. 151, 43–53. https://doi.org/10.1016/j.buildenv.2019.01.023

Raymond, C. M., Frantzeskaki, N., Kabisch, N., Berry, P., Breil, M., Nita, M. R., Geneletti, D., & Calfapietra, C. (2017). A framework for assessing and implementing the co-benefits of nature-based solutions in urban areas. Environmental Science & Policy, 77, 15–24.

RECLAIM (2025). Third RECLAIM Network Plus Conference. https://www.youtube.com/watch?v=ekK0gCXmV5s&list=PL4xPfJG-Mbo4LvhL3rtVptHETgLU6w0GX&index=69.

Reitberger, R., Kooniyara, V.P., Parhizgar, L., & Roetzer, T. (2025). Tree growth simulation in Geographic Information Systems: Coupling CityTree and ArcGIS for solar radiation analysis. Sustainable Cities and Society, 120, 106128.

Řezníček, H., Geletič, J., Belda, M., Beneš, L., Bureš, M., Eben, K., Fuka, V., Krč, P., Michálek, P., Patiño, W., Radović, J., Sühring, M., Vlček, O., Resler, J., 2025. Analysis of the complex role of trees in street canyons using a large-eddy simulation model. Quarterly Journal of the Royal Meteorological Society n/a, e4954.

Ribeiro, A. P., Bollmann, H. A., de Oliveira, A., Rakauskas, F., Cortese, T. T. P., Rodrigues, M. S. C., Quaresma, C. C., & Ferreira, M. L. (2023). The role of tree landscape to reduce effects of urban heat islands: a study in two Brazilian cities. Trees - Structure and Function, 37(1), 17–30.

Rigolon A. and Christensen J. (2019). Greening without gentrification. Parks and recreation. Available online: Parks-Related-Anti-Displacement-Strategies-report-with-appendix.pdf (accessed on 09 May 2025).

Rosentreter, J.A., Borges, A.V., Deemer, B.R., Holgerson, M.A., Liu, S., Song, C., Melack, J., Raymond, P.A., Duarte, C.M., Allen, G.H., Olefeldt, D., Poulter, B., Battin, T.I., & Eyre, B.D. (2021). Half of global methane emissions come from highly variable aquatic ecosystem sources. Nature Geoscience, 14, 225–230

Saher, R., Ott, T. (2025). Assessing the irrigation water requirement and irrigation water use at a house scale in Las Vegas Valley. Agricultural Water Management, 308, 109278.

Salmond, J.A., Williams, D.E., Laing, G., Kingham, S., Dirks, K., Longley, I., Henshaw, G.S. (2013). The influence of vegetation on the horizontal and vertical distribution of pollutants in a street canyon. Science of The Total Environment, 443, 287–298.

Salvador, C. M., Chou, C. C. K., Ho, T.-T., Tsai, C.-Y., Tsao, T.-M., Tsai, M.-J., & Su, T.-C. (2020). Contribution of Terpenes to Ozone Formation and Secondary Organic Aerosols in a Subtropical Forest Impacted by Urban Pollution. Atmosphere, 11, 1232.

Säumel, I., Weber, F., & Kowarik, I. (2016). Toward livable and healthy urban streets: Roadside vegetation provides ecosystem services where people live and move. Environmental Science & Policy, 62, 24-33.

Sawers, B. (2019). Controlling Biogenic Volatile Organic Compounds for Air Quality. Indiana Law Journal, 94, 79-90.

Shi, Z., Yang, J., Zhang, Y., Xiao, X., Xia, C.J. (2022). Urban ventilation corridors and spatiotemporal divergence patterns of urban heat island intensity: a local climate zone perspective. Environmental Science and POllution Research, 29, 74394–74406.

Shokry, G., Anguelovski, I., & Connolly, J. J. T. (2023). (Mis-)belonging to the climate-resilient city: Making place in multi-risk communities of racialized urban America. Journal of Urban Affairs.

Sierra-Heredia, C., North, M., Brook, J., Daly, C., Ellis, A. K., Henderson, D., Henderson, S. B., Lavigne, É., & Takaro, T. K. (2018). Aeroallergens in Canada: Distribution, public health impacts, and opportunities for prevention. International Journal of Environmental Research and Public Health, 15, 1577.

Silva Luz, C. L. D., Reale, R., Candido, L. F., Zappi, D., & Locosselli, G. M. (2024). Using Morphological Characters to Support Decision-Making in Nature-Based Solutions: A Shortcut to Promote Urban Plant Biodiversity. Urban Science, 8, 233.

Sjöman, H., Östberg, J., & Bühler, O. (2012). Diversity and distribution of the urban tree population in ten major Nordic cities. Urban Forestry & Urban Greening, 11, 31–39.

Skandalos, N., & Karamanis, D. (2025). Decarbonizing operational emissions in urban neighborhoods with the integration of rooftop photovoltaics and green infrastructure under current and future climate conditions. Energy & Buildings, 329, 115306.

Sotillo, A., Hardion, L., Chanez, E., Fujiki, K., & Muratet, A. (2024). Plant responses to urban gradients: Extinction, plasticity, adaptation. Journal of Ecology, 112, 2861-2875.

Sousa-Silva, R., Smargiassi, A., Kneeshaw, D., Dupras, J., Zinszer, K., & Paquette, A. (2021). Strong variations in urban allergenicity riskscapes due to poor knowledge of tree pollen allergenic potential. Scientific Reports, 11, 10196.

Stawoska, I., Myszkowska, D., Oliwa, J., Skoczowski, A., Wesełucha-Birczyńska, A., Saja-Garbarz, D., & Ziemianin, M. (2023). Air pollution in the places of Betula pendula growth and development changes the physicochemical properties and the main allergen content of its pollen. Plos one, 18, e0279826.

Sun, Hao, Carlos Jimenez-Bescos, Murtaza Mohammadi, Fangliang Zhong, and John Kaiser Calautit. (2021). Numerical investigation of the influence of vegetation on the aero-thermal performance of buildings with courtyards in hot climates. Energies 14, 5388.

Sun, X., Tan, X., Chen, K., Song, S., Zhu, X., & Hou, D. (2020). Quantifying landscape-metrics impacts on urban green-spaces and water-bodies cooling effect: The study of Nanjing, China. Urban Forestry & Urban Greening, 55, 126838.

Tache A.-V., Popescu O.-C. and Petrișor A.-I. (2024). Planning Blue–Green Infrastructure for Facing Climate Change: The Case Study of Bucharest and Its Metropolitan Area. Urban Science 8:250.

Talal, M. L., Santelmann, M. V., & Tilt, J. H. (2021). Urban park visitor preferences for vegetation – An on-site qualitative research study. Plants, People, Planet, 3, 375–388.

Tan, X., Sun, X., Huang, C., Yuan, Y., & Hou, D. (2021). Comparison of cooling effect between green space and water body. Sustainable Cities and Society, 67, 102711.

Tang, Y.-F., Wen, Y.-B., Chen, H., Tan, Z.-C., Yao, Y.-H., & Zhao, F.-Y. (2023). Airflow Mitigation and Pollutant Purification in an Idealized Urban Street Canyon with Wind Driven Natural Ventilation: Cooperating and Opposing Effects of Roadside Tree Plantings and Non-uniform Building Heights. Sustainable Cities and Society, 92, 104483.

Tao, Q., Gao, G., Xi, H., Wanf, F., Cheng, X., Ou, W., Tao, Y. (2022). An integrated evaluation framework for multiscale ecological protection and restoration based on multi-scenario trade-offs of ecosystem services: Case study of Nanjing City, China.Ecological Indicators, 140, 108962.

Theeuwes, N. E., Solcerová, A., & Steeneveld, G. J. (2013). Modeling the influence of open water surfaces on the summertime temperature and thermal comfort in the city. Journal of Geophysical Research: Atmospheres, 118(16), 8881–8896.

Tomateo, C. (2021). Indigenous land systems and emerging of Green Infrastructure planning in the Peruvian coastal desert: Tensions and opportunities. Journal of Environmental Policy & Planning, 23, 683–700.

Triyuly, W., Triyadi, S., & Wonorahardjo, S. (2021). Synergising the thermal behaviour of water bodies within thermal environment of wetland settlements. International Journal of Energy and Environmental Engineering, 12(1), 55–68.

van Bergen, T.J.H.M., Barros, N., Mendonça, R., Aben, R.C.H., Althuizen, I.H.J., Huszar, V., Lamers, L.P.M., Lürling, M., Roland, F., Kosten, S. (2019). Seasonal and diel variation in greenhouse gas emissions from an urban pond and its major drivers. Limnology and Oceanography, 64, 2129–2139.

Van Mechelen, C., Dutoit, T., Hermy, M. (2015). Adapting green roof irrigation practices for a sustainable future: A review. Sustainable Cities and Society, 19, 74–90.

Vasudevan, M., Pilla, F., & McNabola, A. (2024). Assessment of pollution removal mechanisms in steep-asymmetric city-type environments using wind deflectors. Energy & Built Environment (in press).

Velasco, E., Roth, M., Norford, L., Molina, L.T. (2016). Does urban vegetation enhance carbon sequestration? Landscape and Urban Planning, 148, 99–107.

Wahba, S.M., Kamel, B.A., Nassar, K.M., Abdelsalam, A.S. (2018). Effectiveness of Green Roofs and Green Walls on Energy Consumption and Indoor Comfort in Arid Climates. Civ. Eng. J. 4, 2284–2295.

Wang H. X., Zhao, J. H., Lu, J., Ge, J., Lv, G. Q., Luo, X. Y., Lin, H. Q., Gu, M. Y. (2025). Combined impacts of vertical greening and permeable pavement systems on street Canyons' microclimate in hot and humid regions in China. Urban Climate 59:102333.

Wang, W., He, J. & Wang, X. (2024). Quantitatively comparing the morphological influences on the cool island effect in urban waterfront blue-green spaces across six cities near 30° N. Urban Climate, 56, 102076.

Wang, W., Yang, H., & Xiang, C. (2023a). Green roofs and facades with integrated photovoltaic system for zero energy eco-friendly building–A review. Sustainable Energy Technologies and Assessments, 60, 103426.

Wang, Y., Li, B., Bao, P., Wang, R., Min, A., & Xiong, P. (2023). A Case Study of Leaf Wettability Variability and the Relations with Leaf Traits and Surface Water Storage for Urban Landscape Plants. Water, 15, 2152.

Wedyan M. and Saeidi-Rizi F. (2025). Assessing the impact of walkability indicators on health outcomes using machine learning algorithms: A case study of Michigan. Travel Behaviour and Society 39:100983.

Wei, Y., Li, Z., Zhang, J., & Hu, D. (2023). Effects of artificial light at night and drought on the photosynthesis and physiological traits of two urban plants. Frontiers in Plant Science, 14, 1263795.

Wilfong, M., Litvak, E., Grijseels, N.H., Hamilton, K., Kucera, D., Welsh, L., Endter-Wada, J., Jenerette, G.D., Pataki, D.E. (2025). Irrigation rates and turfgrass evapotranspiration in cities with contrasting water availability. Journal of the American Water Resources Association, 61, e13236.

Wolch, J.R., Byrne, J. & Newell, J.P. (2014). Urban green space, public health, and environmental justice: The challenge of making cities 'just green enough'. Landscape and Urban Planning, 125, 234-244.

Wong C.P., Jiang B. Kinzig A.P., & Ouyangi Z. (2018). Quantifying multiple ecosystem services for adaptive management of green infrastructure. Ecosphere, 9, e02495.

Woszczyk, M., & Schubert, C. J. (2021). Greenhouse gas emissions from Baltic coastal lakes. Science of The Total Environment, 755, 143500.

Woudstra, Y., Kraaiveld, R., Jorritsma, A., Vijverberg, K., Ivanovic, S., Erkens, R., Huber, H., Gravendeel, B., & Verhoeven, K. J. (2024). Some like it hot: adaptation to the urban heat island in common dandelion. Evolution Letters, 8, 881-892.

Xiong Y., Zhang J., Xu X., et al. (2020). Strategies for improving the microclimate and thermal comfort of a classical Chinese garden in the hot-summer and cold-winter zone. Energy and Buildings 215:109914.

Xiong, G., He, R., Wang, G., Hong, J., & Jin, Y. (2024). Environmental Inequalities in Ecosystem Services Benefits of Green Infrastructure: A Case Study from China. Forests, 15(1).

Xiong, J., Li, B., Short, C. A., Kumar, P., & Pain, C. (2024). Comprehensive evaluation of natural ventilation potential of buildings in urban areas under the influence of multiple environment-related factors. Journal of Building Engineering, 89, 109218.

Xu, X., Luo, F., Wang, W., Hong, T., & Fu, X. (2018). Performance-Based Evaluation of Courtyard Design in China's Cold-Winter Hot-Summer Climate Regions. Sustainability, 10, 3950.

Yang, G., Yu, Z., Jørgensen, G., & Vejre, H. (2020). How can urban blue-green space be planned for climate adaption in high-latitude cities? A seasonal perspective. Sustainable Cities and Society, 53, 101932.

Yang, G., Yu, Z., Jørgensen, G., & Vejre, H. (2020). How can urban blue-green space be planned for climate adaption in high-latitude cities? A seasonal perspective. Sustainable Cities and Society, 53, 101932.

Yao, L., Sailor, D., Yang, X., Xu, G., Zhao, L. (2023). Are water bodies effective for urban heat mitigation? Evidence from field studies of urban lakes in two humid subtropical cities, Building and Environment, 245, 110860.

Yin, J., Chen, X., Xie, W., & Wen, L. (2025). Urban lakes as significant sources of greenhouse gas (CO2, CH4, and N2O) emissions: Insights from field measurements and statistical analyses. Environmental Monitoring and Assessment, 197(5), 1–18.

Yuan Q., Meng F., Li W., et al. (2025). Tradeoff optimization of urban roof systems oriented to food-water-energy nexus. Applied Energy 380:124987.

Yuan, Q., Lian, J., Yang, F., Shen, M., Wang, Y., Kong, Q., Chen, B., Cai, X., Tao, H., & Wu, H. (2025). A systematic review on greenhouse gas emissions from constructed wetlands: Focusing on effects of planting strategies and emission reduction measures. Journal of Water Process Engineering, 69, 106696.

Zhang, H., Yu, Z., Zhu, C., Yang, R., Yan, B., & Jiang, G. (2023a). Green or not? Environmental challenges from photovoltaic technology. Environmental Pollution, 320, 121066.

Zhang, N., Wang, G., Gallagher, J., Song, Q., Tam, V. W. Y., & Duan, H. (2020). A dynamic analysis of the global warming potential associated with air conditioning at a city scale: an empirical study in Shenzhen, China, Environmental Impact Assessment Review, 81, 106354

Zhang, W., Li, H., Xiao, Q., & Li, X. (2021). Urban rivers are hotspots of riverine greenhouse gas (N2O, CH4, CO2) emissions in the mixed-landscape chaohu lake basin. Water Research, 189, 116624.

Zhang, X., Buddhika, J. W. G., Wang, J., Weerasuriya, A. U., & Tse, K. T. (2023b). Numerical investigation of effects of trees on cross-ventilation of an isolated building. Journal of Building Engineering, 73, 106808.

Zhang, Y., & Steiner, A. L. (2022). Projected climate-driven changes in pollen emission season length and magnitude over the continental United States. Nature communications, 13, 1234.
Zhao, Z., Li, H., Wang, S. (2024). Machine learning-based surrogate models for fast impact assessment of a new building on urban local microclimate at design stage. Build. Environ. 266, 112142.
Zhao N., Prieur J.-F., Liu Y., et al. (2021). Tree characteristics and environmental noise in complex urban settings–A case study from Montreal, Canada. Environmental Research 202:111887.
Zhong, Q., & Tong, D. (2020). Spatial layout optimization for solar photovoltaic (PV) panel installation. Renewable energy, 150, 1-11.
Zhu Y., Ding J., Zhu Q., et al. (2017). The impact of green open space on community attachment—A case study of three communities in Beijing. Sustainability 9:560.
Schweinberger M. C. (2023). Debt for Nature Swaps-Birth of a New Asset Class? Available at SSRN 4843793.
