# Supplementary material for: Overlooked considerations in prescribing green and blue infrastructure solutions for urban environments
Source: Innovation (Camb). 2025 Nov 19;7(5):101184. doi: 10.1016/j.xinn.2025.101184 (PMC13148010; doi:10.1016/j.xinn.2025.101184)
Supplement: Document S2. Article plus supplemental information [file mmc2.pdf]

REVIEW Environment & Ecology

OPEN ACCESS

Check for updates

Overlooked considerations in prescribing green and blue infrastructure solutions for urban environments

Prashant Kumar,[1,2,16,19,*] Karina Corada Perez,[3] Akash Biswal,[1] Hao Sun,[1] Anubhav Kumar Dwivedi,[1] Sarkawt Hama,[1] Soheila Khalili,[1,2] Ajit Ahlawat,[4,5] Maria de Fatima Andrade,[6] Ronaldo Adriano Alves,[7] Emannuelly A. Amaral dos Santos,[8] Maria Athanassiadou,[9] Camilo Bastos Ribeiro,[10] Prabin Bhusal,[11,12] Miguel Luiz Bucalem,[13] Bonnie G. Buchanan,[14] Leticia Figueiredo Candido,[15] Shi-Jie Cao,[1,16] Amarilis Lucia Casteli Figueiredo Gallardo,[13,17] Ruidong Chang,[18] Amanda K. Chaves Ribeiro,[8] Brian Considine,[19,41] Regina Maura de Miranda,[20] Letícia Aparecida de Paiva,[21] Priyanka de Souza,[22,23] Marco A. Franco,[6] Edmilson D. Freitas,[6] H. Christopher Frey,[24] Marco F. Funari,[1,2] Bruno Furieri,[25] John Gallagher,[19] Leandro Luiz Giatti,[26] Marcos Jeronimo Goroski Rambalducci,[27] Christos H. Halios,[28] Felicity Harris,[29] Leonardo Hoinaski,[10] Colin Horton,[30] Yuhan Huang,[31] Laurence Jones,[32,33] Robyn Jones,[34] John Kandulu,[35] Madhusudan Katti,[11] Giuliano Maselli Locosselli,[15,36] Augusto Akio Lucchezi Miyahara,[15] Jorge Alberto Martins,[27] Leila Droprinchinski Martins,[27] Mauricio Cruz Mantoani,[36] Roberta Consentino Kronka Mülfarth,[37] Yasmin Kaore Lago Kitagawa,[38] Willian Lemker Andreão,[39] Jackson Lemons,[11] Giulia Mariano Machado,[26] Shelagh K. Malham,[40] Meredith P. Martin,[11] Maria Clara V.M. Starling,[8] Aonghus McNabola,[19,41] Otavio Medeiros Sobrinho,[8] Eugene Mohareb,[28] Erick G. Sperandio Nascimento,[1,42,43] Thiago Nogueira,[26] Gwilym Owen,[44] Rajan Parajuli,[11] Hari Prasad Pandey,[45,46] Rizzieri Pedruzzi,[47] Pedro José Pérez Martínez,[21] Janaina Antonino Pinto,[48] Jorge Armando Piscoya Santibañez,[6] Shila Pokhrel,[11,46] Paula Lelis Rabelo Albala,[37,58] Neyval C. Reis,[25] Anderson P. Rudke,[8] Devendra Saroj,[1,2] Yiming Sui,[28] Veronica Soebarto,[18] Yonatal Tefera,[49] Taciana Toledo de Almeida Albuquerque,[8] Bruna Lima Veras Maia,[8] Fang Wang,[50,51,59] Jannis Wenk,[53,54] Robson Will,[10] Carmel Williams,[49] Hannah Sloan Wood,[55] Qingyun Wu,[31] Chang Xi,[16] Russell Yates,[56] and Runming Yao[28,52,57]

*Correspondence: p.kumar@surrey.ac.uk

Received: May 30, 2025; Accepted: November 14, 2025; Published Online: November 19, 2025; https://doi.org/10.1016/j.xinn.2025.101184

© 2025 The Author(s). Published by Elsevier Inc. on behalf of Youth Innovation Co., Ltd. This is an open access article under the CC BY license (http://creativecommons.org/licenses/by/4.0/).

GRAPHICAL ABSTRACT

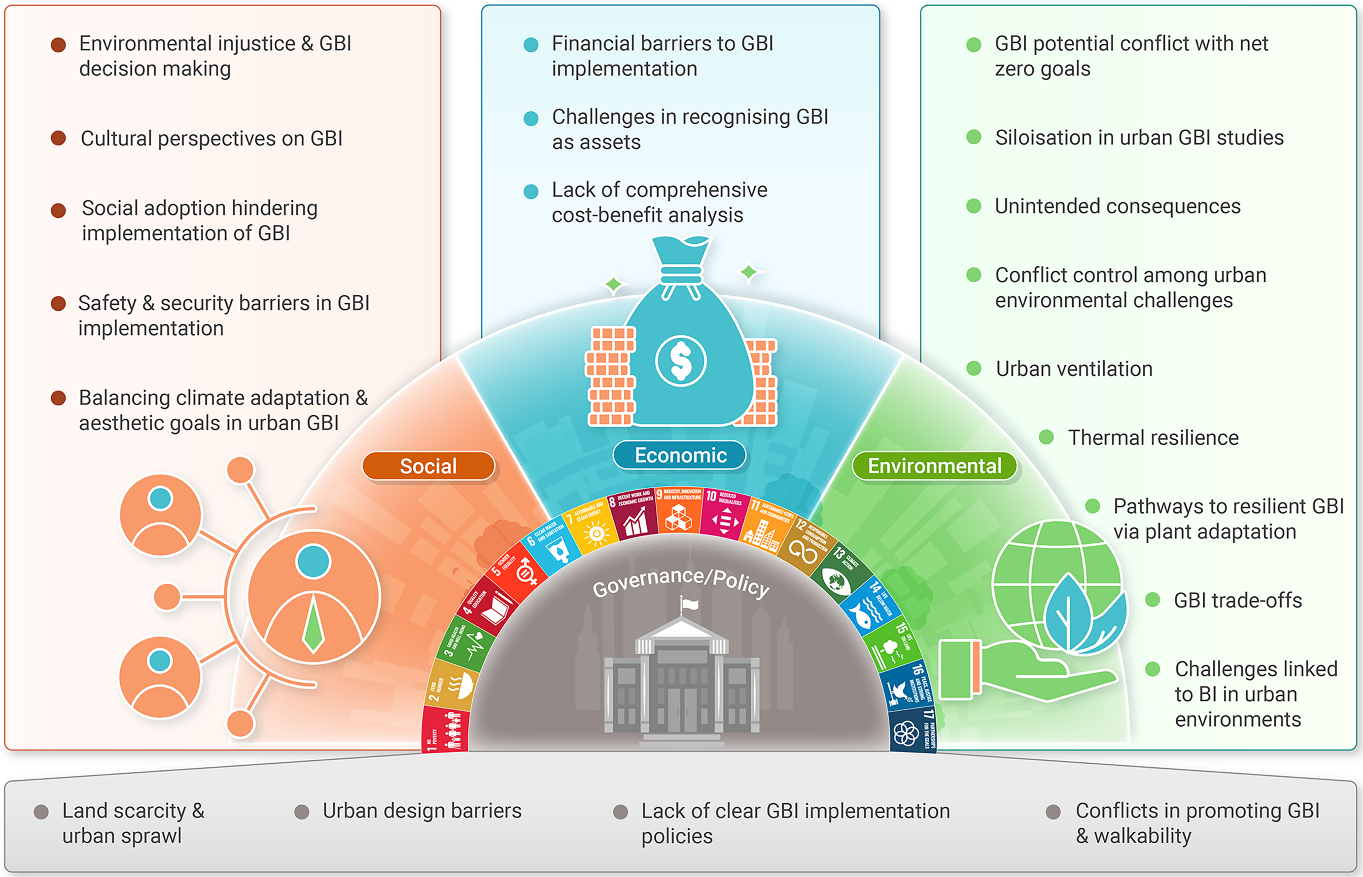

PUBLIC SUMMARY

- 21 unseen barriers to green/blue infrastructure (GBI) are identified, with key solutions to fix these issues.
- GBI environmental barriers include green energy clashes, unwanted side effects, and urban plant stress.
- GBI social barriers include cultural/equity issues, safety concerns, design tastes, and need community input.
- GBI financial barriers include undervaluation, investment gaps, and limited funding/benefit recognition.
- GBI governance barriers include land limits, traditional designs, policy gaps, and competing urban priorities.

Overlooked considerations in prescribing green and blue infrastructure solutions for urban environments

Prashant Kumar,[1,2,16,19,*] Karina Corada Perez,[3] Akash Biswal,[1] Hao Sun,[1] Anubhav Kumar Dwivedi,[1] Sarkawt Hama,[1] Soheila Khalili,[1,2] Ajit Ahlawat,[4,5] Maria de Fatima Andrade,[6] Ronaldo Adriano Alves,[7] Emannuelly A. Amaral dos Santos,[8] Maria Athanassiadou,[9] Camilo Bastos Ribeiro,[10] Prabin Bhusal,[11,12] Miguel Luiz Bucalem,[13] Bonnie G. Buchanan,[14] Leticia Figueiredo Candido,[15] Shi-Jie Cao,[1,16] Amarilis Lucia Casteli Figueiredo Gallardo,[13,17] Ruidong Chang,[18] Amanda K. Chaves Ribeiro,[8] Brian Considine,[19,41] Regina Maura de Miranda,[20] Letícia Aparecida de Paiva,[21] Priyanka de Souza,[22,23] Marco A. Franco,[6] Edmilson D. Freitas,[6] H. Christopher Frey,[24] Marco F. Funari,[1,2] Bruno Furieri,[25] John Gallagher,[19] Leandro Luiz Giatti,[26] Marcos Jeronimo Goroski Rambalducci,[27] Christos H. Halios,[28] Felicity Harris,[29] Leonardo Hoinaski,[10] Colin Horton,[30] Yuhan Huang,[31] Laurence Jones,[32,33] Robyn Jones,[34] John Kandulu,[35]

(Author list continued on next page)

[1]Global Centre for Clean Air Research (GCARE), School of Engineering, Civil and Environmental Engineering, Faculty of Engineering and Physical Sciences, University of Surrey, Guildford GU2 7XH, UK
[2]Institute for Sustainability, University of Surrey, Guildford GU2 7XH, UK
[3]Sustainability Research Institute, University of East London, London E16 2RD, UK
[4]Department of Geoscience and Remote Sensing, Delft University of Technology (TU Delft), 2628 CN Delft, the Netherlands
[5]Leibniz Institute for Tropospheric Research, e.V. (TROPOS), 04318 Leipzig, Germany
[6]Institute of Astronomy, Geophysics and Atmospheric Sciences, Department of Atmospheric Sciences, University of São Paulo, São Paulo 05508-090, Brazil
[7]Department of Geography, State University of Londrina, Londrina 86057-970, Brazil
[8]Department of Sanitary and Environmental Engineering, School of Engineering, Federal University of Minas Gerais, Belo Horizonte 31270-901, Brazil
[9]Urban Climate Applications, Met Office, Exeter EX1 3PB, UK
[10]Department of Sanitary and Environmental Engineering, Federal University of Santa Catarina, Florianópolis 88040-070, Brazil
[11]Department of Forestry and Environmental Resources, North Carolina State University, Raleigh, NC 27695, USA
[12]Institute of Forestry, Tribhuvan University, Pokhara 33700, Nepal
[13]Polytechnic School, University of São Paulo, São Paulo 05508-010, Brazil
[14]Sustainable and Explainable Fintech (SAEF) Center, University of Surrey, Guildford GU2 7XH, UK
[15]Institute of Environmental Research from the State of São Paulo, São Paulo 01061-970, Brazil
[16]School of Architecture, Southeast University, 2 Sipailou, Nanjing 210096, China
[17]Smart and Sustainable Cities Program at the University Nove de Julho, São Paulo 01525-000, Brazil
[18]School of Architecture and Civil Engineering, The University of Adelaide, Adelaide, SA 5005, Australia
[19]Department of Civil, Structural & Environmental Engineering, Trinity College Dublin, The University of Dublin, Dublin D02 PN40, Ireland
[20]School of Arts, Sciences and Humanities, University of São Paulo, São Paulo 03828-000, Brazil
[21]Faculty of Engineering, Architecture and Urbanism, University of Campinas, Campinas 13083-970, Brazil
[22]Department of Urban and Regional Planning, University of Colorado Denver, Denver, CO 80202, USA
[23]CU Population Center, University of Colorado Boulder, Boulder, CO 80302, USA
[24]Department of Civil, Construction, and Environmental Engineering, North Carolina State University, Raleigh, NC 27606, USA
[25]Department of Environmental Engineering, Federal University of Espírito Santo, Vitória, ES 29075-910, Brazil
[26]Department of Environmental Health, School of Public Health, University of São Paulo, São Paulo 01246904, Brazil
[27]Federal University of Technology, Parana 80230-910, Brazil

(Affiliations continued on next page)

Green and blue infrastructure (GBI) is emerging as a key strategy for climate adaptation and urban resilience, yet its implementation often faces critical contextual barriers. This review initially screened over 29,000 publications, ultimately synthesizing more than 500 relevant studies supplemented by diverse expert input. The result is a novel integrative framework that connects previously siloed knowledge and consolidates 21 underexplored barriers across four key domains of GBI implementation: environmental, social, economic, and governance/policy. Environmental barriers include conflicts between GBI and renewable energy goals, specifically photovoltaics, unintended consequences of GBI (such as allergenic pollen production), urban ventilation disruption, and vulnerability of plant species to multiple urban stressors. Effective responses include thoughtful allocation and integration of photovoltaics and GBI, developing context-specific frameworks combining ecological knowledge with technological innovation, fostering cross-disciplinary collaboration across technical and social domains, science-based species selection and implementing multi-scalar strategies that enhance ecological connectivity. Social barriers encompass environmental injustice, cultural disconnection, limited public adoption, safety concerns, and esthetic preferences favoring manicured over ecologically functional landscapes. These challenges highlight the need for participatory design, culturally responsive planning, and inclusive resource allocation to strengthen community engagement and long-term stewardship. Economic barriers stem from biodiversity undervaluation, inadequate asset recognition in accounting frameworks, incomplete cost-benefit analyses, and limited private investment. Innovative financing tools such as green bonds and debt-for-nature swaps offer promising mechanisms for resilient financing, while standardized natural capital accounting frameworks can better capture GBI's multifunctional value. Governance barriers include land scarcity, urban design limitations, policy fragmentation, and disconnects with other urban agendas such as walkability. Overcoming these requires institutional realignment, cross-sectoral collaboration, and integrated spatial planning. The review unifies these findings into 12 actionable recommendations to support holistic decision-making, emphasizing that effective GBI implementation demands context-specific strategies combining innovation, inclusive governance, and long-term stewardship to mainstream GBI in sustainable urban development.

INTRODUCTION

Cities now house ∼55% of the global population, projected to reach 68% by 2050 (Figure S1).[1] Due to high population and building density, cities face serious environmental issues namely air pollution, heat island effects, floods,[2]

www.the-innovation.org

Madhusudan Katti,[11] Giuliano Maselli Locosselli,[15,36] Augusto Akio Lucchezi Miyahara,[15] Jorge Alberto Martins,[27] Leila Droprinchinski Martins,[27] Mauricio Cruz Mantoani,[36] Roberta Consentino Kronka Mülfarth,[37] Yasmin Kaore Lago Kitagawa,[38] Willian Lemker Andreão,[39] Jackson Lemons,[11] Giulia Mariano Machado,[26] Shelagh K. Malham,[40] Meredith P. Martin,[11] Maria Clara V.M. Starling,[8] Aonghus McNabola,[19,41] Otavio Medeiros Sobrinho,[8] Eugene Mohareb,[28] Erick G. Sperandio Nascimento,[1,42,43] Thiago Nogueira,[26] Gwilym Owen,[44] Rajan Parajuli,[11] Hari Prasad Pandey,[45,46] Rizzieri Pedruzzi,[47] Pedro José Pérez Martínez,[21] Janaina Antonino Pinto,[48] Jorge Armando Piscoya Santibañez,[6] Shila Pokhrel,[11,46] Paula Lelis Rabelo Albala,[37,58] Neyval C. Reis,[25] Anderson P. Rudke,[8] Devendra Saroj,[1,2] Yiming Sui,[28] Veronica Soebarto,[18] Yonatal Tefera,[49] Taciana Toledo de Almeida Albuquerque,[8] Bruna Lima Veras Maia,[8] Fang Wang,[50,51,59] Jannis Wenk,[53,54] Robson Will,[10] Carmel Williams,[49] Hannah Sloan Wood,[55] Qingyun Wu,[31] Chang Xi,[16] Russell Yates,[56] and Runming Yao,[28,52,57]

[28]School of Built Environment, University of Reading, Reading RG6 6DF, UK
[29]Portsmouth City Council, Portsmouth PO1 2AL, UK
[30]Rugby Borough Council, Rugby CV21 2RR, UK
[31]Centre for Green Technology, School of Civil and Environmental Engineering, University of Technology Sydney, Sydney, NSW 2007, Australia
[32]UK Centre for Ecology & Hydrology, Deiniol Road, Bangor LL57 2UW, UK
[33]Liverpool Hope University, Department of Geography and Environmental Science, Liverpool L16 9JD, UK
[34]School of Psychology and Sport Science, Bangor University, Bangor LL57 2DG, UK
[35]College of Business, Government and Law, Flinders University, Bedford Park, SA 5042, Australia
[36]Center for Nuclear Energy in Agriculture, University of São Paulo, São Paulo 13416-000, Brazil
[37]Faculty of Architecture, Urbanism and Design, University of São Paulo, São Paulo 05508-080, Brazil
[38]Foundation for Scientific and Cultural Development, Federal University of Lavras, Lavras 37203-202, Brazil
[39]ArcelorMittal, Global Research and Development, Espirito Santo 29161-376, Brazil
[40]School of Ocean Sciences, Bangor University, Menai Bridge, Anglesey LL59 5AU, UK
[41]School of Engineering, RMIT University, Melbourne, VIC 3000, Australia
[42]Surrey Institute for People-Centred Artificial Intelligence, Faculty of Engineering and Physical Sciences, University of Surrey, Guildford GU2 7XH, UK
[43]Stricto Sensu Department, SENAI CIMATEC University, Salvador 41650-010, Brazil
[44]Resilience Unit, Cardiff Council, Cardiff CF10 4UW, UK
[45]University of Southern Queensland, Toowoomba, QLD 4350, Australia
[46]Ministry of Forests and Environment, Government of Nepal, Kathmandu 44600, Nepal
[47]Department of Sanitary and Environmental Engineering, Rio de Janeiro State University, Rio de Janeiro 20550-013, Brazil
[48]Department of Infrastructure and Environment, Faculty of Civil Engineering, Architecture and Urbanism, State University of Campinas, São Paulo 13083-889, Brazil
[49]School of Public Health, The University of Adelaide, Adelaide, SA 5005, Australia
[50]State Key Laboratory of Soil and Sustainable Agriculture, Institute of Soil Science, Chinese Academy of Sciences, Nanjing 210008, China
[51]University of Chinese Academy of Sciences, Beijing 100049, China
[52]Joint International Research Laboratory of Green Buildings and Built Environments (Ministry of Education), Chongqing University, Chongqing 400045, China
[53]Federal Institute of Hydrology (BfG), Department G - Qualitative Hydrology, Am Mainzer Tor 1, 56068 Koblenz, Germany
[54]Department of Chemical Engineering, University of Bath, Bath BA2 7AY, UK
[55]Independent Consultant, 2200 Copenhagen, Denmark
[56]Surrey County Council, Woodhatch, Reigate RH2 8EF, UK
[57]National Centre for International Research of Low-carbon and Green Buildings (Ministry of Science and Technology), Chongqing University, Chongqing 400045, China
[58]Faculty of Architecture and Urbanism, University of Brasília, Brasília 70904-900, Brazil
[59]Joint FAO/IAEA Centre of Nuclear Techniques in Food and Agriculture, International Atomic Energy Agency, Vienna 1400, Austria
*Correspondence: p.kumar@surrey.ac.uk

Received: May 30, 2025; Accepted: November 14, 2025; Published Online: November 19, 2025; https://doi.org/10.1016/j.xinn.2025.101184

© 2025 The Author(s). Published by Elsevier Inc. on behalf of Youth Innovation Co., Ltd. This is an open access article under the CC BY license (http://creativecommons.org/licenses/by/4.0/).

Citation: Kumar P., Corada Perez K., Biswal A., et al., (2026). Overlooked considerations in prescribing green and blue infrastructure solutions for urban environments. The Innovation **7(5)**, 101184.

and droughts,[3] all of which contribute to poor outcomes for human health and biodiversity. These challenges are often addressed separately in a reactive manner rather than holistically. To build resilient cities, implementing green and blue infrastructure (GBI), also called "nature-based solutions (NbSs)" is crucial.[4,5] In this review, we adopt GBI as an umbrella term encompassing green infrastructure (GI) (e.g., parks, street trees, and gardens) and blue infrastructure (BI) (e.g., rivers, ponds, lakes, and natural wetlands), as well as hybrid infrastructure such as green walls, living green roofs, urban agriculture, rain garden, bioswales, permeable paving, and constructed wetlands, which integrate natural elements into or around gray infrastructure.[5,6]

GBI is increasingly recognized as a sustainable strategy to enrich urban resilience, reduce climate risks, and promote ecological sustainability while delivering diverse environmental, health, and economic benefits.[7,8] For instance, in Barcelona, adhering to international exposure recommendations could prevent almost 2,000 premature deaths annually, primarily through increased physical activity and reduced levels of air pollution, traffic congestion, noise, and urban heat, along with improved access to green spaces.[9] Similarly, exposure to greenspace could prevent up to 10% of childhood overweight and obesity cases.[10] School greenery has been linked to a 20% reduction in mental health issues[11] and a 6% improvement in children's memory compared with those with less green surroundings.[12] GBI can increase local property values by 5%–20% depending on proximity and quality of green space.[13] Additionally, strategic urban planning incorporating GBI provides multiple benefits including reduced urban heat, lower cooling energy demand, decreased healthcare costs, and improved stormwater management.[14,15]

Globally, over 130 countries have embedded urban greening initiatives into their national Sustainable Development Goals (SDGs) commitments.[8] These nature-based approaches complement technology-based solutions.[16] In this context, the International Union for Conservation of Nature (IUCN) Global Standard for NbSs connects ecosystem-based approaches to urban greening, supporting SDG implementation.[17] For example, Natural England's Green Infrastructure Framework in the UK sets a 40% green cover for urban residential areas by 2035.[18]

Beyond ecological resilience, GBI fosters cultural vibrancy and environmental education. International frameworks, such as the United Nations (UN) SDGs and UNESCO's initiatives highlight its role in cultural well-being and place-based learning.[19] Urban GBI delivers diverse cultural ecosystem services (CESs) by fostering a sense of place, safeguarding heritage values, and enabling experiential learning in everyday landscapes.[20] National and local initiatives, such as green school initiatives and culturally adaptive landscape planning, further

Table 1. Summary of key relevant review papers from the past decade on GBI research

Author (year)	Key focus area of review	What was covered
Wang et al.[27]	understanding ecosystem services provided by urban GBI, using a bibliometric analysis to map research trends and knowledge clusters	identified major themes such as air quality improvement, biodiversity support, urban cooling, water management, and soil functions
Zarei and Shahab[28]	identifying the success factors and implementation challenges of NbSs in GI, and categorized barriers across institutional, social, economic, and technical dimensions	classified 21 underexplored barriers (e.g., governance gaps, cultural resistance, undervaluation of biodiversity, and financing limitations) from over 500 studies
Chau et al.[29]	understanding the barriers and challenges to implementing GI, with particular insights from Melbourne's urban policy and planning context	recognized obstacles such as fragmented governance, insufficient funding, lack of technical expertise, and limited political prioritization
Seidu et al.[30]	understanding the integration of green and gray infrastructure systems in dense urban regions	presented institutional, technical, financial, and governance challenges, such as a lack of professional capacity, and outlined effective guidelines, including adopting hybrid design approaches and leveraging digital tools
Kim and Kim[31]	understanding the evolution of research on GI for urban flooding	highlighted technical hydrological performance studies toward socio-ecological frameworks, hybrid blue-green-gray systems, and multidisciplinary approaches to GI for flood resilience
Tao et al.[32]	integration of computational fluid dynamics and machine learning for urban GI	examined role of integrated computational fluid dynamics-machine learning approaches for urban GI design, specifically heat mitigation and air quality improvement
Dobrinić et al.[33]	use of computational learning for GI mapping optimization	reviewed various techniques of deep learning for GI mapping used in sustainable urban development
Kumar et al.[8]	barriers, significances, successful case studies, and greening initiatives opportunities in urban settings	emphasized the need for a holistic, inclusive, and cross-sectoral collaboration combined with a forward-looking approach to urban greening to build cities that are more resilient, sustainable, and equitable
Li et al.[34]	environmental justice in NbS implementation	identified key challenges and offered recommendations for NbS use in managing UHI, flooding, wildfire, COVID-19, and air pollution
Muñoz and Duarte[35]	urban planning tools to expand GI in public spaces	analyzed 126 global strategies leveraging GI to address extreme climate change events
Sobhaninia et al.[36]	optimal location of GI to mitigate UHI and manage stormwater	assessed 8 GI types, integrating environmental, social, and economic factors to support informed placement decisions
Khalili et al.[37]	methods for evaluating urban GI benefits	reviewed monitoring, remorse sensing, and modeling approaches assessing GI's impact on heat regulation, human thermal experience, and air pollution; identified strengths, limitations, and key parameters of each method
Kumar et al.[5]	overheating in urban areas and role of GBGI	examined 51 types of GBGI to understand their effectiveness in reducing urban heating
Kumar et al.[4]	air pollution mitigation and GBGI	assessed the air quality benefits of 51 GBGI types across urban environments
Perera et al.[38]	GBI policy framework in 12 global cities	highlighted the emphasis on vegetation cover in policies and the need for GBI policies alignment at state and local government levels
Przestrzelska et al.[39]	GBI in rainwater management	revealed GBI's limited applicability in diverse climates and research bias toward high-GDP countries
Tate et al.[40]	economic evaluation of GBI interventions	revealed a lack of stakeholders' involvement and underrepresentation of studies from low-income and emerging economies
Debele et al.[41]	global role of NbSs in mitigating natural hazards	consolidated and analyzed NbS case studies worldwide; showing effectiveness in reducing natural hazard and climate changes
de Quadros and Mizgier[42]	GI strategies for pedestrian thermal comfort	identified street trees, green walls, and green spaces as effective cooling tools; green roofs had minimal pedestrian-level impact
Li and Lange[43]	GBI and stress resilience	explored links between urban landscapes with green cover (gardens, parks, wetlands, corridors, rivers, canals) and stress responses
Potter et al.[44]	health benefits of GBGI exposure	confirmed positive health outcomes from GBGI, although mechanisms remain insufficiently understood
Adnan et al.[45]	heat vulnerability and mitigation in Australia	highlighted the usefulness of GI and water-conscious urban planning in reducing heat-related risks
Evans et al.[46]	ES from urban agriculture and GI	demonstrated that community gardens, green spaces, parks, and allotments provide a wide array (16+) of ES

(*Continued on next page*)

Table 1. *Continued*

Author (year)	Key focus area of review	What was covered
Jones et al.[6]	ES, trade-offs, and synergies among urban GI	provided a new typology of GI, and reviewed the literature to create a matrix of GI × ES delivery to inform planning and illustrate synergies and trade-offs for environmental and social outcomes
Almaaitah et al.[47]	GBI's dual role in UHI mitigation and stormwater management	found strong evidence for stormwater benefits, with fewer studies focused on UHI mitigation effectiveness
Choi et al.[48]	co-benefits and trade-off for different GI types	found GI strategies primarily focused on climate adaptation, with limited attention to socio-cultural benefits
Kumar et al.[49]	monitoring methods for NbS performance against natural hazards	analyzed NbS monitoring methods and instruments to assess their effectiveness and challenges in addressing droughts, heatwaves, floods, landslides, storm surges, and coastal land loss
Kumar et al.[50]	modeling approaches for NbS efficiency	assessed hydrological and hazard modeling methods for evaluating NbSs, outlining benefits and data limitation. Highlighted the necessity to develop multi-scale process-based models to better assessment NbS benefits
Toxopeus and Polzin[51]	financing challenges and solutions for NbS (parks, trees, allotment gardens, and GBI)	identified key funding barriers and proposed strategies to improve benefit valuation and public-private investment balance
Veerkamp et al.[52]	GBI and ES delivery (local temperature regulation, stormwater management, waste processing, air pollution control, pollination services, and recreational and esthetic benefits)	emphasized gaps in ES and GBI coverage; most studies focused on temperature regulation and esthetics, often in parks or unspecified green spaces
Kumar et al.[53]	operationalizing NbS for hazard mitigation	analyzed the European policy frameworks applicable to hydro-meteorological hazards for NbS in policy and proposed NbS planning with focus on co-benefits and co-designed
Shah et al.[54]	frameworks and indicators for hydro-meteorological risk in NbS	suggested a framework for assessing vulnerability and risk within the scope of NbSs. Critiqued existing hazard and risk assessment indicators, calling for more inclusive NbS relevant metrics
Ying et al.[55]	strategic GI implementation	described GI as a multidisciplinary utility for delivering environmental and socioeconomic benefits simultaneously, with Europe and US leading in GI research
Debele et al.[56]	revised NbS concepts and classification of hydro-meteorological hazards	examined the impacts of hydro-meteorological risks (HMHs) in Europe and explores how NbSs can strengthen resilience, reduce adverse effects of HMHs, and support environmental sustainability
Ruan et al.[57]	understanding the positive and negative impacts of GI on the food-water-energy nexus	developed a framework to characterize the role of GI in sustaining food-water-energy nexus
Meng et al.[58]	quantification of the food-water-energy nexus in urban GBI	highlighted that most GBGI studies examine isolated benefits or life cycle impacts, while neglecting transboundary effects
Bellezoni et al.[59]	understanding how urban GBI influences the food, water, and energy nexus	established need for policies and research to shift from isolated to integrated approaches to fully connect GBI for sustainable urban futures
Venkataramanan et al.[60]	health and well-being outcomes of GI for water management	found limited evidence on human health impact; emphasized the need for community support and maintenance of GI
O'Brien et al.[61]	cultural ES of urban GI	classified well-being outcomes form 7 GI types into capability, experiences, and identities

reinforce how GBI enriches community identity[21] and supports environmental education.[22] However, inequities in access, limited community engagement and adoption, and culturally disconnected design practices reveal that GBI often struggles to resonate with diverse urban social realities. Embedding GBI in inclusive, locally grounded processes is therefore essential, as urban greening is gradually shifting from a purely ecological intervention toward a more socially embedded infrastructure for cultural resilience, social equity, and collective learning.[23]

As climate challenges intensify, GBI has become central to risk mitigation strategies and urban resilience frameworks. The UN SDGs call for increased investment in NbSs, with GBI supporting multiple goals: health and environment (SDG3: Good health and well-being; SDG15: Life on land), urban development (SDG11: Sustainable cities and communities), resource management (SDG2: Zero hunger; SDG6: Clean water and sanitation; SDG12: Responsible consumption and production), energy systems (SDG7: Affordable and clean energy), resilient infrastructure (SDG9: Industry, innovation and infrastructure), and climate response (SDG13: Climate action; SDG14: Life below water). This integrated approach demonstrates GBI's synergistic advancement of sustainable development priorities (see Figure S2). Responding to these imperatives, the IUCN developed the Global Standard for NbSs to provide a framework for integrating ecological, social, and economic goals.[17] In Europe, the EU Green Infrastructure Strategy[24] and the EU Biodiversity Strategy for 2030[25] advocate for coordinated, cross-sectoral greening policies to mitigate air quality, urban heat, flood risks, and biodiversity loss. The latter requires all European cities with populations over 20,000 to develop Urban Greening Plans by 2030. These strategies reflect a widespread policy shift toward evidence-based approaches and multifunctional GBI design. Nevertheless, existing planning practices and academic approaches remain fragmented, with limited integration of climate risk, equity, and co-benefits in GI decision-making. Robust, spatially explicit methods are urgently needed to optimize GBI placement for maximizing environmental and social outcomes[26] as well as to address such conflicts.

A wide range of recent literature has synthesized GBI and NbSs from diverse disciplinary perspectives (Table 1). Table 1 summarizes reviews (2017–2025) highlighting their role in addressing interconnected urban challenges. These studies have predominantly focused on GBI's capacity to mitigate environmental risks in urban areas, such as urban heat island (UHI), air pollution, flooding, and climate-induced stress.[4–6,62] Some

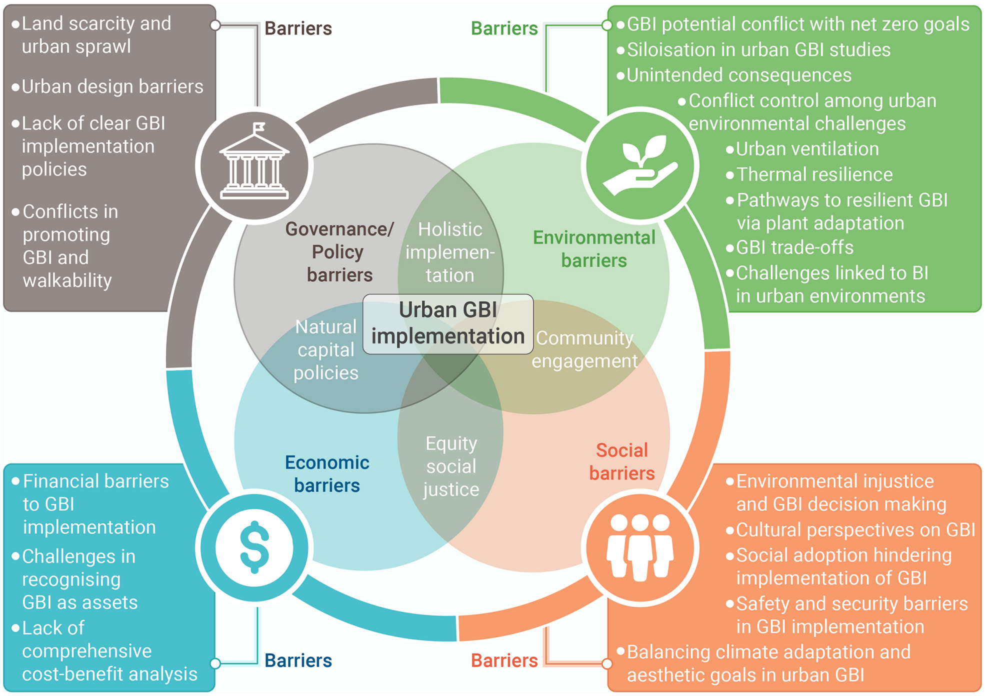

Figure 1. The topic areas covered in this review demonstrate a sustainability framework encompassing environmental, social, economic, and governance barriers hindering GBI implementation

reviews explored optimal GBI placement strategies,[33,36] environmental justice (EJ) implications,[34] and planning instruments for public green space provision.[35] A significant share of these studies focuses on GBI's environmental and climate-regulating functions, including stormwater management[47] and its contribution to hazard reduction and ecosystem service (ES) delivery.[41,52] Few have emphasized the contribution of GI on human health,[44] reducing thermal discomfort,[42] and improving mental resilience,[43] and reducing vulnerability to heat.[45] Others assessed the ES delivered by specific GBIs, such as green roofs, street trees, or urban agriculture, and identified regional disparities in implementation, particularly the underrepresentation of developing countries in both practice and research.[39,40] Recent reviews also advanced methodological frameworks to assess GBI effectiveness in reducing hydro-meteorological hazards,[63,64] proposed standardized evaluation tools,[65,66] and revealed gaps in monitoring, stakeholder engagement, and integration with gray infrastructure.[35,37]

Institutional constraints, competing uses of limited land, and fragmented policies can limit the implementation of GBI at scale. Gaps remain in research on ES,[61] inclusive governance,[34] and the socio-spatial distribution of benefits, particularly in developing countries.[40] Methodological reviews further identify the need for improved modeling, monitoring, and evaluation tools.[37,50] GBI implementation remains constrained by fragmented approaches and narrow technical focus, with deeper integration of cultural, social, and policy dimensions still lacking.[67] Overlooked issues include competing land use, inadequate or absent financing mechanisms, fragmented NbS policies, and unintended environmental consequences such as pollen, biogenic volatile organic compounds (bVOCs), and trade-offs with other urban agendas, such as net zero energy goals or car-centric urban design. Moreover, extreme climatic events, such as heatwaves, intense rainfall, prolonged droughts, and severe storms, may themselves constitute substantial barriers to its implementation and sustained maintenance.[68]

This review shifts focus from GBI benefits to underexplored barriers impeding its urban implementation. The comprehensive cross-disciplinary synthesis bridges fragmented knowledge by integrating perspectives from urban planning, ecology, climate science, economics, and social equity research. It creates a cohesive framework that analyses critical interconnections with energy efficiency, walkability, climate resilience, EJ, and competing land uses, concluding with actionable recommendations for more resilient and socially embedded GBI strategies. While some barriers (e.g., bVOCs, EJ, governance fragmentation) are well documented in the literature, they are typically examined in isolation and remain weakly embedded in planning standards, valuation methods, and governance processes. The term "overlooked" reflects this gap between existing knowledge and systematic application, positioning this review as a crucial integrative contribution that connects disparate research domains with practical implementation.

The overarching goal of this integrative review is to synthesize critical dimensions of GBI implementation that remain underexplored in the current literature. Specifically, the review develops a unified framework examining: (1) environmental barriers, including technical and system integration and strategic alignment challenges (GBI net zero conflicts, research siloization), environmental and ecological performance limitations (unintended consequences such as bVOC emissions), and climate management complexities (ventilation impacts, thermal resilience, plant adaptation, GBI trade-off, and BI); (2) social barriers, encompassing environmental injustice, cultural disconnection, adoption challenges, safety concerns, and esthetic controversy; (3) economic barriers, involving financial undervaluation of biodiversity, asset recognition issues in accounting frameworks, cost-benefit analysis (CBA) limitations, and investment barriers; and (4) governance/policy barriers, comprising land and space constraints, urban design barriers, policy fragmentation, integration challenges with other urban systems, and regulatory gaps. By bridging these previously siloed knowledge domains, this review provides key conclusions and actionable recommendations to support more holistic and effective decision-making in GBI implementation.

SCOPE, METHODS, AND OUTLINE

The scope of this review is confined to underexplored barriers that hinder the implementation of GBI in urban areas. Monitoring and modeling, health impact assessment, and multi-benefit analyses methodologies of GBI interventions lie outside this review's scope. For comprehensive coverage of these aspects, readers are directed to the key resources summarized in Table 1.

Barriers were identified and collated through a series of co-design workshops, involving numerous international experts in the field,[69] with a wider writing team involved in reviewing each topic, and informed by prior research.[70] The co-design process for barrier identification involved a multi-stage approach: (1) initial identification through a large interdisciplinary workshop, (2) independent refinement by a smaller multidisciplinary expert group workshop, and (3) finalization via full-author iteration to ensure a balanced, non-redundant list of overlooked barriers. Further details are provided in section S1. As illustrated in Figure 1, barriers were organized using a sustainability framework,[71] across four domains: environmental, social, economic, and the cross-cutting theme of governance and policy barriers that impede GBI implementation.

Literature search in Web of Science and Scopus databases was conducted, using structured search terms covering the challenge, and relevant to each topic section. The search term used included "Challenge AND Implementation AND ('Green infrastructur' OR 'Blue infrastructure') AND Urban." In addition, combinations of terms such as "Barrier OR Constraint AND (Green infrastructure OR Blue infrastructure) AND Implementation AND Urban and Obstacle AND Adoption AND ('Nature-based solutions' OR 'Ecosystem services') AND City" were also tested to ensure thematic breadth. Separate searches were conducted for each barrier domain (environmental, social, economic, governance/policy), with domain-specific keywords (e.g., "air quality," "heat stress," "public acceptance," "financing," "policy integration") combined with the core GBI terms, yielding ~29,000 results (Figures S2 and S3). Figure S2 illustrates the distribution of GBI publications across different SDGs, aligned SDG11 and SDG13 showing the highest share of publications. Figure S3 shows the year-wise

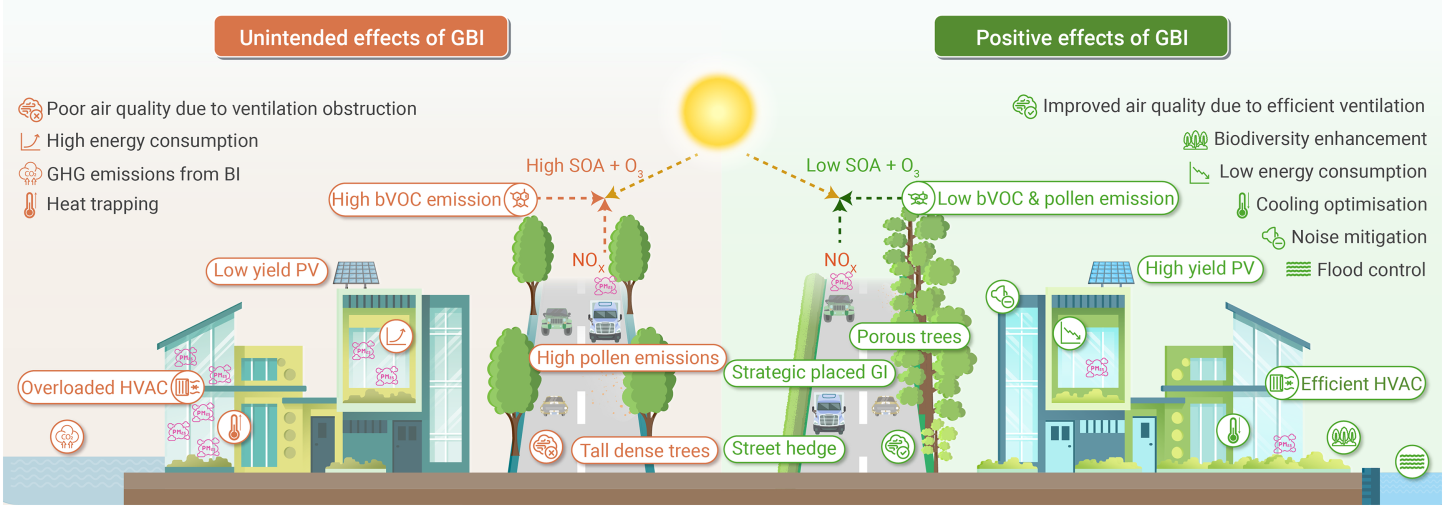

Figure 2. Contrasting potential outcomes of GBI at streets and household scale in an urban setting: unintended negative effects (left, red font) versus positive environmental benefits from strategic implementation (right, green font)

publication trends. First duplicates were removed. Title and abstract screening excluded irrelevant publications: non-urban settings, purely technical hydrological studies without GBI focus, and unrelated engineering fields. Full-text screening applied inclusion criteria: urban relevance, explicit barrier discussion, English language, and peer-reviewed status. A total of 577 studies met criteria and formed the synthesis evidence base, supplemented by authors' expertise and cross-referencing.

Following the introduction and methods, core sections analyze GBI implementation barriers: environmental, social, economic, and governance/policy. The last section presents conclusions, recommendations, and research gaps.

ENVIRONMENTAL BARRIERS

GBI has both positive and negative impact for mitigation of environmental problems at households and street scale as shown in Figure 2. To effectively address these contradictory impacts and optimize GBI functionality, nine environmental barriers (see GBI potential conflict with net zero goals to challenges linked to BI in urban environments) were identified that currently undermine successful implementation in urban contexts (Table 2). Table S1 provides a detailed summary of the case studies discussed below.

GBI potential conflict with net zero goals

While net zero and GBI initiatives share many common goals, including climate resilience efforts, renewable electricity generation can compromise GBI initiatives (Table S2). Declining costs of solar photovoltaic (PV) systems have accelerated their global adoption as the fastest growing renewable energy source.[72] Despite their benefits, PV installations may compete with existing land uses, including wetlands, parks, and forests, which can result in biodiversity and habitat loss.[73–75] For example, in Australia, homeowners with rooftop PVs are advised by local solar companies to reduce tree shade to maximize energy output.[76] PV performance is indeed affected by tree shade, which varies with species, height, and crown width.[77] Such studies are often used to justify tree pruning or removal when shading PV panels, with 30 US states permitting vegetation removal[78] despite trees' carbon absorption[79] and greenhouse gas (GHG) reduction benefits.[80] A German study showed that trees only reduce solar roof radiation by 1% after 20 years of growth,[81] although some argue PV systems provide greater carbon reduction than trees.[82]

Achieving a truly sustainable development rather than merely net zero development requires more than prioritizing PV over GI based solely on GHG reduction considerations.[83] The multiple benefits of a GI (Table 1) need to be considered when balancing against the benefits of PV (Figure S4). Therefore, the questions are: how can we achieve a net zero energy development that is also green? How can urban planners, city councils, and homeowners decide which GI elements to implement, including which tree species to plant and where, while maximizing solar energy production?

GBI reduces urban heat and provides cooling effects, thereby lowering building energy demand. Simulations in representative neighborhoods show that tree shading can reduce annual cooling needs by 2% under today's climate and by 5% in projected 2050 conditions,[84] which may also reduce the required size of solar panels. In Montreal, a case study for a building demonstrates that urban trees alone can mitigate 17% of carbon emissions over a period of 60 years, without even considering on-site electricity generation.[85]

Strategic integration of GI with on-site solar power requires careful planning of tree species selection, placement, and PV positioning. In England, for example, solar farms are predominantly sited in human-modified landscapes, including in urban settings, instead of in sensitive ecological areas and designated conservation sites, indicating environmentally considerate placement.[86] A framework integrating rooftop PV with GI under current and future climate scenarios was developed using urban modeling, energy simulation, and carbon sequestration analysis.[84] Building-integrated photovoltaics with green roofs and facades offer mutual benefits: vegetation cools panels, improving efficiency and optimizing space, although performance depends on panel-plant distance, installation conditions, species selection, and microclimate.[87] In tropical climates, plants combined with polycrystalline PV modules increased efficiency by about 2% at a 15° inclination.[88] Similarly, systems using sedum plants delivered a 1.6% gain when panels were mounted at 1.01 m with a 3° south-facing inclination.[89] Green roofs increase solar installations yields by 5%–15% through reduced albedo.[90] GI-PV integration depends on context, requiring tailored solutions for local climate, vegetation, and building conditions.

Floating PV systems installed on human-made water bodies such as reservoirs and irrigation ponds offer opportunities for urban BI while maximizing land efficiency.[91] For example, Singapore has implemented urban floating solar farms, capable of offsetting more than 4,000 tons of CO_2 annually[92] demonstrating the potential for large-scale deployment within dense city environments. However, research on their integration into urban design remains scarce, and studies on their impacts on water quality and aquatic ecology are limited despite growing implementation.[93]

To achieve net zero developments, GBI, particularly trees, are often perceived as detrimental to the PV systems. However, solar developments can be achieved through thoughtful planning without sacrificing urban landscapes. The vegetation can provide cooling and even improve PV efficiency. Incorporating PV into ecological restoration of urban landscapes also offers promising opportunities, although further research is needed. Decision-makers should embrace technological innovation to maximize the co-benefits of PV and GBI integration.

Siloization in urban GBI studies

Scientific silos (or knowledge compartmentalization) relate to the organization of scientists into discrete communities with minimal interaction. Siloization can slow the propagation of scientific information and impede understanding of inter-relationships of complex systems.[94,95] The siloization of GBI research has been broadly contextualized as the tendency to respond to single-issue problems rather than exploring the multifunctional solutions provided by ES of GI.[6] Urban green corridors (GCs), defined as linear landscape features that allow

Table 2. Summary of environmental barriers, challenges, and potential solutions

Environmental barriers	Challenges	Overcoming challenges
Conflicts between GBI and net zero goals[a]	• PV competes for space with existing GBI<br>• tree shading affects PV performance<br>• PV and GBI net zero goals and other environmental benefits are poorly studied	• green roof and BI can be used as alternatives for PV placement<br>• GBI can reduce cooling energy demand, reducing some PV needs<br>• use modeling tools to evaluate carbon emission and sequestration potential for both GBI and PV
Siloization in urban GBI research and planning[b]	• decision-makers focus on single-issue problems rather than multifunctional solutions<br>• fragmentation of green spaces impacts biodiversity, local climate, energy consumption, and well-being<br>• green corridors studied mainly for biodiversity impacts, neglecting other benefits	• cross-disciplinary approaches and inclusive systems-based analysis<br>• ecological production function based modeling assessment is suitable to identify shortfalls for mitigation actions<br>• ES framework provides context for comprehensive assessment
GBI trade-offs[h]	• GBI benefits vary across spatiotemporal scales<br>• a lack of integrated tools to holistically assess ecosystem service	• identify effective links between GI types using real-time monitoring<br>• integrated modeling and remote sensing can address the trade-offs efficiently
Unintended consequences[c]	• certain vegetation emits bVOCs, leading to SA and ozone (O_3)<br>• wind-pollinated species increase respiratory issues with allergenic pollen<br>• dense vegetation can disrupt airflow, trapping pollutants<br>• GBI contributes to GHG emissions, reducing sequestration potential	• select species with low bVOC emissions, minimal allergenic traits, strong pollutant deposition<br>• location specific design with hedgerows as alternatives to tall canopies in street canyons<br>• periodic hydrological and soil management to limit GHG emissions
Plant adaptation and resilience in urban environments Pathway to resilient GBI via plant adaptation[g]	• urban environments push species beyond their realized niches<br>• literature on plant stress tolerance in urban environments is limited<br>• decision-making relies primarily on practitioners' expert judgment	• select plants based on phenotypic plasticity for resilience to stress such as heat and drought<br>• introduction of species from surrounding biomes that match urban niches<br>• integrate scientific data with tools support evidence-based practitioner decisions
BI integration challenges Challenges linked to BI in urban environments[i]	• BI presents dual character as benefits and risks<br>• space constraints and urbanization restrict implementation of blue spaces<br>• climate and geography limit BI options in arid or elevated regions	• design BI away from populous areas to avoid nighttime heat and maximize benefits<br>• implement participatory planning and community involvement to address the space issue<br>• restore rivers and daylight buried streams to reintegrate waterways
Achieving synergistic control[d]	• large street trees are beneficial to reduce UHI and noise but could restrict pollutant dispersion<br>• high GI evapotranspiration is beneficial to UHI mitigation but worsens water shortage in dry seasons<br>• BI is effective in UHI mitigation and water management but could release GHG and pollutant gases	• multifunctional planning that considers the needs of various urban challenges for synergistic management
Urban ventilation and air quality impacts[e]	• GBI can alter city breathability by increasing resistance to airflow, which restricts the dispersion of air pollution<br>• poorly planned GI may obstruct building heating, ventilation, and air conditioning inlets, worsening IAQ	• integrate microclimate models to mitigate UHI while minimizing airflow blocking<br>• use machine learning methods to understand the nexus between GBI, city, and building ventilation

(*Continued on next page*)

Table 2. *Continued*

Environmental barriers	Challenges	Overcoming challenges
Thermal resilience and microclimate[f]	• research focuses on cooling effects rather than adaptation dynamics<br>• most studies examine GBI effects mainly on general populations, neglecting vulnerable groups<br>• surface temperature alone inadequately represents microclimate conditions	• integration of environmental factors with human comfort considerations<br>• development of city-scale overheating risk warning systems with focused strategies for vulnerable groups<br>• adaptation of digital twins, remote sensing, and AI for analyzing microclimate heat mitigation strategy

Each barrier is discussed in detail in the following subsections, outlining key issues and mitigation strategies.
[a]See GBI potential conflict with net zero goals (Figure S1; Table S2).
[b]See siloization in urban GBI studies (Figure S5; Table S3).
[c]See unintended consequences (Figure S6; Table S4).
[d]See conflict control among urban environmental challenges (Figure S8).
[e]See urban ventilation (Table S5).
[f]See thermal resilience (Table S7).
[g]See pathways to resilient GBI via plant adaptation (Table S8).
[h]See GBI trade-offs.
[i]See challenges linked to BI in urban environments.

biological migration and energy exchange between large patches of ecological source areas,[96,97] provide an illustrative example. GCs are commonly proposed as a means to improve ecological connectivity,[96] counteracting the effect of green space fragmentation, which is occurring alongside the loss of these spaces during urbanization.[98] This trend is associated with development patterns, related to the intensification of infrastructure[99] as well as morphological decisions that are dependent on extensive transportation networks.[100] The resultant habitat fragmentation has well-established implications related to biodiversity loss, associated with the loss of habitat, migration routes, and species connectivity.[101] However, this type of fragmentation also adversely influences local climates (by increasing UHIs),[102] energy consumption patterns,[103] landscape esthetic quality (as revealed by negative correlation between fragmentation index and esthetic quality metrics),[104] human well-being (life satisfaction increases positively with vegetation and less soil sealing),[105] and blue landscape availability.[106] GCs can help mitigate all of these impacts.

Currently, much of the work on GCs has explored the important biodiversity impacts of connecting larger green spaces,[96] often excluding analysis of other impacts mentioned above. A bibliometric analysis map (Figure S5) shows that the main body of scientific research is focused on the relationships between GCs, biodiversity improvement, and habitats for flora and fauna. Life quality including recreation and accessibility issues was less studied. However, there are many thematic categories that are far less examined in the context of GC studies, e.g., carbon storage and thermal exposure reduction,[5] where GCs have been identified as one of the underrepresented GBI elements in heat mitigation studies. Moreover, a close examination of the interconnections between the thematic categories (i.e., the strength of their links) indicates that most of them were studied in silos (section S2): only five studies have examined more than three solutions (Table S3).

All five studies were published after 2018; two were conducted in China (Beijing[107] and Nanjing[108]), two in Cusco, Peru,[109,110] and one in Cagliari, Italy.[111] Three deployed some type of landscape analysis utilizing remote sensing datasets (e.g., Land Use maps, Land Cover maps, Land Surface Temperature), while two studies were based on literature and online tools.[109,110] In two studies,[108,111] energy flow patterns and species along GCs were examined using least-cost path analysis and InVEST software to characterize wildlife movement resistance. The first of these five studies tackled siloization through ES concept application, assessing water storage/purification, local climate regulation, and esthetics before and after construction of a network of artificial lakes, wetlands, and parks along Beijing's Yongding River.[107] Ecological production functions identified shortfalls in ES production using mathematical models[112] to connect ecosystems, stressors, and management actions. Coupling green and built infrastructure can achieve desired multifunctionality, where inadequate ecosystem function exists. These studies demonstrate how cross-disciplinary approaches allow systems-based analysis of GC enhancement measures, revealing complementary or unintended consequences.

More integrative approaches to GI impacts are essential, especially for GCs. Single metric performance assessment undermines their manifold benefits; ES frameworks provide the required context for comprehensive evaluation.

Unintended consequences

GBI is gaining prominence for addressing urban challenges such as air pollution, heat, and biodiversity loss; however, poorly designed GBI interventions can cause unintended negative impacts.[4,113] Certain vegetation types emit bVOCs, leading to salicylic acid (SA) and O_3 formation when interacting with urban pollutants, such as NO_x,[113] while allergenic pollen can trigger respiratory illnesses.[114,115] Dense vegetation can also disrupt airflow (see urban ventilation)[116,117] and some green systems may emit GHGs such as methane (CH_4) or nighttime CO_2, lowering the sequestration potential (Table S4).[118,119]

bVOC emissions vary by species and stress, with high emitters such as *Populus tremula* increasing O_3 risks, while low emitters such as *Acer campestre* may help reduce it when combined with high deposition traits.[120,121] Stressors, such as heat, drought, and pruning can boost emissions by three to five times,[113,122] and since reactive sesquiterpenes drive SA formation,[123] choosing and maintaining low-emitting species is imperative.[4,124]

Urban GI, particularly wind-pollinated species, increase respiratory issues, with urban residents facing up to 20% higher allergy rates than rural populations.[114,125] Air pollutants increase pollen allergenicity,[126] for example, *Betula pendula* produces more potent allergens under polluted conditions.[127] Climate change intensifies pollen seasons with emissions increasing by 200%.[128] Urban planners in cities such as Sacramento, USA, London, UK, and Christchurch, New Zealand, favor often male dioecious trees (e.g., male-deodar cedars) to avoid seed or fruit litter, creating allergenic, monodominant canopies.[115,129–131] These patterns increase public exposure, particularly for vulnerable groups, while synergistic effects between pollen and pollution amplify health impacts.[114,127] Despite GBI's promotion, allergen-related health risks are often ignored, exposing gaps in species selection and risk assessment.[115]

As discussed in urban ventilation, planting layout matters: at the ground level in street canyons, tall and dense tree canopies can obstruct vertical ventilation, leading to elevated levels of air pollutants.[116,117,132,133] However, vegetation with optimized porosity and leaf area density can enhance mixing and filtration, particularly in open-road settings or with well-designed barriers.[117] Symmetrical rows can hinder airflow, while staggered or one-sided designs may better support noise reduction,[134] ventilation, and pollutant capture.[116] Yet, urban planning often neglects these design nuances, increasing air quality risks in dense, high-traffic zones.[133]

As highlighted in pathways to resilient GBI via plant adaptation, water management and poorly designed systems can cause significant GHG emissions, especially in urban wetlands and irrigated green spaces.[118,119] CH_4 fluxes in constructed wetlands can be up to 10 times higher than in natural ones, and CO_2 accumulation is common in poorly ventilated areas, particularly at night.[135,136] Nutrient runoff further exacerbates emissions, yet these disservices are often overlooked in GBI assessments.[117,137] Additionally, urban vegetation's

actual carbon sequestration is limited by plant species and size, site, and maintenance factors, which may offset expected benefits.[138–140]

Despite potential unintended impacts, GBI remains a powerful tool for urban resilience when informed by environmental evidence and local context (Figure S6).[120] A key mitigation strategy is selecting species with low bVOC emissions, minimal allergenic traits, and strong pollutant deposition capacity. Tools such as OPALS (Ogren Plant Allergy Scale) and regional emission inventories support the selection of insect-pollinated, female, or low-allergenic species near sensitive populations, while also boosting biodiversity by providing habitat and resources for pollinators.[115,125,127] Small, rough, and complex leaves further enhance pollutant capture without hindering airflow.[116,120]

Site-specific vegetation design is also critical. In street canyons, hedgerows offer an alternative to tall canopies, improving airflow and pollutant trapping.[116,133] In open areas, wide, dense and optimal porous barriers are effective to balance filtration and air movement.[120,141] Modeling tools can be used here to support GI design decisions under varying conditions in urban environments.[133]

Hydrological and soil management is equally important. Rain gardens, bioswales, and wetlands should avoid prolonged saturation to limit CH_4 emissions from anaerobic activity.[118,119] Over-irrigation and nutrient-rich runoff can be reduced with well-drained soils, plants adapted to wet-dry cycles, and reduced fertilization. Modeling helps optimize performance across environmental scenarios,[37,49] enabling GBI to achieve environmental and health goals while minimizing trade-offs.

As further discussed in conflict control among urban environmental challenges, these unintended impacts often manifest as conflicts among urban environmental challenges. Moreover, GBI trade-offs represents broader trade-offs and planning barriers that hinder integrated urban solutions.

Conflict control among urban environmental challenges

GI implementation in dense, and socially complex urban areas is often hindered by fragmented planning processes and mono-functional design strategies that focus on single objectives while neglecting the complexity and interdependency of urban systems. This can lead to trade-offs, where interventions addressing one challenge may fail to solve or even exacerbate others.[5,142–144] As discussed in siloization in urban GBI studies and supported by various studies, this underscores the need for integrated, multifunctional planning that considers strategies for synergistic management.

Recent GBI research has shifted from biodiversity focus to applied strategies addressing climate adaptation, stormwater, air quality, UHI, public health, and EJ (see Figures S3 and S7). Figure S8 visualizes the multifaceted impacts of GBI on key urban challenges. The major barrier to achieving synergistic control arises from the mixed or even conflicting outcomes that GBI interventions can produce on specific urban challenges. This is particularly evident in the control of UHI, air pollution, and water management. GI can effectively mitigate UHI effect[145–149] and reduce anthropogenic noise,[150] with the scale of intervention playing a critical role. Large tree canopies and leaf area densities are particularly effective in achieving significant UHI and noise reductions. For example, a case study from Montreal, Canada, found that the noise level decreased with an increase in the mean volume of tree crowns and canopy.[151] However, these features often conflict with the requirements for efficient air ventilation and pollutant dispersion in street canyons (unintended consequences and urban ventilation), leading to deteriorated air quality at roadsides.[152–154] Further, while plants with high evapotranspiration rates are beneficial to UHI mitigation, they also require substantial irrigation[155] and result in significant water losses, particularly during dry seasons.[156,157] Similarly, BI is effective in UHI mitigation and water management,[52] but could also cause air quality issues due to degradation of organic matter, particularly when high sediment accumulation is present.[158–160]

The above control conflicts among urban challenges are rooted in their different requirements. While UHI and noise mitigation require large tree canopies and high leaf area densities, effective pollutant dispersion in street canyons typically requires open space. However, studies have shown that GI could also act as porous barriers to stop the dispersion of traffic emissions to roadside pedestrian breathing zones when designed appropriately.[161,162] Recent computational fluid dynamics analyses showed that the uneven distribution of trees in street canyons facilitate horizontal pollutant transport, offering a novel strategy for redirecting pollution hotspots away from densely populated areas.[163] Such synergistic design considerations can help preserve the UHI and noise mitigation benefits of GI while minimizing, or even reversing, its negative impacts on pollutant dispersion.

To resolve the conflicts between vegetation evapotranspiration for UHI mitigation and water scarcity, strategies should prioritize shading and high-albedo effects over evapotranspiration. Studies have reported that the combined solar and surface radiation can cause 10°C–20°C hotter than ambient air temperature.[164] Therefore, selecting native and drought-tolerant species that offer ample canopy cover and effective solar reflectance can provide UHI mitigation benefits while minimizing irrigation demands. Finally, the unintended GHG and pollutant emissions of BI during UHI mitigation can be effectively solved through stricter regulatory measures aimed at controlling eutrophication and reducing organic matter inputs.[165]

The synergistic management of various urban challenges using GBI requires systemic changes in planning, evaluation, and governance. Conflicts arising between key functions, such as large shade for UHI mitigation versus open space for pollutant dispersion, highlight the significance of integrated and holistic design strategies. Key principles for addressing these challenges include species selection and planting design for balanced/synergistic UHI and air pollution control, prioritization of shade and albedo over evapotranspiration for UHI mitigation, and effective sediment management in BI systems.

Urban ventilation

As highlighted in unintended consequences, GBI interacts with natural and mechanical processes influencing indoor-outdoor air exchange in the built environment.[4,5,166,167] Given the dynamic spatiotemporal variations that occur in cities, the GBI and urban form interactions, can impact the performance of natural ventilation or HVAC (heating, ventilation, and air conditioning) systems, which may also indirectly affect GBI health as it influences microclimatic conditions. This has been evidenced in different context and scales: from an individual building,[168,169] to essential urban infrastructure including underground transport tunnels,[170] and in city-scale ventilation (Tables S5 and S6).[171] The bi-directional relationship between these factors have collectively received limited attention due to the complexity of GBI-GI interactions.

HVAC systems in particular provide a vital service to infrastructure across cities[172] despite requiring substantial energy, and associated operational carbon emissions, providing thermal comfort and good indoor air quality (IAQ).[172,173] At the same time they dispel heat externally and contribute to the UHI effect, in turn creating a detrimental loop between the HVAC system's cooling load[174] and UHI,[175] which is further exacerbated by hotter climatic conditions.[176] In dense high-rise urban environments, the dispersion of emissions relies heavily on wind-driven ventilation, described as the "breathability" of a city. This relates to the resistance to air flow created by the features and design of the urban geometry.[177] The addition of GBI can alter city breathability where GI can increase the resistance to airflow while BI can create the opposite effect (see conflict control among urban environmental challenges).

Buildings also "breathe," inhaling via HVAC system inlets, open windows and doors, and exhaling via exhausts and other outlets. Where the ventilation of city streets has been positively or negatively impacted by the presence of GBI, this in turn impacts on building ventilation via increases or decreases in IAQ,[169] energy consumption,[174] and humidity levels.[178] Buildings with higher energy performances, sufficient air tightness, and efficient HVAC systems could see greater energy demands associated with increased ventilation rates to maintain acceptable IAQ.[179] Poorly planned GI obstructing the mechanical ventilation system's fresh air intake could lead to worsening IAQ if the supply of outdoor air is restricted. GI such as green walls and avenue planted trees have also been shown to restrict the vertical dispersion of air pollution.[152,180] Both studies have highlighted the potential negative impact on street level air quality, yet most exposure to air pollution takes place predominantly indoors, and ventilation of buildings often originates from roof-level fresh air intakes. Therefore, GBI has the potential to improve IAQ and reduce heat and humidity loads along the source-receptor pathway of the HVAC intake. This consideration is, however, consistently overlooked in building and city design.

Understanding the GBI-urban ventilation nexus can overcome potential challenges posed by GBI implementation. Where negative impacts are highlighted from GBI designs, local solutions can be created to address insufficient urban

ventilation. City ventilation can be substantially altered by modifying air flow patterns using urban design features,[181] machine learning methods optimizing new building designs in urban areas,[182] and GI.[4] Moreover, machine and deep learning methods using mapping techniques have a role in implementing optimal GBI solutions[33] and could be integrated with urban microclimate models to ensure UHI mitigation and minimize airflow restrictions.[183] Previous investigations have also highlighted that the design, orientation, and location of building fresh air intakes can be significantly impacted by street level emissions altering IAQ.[184,185] Green roofs significantly minimize NO_x, CO_2,[186] O_3,[187,188] and particulate matter concentrations[189] at roof-level, reducing pollutants entering building air intakes. HVAC filters on a Portland, USA, green roof removed up to 14% of incoming ozone depending on humidity conditions, although with higher microbial and VOC loadings than white roof filters.[187] Similarly, a lightweight extensive green roof on a school building in New Belgrade, Serbia, reduced ambient PM_{10}, $PM_{2.5}$, and PM_1 by up to 22.5%, 28%, and 31.8%, respectively, compared with a reference roof.[189] Future work should develop GBI frameworks integrating building and city ventilation concepts.

The GBI-urban ventilation nexus, particularly involving HVAC systems, remains overlooked in urban design and management. GBI design needs to be considered within its wider context to maximize co-benefits and avoid unintended negative impacts.

Thermal resilience

GBI is widely recognized for improving urban microclimates and has emerged as a vital strategy for improving cities' thermal stress tolerance against heat exposure risks driven by rising climate change and urbanization. The key categories driving the enhancement of thermal resilience are social, ecological, and technological domains.[190] Although many studies focused on individual aspects, lack of clarity regarding the interaction of humans with the urban environment during designing thermally comfortable adaptation spaces and developing strategies to improve thermal resilience.[191]

Existing studies have shown that the synergistic effect of GBI could lower surface and ambient air temperature reducing the UHI effect (Tables S6 and S7), which in turn enhances thermal comfort and reduces heat risk. Thermal adaptation in humans mainly involves three processes: physiological, psychological, and behavioral adaptations.[192] Thermal adaptation plays a key role in users' comfort perception in GBI environments,[193] while research predominantly focuses on cooling effects rather than adaptation dynamics in complex urban settings. Few studies have explored how physiological parameters respond to changing thermal conditions in GBI environments.[194] In practice, the Paris Oasis Schoolyard Programme transformed schoolyards into urban cool islands accessible to communities during heatwave days.[195]

Greening designs, plant species selection, and water body distribution may differentially impact overheating risks for vulnerable populations, particularly older people[196] and children.[197] Despite strong evidence of heat-related vulnerability in these demographics, current research predominantly examines GBI effects on general populations.[193,198,199]

To analyze the influence of GBI on thermal environments and human comfort, data are collected through three principal approaches: subjective questionnaires, direct environmental/physiological parameter measurements, and remote sensing techniques (Table S7). While questionnaires and direct measurements provide valuable insights, they often yield limited spatial information, undermining comprehensive analysis.[200] Remote sensing offers an advantage by providing spatial distribution data of GBI across multiple large areas[201] including surface temperatures through inversion techniques.[202] However, surface temperature alone inadequately represents complete microclimate conditions. Comprehensive assessment of GBI's thermal impact requires additional parameters such as humidity and wind speed, which remain challenging to obtain simultaneously through remote sensing.[201–203]

To overcome urban thermal resilience challenges, future GBI research and planning must shift from measuring cooling effectiveness to understanding and quantifying human thermal adaptation. A comprehensive strategy should integrate environmental factors with human comfort considerations to enhance adaptive capacity and resilience. Data collection from field studies on outdoor thermal comfort needs to extend to a wider range of demographic groups, including older people and children. Digital twins, remote sensing, and artificial intelligence can leverage integrated GBI datasets to model thermal environments and user needs, supporting sustainable urban planning through data-driven insights.[203,204] Developing city-scale overheating risk warning and mapping systems, for example, human-centric digital twins[205] that incorporate GBI information would enable proactive adaptation strategies, helping urban residents, particularly vulnerable populations, to avoid dangerous heat exposures while maximizing the effectiveness of implemented GBI solutions.

Future research on thermal resilience through GBI should advance three key areas: comprehensive human-urban interaction across diverse demographic groups, refined methods for evaluating heat exposure in GBI environments, and emerging AI-powered technologies for comprehensive environmental assessment and intervention solutions to mitigate urban heat risks.

Pathways to resilient GBI via plant adaptation

GBI solutions aim to adapt cities to climate change and protect citizens from physical and mental health impairments.[4,206] However, the GBI tools comprise living organisms subject to physiological and metabolic limitations from climate extremes.[207,208] The potential demise of GBI interventions is often overlooked in urban adaptation, which tends to ignore the fact that the same plants, animals, and other organisms are already showing signs of decline in natural ecosystems worldwide.[209–214]

Throughout evolutionary history, organisms have been selected to cope with, survive, and reproduce under heatwaves, droughts, floods, and other sources of acute and chronic stress.[215,216] These adaptations define a species' realized niche—the range of environmental conditions where they naturally occur.[217] While many organisms can thrive under a broader set of conditions—their fundamental niche[218]—all species have tolerance limits beyond which they cannot reproduce or survive. Urban environments frequently push species beyond their realized niches, yet the global success of cosmopolitan species in cities worldwide demonstrates species' remarkable resilience to diverse urban conditions.[219–221]

As the cornerstone of GBI, plants are the focus of this section.[218] Plants, as sessile organisms unable to seek shelter from environmental stress, rely on adaptive strategies.[222] The fitness of plants in urban environments depends on morphological and functional phenotypic plasticity and adaptability, influencing their resilience to stress.[223,224] Despite the importance of species suitability for GBI success, literature on plant stress tolerance in urban environments remains limited.[225] Consequently, decision-making continues to rely primarily on practitioners' empirical knowledge,[226] a valuable but insufficient approach for promoting GBI biodiversity and resilience. There is also a risk that GBI species selection for urban planting comes from a "recipe book" of species long used in urban settings, but which may no longer be suitable under rapidly changing climatic conditions.

Understanding plant morphological and physiological responses to urban conditions under climate change is essential for selecting species that effectively provide ES. Phenotypic plasticity—one genotype-driven ability to produce various phenotypic variations in reaction to environmental conditions[227,228]—offers significant advantages, allowing plants to fine-tune their form and function to cope with local urban challenges.[229] The plastic responses include thickened leaf cuticles, reduced leaf area and stomatal density, facultative shifts in photosynthetic metabolism, and hydraulic system adjustments that help plants cope with heat, drought, and high light intensity.[230–232] Some species display remarkable stress tolerance, such as *Tipuana tipu* (Leguminosae) trees in São Paulo, Brazil, which increased photosynthetic and growth rates during one of the city's worst recent droughts.[219] While adaptation represents another pathway, it typically requires multiple generations under selective pressure and is therefore less likely to occur within time frames relevant to urban environments,[233] except for species with short life cycles that already adapted to urban conditions.[234–239] Urban plantings are typically undertaken with standard stock material and there is little or no scope for natural genetic variation to influence future generations of plants.

Species migration represents a third pathway for organisms responding to stress.[240] Natural plant migration is slow, spanning decades or centuries, making it unlikely to keep pace with rapid urban environmental changes.[241] In urban contexts, humans act as migration agents by introducing species through GBI implementation. While plant selection has historically been dictated by cultural preferences, market interests, and landscape design trends, emerging approaches now focus on introducing species from surrounding biomes that

correspond ecologically to urban niches—a more efficient strategy for enhancing GBI resilience.[242]

Phenotypic plasticity, adaptation, and migration represent the three primary mechanisms by which species establish and occupy new niches (Table S8)—essential processes that must be incorporated into GBI planning and management to achieve both immediate benefits and sustained resilience.[243] This integration remains particularly challenging in cities located within highly biodiverse regions where species knowledge by urban decision-makers is limited. However, practical shortcuts can accelerate appropriate species selection for urban GBI niches. These include leveraging basic scientific information (species descriptions and identification keys) alongside ecological studies of successional processes and plant strategies, providing invaluable insights into species' capacity to tolerate current and future urban climate conditions.[244] Without utilizing at least one of these three pathways, species may face local extinction through landscape transformation, undermining GBI effectiveness.[243,245]

GBI trade-offs

GBI implementation often involves trade-offs (Figure S8) and additional barriers, multi-scalar, technical, and institutional, that prevent synergistic planning.

GBI benefits occur across spatiotemporal scales that often misalign with targeted environmental challenges.[26,246] Spatially, GBI is commonly deployed in fragmented urban areas, while essential ecological processes function at broader scales.[247,248] This spatial mismatch, coupled with weak integration into city-wide planning, limits effectiveness.[249] For instance, scattered green roofs or pocket parks provide local cooling but may not mitigate UHIs (see thermal resilience), and GBI in downstream flood zones can be ineffective without upstream coordination.[250,251] Temporally, GBI often delivers ES slower than urban crises unfold. Static assessments overlook temporal dynamics, compromising long-term evaluations.[252] Some benefits arise quickly (e.g., air quality, noise, cooling) than others (e.g., biodiversity).[117,253] Planning rarely accounts for time needed for full functionality (e.g., tree maturity, soil development), hindering co-benefit integration and political support. This calls for long-term, multi-scalar planning and vision.[246]

A lack of integrated tools to holistically assess ES synergies and trade-offs remains a key gap.[254] GBI planning often relies on siloed (see siloization in urban GBI studies), mono-functional approaches, lacking the interoperability to evaluate interconnected processes and trade-offs (e.g., hydrology, urban climate, pollutant dispersion, and thermal comfort).[255,256] This is compounded in cities with scarce or incompatible data. Even studies emphasizing multifunctionality tend to map priority areas for GBI interventions without accurately accounting for local needs.[257–259] For instance, selecting a GBI with broad co-benefits to target multifunctionality usually relies on generalized criteria and suitability analysis, lacking context-specific design.[257] This reliance on non-local assumptions may reduce relevance.[254] Recent work illustrates the value of context-specific design. Open-air urban farming roofs in Shenzhen, China, could produce up to 7.44 kg of vegetables per m^2 annually and reduce upstream energy and water footprints by a factor of 4.5.[260] This example highlights both the potential and the trade-offs of farming roofs, which depend on local conditions, resources, and governances. Without integrative metrics, GBI decisions are fragmented, hampering planning, and increasing unintended trade-offs.

A key response to scale mismatches is multi-scalar environmental planning aligning GBI interventions from the block and neighborhood levels to watershed and metropolitan scales, enhancing ecological connectivity and integrated risk management. GBI networks should combine regional strategies (e.g., greenbelts, GCs) with local solutions (e.g., rain gardens, street trees).[261,262] A blend of scales and configurations is needed to optimize co-benefits.[26,262] Addressing spatial fragmentation requires cross-sectoral and multilevel governance agreements.[263] Further research is needed to identify effective links between GBI types, supporting governance mechanisms, and resulting multifunctionality benefits.

Emerging planning frameworks use spatial multi-criteria methods to incorporate urban ES into spatial decision-making, combining environmental and social layers to rank priority intervention areas in cities and integrating equity considerations.[256,257,264] These approaches map spatially explicit trade-offs at multiple scales and generate priority areas based on high-resolution geospatial analysis. However, such decision-making approaches are only as good as the data used to underpin them.

Technology and modeling strategies can partially overcome data or information limitations. Affordable sensing technologies and IoT platforms (e.g., air quality monitors, soil moisture sensors, urban rain gauges) enable real-time GI monitoring, supporting adaptive management.[161,265–267] Biomonitoring with bioindicators (e.g., lichens, leaves, and microorganisms) can help infer pollutant loads and ecological health.[268–270] Tools such as InVEST quantify services (e.g., thermal regulation, runoff retention, carbon sequestration, noise mitigation) under various GBI scenarios.[271–274] Finally, integrating these with remote sensing, GIS, and environmental models, will support better GBI siting and selection.[67,256,275,276]

Addressing GBI implementation barriers demands resolving spatial-temporal scale mismatches through multilevel planning, integrated modeling, and high-resolution spatial data, including social and demographic data, to align localized interventions with broader ecological processes and long-term urban resilience objectives.

Challenges linked to BI in urban environments

Urban BI encompasses both natural and artificial water-related elements within city boundaries, such as canals, lakes, ponds, rivers, wetlands, seas, and constructed drainage features that mimic natural hydrological processes.[5,6,277,278] These water bodies support human well-being, flood mitigation, cooling, and recreational and cultural benefits in cities.[279–281] Existing literature on BI challenges and solutions remains limited, especially regarding coastal systems. Space constraints in densely built areas often restrict the implementation and expansion of BI to a greater extent than most GI.[282] Management challenges for BI include balancing flood risks, erosion control, and maintaining attractive, accessible spaces for urban dwellers.[278] Climate and geography often shape BI options; cities in arid regions tend to make use of smaller water features since water is scarce, often pumped from elsewhere, and evaporation rates are high. Cities on elevated terrain will tend to have smaller water bodies compared with urban centers in low lying catchments where large rivers and lakes are more common. Cities with river catchments crossing national borders face additional political complexities beyond typical GI challenges in terms of managing water (high and low) flows and water quality. Cities relying on groundwater face additional complexities as these systems often cross jurisdictions and have unclear boundaries.[26]

BI often presents a dual character as both a benefit and a risk. While rivers and seas enhance well-being through access and visual connection,[283] they also increase flood risks.[284] Steep walls and high flood defenses designed to reduce risk will also reduce direct accessibility for the public, along with the visual connection of the public with these natural features. In tropical areas, there can also be health risks from vector-transmitted illnesses, such as malaria, or dengue, where insect vectors use any pooled water for breeding, from plant pots up to reservoirs. Solutions must balance protection with public accessibility to maximize multifunctional benefits.

A range of solutions can improve the implementation of BI in urban settings. Stronger governance and better policy integration are necessary to prioritize BI benefits, requiring national, and international coordination to manage water flows and water quality.[285] At a local scale, participatory planning and close engagement with residents are often critical for successful long-term implementation of local BI projects, fostering local capacity, appreciation, and stewardship,[277] such as caring for a stretch of canal or urban stream.[286]

While sharing knowledge on best management practices, upskilling personnel, and improving understanding among stakeholders[287–289] are common solutions across multiple types of GBI, these need to be specific to management of water and BI since the technical challenges are much greater around issues such as managing risk of disease transmission, or alleviating flood risk. Sharing lessons learned and data from pilot and demonstration projects is also recommended.[290]

BI planning must consider local context and can leverage historical and cultural connections to water, as demonstrated by the revitalization of traditional urban irrigation canals in Spain.[291] River restoration enhances existing BI by reintegrating waterways into urban environments through techniques such as "daylighting" buried streams, creating self-sustaining systems that deliver ecological benefits, flood control, recreation, and social value.[292,293] In space-constrained coastal cities, offshore NbS and hybrid approaches combining

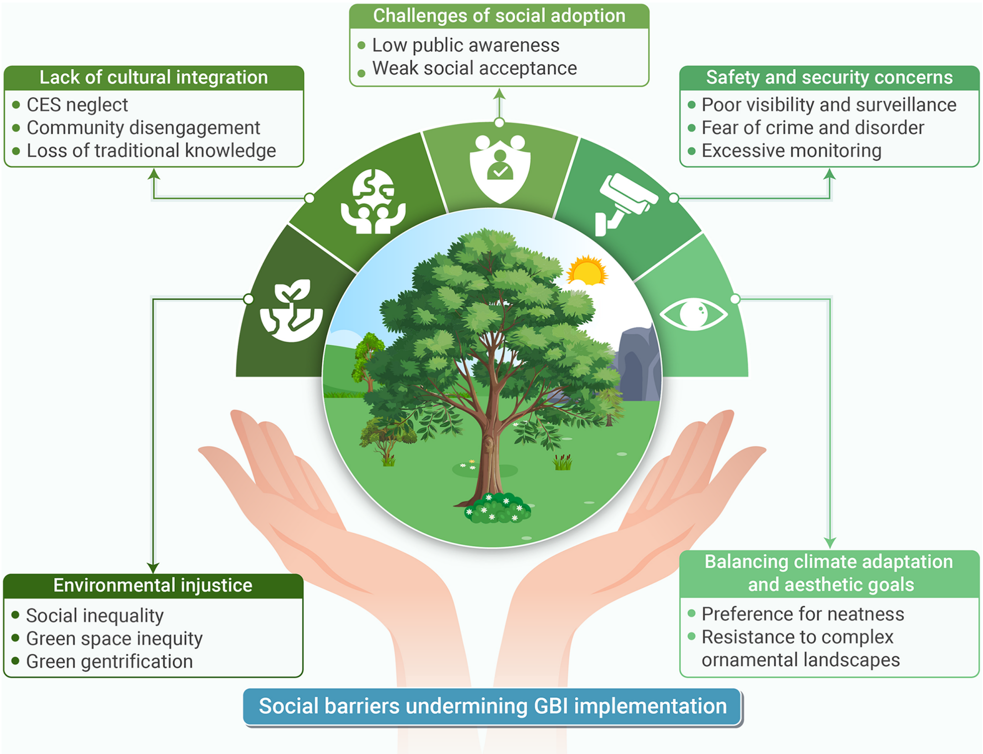

Figure 3. Five less-discussed social barriers that hinder effective urban GBI implementation These barriers include environmental injustice, cultural disconnect, social adoption challenges, safety concerns, and esthetic conflicts. Without inclusive and adaptive planning, they can undermine long-term GBI outcomes.

GBGI enhance resilience and multifunctionality, as exemplified by vegetated water retention basins that support biodiversity.[294–296]

Designing blue spaces with an emphasis on multifunctionality can maximize benefits, such as ecological connectivity, public space, and recreation.[278] Features such as fountains, and play areas with water sources create cooling and social hubs in urban spaces.[297] Improving physical and esthetic access to blue spaces, such as bathing platforms and waterfront walkways, along with better water quality, significantly boosts public engagement and the benefits that city dwellers experience from BI.[298,299]

In summary, addressing these issues for BI requires coordinated governance across scales, community engagement, and technical expertise sensitive to local contexts. Strategic approaches should include multifunctional design, natural water system restoration, and integration with other infrastructure types.

SOCIAL BARRIERS

This section synthesizes five key social barriers: environmental injustice, cultural misalignment, low public adoption, safety concerns, and esthetic resistance (Figure 3). A consolidated summary of their core challenges and corresponding strategies is presented in Table 3 and a summary of discussed case studies in Table S1.

Environmental injustice and GBI decision-making

Cities frequently exhibit significant social inequalities in wealth and access to resources, including uneven distribution of green spaces and the ES they provide.[300–302] Urban populations may be shaped by complex histories that include colonization, migration, and segregation along lines of race, ethnicity, religion, and caste, among others. These are further compounded by contemporary stressors that include increasing wealth inequality, the influx of refugees, and climate-related disasters. Consequently, the environmental trade-offs of green spaces (or lack thereof) in cities are not borne or enjoyed equally by everyone, resulting in systematic patterns of environmental injustice in most cities.[303–305] Historically marginalized groups disproportionately experience greater exposure to environmental hazards, including industrial and vehicular emissions and water contamination, noise pollution, rising heat risks,[306] floods,[307] and green space shortages.[308,309] Moreover, GBI projects in low-income communities often raise real estate prices, thereby pricing long-term residents out of their neighborhoods, a paradoxical phenomenon described as "green gentrification."[310–313] Consequently, the communities and urban areas with the greatest need for GBI investments are often the last to benefit. Equity in GBI implementation remains a key concern, as vulnerable communities often lack access to high-quality infrastructure.[314] Addressing this imbalance requires inclusive strategies, community co-creation, and investment in underserved neighborhoods. The implementation of GBI rarely integrates these equity dimensions, often limited by data and governance constraints.[315]

Persistent environmental disparities are rooted in historic patterns of racial segregation in the US, including the practice of "redlining" created by the Home Owners Loan Corporation to demarcate parts of a city into zones based on perceived loan default risk, with Black and Brown communities relegated to the least desirable red zone.[316,317] Redlining was officially enacted in hundreds of cities across the US from the 1930s to the 1960s, when the policy officially ended. Decades later, urban ecologists continue to find significant differences in tree canopy cover,[318] impervious surface area,[319] building density, air quality, summer temperatures,[320,321] and biodiversity,[322–324] with attendant disparities in public health outcomes.[325]

Cities in Europe and elsewhere have their own specific histories of unequal access for migrants and low-income communities, often with the similar result of low-income areas lacking adequate GBI,[326,327] and therefore experiencing UHI,[328] higher air pollution, lower biodiversity[329–333] and poorer health outcomes over time.[334]

In the Global South, extreme urban inequality can be found in informal settlements shaped by self-construction and social struggle from marginalized groups, seeking the right to housing and the city.[335] Over 1 billion people lived in urban informal settlements in the world in 2020, and, if urban poverty trends persist, these areas will continue to expand.[336] In these excluded spaces, resource optimization is essential, yet they are rarely prioritized in urban green policies.[337]

On the one hand, green policies can generate tension by promoting gentrification, and on the other, the growing trend of increasing socioeconomic disadvantage in urban areas, especially in emerging economies, reproduces urban territories of informality where GBI is difficult to implement due to spatial constraints in dense cities and vulnerability, marginalization, and the lack of basic infrastructure. In the scientific literature, most articles focus on green gentrification and racial inequalities in the implementation of GBI, particularly in cities within the Global North. Table S9 presents a selection of relevant studies on this topic.

GBI projects have the potential to address these environmental legacies of historic segregation and current marginalization, but only if planned and implemented in ways that prioritize the needs and voices of these communities.[338] At a minimum (Figure S9), participatory approaches and democratic governance are essential for ensuring that marginalized groups can participate in planning and governance to shape their neighborhoods.[339] Such approaches should also recognize that barriers to social adaptation and structural access may differ across communities and require context-specific solutions.[340] Some studies show that minority communities also report substantial benefit from local greenspace, and do not always feel marginalized, but only where they feel a sense of belonging.[341] Top-down policy that aims to build GBI in under-resourced areas must include EJ criteria in project design as well as opportunities for meaningful participation in decision-making processes from the target communities. Finally, GBI initiatives should integrate EJ frameworks in both

Table 3. Summary of social, economic, and governance barriers to GBI implementation, along with corresponding potential solutions

Barriers	Challenges	Overcoming challenges
**Social barriers**		
Environmental injustice[a]	• historical segregation and systemic inequalities<br>• unequal distribution of green spaces and ecosystem benefits<br>• green gentrification displacing vulnerable populations<br>• exclusion of marginalized groups from decision-making	• integrate justice frameworks into GBI planning and design<br>• prioritize investments in underserved communities<br>• enable early and meaningful community participation<br>• link local actions to broader multi-scalar governance
Cultural disconnection[b]	• under-recognition of the CESs<br>• exclusion of local and traditional knowledge<br>• poorly contextualized GBI undermining heritage<br>• ignoring culturally specific landscape preferences	• integrate CESs into GBI frameworks<br>• embed local and traditional knowledge into planning<br>• apply heritage-sensitive and culturally adaptive design<br>• foster co-creative public engagement
Public adoption[c]	• low public awareness of GBI benefits<br>• misalignment between expert planning and community needs<br>• lack of early participation weakening stewardship<br>• disconnection between technocratic approaches and local realities	• embed continuous community participation from early stages<br>• design GBI to enhance perceived safety and usability<br>• link GBI benefits visibly to daily life<br>• use environmental education to build trust and ownership
Safety concerns[d]	• vegetation creating surveillance blind spots<br>• obstructed visibility at entry points<br>• poorly monitored parks linked to antisocial behavior<br>• crime stigma reducing GBI use<br>• over-securitization limiting access	• apply crime prevention through environmental design (CPTED) principles<br>• use smart lighting, CCTV, and IoT monitoring discreetly<br>• promote participatory design and local stewardship<br>• balance safety needs with social inclusivity
Esthetic tensions[e]	• public preference for ornamental over functional landscapes<br>• functional GBI perceived as messy or neglected<br>• policies favor visual esthetics over ecological resilience	• apply "cues to care" strategies to improve esthetic acceptance<br>• foster esthetic literacy and public engagement<br>• integrate multifunctional and adaptive design approaches
**Economic barriers**		
Financial undervaluation[f]	• biodiversity undervalued<br>• private funding limited<br>• cost-effectiveness is hard to demonstrate<br>• weak environmental, social, and governance (ESG) metrics	• use green/blue bonds, debt-for-nature swaps<br>• strengthen ESG with AI and satellite data<br>• build resilient financial systems
Asset recognition issues[g]	• existing accounting standards only consider built infrastructures as assets<br>• local governments face financing limits<br>• co-benefits of natural assets are undervalued	• adopt environmental-economic accounting and ISO 55000 standards<br>• integrate GBI into policies<br>• create biodiversity and water credits<br>• reform asset accounting
Lack of comprehensive cost-benefit analysis[h]	• ecosystem services, biodiversity, and well-being are often excluded<br>• high upfront costs<br>• data gaps and inconsistency in valuation methods<br>• weak ESG metrics and lack of standardized evaluation methods	• use real-world proxies and life cycle assessments<br>• develop inclusive, standardized cost-benefit analysis<br>• collect localized, context-specific, stakeholder-driven data in both costs and benefits
**Governance/policy barriers**		
Land use and space constraints[i]	• high land competition in dense cities<br>• rising land values reduce feasibility<br>• fragmented green networks<br>• reliance on gray infrastructure systems	• promote adaptive, space-efficient GBI<br>• prioritize micro-scale interventions (e.g., green roofs, permeable pavements)<br>• strengthen land use regulations

(*Continued on next page*)

Table 3. *Continued*

Barriers	Challenges	Overcoming challenges
Urban design barrier[j]	• conflicting demands among land uses<br>• poor integration of ecological functions<br>• weak coordination across spatial scales	• apply holistic and 3D spatial planning<br>• design multifunctional and compatible GBI<br>• use policy incentives to support implementation
Policy fragmentation and regulatory gaps[k]	• lack of clear, binding GBI legislation<br>• institutional silos and poor coordination<br>• inconsistent environmental standards	• implement legal and regulatory reforms<br>• enhance interjurisdictional coordination<br>• support capacity building and training
Integration challenges[l]	• GBI not embedded in walkability or transport systems<br>• disjointed planning undermines synergies<br>• poor alignment with social and ecological priorities	• foster multi-scale and cross-sector planning<br>• strengthen participatory governance<br>• deploy spatial analysis tools (e.g., GIS) for integration

Each barrier listed is further elaborated in the relevant sections of this review, where the source and context of the challenges are discussed in detail.
[a]See environmental injustice and GBI decision-making (Table S9).
[b]See cultural perspectives on GBI (Table S10).
[c]See social adoption hindering implementation of GBI.
[d]See safety and security barriers in GBI implementation (Table S11).
[e]See balancing climate adaptation and aesthetic goals in urban GBI (Table S12).
[f]See financial barriers to GBI implementation.
[g]See challenges in recognizing GBI as assets.
[h]See lack of comprehensive cost-benefit analysis.
[i]See land scarcity and urban sprawl.
[j]See urban design barriers.
[k]See lack of clear GBI implementation policies.
[l]See conflicts in promoting GBI and walkability.

research and practice if we are to effectively disrupt the cycle of continued marginalization for these communities around the world.[342,343] This approach also requires a multi-scalar perspective linking local decision-making to broader governance mechanisms at city, regional, and national levels.

Environmental injustice perpetuates unequal access to GBI, frequently excluding marginalized communities from its benefits and decision-making processes. Without equity-driven planning, GBI implementation exacerbates existing risks and disparities. Integrating justice frameworks and ensuring inclusive participation is crucial to guarantee that GBI simultaneously advances sustainability and social equity.

Cultural perspectives on GBI

Although GBI is increasingly promoted, its cultural dimensions remain systematically under-addressed in both planning and implementation. A key issue is the persistent under-recognition of CESs, with limited attention to intangible values such as heritage, identity, and education.[118,344,345] Community and cultural engagement is often weak, as top-down planning processes frequently overlook local socio-cultural perspectives, leading to limited public acceptance.[346,347] Institutional procedures frequently privilege technical or esthetic criteria over place-specific cultural values, and indigenous knowledge systems, which offer valuable insights into sustainable land management, are also rarely incorporated into formal GBI strategies.[348]

In addition to these gaps, poorly contextualized interventions, such as inappropriate vertical greening, insensitive vegetation management, or the neglect of traditional spatial arrangements and layout principles can undermine both cultural heritage and microclimatic performance.[349–351] Moreover, culturally specific landscape preferences are often ignored, resulting in GBI designs that fail to resonate with the diverse social, esthetic, and spiritual meanings attributed to green spaces.[352,353] Together, these shortcomings reveal that neglecting cultural perspectives not only weakens public resonance but also undermines the long-term legitimacy of GBI. Table S10 summarizes case studies and reviews that reflect these challenges, revealing persistent gaps. Addressing these challenges requires a more inclusive, culturally grounded, and community-responsive approach to GBI.

Traditional and religious cultural contexts have demonstrated the capacity to enhance both ecological performance and cultural resonance. The Lingering garden in Suzhou, China, illustrates how culturally embedded landscapes can function as effective GBI.[354] Through the integration of water elements, dense plantings, and shading structures, these classical gardens passively regulate microclimates by enhancing thermal comfort and humidity, particularly in shaded or water-adjacent areas.[349] Similarly, the sacred Mughal garden of the Taj Mahal and the minimalist Zen garden of Ryoan-ji reflect how religious traditions have historically embedded GBI elements within spaces of ritual, reflection, and ecological value.[352] These precedents demonstrate that microclimates and cultural narratives are co-produced through spatial codes and symbolic cues. However, such practices remain largely confined to small spatial scales. Scaling them up to the neighborhood or urban level is often hindered by mismatches with modern urban layouts, rigid planning systems, and the complexity of cultural meanings.[295]

Poorly contextualized GBI interventions can undermine the cultural heritage they aim to support. For example, installing vertical greening on historical facades may increase moisture retention and cause masonry cracking, accelerating material deterioration.[350] In heritage sites, inadequate vegetation management may negatively affect microclimatic conditions, visitor experience, and the preservation of delicate historic structures.[351] In Malaysian heritage cities, adaptive reuse of historic buildings has sometimes caused cultural and structural harm when added greenery ignores original layouts or materials.[355] Collectively, these cases show that insufficient contextual sensitivity threatens both tangible heritage (materials, form) and intangible heritage (symbolism, identity).

Insufficient cultural sensitivity and weak integration of local values are key barriers to GBI success. When interventions neglect traditional knowledge, land practices, or community identity, they are often perceived as externally imposed, reducing public engagement and long-term stewardship. In Peru's Tumbes Basin, for example, indigenous fog-harvesting terraces have been excluded from formal planning processes, marginalizing culturally significant land management practices.[348] Christchurch's Wigram basin demonstrated poor recognition of cultural benefits due to limited indigenous consultation.[356] In Moscow's Gorky Park, while recreational use thrives, educational and heritage functions remain underrepresented.[357] These cases illustrate the consequences of undervalued CESs, sidelined traditional ecological knowledge, and heritage-insensitive programming.

To overcome cultural limitations in current GBI implementation, a 3-fold strategy can align design intent with place-specific cultural systems. First, integrating traditional ecological knowledge, such as local water systems, symbolic plantings, and spatial practices helps align GBI with place-specific values and deepens cultural relevance.[358] Second, heritage-compatible assessment should be embedded from concept to maintenance using tailored frameworks, such as vegetation risk indices or heritage-compatible green design principles minimizing physical and symbolic damage.[359] Third, public engagement can be strengthened through participatory processes that involve communities from early planning stages, particularly by embedding spaces for cultural rituals, storytelling, and place-based learning.[360] These measures facilitate convert cultural sensitivity from aspiration into verifiable practices.

Social adoption hindering implementation of GBI

Although urban GBI is widely promoted for its climate and ecological benefits,[361,362] its implementation is often hindered by limited social acceptance and engagement. The durability of GBI depends on whether residents and stakeholders perceive interventions as safe, useful, fair, and worth caring for over time. Factors such as public perceptions, cultural relevance, safety concerns, and historical injustices influence how residents interact with these interventions.[363,364] Social adoption depends on four interlinked dimensions: perceived benefits and risks (e.g., safety, usability), procedural fairness (inclusion and voice), distributional fairness (who gains/losses), and capacity for ongoing stewardship. Without adequate social alignment, even ecologically sound projects risk being underutilized or rejected.

Empirical research underscores the social value of GBI. Inclusive, accessible parks promote interaction, cohesion, and intercultural understanding.[365] Green space design that encourages informal interactions such as bench conversations or children's play, foster trust and belongingness.[366] In Beijing, centralized, high-quality green spaces were more effective in enhancing residents' community attachment than fragmented or inaccessible ones.[367] These findings indicate that design choices facilitating everyday sociability, clear access, and inclusive use strengthen place-based attachment and social adoption.

Despite growing insights, social barriers to GBI adoption persist due to entrenched perceptions and institutional norms.[368] While promoted for its multifunctionality, GBI often reflects planner-led priorities that are misaligned with local values and everyday practices.[363] Planners may prioritize cost-effectiveness or technical feasibility, while residents resist interventions that disrupt cultural ties or land uses.[70] This misalignment can weaken trust, leading to skepticism and reduced willingness to engage with or maintain interventions. Additionally, historical patterns of environmental injustice contribute to unequal access to urban nature, generating skepticism among marginalized groups regarding new GI interventions.[369] These dynamics reflect both distributional inequities (who gets what where), recognition voids (respecting local's values and norms) and procedural gaps (who decides how), which together weaken social license to operate. By contrast, where communities feel they belong in a place, the use of, and benefits from, local green spaces are plentiful.[341]

GBI interventions can trigger green gentrification (see environmental injustice and GBI decision-making), where environmental improvements displace vulnerable populations through increased property values.[370] Governance structures lacking inclusive participation[339] exacerbate these effects by limiting community's inputs.[371] This transforms GBI into a technocratic solution detached from community realities.[372] Mitigating these risks requires anti-displacement measures (affordable housing commitments, community land trusts, benefit-sharing agreements, local opportunities) to prevent environmental gains from causing social losses. From a social adoption perspective, gentrification pressures erode trust and diminish stewardship participation.

A key barrier to GBI adoption is the misalignment between planners' intentions and local communities' values. Projects often prioritize ecological or esthetic goals but overlook concerns about safety, usability, or cultural relevance.[347] In marginalized areas, residents may view dense vegetation or secluded spaces as unsafe (see safety and security barriers in GBI implementation).[373,374] Perceived safety depends on lighting, sightlines, visibility of guardianship, and maintenance quality. Neglect in these areas quickly undermines adoption of GBI.

Limited awareness about GBI functions restricts informed engagement,[70] while psychological barriers (low perceived self-efficacy, uncertainty about usage rules) suppress participation even when physical access exists. Without inclusive processes, communities remain disconnected from projects, weakening long-term stewardship.[70,361] Additionally, green spaces shaped by dominant cultural norms may neglect the needs of women, elderlies, or ethnic minorities, making them feel unwelcome.[375] These factors can cause the technocratic implementation to be disconnected from the social realities of GBI it seeks to serve.[372]

Enhancing social adoption of GBI requires embedding meaningful community engagement into planning and implementation.[339] Participatory approaches such as co-design, mapping, and citizen science increase interventions' legitimacy, relevance, and local ownership.[364,376] Effective engagement is early (agenda-setting, not just consultation), iterative (multiple feedback loops), and accessible (multilingual materials, varied meeting times, paid participation where appropriate). Citizen science initiatives offer unique opportunities to capture place-based knowledge and skills, empower residents, and generate contextually rich data that inform GBI decision-making. Previous research on elderly's perceptions suggest accessibility, safety, and usability, emphasizing the significance of green space availability and condition.[377] Moreover, urban greenery's contribution to supporting healthy aging reflections from elderly people highlights the significance of engaging them in recall assessments.[378] These practices enhance procedural justice and build shared ownership beyond the project phase. Similar perception-base assessments are needed at local level with different backgrounds of the people in a society such as women, children, marginalized communities, etc., for the fairness of GBI projects.

Ensuring equitable access to GBI is critical for fostering public trust and addressing spatial and social inequalities. Prioritizing GBI in underserved areas can address injustices and build community trust.[379,380] Linking these initiatives with local employment, education, or recreation generates co-benefits and strengthens public support.[381] Communication campaigns, school programs, and targeted outreach raise awareness of GBI's multifunctional benefits, from cooling to mental well-being.[382]

Institutional reform must support integrated, socially responsive planning through clear maintenance accountability, ring-fenced dedicated budgets for community partnerships, and metrics tracking inclusions (participation diversity, perceived safety, equitable usage, realized recognition). Cross-sectoral collaboration and adaptive governance can bridge ecological, technical, and social goals.[362,383,384] Formalizing agreements with park friend groups and schools converts episodic engagement into stable care networks, embedding GBI within the urban fabric for its wider social adoption.

While the ecological and infrastructural benefits of GBI are well established, its successful implementation depends on overcoming social adoption barriers. Misaligned priorities, lack of engagement, inequitable access, and institutional fragmentation hinder public support. Addressing these challenges requires inclusive planning, equity-focused investments, effective communication, and integrated governance. A socially grounded approach to GBI is key to building resilient, just, and livable urban futures.

Safety and security barriers in GBI implementation

Despite growing policy support, GBI implementation often faces barriers linked to safety and security concerns (Table S11). GBI can be perceived as both a resilience asset and a security concern.[385,386] Poorly designed or maintained green areas may enable concealment or illegal activities where surveillance is limited.[387,388] In Latin America and Sub-Saharan African regions, over half of urban planners have altered or canceled projects due to crime concerns.[389] Similarly, a 2023 UK survey revealed 44% of urban residents avoid local green spaces due to safety concerns, which increased to 63% in disadvantaged areas.[18] In Malmö, Sweden, residents' concerns about poorly lit parks prompted redesign with improved lighting and surveillance,[390] while London similarly redesigned small parks and paths to address drug use and antisocial behavior in unsupervised areas.[391]

Safety challenges occur across multiple spatial scales. On streets, dense vegetation can obstruct views and create blind spots, reducing passive surveillance.[386,387] In housing areas, green fences may block visibility and undermine defensible space principles.[392,393] In underserved neighborhoods, poorly monitored parks often attract antisocial behavior.[394,395] Unmonitored greenways and gardens can invite antisocial behavior and vandalism despite fostering community engagement.[396] In high-crime informal settlements, GBI faces resistance due to security concerns,[312,397] while paradoxically areas under constant surveillance may restrict access for underprivileged groups (Table S11).[13]

Recent studies incorporate safety directly into GBI planning through multiscale approaches that support climate adaptation while enhancing real and perceived safety.[4,5,398] These frameworks emphasize integrated planning, inter-agency cooperation, and the evaluation of indicators for security and wellbeing across social groups.[397] Multifunctionality indices and ES maps offer instruments to design socially safe.[255,399]

Crime prevention through environmental design (CPTED) complements these approaches by emphasizing natural surveillance, territorial reinforcement, and access control.[392,400] Interventions such as motion-activated lighting and scheduled patrols improve perceived safety in isolated greenways. Technology

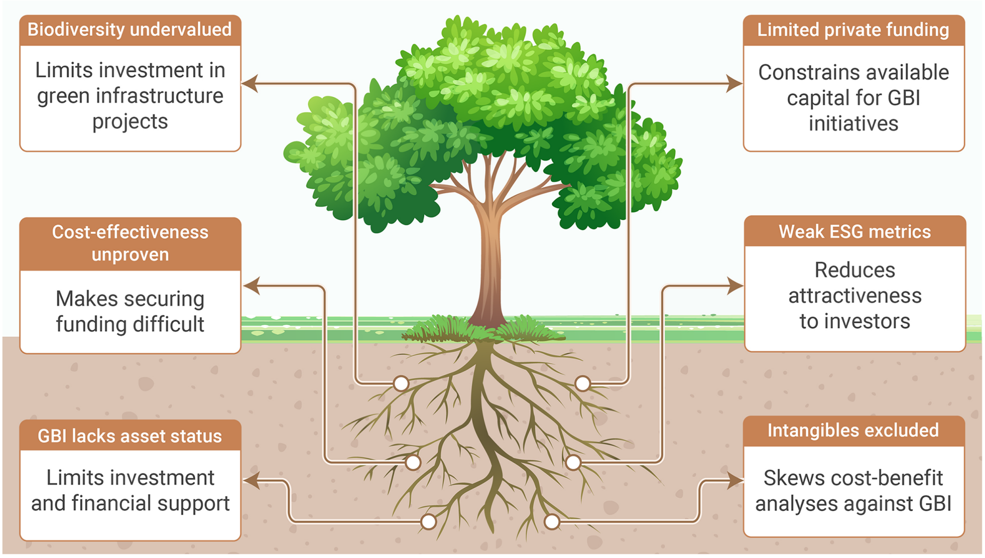

Figure 4. Schematic representation of the six financial challenges obstructing urban GBI implementation The illustration highlights how deeply embedded financial barriers can silently inhibit the growth and success of GBI initiatives. These barriers are further explored in the relevant sections of this review, where their origins, implications, and context-specific dynamics are discussed in detail.

enhances safety through smart lighting, CCTV, and IoT-based monitoring that preserve the landscape's visual character.[401,402]

Community participation in planning, maintenance, and oversight fosters ownership and reduces vandalism through informal social control.[403] Transforming neglected spaces into green spaces has been associated with reduced criminal activity and improved perceived safety.[404] Effective GBI design should be informed by local risk assessments, integrated CPTED principles, and promote community involvement to develop inclusive resilient GBI.

Safety and security remain critical yet underexplored dimensions in GBI implementation (see Table S11). Effective GBI design should: (1) be informed by local risk assessments to reflect area-specific needs, (2) integrate CPTED principles and monitoring technologies to enhance both real and perceived safety while preserving ecological value, and (3) promote community participation to strengthen social oversight and reduce misuse. Addressing these factors is essential for developing inclusive, resilient, and socially sustainable GBI systems.

Balancing climate adaptation and aesthetic goals in urban GBI

A tension between ecological functionality and prevailing esthetic preferences often challenges the implementation of GBI. While climate-resilient GBI prioritizes heterogeneous, function-driven, and ecologically complex design, urban planning and public perception have tended to favor tidy, ornamental landscapes.[405,406] Historically, urban esthetics have emphasized beautification and recreation, prioritizing lawns and formal gardens.[406,407] Increased awareness of climate change is driving a shift toward multifunctional landscapes guided by ecological principles and the provision of ES.[408,409] For example, in 2019, King's College Cambridge, UK, transformed its historic 1772 lawn into a wildflower meadow, reflecting a shift from formal turf to multifunctional landscapes.[410] Nevertheless, modernist esthetic norms continue to shape public expectations and institutional practices, often hindering the adoption of climate-adaptive, structurally diverse, and ecologically robust designs.[405,406] This misalignment between ecological needs and esthetic conventions limits GBI's transformative potential.

Past scholarly works highlight the multifunctionality of GBI, focusing on its ecological, social, and esthetic co-benefits.[399,411] However, recent evidence shows that esthetic priorities often precede ecological function in urban greening projects. For instance, street trees are often selected for their ornamental qualities rather than their ability to withstand drought or urban heat.[412] Although native or climate-resilient species are ecologically valuable, they are perceived as unkempt or undesirable, provoking resistance from communities and policymakers.[413] Esthetic preferences in urban landscapes are shaped by underlying socio-cultural norms and institutional frameworks.

Despite growing recognition of GBI's multifunctionality, challenges remain in aligning ecological resilience with public expectations of beauty. A key conflict lies between ecological design principles, such as heterogeneity and native vegetation, and societal preferences for neatness and ornamental species.[414,415] Functional GBI may appear disordered or untamed, often misinterpreted as neglect, especially in cultural contexts where tidy, manicured landscapes indicate care, safety, and social order.[405,406]

In addition, the lack of design elements that signal human intention and care can undermine public support for GBI projects (Table S12). This underlines the importance of integrating esthetic legibility into functional landscapes by carefully applying design principles grounded in social and cultural understanding.[407]

Institutional and governance frameworks often reinforce perceptual barriers; planning regulations, zoning codes, and funding mechanisms typically prioritize immediate visual appeal and public acceptance over long-term ecological performance and resilience.[415,416] In such contexts, climate-adaptive infrastructure is frequently marginalized, particularly without interdisciplinary collaboration.[417,418] Maintenance practices worsen this issue, as they are typically designed for conventional green spaces rather than complex, dynamic ecosystems requiring distinct expertise.[313]

Public interpretation of landscapes is strongly influenced by visible indicators of care, termed "cues to care."[406] These cues include trimmed edges, pathways, and signage that convey deliberate stewardship.[407] Strategically applied, cues can bridge the gap between ecological functionality and public appeal. Integrating intentional design into the scientific design process, referred to as the "design-in-science" approach, is critical for translating ecological theory into practice.[419] This framework underscores the importance of shaping spatial patterns by both ecological functions and their social meaning and visual clarity (Table S12).

Esthetics in climate-resilient urban design should apply integrative design principles from landscape ecology to guide planning, focusing on patch diversity, ecological corridors, and flows.[409] Embracing "careful messiness" is essential; it reframes wildness as intentional, promoting esthetic literacy and ecological understanding among planners and the public.[406] Urban planning norms must evolve by integrating adaptive principles into zoning regulations and development codes, thereby incentivizing multifunctional GBI.[409] Public education fosters public acceptance; targeted awareness campaigns and participatory design processes can help reconcile community preference with ecological priorities.[368,406,417] Finally, creating resilient and meaningful landscapes requires transdisciplinary collaboration, bringing together ecologists, landscape architects, urban planners, policymakers, and local communities.[409,418]

Many urban GBI projects still prioritize esthetics over climate resilience to contribute meaningfully to climate action. Public perception, policy, and limited funding often make it more challenging to adopt climate-resilient approaches. To truly future-proof cities and communities, GBI must integrate multifunctional, climate-adaptive designs that integrate both ecological and esthetic values. This will require a concerted effort that includes public education, participatory planning, policy reform, and the establishment of sustainable financing mechanisms to support resilient urban environments.

ECONOMIC BARRIERS

This section identifies five financial and institutional challenges that limit GBI implementation: financial undervaluation of biodiversity, limited private investment, weak environment, social, and governance (ESG) metrics, and the lack of recognition of natural systems as formal assets. These issues reduce funding opportunities, lower investor confidence, and restrict policy support. Figure 4

outlines these key barriers and how they are interconnected. Each challenge is provided a detailed discussion in the following sections and summarized in Table 3, and a summary of discussed case studies is presented in Table S1.

Financial barriers to GBI implementation

Over half of global GDP (or $44 trillion) depends on healthy biodiversity.[420–422] Yet, the current economic system consistently undervalues biodiversity and natural capital.[423] Traditional structures treat natural capital like other assets—financial, intellectual, and human—rather than recognizing the economy operates within, not alongside, nature.[423] The financial system is described as the "economy's circulatory system" and the large banking institutions as "the heart."[424,425] Today's financial system differs greatly from earlier generations, mainly due to technological innovation.

Financial and policy decisions over the past century have driven biodiversity loss and now threaten financial stability. Despite its systematic risk, the biodiversity and finance agenda remain underdeveloped due to gaps in appropriate regulation, data, methodologies, and understanding. The finance ecosystem includes not only traditional institutions (banks, insurers, asset managers), central banks, multilateral development banks, accounting firms, and regulators, but also the growing FinTech sector. While regulators are beginning to address systemic risks, such as biodiversity loss, there is an urgency to understand how future biodiversity decline will impact businesses and how these risks may be priced.[420]

A GBI financial barrier is securing public and/or private financing.[51] There is also a vast global disparity between public (86%) and private financing (14%).[421] Private investors are crucial in tackling GBI-related sustainability challenges.[426] Comparing cost-benefits with traditional solutions remains difficult, and the limited awareness of GBI financial benefits restricts funding. A clear understanding of valuation and risk mitigation is crucial. The biodiversity funding gap is estimated at $722–$967 billion.[427] Compared with climate change finance, the biodiversity and finance agenda is underdeveloped, although key opportunities have been identified by UN and WWF TNFD and the WEF Future of Nature and Business Report.[423,428]

Technological innovation, especially AI, is transforming finance and has enormous potential to improve the objectivity of ESG, primarily for communication with regulators and investors.[429] Alternative data, such as earth observation (EO) and satellite tracking, are accelerating AI adoption. Finance companies purchase vast quantities of financial and alternative data so that their funds can maximize performance. Managing these data is complex due to balancing manager performance, data proliferation, and sustainable financial modeling. There is also a lack of consensus on ESG measurement. Without better ESG metrics, biodiversity loss estimates, GBI, and related impacts risk being inaccurate, widening the gap between projected and actual financial losses. There needs to be more resilient and consistent ESG standards, especially with the new International Sustainability Standards Board. The application of satellite/EO data and state-of-the-art AI models (e.g., computer vision, generative AI, foundation models) is enhancing environmental monitoring. Improved monitoring enables stakeholders to access more precise ESG metrics, supporting resilient GBI valuation models and better risk management.

Debt capital markets are becoming an increasingly popular source to fund GBI solutions. The debt-for-nature swap reduces a country's debt burden while allocating funds for conservation.[430,431] In this mechanism, a country repurchases its debt at a discount and issues new debt on improved terms, using proceeds for environmental projects.[430,431] Originating in 1987, the swap has gained traction in large-scale projects in Ecuador (2023), Gabon (2023), Barbados (2022), Belize (2021), and Seychelles (2015/2017).

Green bonds offer a potential solution to GBI issues, with proceeds tied to climate and environmental projects (e.g., green buildings, renewable energy).[432,433] The European Investment Bank in 2007 issued the first green bond for financing renewable energy efficiency projects.[433] Green bonds signal credible commitment through third-party certification and reputational risk for non-compliance.[433] Investors typically respond positively to green bond announcements.[432]

Blue bonds, aimed at sustainable ocean economies, require careful analysis of ecological impact. However, before issuing blue bonds, issuers and investors need to make sure they can synthesize the environmental and social impact on the ocean and marine ecosystems. In 2021, Credit Suisse arranged $364 million in blue bonds in Belize, funding coastal protection and cutting debt by 12% of GDP.[434]

Financial solutions are pivotal in supporting GBI.[435] Capital access and risk mitigation need to be prioritized. By 2030, a nature-based transition could produce US$10 trillion in business and generate 395 million jobs by 2030.[436] Achieving this requires scaling investment to $536 billion annually by 2050.[421] A strong financial ecosystem (Figure S10) is essential. Sustainability aligns finance with net zero, while addressing inequality and inclusion. *Explainability* ensures that systems behave as expected and builds transparent, trusted evidence for stakeholders. If the financial solutions are trustworthy, this will inspire confidence in individuals and organizations. Finally, resilient financial solutions must anticipate risks and support informed, adaptive decisions.

Challenges in recognizing GBI as assets

Although the economic value of GBI can be quantified by comparing the benefits against costs,[437] integrating GBI into policy priorities remains challenging. A key obstacle is that policymakers, planners, and local governments' asset managers often treat GBI as a liability rather than a formal asset. For example, while one study estimated a major London park's true value at £108 million, many councils still record parks at a nominal £1.[438]

Current accounting standards in the US and Australia fail to recognize the natural components of GBI (e.g., trees, soil, vegetation, and water) as formal assets. Under US financial rules, only human-engineered GBI (e.g., constructed stormwater systems) qualifies as an asset, while living systems are effectively assigned zero value despite their proven environmental, social, and health benefits.[439] This artificial divide overlooks the hybrid nature of GBI: for example, parks combine built infrastructures (playgrounds, pipes) with ecological features (wetlands, trees),[67] yet accounting practices capture only the former. Further, actuarial practices recognize risks such as tree-related property damage, while ignoring benefits such as flood mitigation.[440]

The Australian Accounting Standards Board[441] classifies plants as assets only if they are agricultural or bearing crops, valuing them solely by acquisition cost. Water and non-commercial vegetation—even when critical for carbon sequestration—are excluded. Remote sensing and modeling advancements have improved carbon stock quantification, yet frameworks such as the National Carbon Accounting System focus narrowly on emissions rather than asset valuation.[442]

Globally, GBI's asset recognition remains inconsistent. While the UK integrates GBI into planning policies, challenges persist, such as undervaluing GBI components (e.g., sustainable drainage systems) and gaps in performance metrics.[66,443] Meanwhile, private developers exploit the market appeal of green proximity without contributing to GBI upkeep, highlighting a disconnect between perceived value and institutional accounting.[444]

A core barrier to GBI investment lies in its exclusion from institutional asset frameworks. Local governments, which hold the property rights and are chiefly responsible for managing urban GBIs, face significant budgetary and regulatory constraints because GBI rarely generates direct revenue under current accounting standards. The lack of formal recognition of GBI as assets in local accounting rules means that access to innovative financing (e.g., loans backed by tangible revenue streams) is limited.

The demonstrable benefits of GBI demand institutional realignment and formal recognition. Over a decade ago, Barbier[445] advocated for ecosystems such as wetlands to be valued as natural assets, given their provision of goods, services, and cultural benefits. Echoing this, Vardon et al.[446] called for governments to prioritize natural capital by integrating environmental accounting into core decision-making, bridging economic and ecological data.

Notable advancements are being made. Roghani et al.[447] argued that GBI performance must be evaluated holistically, balancing costs against all primary and secondary benefits, aligning with ISO 55000's asset management principles.[448] Practical initiatives, such as EU's Natural Capital Accounting initiative, the IPWEA's guidance for Australian local governments,[449] and the System of Environmental-Economic Accounting (SEEA), in ecosystem accounting[450] are advancing standardized assessment methods for GBI.

Market-based mechanisms offer further promise. Carbon markets exemplify how public-private investment can monetize environmental assets, creating revenue streams for loans and reinvestment.[451] Emerging markets for

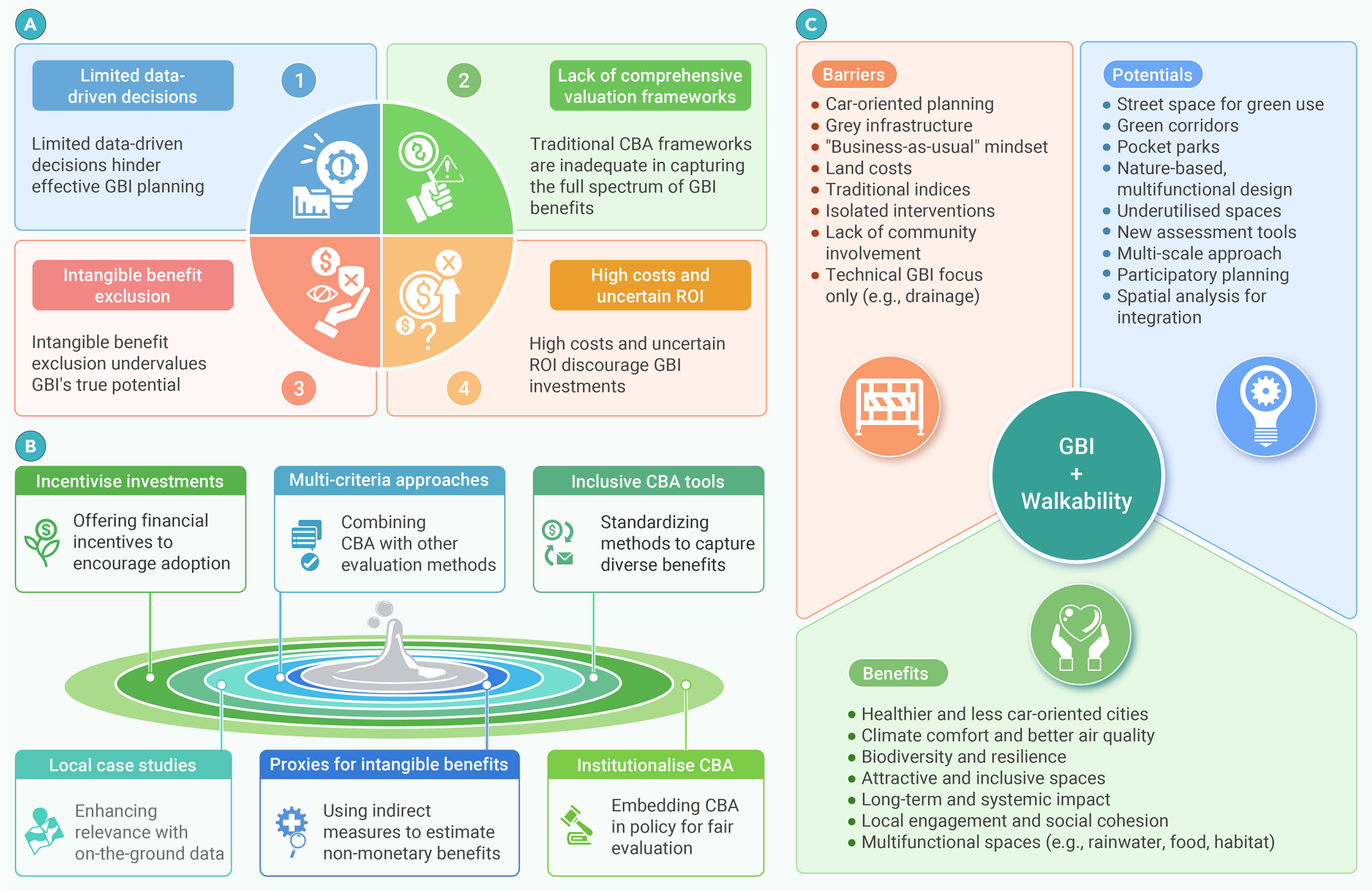

Figure 5. Schematic representation of key CBA and walkability challenges and corresponding strategies (A) Each numbered icon in this figure illustrates the core challenges in CBA that hinder the GBI implementation. (B) Targeted strategies, each linked to one or more of the challenges listed in (A), that help overcome CBA challenges in GBI implementation and drive more informed, fair investment decisions (see lack of comprehensive cost-benefit analysis. (C) Barriers, potentials, and benefits of integrating GBI and walkability strategies in urban design (see conflicts in promoting GBI and walkability).

biodiversity or water quality credits—although currently underdeveloped—could follow a similar path. Initiatives such as Australia's Nature Repair Market signal progress,[452] by credibly monetizing co-benefits through market mechanisms, GBI can shift from being undervalued ecological infrastructure to recognized financial assets. This shift could unlock mainstream investment, empower local governments to act as accredited sellers, and establish sustainable revenue streams that justify GBI's formal inclusion in institutional accounting frameworks, enabling access to innovative financing for GBI managers.

Despite its proven environmental, social, and economic benefits, GBI remains systematically undervalued in policy and practice. Institutional frameworks in many countries—constrained by rigid accounting standards—continue to classify GBI as a liability rather than an asset, with only its human-engineered components meeting traditional asset criteria. To address this imbalance, GBI must be redefined as natural capital assets, supported by valuation frameworks that holistically account for both costs and multifunctional benefits. Emerging approaches, such as ISO 55000-aligned asset management, Environmental-Economic Accounting, nature-positive markets, and hybrid green-gray designs, demonstrate viable pathways to institutionalize GBI's value. Critical steps include revising accounting standards to recognize ecological assets and the benefits they provide, developing tools to quantify GBI performance across environmental, economic, and social dimensions, and leveraging carbon and biodiversity credits to monetize ES. The urgency of climate adaptation and biodiversity loss demands that cities treat GBI as foundational infrastructure, not an optional amenity.

Lack of comprehensive CBA

CBA is commonly employed to assess the economic viability of any GBI intervention before making investment and implementation decisions.[453] It translates ecological values into financial metrics and may shape GBI innovation.[454,455] Lacking comprehensive CBA understates GBI sustainability and effectiveness.[453] Traditional CBAs overlook indirect benefits and externalities in GBI,[453,456,457] leading to underinvestment.[456,458]

Methodological gaps present significant challenges in GBI evaluation. The difficulty in monetizing intangible benefits (ecological and social values) hinders the full cost-benefit analysis.[459,460] Specifically, comprehensive CBA of cultural heritage, biodiversity, equity, and well-being remains challenging in terms of putting monetary values of such services,[459–462] while benefits such as stormwater control and energy savings from green roofs are more straightforward to quantify.[460] GBI benefits with direct or indirect impacts on human health would class as intermediate complexity to value in monetary terms, but are increasingly included for regulating ES functions that GBI provide, such as air pollution removal,[463,464] cooling,[376,465] and noise mitigation.[150] The absence of adequate valuation techniques and inconsistent methodologies[457] further complicates full-scale assessments of GBI's ecosystem functions.

Economic barriers also impede GBI implementation. High upfront costs and uncertain returns discourage stakeholder investment.[466] Studies show that green roofs, water squares, and other GBI often fail to achieve favorable benefit-cost ratios within typical 30-year project spans, losing investor and policymaker interest.[457,467] Green roofs, in particular, are economically viable only with subsidies due to high construction and maintenance costs, often yielding zero or negative net present value.[468] Even if savings from avoided may not offset the high initial costs when other co-benefits are excluded.[469]

Contextual variability poses additional analytical challenges in CBA of GBI. Data scarcity and variations by geography, neighborhood, or GBI type complicate analysis due to benefits' dynamic spatial and temporal variability (Figure 5A).[457,470,471] CBAs are sensitive to uncertainties, model assumptions,

and data availability,[470] especially for impacts such as mental well-being or noise reduction.[460,472] Context-specific performance variability[473] and poor empirical evidence[454] further limit comprehensive evaluation of GBI's values and services.

These methodological and contextual gaps demonstrate the need to institutionalize CBA in GBI planning. Undervalued intangible benefits suggest less recognition of ES and social cohesion as GBI.[461,474] The insufficient consideration of improved air and water quality, biodiversity, ecosystem resilience, noise reduction, and increased property values[454] results in underestimation of GBI's total value, deterring further investments.[475] Table 4 summarizes case studies highlighting these challenges, revealing persistent gaps in CBA that hinder GBI implementation, along with key recommendations to overcome these challenges.

Integrated strategies can address challenges and promote wider GBI investment (Figure 5B). Real-world case studies as proxies, such as value changes and insurance data, help quantify the benefits of urban greening and flood mitigation.[482,483] These approaches reduce uncertainty and help build more inclusive valuation frameworks. Studies suggest the need for long-term, context-specific data (e.g., real estate, insurance, and hospital records) to assess flood and health benefits.[484] Incorporating local case studies and stakeholder input improves data relevance and reflects real-world conditions.[455,485] Financial concerns, particularly high upfront costs and uncertain return on investment, can be mitigated by integrating co-benefits comprehensively through life cycle assessments and offering targeted incentives, such as tax relief or subsidies.[226] Similarly, institutionalizing CBA by integrating the CBA framework into the planning processes could result in a fair and comprehensive manner of evaluating NbSs.[457] Improved modeling approaches, which are better able to capture context-specific GBI performance, coupled with health assessments and economic valuation of the associated benefits, could address some of the valuation challenges,[464] but are still underutilized and not fully standardized. For instance, a comprehensive CBA of street trees in Adelaide, Australia, demonstrated a 1.6 benefit-cost ratio across various neighborhoods, providing valuable evidence to support urban greening policies.[486]

While CBA holds potential to guide innovation and investment in GBI, its current application remains limited and incomplete. To account for the whole economic profitability of GBI, a standard CBA method must be developed incorporating more inclusive, transparent, and context-aware tools that can capture the full spectrum of ecological, social, and economic values, ensuring that NbSs are not just observed but valued and implemented for their full economic potential.

GOVERNANCE/POLICY BARRIERS

This section identifies four barriers to GBI implementation: land scarcity and urban sprawl, urban design limitations, unclear GBI policies, and the disconnect with walkability. Governance frameworks are essential for scaling and sustaining GBI, yet progress is hindered by fragmented legislation, weak political support, and outdated planning practices. Figure 6 presents the barriers in a layered format, linking each to broader governance shortcomings and corresponding strategies—ranging from spatial planning improvements to enhanced coordination. Challenges and corresponding solutions are explored in the subsections and summarized in Table 3, and a summary of discussed case studies is presented in Table S1.

Land scarcity and urban sprawl

Land scarcity and urban sprawl present interconnected challenges for implementing GBI in cities. Intense competition among residential, commercial, and infrastructural uses in dense urban areas makes space for GBI financially and spatially limited.[487,488] This constraint is especially acute in rapidly growing metropolises such as São Paulo, where new GBI creation can be considerably limited by land use disputes.[489] Rising land values further restrict the feasibility of large-scale GBI projects, as development priorities favor profit-driven densification over public benefit.[490,491] Simultaneously, urban sprawl, characterized by low-density expansion consumes peri-urban lands that could otherwise host greenbelts or ecological corridors. It fragments natural ecosystems, reduces biodiversity, and increases car dependency and emissions.[487,492,493] Bucharest exemplifies how unchecked sprawl directly converts natural land and disrupts ecological continuity, while also inflating the cost of sustainable infrastructure, leading to reliance on traditional gray systems instead of integrated approaches such as water-sensitive urban design.[487,494,495]

Both challenges contribute to fragmented, underperforming green networks. In dense cities, limited space constrains the dimensions and connectivity of parks and ecological corridors, while making retrofitting technically and financially challenging.[496,497] In sprawled areas, although land may be more abundant, GBI is often poorly maintained, spatially isolated, or inaccessible, diminishing the ability of GBI to deliver critical ES, such as flood and noise mitigation and recreational opportunities.

To effectively address these constraints, cities must adopt space-efficient solutions and strategic approaches. Micro-scale interventions such as green walls and roofs can be incorporated into existing buildings[4,5] without requiring additional land,[498–502] while supporting multiple functions including heat mitigation, biodiversity, reduction of energy use, and air and noise pollution.[503–505] Similarly, permeable surfaces help water infiltration, reducing stormwater runoff, and recharging groundwater.[506] Street trees and pocket parks maximize urban space, providing shade, cooling, and green areas that prevent the UHI effect.[507–509] By strategically integrating these GBI elements into the existing urban fabric, cities can achieve sustainability benefits without contributing to further sprawl,[510–512] although regular maintenance is essential for maintaining effectiveness.

Urban design barriers

GBI faces distinct challenges across technical, economic, social, and policy dimensions. These include: (1) technical integration challenges: balancing architectural demands and costs with ecological needs, particularly in innovative solutions such as rooftop greening; (2) functional conflicts: competition between ecological benefits and practical functionality in limited urban space; (3) multi-scale coordination challenges: fragmentation of regional ecological networks by property divisions; (4) design maintenance disconnect: insufficient consideration of maintenance requirements, leading to functional degradation and becoming "one-time projects"; (5) policy and technological inertia: traditional frameworks and established technical configurations that resist innovation and cross-sectoral collaboration (Table 5).

While expanding GBI areas can help reduce temperatures,[513] high building coverage in high-density urban areas often limits its implementation.[514] Additionally, urban expansion's encroachment on natural GBI poses a severe problem.[515] Against this backdrop, there is a need to combine multifunctionality and regional coordination for GBI deployment,[516] requiring a paradigm shift from traditional design approaches to an ecological framework. Traditional planning models are strictly based on land use classifications (residential, industrial, or commercial) and rarely consider whether land is suitable for specific functions or its consistency within a broader territorial context.[261,517]

Given GBI's critical role in ecological service systems, connectivity and multifunctionality have become core design principles.[518] However, current GBI development and design research remains fragmented, focusing on micro-scale elements such as street trees,[519,520] community green spaces,[521] or city-level tree canopy coverage,[522] with limited consideration of multi-scale connectivity. Furthermore, while many studies aim to enhance GBI's potential through life cycle risk assessment models[523] and CBA,[524,525] life cycle aspects such as maintenance are often overlooked during actual design and implementation.

Current policies and technical preferences prioritize conventional engineered solutions,[526] while established technical configurations have become institutionalized and rigid over time,[527] further influencing decision-making by urban designers and policymakers to favor entrenched methods.[362] Insufficient cross-sectoral coordination and inadequate public participation mechanisms further constrain GBI implementation. However, studies show that integrated planning approaches combining technical solutions with community co-governance are gradually improving feasibility.[528,529] While many cities recognize GBI's importance in local regulations, developers are often unaware of these guidelines, particularly in low- and middle-income countries (LMICs).[530] For example, Ekostaden Augustenborg in Malmö, Sweden, transformed a declining 1950s housing district through integrated redevelopment that combined nature-based stormwater systems and green roofs with community co-design and governance.[531]

Integrating GBI into urban design requires a holistic approach that reimagines space, technology, and governance (Table 5). Spatial constraints can be

Table 4. Summary of the most relevant studies highlighting CBA hindering GBI implementation along with the key recommendations to overcome these challenges

CBA challenges	Case study/review	Key findings	Key recommendations	Reference
Challenges in monetizing intangible benefits	Ayutthaya Island, Thailand	valuing ES is difficult, limiting recognition of mitigation benefits	develop indicators reflecting various ecosystem service to enhance valuation	Vojinovic et al.[461]
	43 US, D.C., and Canada	challenges in quantifying intangible benefits due to GI's complex structure	collect detailed data to better understand and value intangible benefits	Kim and Song[462]
	Systematic review (79 articles)	difficulty in monetizing benefits such as well-being and noise reduction; often excluded from economic evaluations	develop consistent methodologies to account for intangible benefits	Teotónio et al.[460]
	literature review (129 studies)	challenges in monetizing intangibles such as quality of life, well-being, and biodiversity	utilize multi-criteria analysis to quantify these benefits for integration into CBAs	Manso et al.[459]
Exclusion of co-benefits and externalities	systematic review (114 observations)	many CBAs overlook indirect benefits and environmental externalities underrepresenting GBI	combine CBA with complementary methods such as multi-criteria analysis, cost-effectiveness analysis, and qualitative assessments to better capture co-benefits	Chelli et al.[453]
	Sheffield, England, and other European cities	traditional valuations often miss co-benefits such as health, equity, and ecosystem resilience	adopt holistic frameworks and increase public sector engagement to support investments that reflect all benefits	Wild et al.[458]
	case study: Modesto, CA	existing CBAs may exclude urban forestry co-benefits such as species variability and ecological functions	promote research on species-level ecological performance to better incorporate of benefits into economic models	McPherson[476]
Lack of comprehensive data and standardized methods	Bruges, Belgium	difficulty in estimating exact values due to combined use, non-use, and investment values	develop a clear handbook to guide users in generating and understanding benefit transfer values	Vandermeulen et al.[477]
	Sint Maarten Island, Caribbean	challenges in monetizing co-benefits and uncertainties due to data availability and local issues	include a broader range of co-benefits to improve data collection and integration	Alves et al.[456]
	Barcelona and Badalona, Spain	lack of reliable data and standardized methods for hydrological performance of GI	use approaches such as Monte Carlo simulations to address uncertainties	Locatelli et al.[470]
	rapid evidence assessment (1,700 documents)	lack of reliable data and standardized methods for assessing NbS impacts	develop new methods beyond traditional CBAs to improve data collection and integration	Raymond et al.[472]
	book review (Europe, North America, China)	lack of comprehensive data and standardized methods for evaluating NbS effectiveness	systematically analyze data, including CBAs, and identify causal mechanisms to improve data collection and standardization	Kabisch et al.[478]
	London, UK	lack of comprehensive data and standardized methods for evaluating physical and social outputs of community gardens	use simplified CBA methodologies to improve accessibility and standardization	Schoen et al.[455]

(*Continued on next page*)

Table 4. *Continued*

CBA challenges	Case study/review	Key findings	Key recommendations	Reference
CBA uncertainty	Oslo, Norway	CBAs are sensitive to data availability, uncertainties, and model assumptions	validate results using real-world data sources, such as insurance data, to improve accuracy	Wilbers et al.[457]
	Jung-gu, Seoul, South Korea	uncertain assumptions and regional factors reduce reliability of CBA	undertake additional region-specific studies that consider environmental, socioeconomic, and physical factors to improve decision-making	Shin and Kim[454]
	Madrid, Spain	critique of CBAs due to uncertain assumptions, discounting and lack of communicating output uncertainties	consider both monetary and biophysical values in decision-making to reduce uncertainty	Babi Almenar et al.[479]
	Newcastle, UK	difficulty in monetarily valuing multiple benefits of GBI	use tools such as CIRIA BeST to structure assessments for quantifying and monetizing each benefit	O'Donnell et al.[480]
High costs and uncertain return on investment (ROI)	Tanyard Branch Watershed, Athens, GA	green roofs have a higher net present value (NPV) compared with conventional roofing, indicating higher costs and uncertain ROI	gain more experience and establish long-term warranties to justify investments	Carter and Keeler[467]
	Grand Rapids, MI	high costs and uncertain ROI for green roofs; negative NPVs unless part of LEED-certified buildings	incorporate green roofs into LEED-certified buildings to leverage additional benefits such as rent premiums	Nordman et al.[468]
	Genoa, Italy	high installation and maintenance costs lead to uncertain ROI, especially for intensive green roofs	recommend tax incentives to improve ROI	Perini and Rosasco[475]
	Southern France and Rotterdam, the Netherlands	benefits from avoided damages are often insufficient to cover costs, leading to high costs and uncertain ROI	adapt public funding rules to assess for cross-sectoral assistances of NbS	Le Coent et al.[469]
	systematic review (116 articles)	elevated costs and uncertain ROI stem from insufficient knowledge about expenses, benefits, and impacts	develop guided examples of cost calculation, depreciation, and discounting to aid in creating credible business cases	Van Oijstaeijen et al.[226]
	Kuala Lumpur and Johor Bahru, Malaysia	high costs and uncertain ROI of green roofs, particularly for local authorities make the economic worth unclear	integrate intensive green roofs on flat rooftops to reduce costs and improve ROI	Shazmin Shareena and Nur Amira[481]

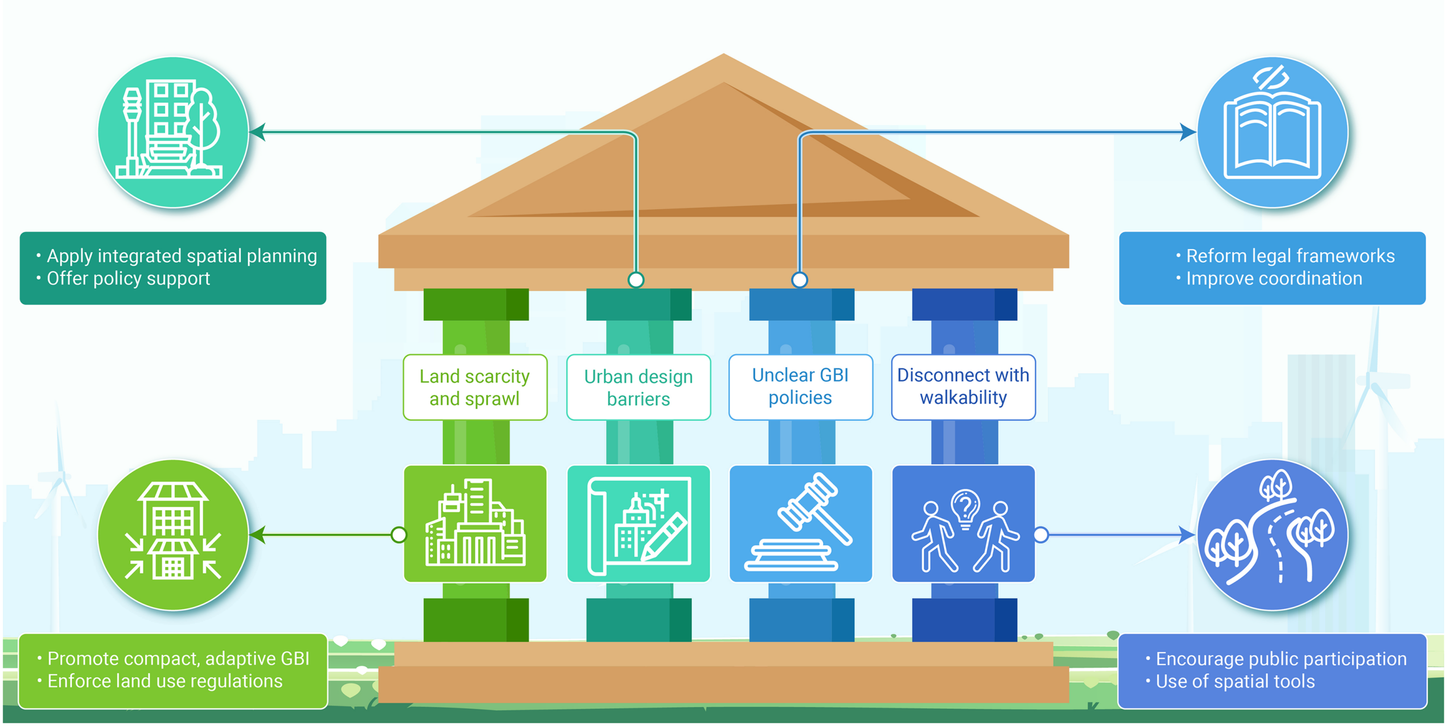

Figure 6. Governance and policy barriers to GBI implementation and corresponding solutions Each pillar highlights a critical challenge—land scarcity and urban sprawl, urban design limitations, unclear policies, and disconnect with walkability—alongside strategic interventions required to overcome each barrier.

addressed through three-dimensional design,[532,533] such as optimizing structural load distribution while incorporating hydroponic or modular planters to reduce heat islands and improve air quality.[534,535] Rooftop agriculture can be designed using lightweight substrates and automated irrigation systems to maximize productivity without compromizing building integrity.[536] Functional compatibility demands synergistic design, where permeable pavements are paired with underground retention systems to manage peak flows and prevent flooding, while bio-retention facilities replace conventional drainage channels to enhance biodiversity and water quality.[537] Multi-scale coordination relies on GIS-driven planning tools to map ecological corridors and align regional networks with site-specific development, ensuring seamless integration across urban scales.[538] Maintenance challenges are mitigated through low-maintenance technologies, such as drought-resistant plant communities and modular, easily replaceable components, while community-based stewardship programs foster accountability and long-term sustainability. Policy frameworks must evolve to incentivize GBI through tax abatements and regulatory updates, while interdisciplinary collaboration ensures that ecological, social, and economic objectives are balanced.[8] By embedding resilience thinking into design, GBI transitions from an ancillary feature to a foundational element of urban systems, creating adaptive, equitable, and ecologically functional cities.

In order to overcome the challenges of GBI in urban design, it is necessary to take multidimensional measures.[247] The use of data-driven design and cross-disciplinary technological integration is crucial,[529] while also focusing on policy innovation and institutional safeguards. Establishing specialized agencies and cross-departmental cooperation mechanisms can help strengthen supervision and reform. In terms of talent cultivation, both cross-disciplinary talent training and vocational staff training can enhance the professional quality and the collaborative abilities of relevant personnel. Comprehensive participation mechanisms and capacity building and education programs can be used to engage community residents.[8] By implementing these structural and institutional approaches in conjunction with practical technical measures, GBI can be transformed into a core framework for urban resilient development, ultimately promoting sustainable urban transformation.

Lack of clear GBI implementation policies

The lack of clear policies for GBI implementation is a widely discussed barrier in the literature and generally exhibits a high degree of transversality, inherent to the nature of policymaking itself. This barrier does not always appear in the scientific literature as a topic with clearly defined boundaries. Rather, it can be addressed as an institutional and governance barrier,[70] encompassing critical themes such as leadership, strategic vision, political commitment, inter-agency cooperation, legislative frameworks, environmental policies, and conflicting policy actions. Alternatively, it may be framed within a broader socio-political understanding of barriers, capturing intrinsic societal elements that ultimately shape the reluctant behavioral patterns of policymakers in the gray-green transition.[480] Within this socio-political perspective, the lack of clear GBI policies appears even more transversal, expanding to include additional factors, such as negative experiences, responsibility, capacity, knowledge, organizational culture, financial constraints, administrative bureaucracy, lack of clear standards and guidelines, and resistance to change.[539–542] Considering the broad scope of this barrier, its challenges will be grouped into five key topics: leadership, tradition, legislation, knowledge, and priority conflicts.

The lack of leadership, clear vision, and political will constitutes a major barrier to the implementation of GBI, particularly at lower levels of public administration where GBI is often absent from urban planning agendas.[143,543,544] Weak leadership is further reflected in institutional fragmentation, ineffective communication, lack of commitment, ambiguous role definitions, and the isolation of managers across different sectors.[372,544,545] Without a long-term vision, managers are unlikely to support initiatives whose results extend beyond their administrative term.

Economic interests historically associated with gray infrastructure valued for its visual prominence, perceived contribution to economic growth, and potential for job creation continue to pose a significant political barrier to the adoption of GBI.[480] Since GBI projects mature slowly and lack immediate electoral visibility, they are less politically attractive.[480,546]

The absence of specific legislation and interjurisdictional authority limits GBI implementation.[70,480,540,547] Environmental challenges that cross administrative boundaries demand federal regulation. Current legal frameworks often mandate conventional materials, reinforcing gray infrastructure solutions.[70,548] The limited integration of scientific knowledge into policymaking often results in unrealistic expectations for GBI projects. Climate change and urban environmental shifts are often inadequately considered, reducing GBI efficiency and adoption continuity.[480,545] Gaps in understanding the societal benefits of GBI and inadequate training, particularly in engineering fields, exacerbate the problem.

Limited financial resources, especially in LMICs, hinder GBI policy development, as these initiatives often struggle to compete with gray infrastructure

Table 5. Key urban design challenges hindering effective GBI implementation and potential technical and strategic solutions

Challenges	Solutions	References
Spatial integration challenges	• vertical greening systems<br>• optimize structural load distribution<br>• incorporating hydroponic or modular planters<br>• rooftop agriculture with lightweight substrates and automated irrigation systems	Xi et al.[513]; Su et al.[514]; Blair and Johnson[515]
Functional conflicts	• bio-retention areas integrated into parking lots<br>• permeable pavements paired with underground retention systems<br>• bio-retention facilities replace conventional drainage channels	Cortinovis et al.[516]; Ronchi et al.[261]; Pogliani et al.[517]
Multi-scale coordination challenges	• map ecological corridors<br>• align regional networks with site-specific development<br>• ensuring GBI integration across urban scales	Pauleit et al.[518]; Xi et al.[519]; Xi et al.[520]; Xi et al.[521]; Kanniah[522]
Maintenance deficiencies	• drought-resistant plant communities and modular<br>• easily replaceable components	Tabatabaee et al.[523]; Rosa et al.[524]; Barbosa et al.[525]
Policy and technological lag	• GBI design by embedding resilience thinking<br>• tax abatements and regulatory updates<br>• interdisciplinary collaboration	D'Amato and Korhonen[526]; Fuenfschilling et al.[527]; Davies and Lafortezza[362]; Tapia and Reith[528]; Tapia et al.[529]; Ahmed and de Oliveira[530]

in CBAs.[70,372,480] Without public recognition of GBI's value, political pressure is minimal, perpetuating a cycle of invisibility and exclusion from the political agenda.

Overcoming the challenges to GBI implementation policies requires addressing two fundamental and interdependent aspects. First, the transition from gray-to-green infrastructure will be slow and gradual, demanding continuous relearning by academics, politicians, and managers. As this transformation unfolds over a longer timescale than electoral cycles, strong and comprehensive environmental policies across all levels of public administration are essential.[549] Over time, as the benefits of GBI become better valued, political and economic interests traditionally tied to gray infrastructure are expected to shift toward greener solutions.[550] Melbourne's long-running gray-to-green initiative is a striking example of a slow, strategic shift from gray to GI, supported by enduring policy frameworks across local government.[551]

Second, human resource training and knowledge production are critical.[552,553] GBI solutions are relatively new compared with conventional structures and remain unfamiliar to many managers and technicians. Professionals trained under traditional urban engineering paradigms often resist innovative, systemic approaches and lack technical training, didactic guides, regulatory support, and confidence in GBI models. To enable the gray-to-green transition, curricular reforms are necessary to equip future professionals with the skills needed to implement GBI solutions.[554,555]

The effective implementation of GBI is hampered by the lack of clear, cohesive policies, rooted in both governance challenges and broader socio-political dynamics. Key obstacles include inadequate leadership, vision, and political will; economic interests favoring gray infrastructure; outdated legislation and fragmented regulatory authority; and poor integration of scientific knowledge into policymaking. Financial constraints and competing priorities, especially in LMICs, further marginalized GBI, creating a cycle of invisibility due to limited public demand. Overcoming these barriers requires strengthening leadership, reforming legislation, promoting knowledge dissemination, and increasing public awareness to establish GBI as a political and societal priority.

Conflicts in promoting GBI and walkability

Urban GBI is a multipurpose strategy to address urgent societal problems by offering ESs that improve public health and quality of life and thus can increase walkability. However, despite these benefits, recent research has shown that GBI and walkability have been poorly integrated, revealing a paradox that demands deeper reflection on the barriers hindering their synergy.[556] A multi-scale approach is essential, combining urban design with social and environmental needs. Car-centric policies, gray solutions, prioritization of conventional infrastructure investments, and "business as usual" planning need to be revisited to avoid obstructing integration. In addition, GBI projects must be developed from a multipurpose perspective, including enhancing pedestrian attraction.

Walkability contributes significantly to urban quality and public health,[557,558] being defined as the effectiveness in promoting and facilitating walkways by ensuring comfort and safety, linking people to diverse destinations, and creating visually engaging routes.[559,560] This relationship is exemplified by a study from Michigan, USA, where walkability is associated with reduced obesity rates and improved mental health outcomes.[561]

Features that support well-being relate to the provision of walkable, community, and vegetated areas, emphasizing the importance of integrating urban planning, biodiversity, and ES.[562] Thus, the production of CESs arises from the interrelations between people and the landscape, with urban green areas understood as cohesive systems that offer comprehensive human experiences.[67,563]

Promoting walkability decreases sedentary behavior and encourages physical activity, thereby reducing incidence of various health conditions such as obesity, diabetes, and anxiety.[564,565] The pedestrian pathways quality depends on multiple aspects of the built environment, including sidewalk width, presence of trees, safety, mixed land use, active frontages, lighting, etc.[560,566] Walkable areas tend to be less car dependent, which leads to improved air quality, reduced noise, decreased urban heat, and an increased offer of many other ES. The implementation of GBI contributes to increasing urban greenery and can significantly improve walkability. In addition, these strategies support resilience.[567–569]

However, despite multiple benefits, recent research indicates a poor correlation between GBI and walkability.[556,570,571] Factors include car-oriented planning, the prioritization of gray infrastructure, and land costs in denser areas. Engineering projects for drainage, heat islands, brownfield revitalization, and green areas that prioritize conventional solutions are examples of barriers. Also, many walkability assessment indices do not properly incorporate solutions focused on GBI, overlooking broader benefits of a walkable environment.[556] Finally, a lack of public participation and local contextualization limit success.[568,572]

In terms of streetscape design, a diverse array of GBI strategies can be adopted, ranging from creating GCs that provide shade and thermal comfort to technical solutions that absorb stormwater. For a more effective implementation, it is essential to consider multiple scales of walkability. Alignment between GBI and site potential at the macro-scale is necessary prior to micro-scale interventions, ensuring that impacts extend site-specific actions. In denser areas, promoting linear and pocket parks, or reappropriating smaller spaces such as parking spots, can create attractive areas for pedestrians, recognizing that their long-term benefits outweigh the initial costs.[573]

Spatial analysis tools, including GIS, can be used to identify vegetated spaces and evaluate their potential to provide ES.[574] These areas can be transformed into pedestrian networks integrated with GBI,[568,575] offering benefits such as improved air quality.[5,37] Moreover, new methodologies for public policymaking and engaging communities are necessary, particularly those addressing climate change and biodiversity in walkability initiatives.[572] In this context, GBI emerged as a strong ally, offering potential to mitigate and adapt the impacts of extreme weather while increasing biodiversity and other urban benefits.[576]

By working across macro and micro scales, interventions can foster more walkable green spaces,[247] enhancing public health and social interaction while also contributing to broader ES benefits such as enhancing habitats, reducing urban heat, improving air quality, mitigating floods, capturing rainwater, and promoting food production.

Although GBI and walkability are often recognized as complementary components of sustainable urban development, their relationship is complex. The observed disconnection between greenness and walkability highlights the need to move beyond merely co-locating green spaces and pedestrian infrastructure (Figure 5C). Instead, a more integrated, multi-scalar approach is required, incorporating innovative analysis, policymaking, and active public participation. Further research is essential to deepen understanding and strengthen the synergies between GBI and walkability, ensuring that their combined potential can be implemented in future urban environments.

CONCLUSION AND RECOMMENDATIONS

This review presents a comprehensive cross-disciplinary synthesis of underexplored barriers and emerging challenges in GBI implementation within urban environments. By integrating previously fragmented perspectives from urban design, climate science, economics, and social equity research into a cohesive framework, the study bridges disparate knowledge domains and situates GBI within broader urban system dynamics to identify actionable strategies for resilient adoption.

Environmental barriers

- Potential conflicts between solar energy production and GBI can be resolved through integrated planning approaches that harmonize climate resilience and carbon neutrality goals without sacrificing valuable urban landscapes.
- The fragmentation of GBI research into disciplinary silos prevents full realization of its multifunctional benefits. Interdisciplinary studies integrating physical measurements with human adaptation factors are critical to properly capture GBI's multifunctionality in urban environments.
- Potential negative impacts of GBI, including effects on air quality, allergen production, GHG emissions, and water/soil dynamics, require thorough consideration during planning stages and continuous monitoring throughout implementation.
- Implementation requires careful consideration of interactions between building systems, ventilation, and climate management across local and wider urban scales to maximize co-benefits and prevent unintended consequences.
- Comprehensive assessment of GBI thermal resilience potential requires integrating physical temperature measurements with subjective human adaptation factors, leveraging technologies such as remote sensing, digital twins, and AI to improve heat risk mapping across demographics.
- Long-term GBI robustness requires science-based plant selection informed by ecological and evolutionary research on species' adaptability to harsh urban environments and changing climate conditions, creating low-maintenance, long-lived solutions.
- Multi-scalar environmental planning must align interventions across block, neighborhood, watershed, and metropolitan scales, to overcome spatial mismatches between fragmented deployments and broader ecological processes.
- Urban BI requires context-sensitive restoration approaches that balance inherent risks (e.g., flooding and water-borne diseases) with potential benefits to urban character, ESs, and community well-being.

Social barriers

- Justice frameworks must be embedded throughout planning, design, and governance processes, acknowledging historic inequalities while ensuring inclusive approaches that prevent green gentrification and actively disrupt cycles of environmental marginalization.
- Planning must incorporate traditional ecological knowledge and heritage values beyond tokenistic engagement, as interventions that resonate with community identity enhance both ecological performance and long-term stewardship of GBI.
- Successful implementation requires addressing social adoption barriers through genuine community engagement that aligns technical and ecological priorities with local values, reforming fragmented institutional structures, and ensuring equitable access.
- Safety concerns must be systematically addressed through integrated approaches combining CPTED principles, smart technologies, and community participation to ensure all residents feel secure and welcome.
- The tension between ecological functionality and conventional esthetics can be addressed through design elements that signal human care, institutional reforms that value resilience over immediate visual appeal, and approaches that transform public understanding of "beautiful" landscapes.

Economic barriers

- Scaling GBI investment requires developing innovative financing mechanisms (eco-bonds, conservation-linked debt) and leveraging new technologies for credible ESG metrics that attract private capital while properly valuing nature's contribution to economic prosperity.
- Recognizing GBI's true value requires institutional realignment through standardized capital accounting frameworks such as SEEA, performance-based asset management approaches aligned with ISO 55000, and credible market mechanisms that monetize ES.
- Implementation requires standardized cost-benefit methodologies incorporating real-world proxies, long-term case studies, and life cycle assessments that capture previously overlooked intangible benefits, enabling decision-makers to quantify cultural, health, and biodiversity values despite high upfront costs.

Governance barriers

- Urban land constraints can be addressed by maximizing existing infrastructure through strategic integration of micro-scale interventions such as green roofs, walls, permeable surfaces, and pocket parks that deliver multiple benefits without requiring land acquisition or contributing to further sprawl.
- Advancing GBI in urban design requires data-driven three-dimensional spatial design strategies, interdisciplinary integration of ecological and gray infrastructure, innovative policies, and active community engagement to elevate GBI from isolated interventions to a foundational element of resilient urban development.
- Implementation demands both strong environmental frameworks across governance levels and reformed educational curricula to equip professionals with systemic thinking skills that build resilience into urban governance structures and outlast electoral cycles.
- Bridging the GBI-walkability disconnect requires multi-scale integration strategies that combine GC networks with streetscape interventions, supported by GIS-powered spatial analysis and inclusive governance mechanisms, to fragmented urban landscapes into interconnected systems.

Twelve integrated recommendations derived from 21 barriers were developed. Each recommendation is mapped to its specific barrier(s) in the list below, with comprehensive linkage details provided in Table S13.

Strengthen interdisciplinary collaboration to overcome siloed GBI approaches. Cross-sectoral cooperation among ecologists, planners, engineers, and climate and air pollution scientists can shift the focus from

single-issue solutions to multifunctional strategies. Employing ecological production functions and ES frameworks enables holistic planning, enhancing biodiversity, mitigating urban heat, minimizing air pollution, and optimizing energy usage in buildings (see siloization in urban GBI studies and conflict control among urban environmental challenges). This recommendation is aimed at municipal policymakers and planners, fostering inter-agency and cross-sectoral collaboration.

Prioritize science-driven species selection and adaptive design to minimize environmental threats. For example, to avoid air quality disbenefits, selection of low-allergenic and insect-pollinated species using tools such as OPALS, can reduce respiratory health impacts in vulnerable groups with higher allergy susceptibility, while choosing plants with minimal bVOC emissions prevents secondary air pollution. Incorporating optimal canopy structures and staggered arrangements maintains street level ventilation and prevents pollution trapping (see unintended consequences and pathways to resilient GBI via plant adaptation). This recommendation is directed at urban ecologists, planners, and public health professionals, encouraging species choice and adaptive design to reduce pollution and allergen risks.

Harness microclimate modeling and AI for indoor-outdoor ventilation needs and GBI design. To integrate GBI into urban ventilation strategies, there is a need for strengthening collaboration among planners, architects, ecologists, and health experts. Using urban microclimate models and integrating machine learning with GI mapping can help develop context-specific solutions that preserve airflow, protect HVAC inlets, and prevent heat and pollutant entrapment, while ensuring that vegetation is spatially configured for maximum benefit (see urban ventilation and GBI trade-offs). This recommendation highlights the role of urban planners and ecologists in applying microclimate modeling and AI for evidence-based airflow, heat regulation, and pollution management.

Integrate thermal adaptation and vulnerability information into GBI planning. Shift the focus from generalized cooling benefits to nuanced human thermal adaptation, particularly for vulnerable populations. Remote sensing, digital twins, and heat exposure evaluation can identify urban overheating risks to develop targeted interventions that align with both physiological response patterns and microclimate (see thermal resilience and environmental injustice and GBI decision-making"). This recommendation calls on urban planners, public health agencies, and adaptation teams to embed vulnerability mapping into planning to better target interventions for sensitive populations.

Integrate social dimensions explicitly into GBI research frameworks and performance evaluation. Future studies should systematically incorporate metrics for social acceptance, cultural relevance, safety perception, and equity outcomes when assessing GBI effectiveness, moving beyond purely ecological or technical indicators (see cultural perspectives on GBI, social adoption hindering implementation of GBI, and balancing climate adaptation and esthetic goals in urban GBI). This recommendation is aimed at urban researchers, social scientists, and local governments, embedding equity, cultural acceptance, and community priorities in GBI performance evaluation to embed equity, and cultural and social acceptance.

Promote cross-disciplinary collaboration to align GBI design with community realities. GBI planning must co-develop ecological functions and social values by involving social scientists, community groups, and community knowledge holders during initial planning and design phases (see social adoption hindering implementation of GBI and safety and security barriers in GBI implementation). This recommendation emphasizes collaboration between planners, social scientists, local governments, and community groups to co-develop designs that balance ecological benefits with lived social values.

Develop context-sensitive policies that balance ecological resilience, public safety, and esthetic expectations. Urban policies should guide GBI implementation to address real and perceived safety concerns, respect diverse cultural esthetics, and ensure equitable access, avoiding one-size-fits-all greening solutions (see safety and security barriers in GBI implementation, balancing climate adaptation and esthetic goals in urban GBI, and land scarcity and urban sprawl). This recommendation is directed at landscape architects and urban designers, ensuring the balance between ecological function and esthetics to guide GBI implementation to align with esthetics and equity.

Strengthen the financial ecosystem through standardized ESG metrics and monitoring. Prioritize the development and global adoption of resilient, standardized ESG metrics and risk valuation frameworks, leveraging emerging technologies such as AI, EO, and satellite data. Reliable and comparable data will strengthen GBI valuation, support better risk assessment, and attract sustainable investments (see financial barriers to GBI implementation and challenges in recognizing GBI as assets). This recommendation calls on investors, financial institutions, and local governments to adopt robust and standardized ESG metrics, providing reliable data for valuation, risk assessment, and sustainable investment.

Scale innovative financing mechanisms for GBI deployment. Expand the use of green/blue bonds, and debt-for-nature swaps by creating clear, accessible frameworks for environmental and social impact evaluation. Third-party certifications and structured de-risking mechanisms will help build investor confidence and close the biodiversity financing gap (see lack of comprehensive cost-benefit analysis and conflicts in promoting GBI and walkability). This recommendation urges financial institutions and developers to expand access to GBI through frameworks that evaluate and communicate environmental and social impacts.

Align financial regulation and incentives with biodiversity and natural capital goals. Incorporate ecological risks into finance regulations and monetary and fiscal policies. Coupled with incentives such as tax benefits and blended finance models, this will encourage larger and more stable investments in GBI (see financial barriers to GBI implementation, challenges in recognizing GBI as assets, and lack of comprehensive cost-benefit analysis). This recommendation highlights the need for policymakers, local governments, and investors to align fiscal and regulatory instruments with biodiversity protection and natural capital objectives.

Prioritize micro-scale, three-dimensional GBI to optimize urban land use. Support micro-scale, three-dimensional GBI, including rooftop greenery, vertical gardens, and pocket parks to promote ecological diversity and livability in space-constrained urban areas. Ensure integration into city sustainability plans with long-term maintenance strategies (see land scarcity and urban sprawl). This recommendation is aimed at urban planners, architects, and developers, promoting compact three-dimensional solutions such as green roofs, vertical gardens, and pocket parks.

Embed GBI in urban planning through adaptive governance and policy incentives. Position GBI as a core element of urban planning rather than a supplementary feature. Implement integrated spatial planning and foster collaboration across sectors and disciplines. Adopt adaptive governance structures that offer clear incentives for ecological design. Ensure GBI aligns with economic, social, and environmental objectives to support resilient, inclusive cities (see urban design barriers, lack of clear GBI implementation policies, and conflicts in promoting GBI and walkability). This recommendation emphasizes the role of local governments, policymakers, and planners in embedding GBI as core infrastructure through adaptive governance and policy frameworks that integrate economic, social, and environmental priorities.

This synthesized framework advances GBI implementation by connecting theory with practice, enabling a shift from standardized contextually responsive models that reflect local conditions across environmental, social, economic, and governance dimensions. Interdisciplinary collaboration is essential to align diverse stakeholder priorities, supported by long-term monitoring to assess performance and trade-offs. Adaptive governance must reflect complex urban dynamics. The future of GBI lies in science-driven, justice-oriented approaches, transitioning from isolated, esthetic interventions to multifunctional, context-sensitive systems. As climate pressures grow, integration with PVs and data-driven monitoring will enhance energy resilience and urban cooling. Equity, inclusion, and participatory governance must underpin implementation, while embedding natural capital in policy and finance is key to scaling investment and achieving climate-resilient, inclusive urban futures.

FUNDING AND ACKNOWLEDGMENTS

This work is carried out under the framework of UKRI (EPSRC, NERC, AHRC) funded RECLAIM Network Plus (EP/W034034/1; EP/W033984/1) and GP4Streets (UKRI1281) projects. P.K. and co-authors acknowledge the support received through the UKRI-funded GreenCities (NE/X002799/1, NE/X002772/1, FAPESP grant nos. 2016/18438-0, 2019/08783-0, 2022/02365-5, 2022/04619-4, 2024/01097-2, 2024/23425-1, and 25/03337-3), GREENIN Micro Network Plus (APP55977), DEFRAG (NE/W002892/1) and UGPN-funded (UGPN-NBS and GREENICON) projects. All the co-authors contributed equally, and their names are listed in alphabetical order following the core writing team. The funders had

www.the-innovation.org

no role in study design, data collection and analysis, decision to publish, or preparation of the manuscript.

AUTHOR CONTRIBUTIONS

Conceptualization, P.K., methods, P.K., K.C.P., A.B., H.S., and A.K.D.; supervision, P.K.; project administration, P.K.; funding, P.K.; data analysis (figures, tables), P.K., K.C.P., A.B., H.S., and A.K.D.; data extraction, K.C.P., A.B., H.S., and A.K.D.; writing – original draft, P.K., K.C.P., A.B., H.S., A.K.D., S.H., and S.K.; writing – review & editing, P.K., K.C.P., A.B., H.S., A.K.D., S.H., S.K., A.A., M.d.F.A., R.A.A., E.A.A.d.S., M.A., C.B.R., P.B., M.L.B., B.G.B., L.F.C., S.-J.C., A.L.C.F.G., R.C., A.K.C.R., B.C., R.M.d.M., L.A.d.P., P.d.S., M.A.F., E.D.F., M.F.F., B.F., J.G., L.L.G., M.J.G.R., C.H.H., WF.H., L.H., C.H., Y.H., L.J., R.J., J.K., M.K., G.M.L., A.A.L.M., J.A.M., L.D.M., M.C.M., R.C.K.M., Y.K.L.K., W.L.A., J.L., G.M.M., S.K.M., M.P.M., M.C.V.M.S., A.Mc., O.M.S., E.M., E.G.S.N., T.N., G.O., R.P., H.P.P., R.P., P.J.P.M., J.A.P., S.P., J.A.P.S., P.L.R.A., N.C.R., A.P.R., D.S., Y.S., V.S., Y.T., T.T.d.A.A., B.L.V.M., F.W., J.W., C.W., H.S.W., Q.W., R.W., C.X., R.Y., and R.Y. The authors' names appear in alphabetical order after the core writing team. All authors commented on the draft manuscript and assisted in the conceptual development of the text, tables, figures, and the overall cohesiveness and proofreading of the paper.

DECLARATION OF INTERESTS

The authors declare no competing interests.

SUPPLEMENTAL INFORMATION

It can be found online at https://doi.org/10.1016/j.xinn.2025.101184.

LEAD CONTACT WEBSITE

https://www.surrey.ac.uk/people/prashant-kumar and www.surrey.ac.uk/gcare.

REFERENCES

1. United Nations Department of Economic and Social Affairs (2019). World Urbanization Prospects: The 2018 Revision. Statistical Papers - United Nations (Ser. A), Population and Vital Statistics Report. DOI:10.18356/b9e995fe-en
2. Yang, W., Zhang, J., Hua, P. et al. (2023). Global framework for flood risk management under climate change and urbanization. *Innov. Geosci.* **1**:100009. DOI:10.59717/j.xinn-geo.2023.100009
3. Yin, J. and Slater, L. (2023). Understanding heatwave-drought compound hazards and impacts on socio-ecosystems. *Innov. Geosci.* **1**:100042. DOI:10.59717/j.xinn-geo.2023.100042
4. Kumar, P., Corada, K., Debele, S.E. et al. (2024). Air pollution abatement from Green-Blue-Grey infrastructure. *Innov. Geosci.* **2**:100100. DOI:10.59717/j.xinn-geo.2024.100100
5. Kumar, P., Debele, S.E., Khalili, S. et al. (2024). Urban heat mitigation by green and blue infrastructure: Drivers, effectiveness, and future needs. *Innov* **5**:100588. DOI:10.1016/j.xinn.2024.100588
6. Jones, L., Anderson, S., Læssøe, J. et al. (2022). A typology for urban Green Infrastructure to guide multifunctional planning of nature-based solutions. *Nat.-Based Solut.* **2**:100041. DOI:10.1016/j.nbsj.2022.100041
7. Nieuwenhuijsen, M.J., Khreis, H., Triguero-Mas, M. et al. (2017). Fifty shades of green: pathway to healthy urban living. *Epidemiology* **28**:63–71. DOI:10.1097/ede.0000000000000549
8. Kumar, P., Sahani, J., Corada, K. et al. (2025). Urban greening for climate resilient and sustainable cities: Grand challenges and opportunities. *Front. Sustain. Cities* **7**:1595280. DOI:10.3389/frsc.2025.1595280
9. Mueller, N., Rojas-Rueda, D., Basagaña, X. et al. (2017). Urban and transport planning related exposures and mortality: a health impact assessment for cities. *Environ. Health Perspect.* **125**:89–96. DOI:10.1289/EHP220
10. Nguyen Thi Khanh, H., Rigau-Sabadell, M., Khomenko, S. et al. (2025). Ambient air pollution, urban green space and childhood overweight and obesity: A health impact assessment for Barcelona, Spain. *Environ. Res.* **264**:120306. DOI:10.1016/j.envres.2024.120306
11. Triguero-Mas, M., Dadvand, P., Cirach, M. et al. (2015). Natural outdoor environments and mental and physical health: relationships and mechanisms. *Environ. Int.* **77**:35–41. DOI:10.1016/j.envint.2015.01.012
12. Dadvand, P., Nieuwenhuijsen, M.J., Esnaola, M. et al. (2015). Green spaces and cognitive development in primary schoolchildren. *Proc. Natl. Acad. Sci. USA* **112**:7937–7942. DOI:10.1073/pnas.1503402112
13. Wolch, J.R., Byrne, J. and Newell, J.P. (2014). Urban green space, public health, and environmental justice: The challenge of making cities 'just green enough'. *Landsc. Urban Plann.* **125**:234–244. DOI:10.1016/j.landurbplan.2014.01.017
14. Berland, A., Shiflett, S.A., Shuster, W.D. et al. (2017). The role of trees in urban stormwater management. *Landsc. Urban Plann.* **162**:167–177. DOI:10.1016/j.landurbplan.2017.02.017
15. Donovan, G.H. and Butry, D.T. (2009). The value of shade: Estimating the effect of urban trees on summertime electricity use. *Energy Build.* **41**:662–668. DOI:10.1016/j.enbuild.2009.01.002
16. Cohen-Shacham, E., Walters, G., Janzen, C. et al. (2016). Nature-based Solutions to Address Global Societal Challenges (International Union for Conservation of Nature (IUCN)). DOI:10.2305/IUCN.CH.2016.13.en
17. IUCN (2020). IUCN Global Standard for Nature-Based Solutions: A User-Friendly Framework for the Verification, Design and Scaling up of NbS, First edition (International Union for Conservation of Nature (IUCN)). https://portals.iucn.org/library/sites/library/files/documents/2020-020-En.pdf
18. England, N. (2023). NECR500 People and Nature Survey Analysis 2023 (NECR500). https://publications.naturalengland.org.uk/publication/6549867611291648
19. Sokolova, M.V., Fath, B.D., Grande, U. et al. (2024). The role of green infrastructure in providing urban ecosystem services: Insights from a bibliometric perspective. *Land* **13**:1664. DOI:10.3390/land13101664
20. Cheng, X., Van Damme, S. and Uyttenhove, P. (2021). A review of empirical studies of cultural ecosystem services in urban green infrastructure. *J. Environ. Manage.* **293**:112895. DOI:10.1016/j.jenvman.2021.112895
21. O'Brien, L. and Reeder, M.J. (2017). Southern Hemisphere summertime Rossby waves and weather in the Australian region. *Q. J. R. Meteorol. Soc.* **143**:2374–2388. DOI:10.1002/qj.3090
22. Dimouli, I., Koumparou, D. and Golfinopoulos, S.K. (2024). From School Gardens to Community Oases: Fostering Environmental and Social Resilience in Urban Spaces. *Geographies* **4**:687–712. DOI:10.3390/geographies4040038
23. Li, J., Xu, H., Ren, M. et al. (2024). Knowledge Mapping of Cultural Ecosystem Services Applied on Blue-Green Infrastructure—A Scientometric Review with CiteSpace. *Forests* **15**:1736. DOI:10.3390/f15101736
24. Commission E (2013). Green Infrastructure: Promoting the Use of Green Infrastructure in All EU Policies, to Help Restore Nature and Boost Biodiversity (European Commission). https://environment.ec.europa.eu/topics/nature-and-biodiversity/green-infrastructure_en
25. Commission, E. (2020). The Habitats Directive: EU measures to conserve Europe's wild flora and fauna. European Commission. https://environment.ec.europa.eu/topics/nature-and-biodiversity/habitats-directive_en
26. Jones, L., Reis, S., Hutchins, M. et al. (2022). Airsheds, watersheds and more–The flows that drive intra-extra-urban connections, and their implications for nature-based solutions (NBS). *Nat.-Based Solut.* **2**:100040. DOI:10.1016/j.nbsj.2022.100040
27. Wang, X., Hu, Q., Zhang, R. et al. (2025). Ecosystem Services in Urban Blue-Green Infrastructure: A Bibliometric Review. *Water* **17**:2273. DOI:10.3390/w17152273
28. Zarei, M. and Shahab, S. (2025). Nature-Based Solutions in Urban Green Infrastructure: A Systematic Review of Success Factors and Implementation Challenges. *Land* **14**:818. DOI:10.3390/land14040818
29. Chau, H.-W., Abuseif, M., Geng, S. et al. (2025). Key Barriers and Challenges to Green Infrastructure Implementation: Policy Insights from the Melbourne Case. *Land* **14**:961. DOI:10.3390/land14050961
30. Seidu, S., Chan, D.W.M. and Taiwo, R. (2025). Integrating green and grey infrastructure systems in dense urban regions: a synthesis of critical barriers and effective implementation guidelines. *Clean Technol. Environ.* 1–22. DOI:10.1007/s10098-025-03309-3
31. Kim, J.-P. and Kim, J.-O. (2025). Green Infrastructure for Urban Flooding: Knowledge Domains and Research Evolution (2015–2024). *Land* **14**:921. DOI:10.3390/land14050921
32. Tao, Z., Sun, H., Deng, B. et al. (2025). Integrating computational fluid dynamics (CFD) and machine learning to improve urban green infrastructure for heat mitigation and air quality: A systematic review. *Build. Environ.* **284**:113516. DOI:10.1016/j.buildenv.2025.113516
33. Dobrinić, D., Miler, M. and Medak, D. (2025). Mapping the Green Urban: A Comprehensive Review of Materials and Learning Methods for Green Infrastructure Mapping. *Sensors* **25**:464. DOI:10.3390/s25020464
34. Li, Y., Zhai, W., Huang, H. et al. (2025). A systematic review of nature-based solutions to urban hazards through the lens of environmental justice. *Hum. Settlem. Sustain.* **1**:1–14. DOI:10.1016/j.hssust.2024.12.002
35. Muñoz, L.S. and Duarte, D.H.S. (2025). Green infrastructure as a planning tool: A comprehensive systematization of urban redesign strategies to increase vegetation within public places. *Cities* **156**:105551. DOI:10.1016/j.cities.2024.105551
36. Sobhaninia, S., Meerow, S., Dugger, A. et al. (2025). Where should the green go? A systematic literature review of methods for siting green infrastructure to mitigate rising heat and stormwater risks in cities worldwide. *Urban For. Urban Green.* **107**:128790. DOI:10.1016/j.ufug.2025.128790
37. Khalili, S., Kumar, P. and Jones, L. (2024). Evaluating the benefits of urban green infrastructure: Methods, indicators, and gaps. *Heliyon* **10**:e38446. DOI:10.1016/j.heliyon.2024.e38446
38. Perera, A.C.S., Davies, P.J. and Graham, P.L. (2024). A global review of urban blue-green planning tools. *Land Use Policy* **140**:107093. DOI:10.1016/j.landusepol.2024.107093
39. Przestrzelska, K., Wartalska, K., Rosińska, W. et al. (2024). Climate resilient cities: a review of blue-green solutions worldwide. *Water Resour. Manag.* **38**:5885–5910. DOI:10.1007/s11269-024-03950-5
40. Tate, C., Tran, N., Longo, A. et al. (2024). Economic evaluations of urban green and blue space interventions: A scoping review. *Ecol. Econ.* **222**:108217. DOI:10.1016/j.ecolecon.2024.108217
41. Debele, S.E., Leo, L.S., Kumar, P. et al. (2023). Nature-based solutions can help reduce the impact of natural hazards: A global analysis of NBS case studies. *Sci. Total Environ.* **902**:165824. DOI:10.1016/j.scitotenv.2023.165824

42. de Quadros, B.M. and Mizgier, M.G.O. (2023). Urban green infrastructures to improve pedestrian thermal comfort: A systematic review. *Urban For. Urban Green.* **88**:128091. DOI:10.1016/j.ufug.2023.128091
43. Li, L. and Lange, K.W. (2023). Assessing the relationship between urban blue-green infrastructure and stress resilience in real settings: A systematic review. *Sustainability* **15**:9240. DOI:10.3390/su15129240
44. Potter, J.D., Brooks, C., Donovan, G. et al. (2023). A perspective on green, blue, and grey spaces, biodiversity, microbiota, and human health. *Sci. Total Environ.* **892**:164772. DOI:10.1016/j.scitotenv.2023.164772
45. Adnan, M.S.G., Dewan, A., Botje, D. et al. (2022). Vulnerability of Australia to heatwaves: A systematic review on influencing factors, impacts, and mitigation options. *Environ. Res.* **213**:113703. DOI:10.1016/j.envres.2022.113703
46. Evans, D.L., Falagán, N., Hardman, C.A. et al. (2022). Ecosystem service delivery by urban agriculture and green infrastructure–a systematic review. *Ecosyst. Serv.* **54**:101405. DOI:10.1016/j.ecoser.2022.101405
47. Almaaitah, T., Appleby, M., Rosenblat, H. et al. (2021). The potential of Blue-Green infrastructure as a climate change adaptation strategy: a systematic literature review. *Blue-Green Syst.* **3**:223–248. DOI:10.2166/bgs.2021.016
48. Choi, C., Berry, P. and Smith, A. (2021). The climate benefits, co-benefits, and trade-offs of green infrastructure: A systematic literature review. *J. Environ. Manage.* **291**:112583. DOI:10.1016/j.jenvman.2021.112583
49. Kumar, P., Debele, S.E., Sahani, J. et al. (2021). An overview of monitoring methods for assessing the performance of nature-based solutions against natural hazards. *Earth Sci. Rev.* **217**:103603. DOI:10.1016/j.earscirev.2021.103603
50. Kumar, P., Debele, S.E., Sahani, J. et al. (2021). Nature-based solutions efficiency evaluation against natural hazards: Modelling methods, advantages and limitations. *Sci. Total Environ.* **784**:147058. DOI:10.1016/j.scitotenv.2021.147058
51. Toxopeus, H. and Polzin, F. (2021). Reviewing financing barriers and strategies for urban nature-based solutions. *J. Environ. Manage.* **289**:112371. DOI:10.1016/j.jenvman.2021.112371
52. Veerkamp, C.J., Schipper, A.M., Hedlund, K. et al. (2021). A review of studies assessing ecosystem services provided by urban green and blue infrastructure. *Ecosyst. Serv.* **52**:101367. DOI:10.1016/j.ecoser.2021.101367
53. Kumar, P., Debele, S.E., Sahani, J. et al. (2020). Towards an operationalisation of nature-based solutions for natural hazards. *Sci. Total Environ.* **731**:138855. DOI:10.1016/j.scitotenv.2020.138855
54. Shah, M.A.R., Renaud, F.G., Anderson, C.C. et al. (2020). A review of hydro-meteorological hazard, vulnerability, and risk assessment frameworks and indicators in the context of nature-based solutions. *Int. J. Disaster Risk Reduct.* **50**:101728. DOI:10.1016/j.ijdrr.2020.101728
55. Ying, J., Zhang, X., Zhang, Y. et al. (2021). Green infrastructure: Systematic literature review. *Econ. Res.* **35**:343–366. DOI:10.1080/1331677X.2021.1893202
56. Debele, S.E., Kumar, P., Sahani, J. et al. (2019). Nature-based solutions for hydro-meteorological hazards: Revised concepts, classification schemes and databases. *Environ. Res.* **179**:108799. DOI:10.1016/j.envres.2019.108799
57. Ruan, T., Xu, Y., Jones, L. et al. (2023). Green infrastructure sustains the food-energy-water-habitat nexus. *Sustain. Cities Soc.* **98**:104845. DOI:10.1016/j.scs.2023.104845
58. Meng, F., Yuan, Q., Bellezoni, R.A. et al. (2023). Quantification of the food-water-energy nexus in urban green and blue infrastructure: A synthesis of the literature. *Resour. Conserv. Recycl.* **188**:106658. DOI:10.1016/j.resconrec.2022.106658
59. Bellezoni, R.A., Meng, F., He, P. et al. (2021). Understanding and conceptualizing how urban green and blue infrastructure affects the food, water, and energy nexus: A synthesis of the literature. *J. Clean. Prod.* **289**:125825. DOI:10.1016/j.jclepro.2021.125825
60. Venkataramanan, V., Packman, A.I., Peters, D.R. et al. (2019). A systematic review of the human health and social well-being outcomes of green infrastructure for stormwater and flood management. *J. Environ. Manage.* **246**:868–880. DOI:10.1016/j.jenvman.2019.05.028
61. O'Brien, L., De Vreese, R., Kern, M. et al. (2017). Cultural ecosystem benefits of urban and peri-urban green infrastructure across different European countries. *Urban For. Urban Green.* **24**:236–248. DOI:10.1016/j.ufug.2017.03.002
62. Kumar, P., Sahani, J., Corada Perez, K. et al. (2025). Urban greening for climate resilient and sustainable cities: grand challenges and opportunities. *Front. Sustain. Cities* **7**:1595280. DOI:10.3389/frsc.2025.1595280
63. Caroppi, G., Pugliese, F., Gerundo, C. et al. (2024). A comprehensive framework tool for performance assessment of NBS for hydro-meteorological risk management. *J. Environ. Plan. Manag.* **67**:1231–1257. DOI:10.1080/09640568.2023.2166818
64. Shah, S.I.H., Navvazz, R., Ahmad, S. et al. (2020). Sustainability assessment of modern urban transport and its role in the reduction of greenhouse gas emissions: A case study of Metro Bus System (MBS), Lahore. *Kuwait J Sci* **47**:67–81. https://journalskuwait.org/kjs/index.php/KJS/article/view/5407
65. Rehman, K.U., Liu, X., Wang, H. et al. (2018). Effects of black soldier fly biodiesel blended with diesel fuel on combustion, performance and emission characteristics of diesel engine. *Energy Convers. Manag.* **173**:489–498. DOI:10.1016/j.enconman.2018.07.102
66. Scott, A. and Hislop, M. (2024). What does good green and blue infrastructure policy look like: A comparative assessment of UK national planning guidance. *Urban For. Urban Green.* **99**:128440. DOI:10.1016/j.ufug.2024.128440
67. Jones, L., Anderson, S., Læssøe, J. et al. (2025). Re-thinking people and nature interactions in urban nature-based solutions. *Sustainability* **17**:3043. DOI:10.3390/su17073043
68. Xiao, H., Xu, Z., Ren, J. et al. (2022). Navigating Chinese cities to achieve sustainable development goals by 2030. *Innov* **3**:100288. DOI:10.1016/j.xinn.2022.100288
69. RECLAIM (2025). Third RECLAIM Network Plus Conference. https://www.youtube.com/watch?v=ekK0gCXmV5s&list=PL4xPfJG-Mbo4LvhL3rtVptHETgLU6w0GX&index=69
70. Deely, J., Hynes, S., Barquín, J. et al. (2020). Barrier identification framework for the implementation of blue and green infrastructures. *Land Use Policy* **99**:105108. DOI:10.1016/j.landusepol.2020.105108
71. Purvis, B., Mao, Y. and Robinson, D. (2019). Three pillars of sustainability: in search of conceptual origins. *Sustain. Sci.* **14**:681–695. DOI:10.1007/s11625-018-0627-5
72. Zhang, H., Yu, Z., Zhu, C. et al. (2023). Green or not? Environmental challenges from photovoltaic technology. *Environ. Pollut.* **320**:121066. DOI:10.1016/j.envpol.2023.121066
73. Hernandez, R.R., Easter, S.B., Murphy-Mariscal, M.L. et al. (2014). Environmental impacts of utility-scale solar energy. *Renew. Sustain. Energy Rev.* **29**:766–779. DOI:10.1016/j.rser.2013.08.041
74. Dhar, A., Naeth, M.A., Jennings, P.D. et al. (2020). Perspectives on environmental impacts and a land reclamation strategy for solar and wind energy systems. *Sci. Total Environ.* **718**:134602. DOI:10.1016/j.scitotenv.2019.134602
75. Kruitwagen, L., Story, K.T., Friedrich, J. et al. (2021). A global inventory of photovoltaic solar energy generating units. *Nature* **598**:604–610. DOI:10.1038/s41586-021-03957-7
76. AussieSolarTech (2024). How Trees Affect Solar Panels: The Shady Impact. https://aussiesolartech.com.au/solar-panel/how-trees-affect-solar-panels/
77. Zhong, Q. and Tong, D. (2020). Spatial layout optimization for solar photovoltaic (PV) panel installation. *Renew. Energy* **150**:1–11. DOI:10.1016/j.renene.2019.12.099
78. Anders, S., Day, T. and Kuduk, C.A. (2010). "Hey, Your Tree Is Shading My Solar Panels": California's Solar Shade Control Act. *J. Sustain. Real Estate* **2**:361–381. DOI:10.1080/10835547.2010.12091808
79. Domke, G.M., Oswalt, S.N., Walters, B.F. et al. (2020). Tree planting has the potential to increase carbon sequestration capacity of forests in the United States. *Proc. Natl. Acad. Sci. USA* **117**:24649–24651. DOI:10.1073/pnas.2010840117
80. Liu, Y., Han, B., Jiang, C.Q. et al. (2024). Uncovering the role of urban green infrastructure in carbon neutrality: A novel pathway from the urban green infrastructure and cooling power saving. *J. Clean. Prod.* **452**:142193. DOI:10.1016/j.jclepro.2024.142193
81. Reitberger, R., Kooniyara, V.P., Parhizgar, L. et al. (2025). Tree growth simulation in Geographic Information Systems: Coupling CityTree and ArcGIS for solar radiation analysis. *Sustain. Cities Soc.* **120**:106128. DOI:10.1016/j.scs.2025.106128
82. Service, U.F. (2023). i-Tree Eco. https://www.itreetools.org/tools/i-tree-eco
83. Liu, C.N., Lin, F.C., Manga, M. et al. (2023). Thumping Cycle Variations of Doublet Pool in Yellowstone National Park, USA. *Geophys. Res. Lett.* **50**:e2022GL101175. DOI:10.1029/2022gl101175
84. Skandalos, N. and Karamanis, D. (2025). Decarbonizing operational emissions in urban neighborhoods with the integration of rooftop photovoltaics and green infrastructure under current and future climate conditions. *Energy Build.* **329**:115306. DOI:10.1016/j.enbuild.2025.115306
85. Grossi, F., Ge, H., Zmeureanu, R. et al. (2022). Feasibility of planting trees around buildings as a nature-based solution of carbon sequestration—An LCA approach using two case studies. *Buildings* **13**:41. DOI:10.3390/buildings13010041
86. Tinsley, E., Froidevaux, J.S.P. and Jones, G. (2024). The location of solar farms within England's ecological landscape: Implications for biodiversity conservation. *J. Environ. Manage.* **372**:123372. DOI:10.1016/j.jenvman.2024.123372
87. Wang, W., Yang, H. and Xiang, C. (2023). Green roofs and facades with integrated photovoltaic system for zero energy eco-friendly building–A review. *Sustain. Energy Technol. Assess.* **60**:103426. DOI:10.1016/j.seta.2023.103426
88. Almutairi, K., Esfahani, E.M., Mostafaeipour, A. et al. (2021). A Novel Policy to Optimize Energy Consumption for Dairy Product Warehouses: A Case Study. *Sustainability* **13**:2445. DOI:10.3390/su13052445
89. Arenandan, V., Wong, J.K., Ahmed, A.N. et al. (2022). Efficiency enhancement in energy production of photovoltaic modules through green roof installation under tropical climates. *Ain Shams Eng. J.* **13**:101741. DOI:10.1016/j.asej.2022.101741
90. Baumann, T., Nussbaumer, H., Klenk, M. et al. (2019). Photovoltaic systems with vertically mounted bifacial PV modules in combination with green roofs. *Sol. Energy* **190**:139–146. DOI:10.1016/j.solener.2019.08.014
91. Essak, L. and Ghosh, A. (2022). Floating photovoltaics: A review. *Clean Technol.* **4**:752–769. DOI:10.3390/cleantechnol4030046
92. Chong, C. (2021). Singapore Now Home to One of the World's Largest Floating Solar Farms (The Straits Times). https://www.straitstimes.com/singapore/singapore-now-home-to-one-of-the-worlds-largest-floating-solar-farms
93. Oliveira, P.M.B., Almeida, R.M. and Cardoso, S.J. (2024). Effects of floating photovoltaics on aquatic organisms: a review. *Hydrobiologia* **852**:3155–3170. DOI:10.1007/s10750-024-05686-0
94. Rodriguez-Burgos, A.M., Briceno-Zuluaga, F.J., Jimenez, J.L.A. et al. (2022). The impact of climate change on the distribution of Sphyrna lewini in the tropical eastern Pacific. *Mar. Environ. Res.* **180**:105696. DOI:10.1016/j.marenvres.2022.105696
95. Leischow, S.J., Best, A., Trochim, W.M. et al. (2008). Systems thinking to improve the public's health. *Am. J. Prev. Med.* **35**:S196–S203. DOI:10.1016/j.amepre.2008.05.014
96. Peng, J., Zhao, H. and Liu, Y. (2017). Urban ecological corridors construction: A review. *Acta Ecol. Sin.* **37**:23–30. DOI:10.1016/j.chnaes.2016.12.002
97. Tian, P., Li, J., Cao, L. et al. (2021). Assessing spatiotemporal characteristics of urban heat islands from the perspective of an urban expansion and green infrastructure. *Sustain. Cities Soc.* **74**:103208. DOI:10.1016/j.scs.2021.103208

98. Liu, Z., He, C. and Wu, J. (2016). The relationship between habitat loss and fragmentation during urbanization: an empirical evaluation from 16 world cities. *PLoS One* **11**:e0154613. DOI:10.1371/journal.pone.0154613
99. Yin, J., Wei, Q., Shao, D. et al. (2025). The impacts of power transmission and transformation projects on ecological corridors and landscape connectivity: a case study of Shandong province, China. *Sci. Rep.* **15**:6709. DOI:10.1038/s41598-025-91474-2
100. Kowe, P., Mutanga, O. and Dube, T. (2021). Advancements in the remote sensing of landscape pattern of urban green spaces and vegetation fragmentation. *Int. J. Remote Sens.* **42**:3797–3832. DOI:10.1080/01431161.2021.1881185
101. Bierwagen, B.G. (2007). Connectivity in urbanizing landscapes: The importance of habitat configuration, urban area size, and dispersal. *Urban Ecosyst.* **10**:29–42. DOI:10.1007/S11252-006-0011-6
102. Estoque, R.C. and Murayama, Y. (2017). Monitoring surface urban heat island formation in a tropical mountain city using Landsat data (1987–2015). *ISPRS J. Photogramm. Remote Sens.* **133**:18–29. DOI:10.1016/j.isprsjprs.2017.09.008
103. Moyer, J.M. and Raheem, A.A. (2020). Study of past and future spatiotemporal patterns and impact on electricity consumption for sustainable planning: A case study of El Paso, Texas. *Sustainability* **12**:8480. DOI:10.3390/su12208480
104. Ahmadi Mirghaed, F., Mohammadzadeh, M., Salmanmahiny, A. et al. (2020). Assessing the interactions between landscape aesthetic quality and spatial indices in Gharasoo watershed, North of Iran. *Int. J. Environ. Sci. Technol.* **17**:231–242. DOI:10.1007/s13762-019-02342-2
105. Bertram, C., Goebel, J., Krekel, C. et al. (2022). Urban land use fragmentation and human well-being. *Land Econ.* **98**:399–420. DOI:10.3368/le.98.2.122019-0175r1
106. Kundu, S., Rana, N.K. and Mahato, S. (2024). Unravelling blue landscape fragmentation effects on ecosystem services in urban agglomerations. *Sustain. Cities Soc.* **102**:105192. DOI:10.1016/j.scs.2024.105192
107. Wong, C.P., Jiang, B., Kinzig, A.P. et al. (2018). Quantifying multiple ecosystem services for adaptive management of green infrastructure. *Ecosphere* **9**:e02495. DOI:10.1002/ecs2.2495
108. Tao, Q., Gao, G., Xi, H. et al. (2022). An integrated evaluation framework for multiscale ecological protection and restoration based on multi-scenario trade-offs of ecosystem services: Case study of Nanjing City, China. *Ecol. Indic.* **140**:108962. DOI:10.1016/j.ecolind.2022.108962
109. Larrea, V., Pelaez, F. and Esenarro, D. (2024). Design of a Green Corridor and the Revitalization of the Huatanay River, City of Cuzco, Peru—2024. *Urban Sci.* **8**:185. DOI:10.3390/urbansci8040185
110. Mancilla, D., Robledo, S., Esenarro, D. et al. (2024). Green Corridors and Social Connectivity with a Sustainable Approach in the City of Cuzco in Peru. *Urban Sci.* **8**:79. DOI:10.3390/urbansci8030079
111. Isola, F., Lai, S., Leone, F. et al. (2024). Urban Green Infrastructure and Ecosystem Service Supply: A Study Concerning the Functional Urban Area of Cagliari, Italy. *Sustainability* **16**:8628. DOI:10.3390/su16198628
112. Bruins, R.J., Canfield, T.J., Duke, C. et al. (2017). Using ecological production functions to link ecological processes to ecosystem services. *Integr. Environ. Assess. Manag.* **13**:52–61. DOI:10.1002/ieam.1842
113. Calfapietra, C., Fares, S., Manes, F. et al. (2013). Role of Biogenic Volatile Organic Compounds (BVOC) emitted by urban trees on ozone concentration in cities: A review. *Environ. Pollut.* **183**:71–80. DOI:10.1016/j.envpol.2013.03.012
114. Sierra-Heredia, C., North, M., Brook, J. et al. (2018). Aeroallergens in Canada: distribution, public health impacts, and opportunities for prevention. *Int. J. Environ. Res. Public Health* **15**:1577. DOI:10.3390/ijerph15081577
115. Sousa-Silva, R., Smargiassi, A., Kneeshaw, D. et al. (2021). Strong variations in urban allergenicity riskscapes due to poor knowledge of tree pollen allergenic potential. *Sci. Rep.* **11**:10196. DOI:10.1038/s41598-021-89353-7
116. Janhäll, S. (2015). Review on urban vegetation and particle air pollution–Deposition and dispersion. *Atmos. Environ.* **105**:130–137. DOI:10.1016/j.atmosenv.2015.01.052
117. Abhijith, K.V., Kumar, P., Gallagher, J. et al. (2017). Air pollution abatement performances of green infrastructure in open road and built-up street canyon environments–A review. *Atmos. Environ. X.* **162**:71–86. DOI:10.1016/j.atmosenv.2017.05.014
118. Li, Z., Kong, L., Hu, L. et al. (2024). Greenhouse gas emissions from constructed wetlands: A bibliometric analysis and mini-review. *Sci. Total Environ.* **906**:167582. DOI:10.1016/j.scitotenv.2023.167582
119. Yuan, Q., Lian, J., Yang, F. et al. (2025). A systematic review on greenhouse gas emissions from constructed wetlands: Focusing on effects of planting strategies and emission reduction measures. *J. Water Process Eng.* **69**:106696. DOI:10.1016/j.jwpe.2024.106696
120. Barwise, Y. and Kumar, P. (2020). Designing vegetation barriers for urban air pollution abatement: A practical review for appropriate plant species selection. *npj Clim. Atmos. Sci.* **3**:12. DOI:10.1038/s41612-020-0115-3
121. Maison, A., Lugon, L., Park, S.-J. et al. (2024). Contrasting effects of urban trees on air quality: From the aerodynamic effects in streets to impacts of biogenic emissions in cities. *Sci. Total Environ.* **946**:174116. DOI:10.1016/j.scitotenv.2024.174116
122. Zhang, S., Lyu, Y., Yang, X. et al. (2022). Modeling biogenic volatile organic compounds emissions and subsequent impacts on ozone air quality in the Sichuan Basin, southwestern China. *Front. Ecol. Evol.* **10**:924944. DOI:10.3389/fevo.2022.924944
123. Gu, S., Guenther, A. and Faiola, C. (2021). Effects of anthropogenic and biogenic volatile organic compounds on Los Angeles air quality. *Environ. Sci. Technol.* **55**:12191–12201. DOI:10.1021/acs.est.1c01481
124. Araya, M., Vera, J. and Préndez, M. (2025). Urban Tree Species Capturing Anthropogenic Volatile Organic Compounds—Impact on Air Quality. *Atmosphere* **16**:356. DOI:10.3390/atmos16040356
125. Cariñanos, P. and Casares-Porcel, M. (2011). Urban green zones and related pollen allergy: A review. Some guidelines for designing spaces with low allergy impact. *Landsc. Urban Plann.* **101**:205–214. DOI:10.1016/j.landurbplan.2011.03.006
126. Ravindra, K., Goyal, A. and Mor, S. (2022). Influence of meteorological parameters and air pollutants on the airborne pollen of city Chandigarh, India. *Sci. Total Environ.* **818**:151829. DOI:10.1016/j.scitotenv.2021.151829
127. Stawoska, I., Myszkowska, D., Oliwa, J. et al. (2023). Air pollution in the places of Betula pendula growth and development changes the physicochemical properties and the main allergen content of its pollen. *PLoS One* **18**:e0279826. DOI:10.1371/journal.pone.0279826
128. Zhang, Y. and Steiner, A.L. (2022). Projected climate-driven changes in pollen emission season length and magnitude over the continental United States. *Nat. Commun.* **13**:1234. DOI:10.1038/s41467-022-28764-0
129. Eisenman, T.S., Jariwala, S.P. and Lovasi, G.S. (2019). Urban trees and asthma: a call for epidemiological research. *Lancet Respir. Med.* **7**:e19–e20. DOI:10.1016/s2213-2600(19)30193-6
130. Browning, M.H.E.M., Rigolon, A., McAnirlin, O. et al. (2022). Where greenspace matters most: A systematic review of urbanicity, greenspace, and physical health. *Landsc. Urban Plann.* **217**:104233. DOI:10.1016/j.landurbplan.2021.104233
131. Hirschlag, A. (2020). How urban planners' preference for male trees has made your hay fever worse. *Guardian*. https://www.theguardian.com/environment/2020/may/16/how-urban-planners-preference-for-male-trees-has-made-your-hay-fever-worse
132. Venter, Z.S., Hassani, A., Stange, E. et al. (2024). Reassessing the role of urban green space in air pollution control. *Proc. Natl. Acad. Sci. USA* **121**:e2306200121. DOI:10.1073/pnas.2306200121
133. Fellini, S., Marro, M., Del Ponte, A.V. et al. (2022). High resolution wind-tunnel investigation about the effect of street trees on pollutant concentration and street canyon ventilation. *Build. Environ.* **226**:109763. DOI:10.1016/j.buildenv.2022.109763
134. Van Renterghem, T., Hornikx, M., Forssen, J. et al. (2013). The potential of building envelope greening to achieve quietness. *Build. Environ.* **61**:34–44. DOI:10.1016/j.buildenv.2012.12.001
135. Livesley, S.J., McPherson, G.M. and Calfapietra, C. (2016). The urban forest and ecosystem services: impacts on urban water, heat, and pollution cycles at the tree, street, and city scale. *J. Environ. Qual.* **45**:119–124. DOI:10.2134/jeq2015.11.0567
136. Pugh, T.A.M., MacKenzie, A.R., Whyatt, J.D. et al. (2012). Effectiveness of green infrastructure for improvement of air quality in urban street canyons. *Environ. Sci. Technol.* **46**:7692–7699. DOI:10.1021/es300826w
137. Nowak, D.J. and Crane, D.E. (2002). Carbon storage and sequestration by urban trees in the USA. *Environ. Pollut.* **116**:381–389. DOI:10.1016/s0269-7491(01)00214-7
138. Velasco, E., Roth, M., Norford, L. et al. (2016). Does urban vegetation enhance carbon sequestration? *Landsc. Urban Plann.* **148**:99–107. DOI:10.1016/j.landurbplan.2015.12.003
139. Saumel, I., Weber, F. and Kowarik, I. (2016). Toward livable and healthy urban streets: Roadside vegetation provides ecosystem services where people live and move. *Environ. Sci. Policy* **62**:24–33. DOI:10.1016/j.envsci.2015.11.012
140. Nowak, D.J., Greenfield, E.J., Hoehn, R.E. et al. (2013). Carbon storage and sequestration by trees in urban and community areas of the United States. *Environ. Pollut.* **178**:229–236. DOI:10.1016/j.envpol.2013.03.019
141. Barwise, Y., Kumar, P., Tiwari, A. et al. (2021). The co-development of HedgeDATE, a public engagement and decision support tool for air pollution exposure mitigation by green infrastructure. *Sustain. Cities Soc.* **75**:103299. DOI:10.1016/j.scs.2021.103299
142. Bush, J. and Doyon, A. (2019). Building urban resilience with nature-based solutions: How can urban planning contribute? *Cities* **95**:102483. DOI:10.1016/j.cities.2019.102483
143. Zuniga-Teran, A.A., Staddon, C., De Vito, L. et al. (2020). Challenges of mainstreaming green infrastructure in built environment professions. *J. Environ. Plan. Manag.* **63**:710–732. DOI:10.1080/09640568.2019.1605890
144. Wu, Q., Huang, Y., Irga, P. et al. (2024). Synergistic control of urban heat island and urban pollution island effects using green infrastructure. *J. Environ. Manage.* **370**:122985. DOI:10.1016/j.jenvman.2024.122985
145. Marando, F., Heris, M.P., Zulian, G. et al. (2022). Urban heat island mitigation by green infrastructure in European Functional Urban Areas. *Sustain. Cities Soc.* **77**:103564. DOI:10.1016/j.scs.2021.103564
146. Das, M., Das, A. and Momin, S. (2022). Quantifying the cooling effect of urban green space: A case from urban parks in a tropical mega metropolitan area (India). *Sustain. Cities Soc.* **87**:104062. DOI:10.1016/j.scs.2022.104062
147. Yang, M., Ren, C., Wang, H. et al. (2024). Mitigating urban heat island through neighboring rural land cover. *Nat. Cities* **1**:522–532. DOI:10.1038/s44284-024-00091-z
148. Rey-Gozalo, G., Barrigón Morillas, J.M., Montes, G.D. et al. (2023). Influence of green areas on the urban sound environment. *Curr. Pollut. Rep.* **9**:746–759. DOI:10.1007/s40726-023-00284-5
149. Kang, G. and Kim, J.-J. (2025). How to plant trees on an elevated road to improve thermal comfort in a street canyon. *Sustain. Cities Soc.* **121**:106207. DOI:10.2139/ssrn.4948133
150. Fletcher, D.H., Garrett, J.K., Thomas, A. et al. (2022). Location, location, location: Modelling of noise mitigation by urban woodland shows the benefit of targeted tree planting in cities. *Sustainability* **14**:7079. DOI:10.3390/su14127079

151. Zhao, N., Prieur, J.-F., Liu, Y. et al. (2021). Tree characteristics and environmental noise in complex urban settings–A case study from Montreal, Canada. *Environ. Res.* **202**:111887. DOI:10.1016/j.envres.2021.111887
152. Salmond, J.A., Williams, D.E., Laing, G. et al. (2013). The influence of vegetation on the horizontal and vertical distribution of pollutants in a street canyon. *Sci. Total Environ.* **443**:287–298. DOI:10.1016/j.scitotenv.2012.10.101
153. Buccolieri, R., Jeanjean, A.P.R., Gatto, E. et al. (2018). The impact of trees on street ventilation, NOx and PM2.5 concentrations across heights in Marylebone Rd street canyon, central London. *Sustain. Cities Soc.* **41**:227–241. DOI:10.1016/j.scs.2018.05.030
154. Li, Z., Zhang, H., Juan, Y.-H. et al. (2023). Effects of urban tree planting on thermal comfort and air quality in the street canyon in a subtropical climate. *SSRN Journal* **91**:104334. DOI:10.2139/ssrn.4150587
155. Back, Y., Jasper-Tönnies, A., Bach, P.M. et al. (2025). Current interventions are inadequate to maintain cities' resilience during concurrent drought and excessive heat. *Earths Future* **13**:e2024EF005208. DOI:10.1029/2024ef005208
156. Litvak, E., Manago, K.F., Hogue, T.S. et al. (2017). Evapotranspiration of urban landscapes in L os A ngeles, C alifornia at the municipal scale. *Water Resour. Res.* **53**:4236–4252. DOI:10.1002/2016wr020254
157. Van Mechelen, C., Dutoit, T. and Hermy, M. (2015). Adapting green roof irrigation practices for a sustainable future: A review. *Sustain. Cities Soc.* **19**:74–90. DOI:10.1016/j.scs.2015.07.007
158. Bauduin, T., Gypens, N. and Borges, A.V. (2024). Seasonal and spatial variations of greenhouse gas (CO2, CH4 and N2O) emissions from urban ponds in Brussels. *Water Res.* **253**:121257. DOI:10.1016/j.watres.2024.121257
159. Rosentreter, J.A., Borges, A.V., Deemer, B.R. et al. (2021). Half of global methane emissions come from highly variable aquatic ecosystem sources. *Nat. Geosci.* **14**:225–230. DOI:10.1038/s41561-021-00715-2
160. Holgerson, M.A. and Raymond, P.A. (2016). Large contribution to inland water CO2 and CH4 emissions from very small ponds. *Nat. Geosci.* **9**:222–226. DOI:10.1038/ngeo2654
161. Ottosen, T.-B. and Kumar, P. (2020). The influence of the vegetation cycle on the mitigation of air pollution by a deciduous roadside hedge. *Sustain. Cities Soc.* **53**:101919. DOI:10.1016/j.scs.2019.101919
162. Baldauf, R. (2017). Roadside vegetation design characteristics that can improve local, near-road air quality. *Transp. Res. D* **52**:354–361. DOI:10.1016/j.trd.2017.03.013
163. L Chen, C.M., Y, H. et al. (2024). Modelling the dispersion of traffic pollutants in a real-world street canyon with unevenly distributed trees. 24th Australasian Fluid Mechanics Conference. Paper No: AFMC2024-99. https://afmc2024.org/papers/non/AFMC2024_non_listed_paper_99.pdf
164. Turner, V.K., Middel, A. and Vanos, J.K. (2023). Shade is an essential solution for hotter cities. *Nature* **619**:694–697. DOI:10.1038/d41586-023-02311-3
165. van Bergen, T.J., Barros, N., Mendonça, R. et al. (2019). Seasonal and diel variation in greenhouse gas emissions from an urban pond and its major drivers. *Limnol. Oceanogr.* **64**:2129–2139. DOI:10.1002/lno.11173
166. Palusci, O. and Cecere, C. (2022). Urban ventilation in the compact city: A critical review and a multidisciplinary methodology for improving sustainability and resilience in urban areas. *Sustainability* **14**:3948. DOI:10.3390/su14073948
167. Xiong, J., Li, B., Short, C.A. et al. (2024). Comprehensive evaluation of natural ventilation potential of buildings in urban areas under the influence of multiple environment-related factors. *J. Build. Eng.* **89**:109218. DOI:10.1016/j.jobe.2024.109218
168. Zhang, X., Buddhika, J.W.G., Wang, J. et al. (2023). Numerical investigation of effects of trees on cross-ventilation of an isolated building. *J. Build. Eng.* **73**:106808. DOI:10.1016/j.jobe.2023.106808
169. Tang, Y., Gao, F., Wang, C. et al. (2023). Vertical Greenery System (VGS) Renovation for Sustainable Arcade-Housing: Building Energy Efficiency Analysis Based on Digital Twin. *Sustainability* **15**:2310. DOI:10.3390/su15032310
170. Irga, P., Bailes, S., Matheson, S. et al. (2023). Incorporation of Green Infrastructure on Road Tunnel Ventilation Stacks: Potential Ambient Air Quality Improvement. *J. Living Archit.* **10**:1–15. DOI:10.46534/jliv.2023.10.02.01
171. Badach, J., Szczepański, J., Bonenberg, W. et al. (2022). Developing the urban blue-green infrastructure as a tool for urban air quality management. *Sustainability* **14**:9688. DOI:10.3390/su14159688
172. Zhang, N., Wang, H., Gallagher, J. et al. (2020). A dynamic analysis of the global warming potential associated with air conditioning at a city scale: an empirical study in Shenzhen, China. *Environ. Impact Assess. Rev.* **81**:106354. DOI:10.1016/j.eiar.2019.106354
173. Morgan, D.T., Daly, T., Gallagher, J. et al. (2017). Reducing energy consumption and increasing filter life in HVAC systems using an aspiration efficiency reducer: Long-term performance assessment at full-scale. *J. Build. Eng.* **12**:267–274. DOI:10.1016/j.jobe.2017.06.014
174. Wahba, S.M., Kamel, B.A., Nassar, K.M. et al. (2018). Effectiveness of Green Roofs and Green Walls on Energy Consumption and Indoor Comfort in Arid Climates. *Civ. Eng. J.* **4**:2284–2295. DOI:10.28991/cej-03091158
175. Beier, M., Gerstendörfer, J., Mendzigall, K. et al. (2022). Climate impact and model approaches of blue-green infrastructure measures for neighborhood planning. *Sustainability* **14**:6861. DOI:10.3390/su14116861
176. Shi, Z., Yang, J., Zhang, Y. et al. (2022). Urban ventilation corridors and spatiotemporal divergence patterns of urban heat island intensity: a local climate zone perspective. *Environ. Sci. Pollut. Res. Int.* **29**:74394–74406. DOI:10.1007/s11356-022-21037-9
177. Buccolieri, R., Sandberg, M. and Di Sabatino, S. (2010). City breathability and its link to pollutant concentration distribution within urban-like geometries. *Atmos. Environ. X.* **44**:1894–1903. DOI:10.1016/j.atmosenv.2010.02.022
178. Park, B.J., Lee, D.K., Yun, S.H. et al. (2024). Assessing the impact of green infrastructure on thermal comfort in relation to humidity: A case study in Korea. *Urban For. Urban Green.* **95**:128305. DOI:10.1016/j.ufug.2024.128305
179. Asere, L. and Blumberga, A. (2020). Does energy efficiency-indoor air quality dilemma have an impact on the gross domestic product? *J. Environ. Manage.* **262**:110270. DOI:10.1016/j.jenvman.2020.110270
180. Amorim, J.H., Rodrigues, V., Tavares, R. et al. (2013). CFD modelling of the aerodynamic effect of trees on urban air pollution dispersion. *Sci. Total Environ.* **461–462**:541–551. DOI:10.1016/j.scitotenv.2013.05.031
181. Vasudevan, M., Pilla, F. and McNabola, A. (2024). Assessment of pollution removal mechanisms in steep-asymmetric city-type environments using wind deflectors. *Energy Built Environ* **25**. DOI:10.1016/j.enbenv.2024.07.007
182. Zhao, Z., Li, H. and Wang, S. (2024). Machine learning-based surrogate models for fast impact assessment of a new building on urban local microclimate at design stage. *Build. Environ.* **266**:112142. DOI:10.1016/j.buildenv.2024.112142
183. Belda, M., Resler, J., Geletič, J. et al. (2021). Sensitivity analysis of the PALM model system 6.0 in the urban environment. *Geosci. Model Dev. (GMD)* **14**:4443–4464. DOI:10.5194/gmd-14-4443-2021
184. Considine, B., Gallagher, J., Kumar, P. et al. (2023). The impact of street level particulate emissions on the energy performance of roof level building ventilation systems. *J. Wind Eng. Ind. Aerodyn.* **233**:105310. DOI:10.1016/j.jweia.2023.105310
185. Considine, B., McNabola, A., Kumar, P. et al. (2022). A numerical analysis of particulate matter control technology integrated with HVAC system inlet design and implications on energy consumption. *Build. Environ.* **211**:108726. DOI:10.1016/j.jweia.2023.105310
186. Liu, H., Kong, F., Yin, H. et al. (2021). Impacts of green roofs on water, temperature, and air quality: A bibliometric review. *Build. Environ.* **196**:107794. DOI:10.1016/j.buildenv.2021.107794
187. Ramasubramanian, P., Luhung, I., Lim, S.B.Y. et al. (2021). Impact of green and white roofs on air handler filters and indoor ventilation air. *Build. Environ.* **197**:107860. DOI:10.1016/j.buildenv.2021.107860
188. Ramasubramanian, P., Starry, O., Rosenstiel, T. et al. (2019). Pilot study on the impact of green roofs on ozone levels near building ventilation air supply. *Build. Environ.* **151**:43–53. DOI:10.1016/j.buildenv.2019.01.023
189. Kostadinović, D., Jovanović, M., Bakić, V. et al. (2023). Mitigation of urban particulate pollution using lightweight green roof system. *Energy Build.* **293**:113203. DOI:10.1016/j.enbuild.2023.113203
190. Sharifi, A. (2023). Resilience of urban social-ecological-technological systems (SETS): A review. *Sustain. Cities Soc.* **99**:104910. DOI:10.1016/j.scs.2023.104910
191. Huang, X., Yao, R., Halios, C.H. et al. (2025). Integrating green infrastructure, design scenarios, and social-ecological-technological systems for thermal resilience and adaptation: Mechanisms and approaches. *Renew. Sustain. Energy Rev.* **212**:115422. DOI:10.1016/j.rser.2025.115422
192. Brager, G.S. and De Dear, R.J. (1998). Thermal adaptation in the built environment: a literature review. *Energy Build.* **27**:83–96. DOI:10.1016/s0378-7788(97)00053-4
193. Dashti, A., Mohammadsharifi, N., Shokuhi, M. et al. (2024). A comprehensive study on wintertime outdoor thermal comfort of blue-green infrastructure in an arid climate: A case of Isfahan, Iran. *Sustain. Cities Soc.* **113**:105658. DOI:10.1016/j.scs.2024.105658
194. Huang, B., Zhao, Y., Yang, J. et al. (2024). Thermal Comfort and Restorative Benefits of Waterfront Green Spaces for College Students in Hot and Humid Regions. *Sustainability* **16**:8924. DOI:10.3390/su16208924
195. Climate-ADAPT (2022). Paris Oasis Schoolyard Programme (European Environment Agency). https://climate-adapt.eea.europa.eu/en/metadata/case-studies/paris-oasis-schoolyard-programme-france
196. Benmarhnia, T., Deguen, S., Kaufman, J.S. et al. (2015). Vulnerability to heat-related mortality: A systematic review, meta-analysis, and meta-regression analysis. *Epidemiology* **26**:781–793. DOI:10.1097/ede.0000000000000375
197. Qi, J., Wang, J., Zhai, W. et al. (2022). Are There Differences in Thermal Comfort Perception of Children in Comparison to Their Caregivers' Judgments? A Study on the Playgrounds of Parks in China's Hot Summer and Cold Winter Region. *Sustainability* **14**:10926. DOI:10.3390/su141710926
198. Fei, F., Wang, L., Wang, Y. et al. (2023). A new method for evaluating the synergistic effect of urban water body and vegetation in the summer outdoor thermal environment. *J. Clean. Prod.* **414**:137680. DOI:10.1016/j.jclepro.2023.137680
199. Fei, F., Wang, Y., Wang, L. et al. (2023). Mechanisms of urban blue-green infrastructure on winter microclimate using artificial neural network. *Energy Build.* **293**:113188. DOI:10.1016/j.enbuild.2023.113188
200. Zhu, S. and Zhang, G. (2011). Progress in near surface air temperature retrieved by remote sensing technology. *Adv. Earth Sci.* **26**:724. DOI:10.11867/j.issn.1001-8166.2011.07.0724
201. Manavvi, S. and Milosevic, D. (2025). Chasing cool: Unveiling the influence of green-blue features on outdoor thermal environment in Roorkee (India). *Build. Environ.* **267**:112238. DOI:10.1016/j.buildenv.2024.112238
202. Sheng, S. and Wang, Y. (2024). Configuration characteristics of green-blue spaces for efficient cooling in urban environments. *Sustain. Cities Soc.* **100**:105040. DOI:10.1016/j.scs.2023.105040
203. Islam, M.R., Talukdar, S., Talukdar, S. et al. (2024). Evaluating cooling effect of blue-green infrastructure on urban thermal environment in a metropolitan city: Using geospatial and

machine learning techniques. *Sustain. Cities Soc.* **113**:105666. DOI:10.1016/j.scs.2024.105666
204. Wang, W., He, J. and Wang, X. (2024). Quantitatively comparing the morphological influences on the cool island effect in urban waterfront blue-green spaces across six cities near 30° N. *Urban Clim.* **56**:102076. DOI:10.1016/j.uclim.2024.102076
205. Liu, X., Gou, Z. and Yuan, C. (2024). Application of human-centric digital twins: Predicting outdoor thermal comfort distribution in Singapore using multi-source data and machine learning. *Urban Clim.* **58**:102210. DOI:10.1016/j.uclim.2024.102210
206. Andersson, E., Langemeyer, J., Borgström, S. et al. (2019). Enabling green and blue infrastructure to improve contributions to human well-being and equity in urban systems. *Bioscience* **69**:566–574. DOI:10.1093/biosci/biz058
207. López, J., Way, D.A. and Sadok, W. (2021). Systemic effects of rising atmospheric vapor pressure deficit on plant physiology and productivity. *Glob. Change Biol.* **27**:1704–1720. DOI:10.1111/gcb.15548
208. Matsumoto, M., Kiyomizu, T., Yamagishi, S. et al. (2022). Responses of photosynthesis and long-term water use efficiency to ambient air pollution in urban roadside trees. *Urban Ecosyst.* **25**:1029–1042. DOI:10.1007/s11252-022-01212-z
209. Locosselli, G.M., Brienen, R.J.W., Leite, M.D.S. et al. (2020). Global tree-ring analysis reveals rapid decrease in tropical tree longevity with temperature. *Proc. Natl. Acad. Sci. USA* **117**:33358–33364. DOI:10.1073/pnas.2003873117
210. Brienen, R.J.W., Caldwell, L., Duchesne, L. et al. (2020). Forest carbon sink neutralized by pervasive growth-lifespan trade-offs. *Nat. Commun.* **11**:4241. DOI:10.1038/s41467-020-17966-z
211. Hammond, W.M., Williams, A.P., Abatzoglou, J.T. et al. (2022). Global field observations of tree die-off reveal hotter-drought fingerprint for Earth's forests. *Nat. Commun.* **13**:1761. DOI:10.1038/s41467-022-29289-2
212. Wiens, J.J. and Zelinka, J. (2024). How many species will Earth lose to climate change? *Glob. Change Biol.* **30**:e17125. DOI:10.1111/gcb.17125
213. Dobson, A., Rowe, Z., Berger, J. et al. (2021). Biodiversity loss due to more than climate change. *Science* **374**:699–700. DOI:10.1126/science.abm6216
214. Habibullah, M.S., Din, B.H., Tan, S.-H. et al. (2022). Impact of climate change on biodiversity loss: global evidence. *Environ. Sci. Pollut. Res. Int.* **29**:1073–1086. DOI:10.1007/s11356-021-15702-8
215. Zuidema, P.A., Groenendijk, P., Rahman, M. et al. (2025). Pantropical tree rings show small effects of drought on stem growth. *Science* **389**:532–538. DOI:10.1126/science.adq6607
216. Stewart, M., Carleton, W.C. and Groucutt, H.S. (2022). Extreme events in biological, societal, and earth sciences: A systematic review of the literature. *Front. Earth Sci.* **10**:786829. DOI:10.3389/feart.2022.786829
217. Carscadden, K.A., Emery, N.C., Arnillas, C.A. et al. (2020). Niche breadth: causes and consequences for ecology, evolution, and conservation. *Q. Rev. Biol.* **95**:179–214. DOI:10.1086/710388
218. Phillips, R.D., Peakall, R., van der Niet, T. et al. (2020). Niche perspectives on plant–pollinator interactions. *Trends Plant Sci.* **25**:779–793. DOI:10.1016/j.tplants.2020.03.009
219. Locosselli, G.M., Cintra, B.B.L., Ferreira, L.S. et al. (2024). Stress-tolerant trees for resilient cities: Tree-ring analysis reveals species suitable for a future climate. *Urban Clim.* **55**:101964. DOI:10.1016/j.uclim.2024.101964
220. Das, S., Ossola, A. and Beaumont, L.J. (2024). Records of urban occurrences expand estimates of the climate niches in tree species. *Glob. Ecol. Biogeogr.* **33**:e13809. DOI:10.1111/geb.13809
221. Hanley, P.A., Arndt, S.K., Livesley, S.J. et al. (2021). Relating the climate envelopes of urban tree species to their drought and thermal tolerance. *Sci. Total Environ.* **753**:142012. DOI:10.1016/j.scitotenv.2020.142012
222. Traubenik, S., Charon, C. and Blein, T. (2024). From environmental responses to adaptation: the roles of plant lncRNAs. *Plant Physiol.* **195**:232–244. DOI:10.1093/plphys/kiae034
223. Esperon-Rodriguez, M., Rymer, P.D., Power, S.A. et al. (2020). Functional adaptations and trait plasticity of urban trees along a climatic gradient. *Urban For. Urban Green.* **54**:126771. DOI:10.1016/j.ufug.2020.126771
224. Martínez-Villa, J.A., Paquette, A., Feeley, K.J. et al. (2024). Changes in morphological and physiological traits of urban trees in response to elevated temperatures within an Urban Heat Island. *Tree Physiol.* **44**:tpae145. DOI:10.1093/treephys/tpae145
225. Farrell, C.C., Penuel, W.R., Allen, A. et al. (2022). Learning at the boundaries of research and practice: A framework for understanding research–practice partnerships. *Educ. Res.* **51**:197–208. DOI:10.3102/0013189x211069073
226. Van Oijstaeijen, W., Van Passel, S. and Cools, J. (2020). Urban green infrastructure: A review on valuation toolkits from an urban planning perspective. *J. Environ. Manage.* **267**:110603. DOI:10.1016/j.jenvman.2020.110603
227. Des Marais, D.L., Hernandez, K.M. and Juenger, T.E. (2013). Genotype-by-environment interaction and plasticity: exploring genomic responses of plants to the abiotic environment. *Annu. Rev. Ecol. Evol. Syst.* **44**:5–29. DOI:10.1146/annurev-ecolsys-110512-135806
228. Nicotra, A.B., Atkin, O.K., Bonser, S.P. et al. (2010). Plant phenotypic plasticity in a changing climate. *Trends Plant Sci.* **15**:684–692. DOI:10.1016/j.tplants.2010.09.008
229. Géron, C., Lembrechts, J.J., Fameree, M. et al. (2024). Phenotypic plasticity as the main driver of alien plant trait variation in urban versus rural microclimate for the model species Veronica persica. *Oecologia* **205**:643–654. DOI:10.1007/s00442-024-05597-w
230. Papadopoulou, S., Stefi, A.L., Meletiou-Christou, M.-S. et al. (2023). Structural and physiological traits of compound leaves of Ceratonia siliqua trees grown in urban and suburban ambient conditions. *Plants* **12**:514. DOI:10.3390/plants12030514
231. Wei, Y., Li, Z., Zhang, J. et al. (2023). Effects of artificial light at night and drought on the photosynthesis and physiological traits of two urban plants. *Front. Plant Sci.* **14**:1263795. DOI:10.3389/fpls.2023.1263795
232. Wang, Y., Li, B., Bao, P. et al. (2023). A case study of leaf wettability variability and the relations with leaf traits and surface water storage for urban landscape plants. *Water* **15**:2152. DOI:10.3390/w15122152
233. Krashniak, A. (2021). The struggle for life and adaptation by natural selection. *Biol. Philos.* **36**:28. DOI:10.1007/s10539-021-09803-4
234. Fukano, Y., Uchida, K. and Tachiki, Y. (2023). Urban-rural gradients: how landscape changes drive adaptive evolution of plant competitive traits. *Evol. Ecol.* **37**:215–232. DOI:10.1007/s10682-022-10215-3
235. Fukano, Y., Yamori, W., Misu, H. et al. (2023). From green to red: Urban heat stress drives leaf color evolution. *Sci. Adv.* **9**:eabq3542. DOI:10.1126/sciadv.abq3542
236. Fukano, Y., Guo, W., Uchida, K. et al. (2020). Contemporary adaptive divergence of plant competitive traits in urban and rural populations and its implication for weed management. *J. Ecol.* **108**:2521–2530. DOI:10.1111/1365-2745.13472
237. de Barros Ruas, R., Costa, L.M.S. and Bered, F. (2022). Urbanization driving changes in plant species and communities–A global view. *Glob. Ecol. Conserv.* **38**:e02243. DOI:10.1016/j.gecco.2022.e02243
238. Woudstra, Y., Kraaiveld, R., Jorritsma, A. et al. (2024). Some like it hot: adaptation to the urban heat island in common dandelion. *Evol. Lett.* **8**:881–892. DOI:10.1093/evlett/qrae040
239. Lambert, M.R., Brans, K.I., Des Roches, S. et al. (2021). Adaptive evolution in cities: progress and misconceptions. *Trends Ecol. Evol.* **36**:239–257. DOI:10.1016/j.tree.2020.11.002
240. Bertrand, R., Lenoir, J., Piedallu, C. et al. (2011). Changes in plant community composition lag behind climate warming in lowland forests. *Nature* **479**:517–520. DOI:10.1038/nature10548
241. Haldane, J.B.S. (1957). The cost of natural selection. *J. Genet.* **55**:511–524. http://www.jstor.org/stable/24094587
242. Akio Lucchezi Miyahara, A., Wild, T., Afonso Sandre, A. et al. (2022). Developing and classifying urban biomes as a basis for nature-based solutions. *Urban Clim.* **45**:101251. DOI:10.1016/j.uclim.2022.101251
243. Sotillo, A., Hardion, L., Chanez, E. et al. (2024). Plant responses to urban gradients: Extinction, plasticity, adaptation. *J. Ecol.* **112**:2861–2875. DOI:10.1111/1365-2745.14427
244. Silva Luz, C.L.d., Reale, R., Candido, L.F. et al. (2024). Using Morphological Characters to Support Decision-Making in Nature-Based Solutions: A Shortcut to Promote Urban Plant Biodiversity. *Urban Sci.* **8**:233. DOI:10.3390/urbansci8040233
245. Hahs, A.K., McDonnell, M.J., McCarthy, M.A. et al. (2009). A global synthesis of plant extinction rates in urban areas. *Ecol. Lett.* **12**:1165–1173. DOI:10.1111/j.1461-0248.2009.01372.x
246. Mell, I. and Clement, S. (2020). Progressing green infrastructure planning: understanding its scalar, temporal, geo-spatial and disciplinary evolution. *IAPA* **38**:449–463. DOI:10.1080/14615517.2019.1617517
247. Puppim de Oliveira, J.A., Bellezoni, R.A., Shih, W.-Y. et al. (2022). Innovations in Urban Green and Blue Infrastructure: Tackling local and global challenges in cities. *J. Clean. Prod.* **362**:132355. DOI:10.1016/j.jclepro.2022.132355
248. Hutchins, M.G., Fletcher, D., Hagen-Zanker, A. et al. (2021). Why scale is vital to plan optimal Nature-Based Solutions for resilient cities. *Environ. Res. Lett.* **16**:044008. DOI:10.1088/1748-9326/abd9f4
249. Paredes Méndez, D., Pérez-Sánchez, M., Sánchez-Romero, F.J. et al. (2025). Assessment of the Effectiveness of Green Infrastructure Interventions to Enhance the Ecosystem Services in Developing Countries. *Urban Sci.* **9**:85. DOI:10.3390/urbansci9030085
250. Alves, R.A., Santos, M.M.D., Rudke, A.P. et al. (2024). Site selection for nature-based solutions for stormwater management in urban areas: An approach combining GIS and multi-criteria analysis. *J. Environ. Manage.* **359**:120999. DOI:10.1016/j.jenvman.2024.120999
251. King, P. and Bark, R.H. (2024). From local solutions to catchment-wide management: an investigation of upstream-downstream trade-offs when scaling out nature-based flood risk management. *Ecosyst. People* **20**:2426716. DOI:10.1080/26395916.2024.2426716
252. Obiang Ndong, G., Therond, O. and Cousin, I. (2020). Analysis of relationships between ecosystem services: A generic classification and review of the literature. *Ecosyst. Serv.* **43**:101120. DOI:10.1016/j.ecoser.2020.101120
253. Alves, R.A., Rudke, A.P., Martins, L.D. et al. (2024). Urban stormwater management from the perspective of nature-based solutions: a bibliometric review. *TJoE* **9**:218–238. DOI:10.1080/24705357.2023.2284392
254. Zhang, B. and MacKenzie, A. (2024). Trade-offs and synergies in urban green infrastructure: A systematic review. *Urban For. Urban Green.* **94**:128262. DOI:10.1016/j.ufug.2024.128262
255. Hansen, R., Olafsson, A.S., van der Jagt, A.P.N. et al. (2019). Planning multifunctional green infrastructure for compact cities: What is the state of practice? *Ecol. Indic.* **96**:99–110. DOI:10.1016/j.ecolind.2017.09.042
256. Meerow, S., Pajouhesh, P. and Miller, T.R. (2019). Social equity in urban resilience planning. *Local Environ.* **24**:793–808. DOI:10.1080/13549839.2019.1645103
257. Alves, A., van Opstal, C., Keijzer, N. et al. (2024). Planning the multifunctionality of nature-based solutions in urban spaces. *Cities* **146**:104751. DOI:10.1016/j.cities.2023.104751
258. Chen, H., Wang, N., Liu, Y. et al. (2022). A green infrastructure planning framework–guidance for priority, hubs and types. *Urban For. Urban Green.* **70**:127545. DOI:10.1016/j.ufug.2022.127545

259. Lourdes, K.T., Hamel, P., Gibbins, C.N. et al. (2022). Planning for green infrastructure using multiple urban ecosystem service models and multicriteria analysis. *Landsc. Urban Plann.* **226**:104500. DOI:10.1016/j.landurbplan.2022.104500
260. Yuan, Q., Meng, F., Li, W. et al. (2025). Tradeoff optimization of urban roof systems oriented to food-water-energy nexus. *Appl. Energy* **380**:124987. DOI:10.1016/j.apenergy.2024.124987
261. Ronchi, S., Arcidiacono, A. and Pogliani, L. (2020). Integrating green infrastructure into spatial planning regulations to improve the performance of urban ecosystems. Insights from an Italian case study. *Sustain. Cities Soc.* **53**:101907. DOI:10.1016/j.scs.2019.101907
262. Li, L. and Carter, J. (2025). Exploring the relationship between urban green infrastructure connectivity, size and multifunctionality: a systematic review. *Landsc. Ecol.* **40**:61. DOI:10.1007/s10980-025-02069-1
263. Cook, L.M., Good, K.D., Moretti, M. et al. (2024). Towards the intentional multifunctionality of urban green infrastructure: a paradox of choice? *npj Urban Sustain.* **4**:12. DOI:10.1038/s42949-024-00145-0
264. Brom, P., Engemann, K., Breed, C. et al. (2023). A decision support tool for green infrastructure planning in the face of rapid urbanization. *Land* **12**:415. DOI:10.3390/land12020415
265. Anderson, V., Zgela, M. and Gough, W.A. (2023). Building urban resilience with Nature-Based Solutions: A multi-scale case study of the atmospheric cleansing potential of Green Infrastructure in Southern Ontario, Canada. *Sustainability* **15**:14146. DOI:10.3390/su151914146
266. Chen, X., Wang, H. and Yang, J. (2024). Effect of green blue spaces on the urban thermal environment: A field study in Hong Kong. *Urban Clim.* **55**:101912. DOI:10.1016/j.uclim.2024.101912
267. Tseng, K.H., Chung, M.Y., Chen, L.H. et al. (2022). Applying an Integrated System of Cloud Management and Wireless Sensing Network to Green Smart Environments-Green Energy Monitoring on Campus. *Sensors-Basel* **22**:6521
268. Calatayud-Vernich, P., Calatayud, F., Simó, E. et al. (2018). Pesticide residues in honey bees, pollen and beeswax: Assessing beehive exposure. *Environ. Pollut.* **241**:106–114. DOI:10.1016/j.envpol.2018.05.062
269. Lamano Ferreira, M., Portela Ribeiro, A., Rakauskas, F. et al. (2024). Spatiotemporal monitoring of subtropical urban forests in mitigating air pollution: Policy implications for nature-based solutions. *Ecol. Indic.* **158**:111386. DOI:10.1016/j.ecolind.2023.111386
270. Theophilo, C.Y.S., Ribeiro, A.P., Moreira, E.G. et al. (2021). Biomonitoring as a nature-based solution to assess atmospheric pollution and impacts on public health. *Bull. Environ. Contam. Toxicol.* **107**:29–36. DOI:10.1007/s00128-021-03205-8
271. Rachid, L., Elmostafa, A., Mehdi, M. et al. (2024). Assessing carbon storage and sequestration benefits of urban greening in Nador City, Morocco, utilizing GIS and the InVEST model. *Sustain. Futur.* **7**:100171. DOI:10.1016/j.sftr.2024.100171
272. Hamel, P., Guerry, A.D., Polasky, S. et al. (2021). Mapping the benefits of nature in cities with the InVEST software. *npj Urban Sustain.* **1**:25. DOI:10.1038/s42949-021-00027-9
273. Fletcher, D.H., Likongwe, P.J., Chiotha, S.S. et al. (2021). Using demand mapping to assess the benefits of urban green and blue space in cities from four continents. *Sci. Total Environ.* **785**:147238. DOI:10.1016/j.scitotenv.2021.147238
274. Vesuviano, G., Fitch, A., Owen, D. et al. (2025). How well does the 3–30–300 rule mitigate urban flooding? *Urban For. Urban Green.* **104**:128661. DOI:10.1016/j.ufug.2024.128661
275. Dong, X., Ye, Y., Su, D. et al. (2025). Adaptive ranking of specific tree species for targeted green infrastructure intervention in response to urban hazards. *Urban For. Urban Green.* **107**:128776. DOI:10.1016/j.ufug.2025.128776
276. Oukawa, G.Y., Krecl, P., Targino, A.C. et al. (2025). Mitigating urban heat stress through green infrastructure: A climate service approach. *Urban Clim.* **61**:102384. DOI:10.1016/j.uclim.2025.102384
277. Mulligan, J., Bukachi, V., Clause, J.C. et al. (2020). Hybrid infrastructures, hybrid governance: New evidence from Nairobi (Kenya) on green-blue-grey infrastructure in informal settlements. *Anthropocene* **29**:100227. DOI:10.1016/j.ancene.2019.100227
278. Iojă, C.I., Badiu, D.L., Haase, D. et al. (2021). How about water? Urban blue infrastructure management in Romania. *Cities* **110**:103084. DOI:10.1016/j.cities.2020.103084
279. Ampatzidis, P. and Kershaw, T. (2020). A review of the impact of blue space on the urban microclimate. *Sci. Total Environ.* **730**:139068. DOI:10.1016/j.scitotenv.2020.139068
280. Cheng, Y., Bartesaghi-Koc, C., Tian, Y. et al. (2023). Where and how to cool through blue infrastructure? Large lake groups to ameliorate urban overheating in a typical inland multi-lake megacity. *Sustain. Cities Soc.* **98**:104869. DOI:10.1016/j.scs.2023.104869
281. Abd-Elmabod, S.K., Gui, D., Liu, Q. et al. (2024). Seasonal environmental cooling benefits of urban green and blue spaces in arid regions. *Sustain. Cities Soc.* **115**:105805. DOI:10.1016/j.scs.2024.105805
282. Castellar, J.A.C., Torrens, A., Buttiglieri, G. et al. (2022). Nature-based solutions coupled with advanced technologies: An opportunity for decentralized water reuse in cities. *J. Clean. Prod.* **340**:130660. DOI:10.1016/j.jclepro.2022.130660
283. Hermanski, A., McClelland, J., Pearce-Walker, J. et al. (2022). The effects of blue spaces on mental health and associated biomarkers. *Int. J. Ment. Health* **51**:203–217. DOI:10.1080/00207411.2021.1910173
284. Cea, L. and Costabile, P. (2022). Flood risk in urban areas: Modelling, management and adaptation to climate change. A review. *Hydrology* **9**:50. DOI:10.3390/hydrology9030050
285. Giordano, M.A. and Wolf, A.T. (2003). Sharing waters: Post-Rio international water management. *Nat. Resour. Forum* **27**:163–171. DOI:10.1111/1477-8947.00051
286. McEwen, L., Gorell Barnes, L., Phillips, K. et al. (2020). Reweaving urban water-community relations: Creative, participatory river "daylighting" and local hydrocitizenship. *Trans. Inst. British Geog.* **45**:779–801. DOI:10.1111/tran.12375
287. Moosavi, S. (2017). Ecological coastal protection: pathways to living shorelines. *Procedia Eng.* **196**:930–938. DOI:10.1016/j.proeng.2017.08.027
288. Seddon, N., Chausson, A., Berry, P. et al. (2020). Understanding the value and limits of nature-based solutions to climate change and other global challenges. *Phil. Trans. R. Soc. B* **375**:20190120. DOI:10.1098/rstb.2019.0120
289. Aziz, F., Wang, X., Mahmood, M.Q. et al. (2024). Coastal urban flood risk management: Challenges and opportunities— A systematic review. *J. Hydrol. X.* **645**:132271. DOI:10.1016/j.jhydrol.2024.132271
290. Davis, M. and Naumann, S. (2017). Making the case for sustainable urban drainage systems as a nature-based solution to urban flooding. Nature-Based Solutions to Climate Change Adaptation in Urban Areas: Linkages between Science. *Policy and Practice*:pp123–pp137. DOI:10.1007/978-3-319-56091-5_8
291. Ikemoto, F., Sakura, K. and Torres Astaburuaga, A. (2021). The influence of historical irrigation canals on urban morphology in Valencia, Spain. *Land* **10**:738. DOI:10.3390/land10070738
292. Guimarães, L.F., Teixeira, F.C., Pereira, J.N. et al. (2021). The challenges of urban river restoration and the proposition of a framework towards river restoration goals. *J. Clean. Prod.* **316**:128330. DOI:10.1016/j.jclepro.2021.128330
293. Wantzen, K.M., Piednoir, T., Cao, Y. et al. (2022). Back to the surface—Daylighting urban streams in a Global North–South comparison. *Front. Ecol. Evol.* **10**:838794. DOI:10.3389/fevo.2022.838794
294. Alves, A., Vojinovic, Z., Kapelan, Z. et al. (2020). Exploring trade-offs among the multiple benefits of green-blue-grey infrastructure for urban flood mitigation. *Sci. Total Environ.* **703**:134980. DOI:10.1016/j.scitotenv.2019.134980
295. Kronenberg, J., Andersson, E., Barton, D.N. et al. (2021). The thorny path toward greening: Unintended consequences, trade-offs, and constraints in green and blue infrastructure planning, implementation, and management. *Ecol. Soc.* **26**:36. DOI:10.5751/ES-12445-260236
296. Louarn, A., Meur-Ferec, C. and Hervé-Fournereau, N. (2025). The concept of'nature-based solutions' applied to urban coastal risks: A bibliometric and content analysis review. *Ocean Coast Manag.* **261**:107530. DOI:10.1016/j.ocecoaman.2024.107530
297. Bozkurt, M. and Woolley, H. (2020). Let's splash: Children's active and passive water play in constructed and natural water features in urban green spaces in Sheffield. *Urban For. Urban Green.* **52**:126696. DOI:10.1016/j.ufug.2020.126696
298. van den, B.N., Elliott, L.R., White, M.P. et al. (2021). Urban blue space renovation and local resident and visitor well-being: A case study from Plymouth, UK. *Landsc. Urban Plann.* **215**:104232. DOI:10.1016/j.landurbplan.2021.104232
299. Penz, V., Mumladze-Detering, A. and Sperger, A. (2024). Swimming Is Winning- - The Case of an Urban Bathing Revival in Vienna's Danube Canal. *UXUC* **6**:54–71. DOI:10.48619/UXUC.V6I1.907
300. Riley, C.B. and Gardiner, M.M. (2020). Examining the distributional equity of urban tree canopy cover and ecosystem services across United States cities. *PLoS One* **15**:e0228499. DOI:10.1371/journal.pone.0228499
301. Volin, E., Ellis, A., Hirabayashi, S. et al. (2020). Assessing macro-scale patterns in urban tree canopy and inequality. *Urban For. Urban Green.* **55**:126818. DOI:10.1016/j.ufug.2020.126818
302. Schwarz, K., Fragkias, M., Boone, C.G. et al. (2015). Trees grow on money: urban tree canopy cover and environmental justice. *PLoS One* **10**:e0122051. DOI:10.1371/journal.pone.0122051
303. Roman, L., Catton, I., Greenfield, E. et al. (2021). Linking urban tree cover change and local history in a post-industrial city. *Land* **10**:403. DOI:10.3390/land10040403
304. Landry, F., Dupras, J. and Messier, C. (2020). Convergence of urban forest and socio-economic indicators of resilience: A study of environmental inequality in four major cities in eastern Canada. *Landsc. Urban Plann.* **202**:103856. DOI:10.1016/j.landurbplan.2020.103856
305. Gerrish, E. and Watkins, S.L. (2018). The relationship between urban forests and income: A meta-analysis. *Landsc. Urban Plann.* **170**:293–308. DOI:10.1016/j.landurbplan.2017.09.005
306. Saverino, K.C., Routman, E., Lookingbill, T.R. et al. (2021). Thermal inequity in Richmond, VA: the effect of an unjust evolution of the urban landscape on urban heat islands. *Sustainability* **13**:1511. DOI:10.3390/su13031511
307. Debbage, N. (2019). Multiscalar spatial analysis of urban flood risk and environmental justice in the Charlanta megaregion, USA. *Anthropocene* **28**:100226. DOI:10.1016/j.ancene.2019.100226
308. Venter, Z.S., Shackleton, C.M., Van Staden, F. et al. (2020). Green Apartheid: Urban green infrastructure remains unequally distributed across income and race geographies in South Africa. *Landsc. Urban Plann.* **203**:103889. DOI:10.1016/j.landurbplan.2020.103889
309. Heynen, N., Perkins, H.A. and Roy, P. (2006). The political ecology of uneven urban green space: The impact of political economy on race and ethnicity in producing environmental inequality in Milwaukee. *Urban Aff. Rev.* **42**:3–25. DOI:10.1177/1078087406290729
310. Anguelovski, I. and Connolly, J.J. (2024). Segregating by greening: What do we mean by green gentrification? *J. Plan. Lit.* **39**:386–394. DOI:10.1177/08854122241227804
311. Anguelovski, I., Connolly, J.J.T., Cole, H. et al. (2022). Green gentrification in European and North American cities. *Nat. Commun.* **13**:3816. DOI:10.1038/s41467-022-31572-1

312. Anguelovski, I., Connolly, J.J.T., Masip, L. et al. (2018). Assessing green gentrification in historically disenfranchised neighborhoods: a longitudinal and spatial analysis of Barcelona. *Urban Geogr.* **39**:458–491. DOI:10.1080/02723638.2017.1349987
313. Haase, D., Kabisch, S., Haase, A. et al. (2017). Greening cities–To be socially inclusive? About the alleged paradox of society and ecology in cities. *Habitat Int.* **64**:41–48. DOI:10.1016/j.habitatint.2017.04.005
314. Jennings, V., Johnson Gaither, C. and Gragg, R.S. (2012). Promoting environmental justice through urban green space access: A synopsis. *Environ. Justice* **5**:1–7. DOI:10.1089/env.2011.0007
315. Zuniga-Teran, A.A., Gerlak, A.K., Mayer, B. et al. (2020). Urban resilience and green infrastructure systems: Towards a multidimensional evaluation. *Curr. Opin. Environ. Sustain.* **44**:42–47. DOI:10.1016/j.cosust.2020.05.001
316. Gibbons, J. (2023). Examining the long-term influence of New Deal era redlining on contemporary gentrification. *Urban Stud.* **60**:2816–2834. DOI:10.1177/00420980231160469
317. Nyelele, C. and Kroll, C.N. (2020). The equity of urban forest ecosystem services and benefits in the Bronx, NY. *Urban For. Urban Green.* **53**:126723. DOI:10.1016/j.ufug.2020.126723
318. Locke, D.H., Hall, B., Grove, J.M. et al. (2021). Residential housing segregation and urban tree canopy in 37 US Cities. *npj Urban Sustain.* **1**:15. DOI:10.1038/s42949-021-00022-0
319. Zhang, Z., Martin, K.L., Stevenson, K.T. et al. (2022). Equally green? Understanding the distribution of urban green infrastructure across student demographics in four public school districts in North Carolina, USA. *Urban For. Urban Green.* **67**:127434. DOI:10.1016/j.ufug.2021.127434
320. Benz, S.A. and Burney, J.A. (2021). Widespread Race and Class Disparities in Surface Urban Heat Extremes Across the United States. *Earths Future* **9**:e2021EF002016. DOI:10.1029/2021ef002016
321. Ferguson, L., Taylor, J., Zhou, K. et al. (2021). Systemic inequalities in indoor air pollution exposure in London, UK. *Build. Cities* **2**:425–448. DOI:10.5334/bc.100
322. Leong, M., Bertone, M.A., Bayless, K.M. et al. (2016). Exoskeletons and economics: indoor arthropod diversity increases in affluent neighbourhoods. *Biol. Lett.* **12**:20160322. DOI:10.1098/rsbl.2016.0322
323. Avolio, M.L., Pataki, D.E., Gillespie, T.W. et al. (2015). Tree diversity in southern California's urban forest: the interacting roles of social and environmental variables. *Front. Ecol. Evol.* **3**:73. DOI:10.3389/fevo.2015.00073
324. Clarke, L.W., Jenerette, G.D. and Davila, A. (2013). The luxury of vegetation and the legacy of tree biodiversity in Los Angeles, CA. *Landsc. Urban Plann.* **116**:48–59. DOI:10.1016/j.landurbplan.2013.04.006
325. Abi, D.T., English, S., Hickel, J. et al. (2023). Envisioning environmental equity: climate change, health, and racial justice. *Lancet* **402**:64–78. DOI:10.1016/s0140-6736(23)00919-4
326. Azadgar, A., Luciani, G. and Nyka, L. (2025). Spatial allocation of nature-based solutions in the form of public green infrastructure in relation to the socio-economic district profile–a GIS-based comparative study of Gdańsk and Rome. *Land Use Policy* **150**:107454. DOI:10.1016/j.landusepol.2024.107454
327. Grabowski, Z.J., McPhearson, T. and Pickett, S.T.A. (2023). Transforming US urban green infrastructure planning to address equity. *Landsc. Urban Plann.* **229**:104591. DOI:10.1016/j.landurbplan.2022.104591
328. Lopes, H.S., Vidal, D.G., Cherif, N. et al. (2025). Green infrastructure and its influence on urban heat island, heat risk, and air pollution: A case study of Porto (Portugal). *J. Environ. Manage.* **376**:124446. DOI:10.1016/j.jenvman.2025.124446
329. Kuras, E.R., Warren, P.S., Zinda, J.A. et al. (2020). Urban socioeconomic inequality and biodiversity often converge, but not always: A global meta-analysis. *Landsc. Urban Plann.* **198**:103799. DOI:10.1016/j.landurbplan.2020.103799
330. Chamberlain, D.E., Henry, D.A.W., Reynolds, C. et al. (2019). The relationship between wealth and biodiversity: A test of the Luxury Effect on bird species richness in the developing world. *Glob. Change Biol.* **25**:3045–3055. DOI:10.1111/gcb.14682
331. Zivanovic, A.J. and Luck, G.W. (2016). Social and environmental factors drive variation in plant and bird communities across urban greenspace in Sydney, Australia. *J. Environ. Manage.* **169**:210–222. DOI:10.1016/j.jenvman.2015.11.052
332. Luck, G.W., Smallbone, L.T. and Sheffield, K.J. (2012). Environmental and socio-economic factors related to urban bird communities. *Austral Ecol.* **38**:111–120. DOI:10.1111/j.1442-9993.2012.02383.x
333. Chamberlain, D., Reynolds, C., Amar, A. et al. (2020). Wealth, water and wildlife: Landscape aridity intensifies the urban luxury effect. *Glob. Ecol. Biogeogr.* **29**:1595–1605. DOI:10.1111/geb.13122
334. Graça, M.S., Gonçalves, J.F., Alves, P.J.M. et al. (2017). Assessing mismatches in ecosystem services proficiency across the urban fabric of Porto (Portugal): The influence of structural and socioeconomic variables. *Ecosyst. Serv.* **23**:82–93. DOI:10.1016/j.ecoser.2016.11.015
335. Holston, J. (2009). Insurgent citizenship in an era of global urban peripheries. *City Soc.* **21**:245–267. DOI:10.1111/j.1548-744x.2009.01024.x
336. United Nations Human Settlements Programme (2022). World Cities Report 2022: Envisaging the Future of Cities (UN iLibrary). DOI:10.18356/9789210028592
337. Arcidiacono, A., Causone, F., Grosso, M. et al. (2017). Environmental Performance and social inclusion: A project for the Rocinha Favela in Rio de Janeiro. *Energy Proc.* **134**:356–365. DOI:10.1016/j.egypro.2017.09.546
338. Pearsall, H. and Anguelovski, I. (2016). Contesting and resisting environmental gentrification: Responses to new paradoxes and challenges for urban environmental justice. *Sociol. Res. Online* **21**:121–127. DOI:10.5153/sro.3979
339. Poulton Kamakura, R., Bai, J., Sheel, V. et al. (2024). Biodiversity is not a luxury: Unpacking wealth and power to accommodate the complexity of urban biodiversity. *Ecosphere* **15**:e70049. DOI:10.1002/ecs2.70049
340. Breyer, B. and Mohr, H. (2023). Right tree, right place for whom? Environmental justice and practices of urban forest assessment. *Local Environ.* **28**:1082–1096. DOI:10.1080/13549839.2023.2184784
341. Palmer, A.K., Riley, M., Clement, S. et al. (2025). In and out of place: Diverse experiences and perceived exclusion in UK greenspace settings. *Environ. Plan. E* **8**:742–769. DOI:10.1177/25148486251316124
342. Trisos, C.H., Auerbach, J. and Katti, M. (2021). Decoloniality and anti-oppressive practices for a more ethical ecology. *Nat. Ecol. Evol.* **5**:1205–1212. DOI:10.1038/s41559-021-01460-w
343. Schell, C.J., Dyson, K., Fuentes, T.L. et al. (2020). The ecological and evolutionary consequences of systemic racism in urban environments. *Science* **369**:eaay4497. DOI:10.1126/science.aay4497
344. Elmqvist, T., Setälä, H., Handel, S. et al. (2015). Benefits of restoring ecosystem services in urban areas. *Curr. Opin. Environ. Sustain.* **14**:101–108. DOI:10.1016/j.cosust.2015.05.001
345. Palmer, A.K., Riley, M., Brockett, B.F.T. et al. (2023). Towards an understanding of quality and inclusivity in human-environment experiences. *Geogr. Compass* **17**:e12723. DOI:10.1111/gec3.12723
346. Drosou, N., Soetanto, R., Hermawan, F. et al. (2019). Key factors influencing wider adoption of blue–green infrastructure in developing cities. *Water* **11**:1234. DOI:10.3390/w11061234
347. Kati, V. and Jari, N. (2016). Bottom-up thinking—Identifying socio-cultural values of ecosystem services in local blue–green infrastructure planning in Helsinki, Finland. *Land Use Policy* **50**:537–547. DOI:10.1016/j.landusepol.2015.09.031
348. Tomateo, C. (2021). Indigenous land systems and emerging of Green Infrastructure planning in the Peruvian coastal desert: tensions and opportunities. *J. Environ. Policy Plan.* **23**:683–700. DOI:10.1080/1523908x.2021.1960806
349. Xu, X., Luo, F., Wang, W. et al. (2018). Performance-based evaluation of courtyard design in China's cold-winter hot-summer climate regions. *Sustainability* **10**:3950. DOI:10.3390/su10113950
350. Kale, E., De Groeve, M., Pinnel, L. et al. (2023). Mapping Vertical Greening on Urban Built Heritage Exposed to Environmental Stressors–A Case Study in Antwerp, Belgium. *Sustainability* **15**:12987. DOI:10.3390/su151712987
351. Fabbri, K., Catalano, M. and Ugolini, A. (2025). Vegetation in Archaeological Areas: Risks, Opportunities, and Guidelines to Preserve or Remove: An Italian Case Study. *Sustainability* **17**:2712. DOI:10.3390/su17062712
352. Minnema, L. (2019). Cross-Cultural Comparisons between the Mughal Tomb Garden of Taj Mahal in Agra (India) and the Dry Landscape Garden of the Ryoan-Ji Zen Monastery in Kyoto (Japan): An Analysis of Cultural and Religious Layers of Meaning in Two Cases of Classical Garden Landscape Architecture. *Worldviews* **23**:197–229. DOI:10.1163/15685357-02302005
353. Kabisch, N. and Haase, D. (2014). Green justice or just green? Provision of urban green spaces in Berlin, Germany. *Landsc. Urban Plann.* **122**:129–139. DOI:10.1016/j.landurbplan.2013.11.016
354. Xiong, Y., Zhang, J., Xu, X. et al. (2020). Strategies for improving the microclimate and thermal comfort of a classical Chinese garden in the hot-summer and cold-winter zone. *Energy Build.* **215**:109914. DOI:10.1016/j.enbuild.2020.109914
355. Alauddin, K., Nusa, F.N.M., Baharuddin, M.N. et al. (2022). The application of green adaptive reuse of historical buildings in UNESCO cities. *Plan. Malays.* **20**:1137. DOI:10.21837/pm.v20i22.1137
356. McNabb, T., Charters, F.J., Challies, E. et al. (2024). Unlocking urban blue-green infrastructure: an interdisciplinary literature review analysing co-benefits and synergies between bio-physical and socio-cultural outcomes. *Blue-Green Syst.* **6**:217–231. DOI:10.2166/bgs.2024.007
357. Dushkova, D., Taherkhani, M., Konstantinova, A. et al. (2025). Understanding Factors Affecting the Use of Urban Parks Through the Lens of Ecosystem Services and Blue–Green Infrastructure: The Case of Gorky Park, Moscow, Russia. *Land* **14**:237. DOI:10.3390/land14020237
358. Asad, R., Vaughan, J. and Ahmed, I. (2023). Integrated traditional water knowledge in urban design and planning practices for sustainable development: challenges and opportunities. *Sustainability* **15**:12434. DOI:10.3390/su151612434
359. Taylor, K. (2025). Urban Open Space Systems and Green Cities: History, Heritage, and All That. *Land* **14**:582. DOI:10.3390/land14030582
360. Adib, M., Wu, H. and Flohr, T. (2023). Professional perceptions of participatory practices in green stormwater infrastructure development. *PLoS Water* **2**:e0000084. DOI:10.1371/journal.pwat.0000084
361. Frantzeskaki, N., McPhearson, T., Collier, M.J. et al. (2019). Nature-based solutions for urban climate change adaptation: linking science, policy, and practice communities for evidence-based decision-making. *Bioscience* **69**:455–466. DOI:10.1093/biosci/biz042
362. Davies, C. and Lafortezza, R. (2019). Transitional path to the adoption of nature-based solutions. *Land Use Policy* **80**:406–409. DOI:10.1016/j.landusepol.2018.09.020
363. Koohestani, S., Mukheibir, S., Wakefield-Rann, P. et al. (2025). Adopting a socio-technical perspective on the challenges and barriers in transitioning to Blue-Green Infrastructure (BGI). *Blue-Green Syst.* **7**:79–94. DOI:10.2166/bgs.2025.011
364. Andersson, E., Elmqvist, T., Haase, D. et al. (2022). Based on nature, enabled by social-ecological-technological context: deriving benefit from urban green and blue infrastructure. *Ecol. Soc.* **27**:18. DOI:10.5751/es-13580-270418

365. Peters, M., Strauss, H., Farquhar, J. et al. (2010). Sulfur cycling at the Mid-Atlantic Ridge: A multiple sulfur isotope approach. *Chem. Geol.* **269**:180–196. DOI:10.1016/j.chemgeo.2009.09.016
366. Wan, C., Shen, G.Q. and Choi, S. (2021). Underlying relationships between public urban green spaces and social cohesion: A systematic literature review. *City Cult. Soc.* **24**:100383. DOI:10.1016/j.ccs.2021.100383
367. Zhu, Y., Ding, J., Zhu, Q. et al. (2017). The impact of green open space on community attachment—A case study of three communities in Beijing. *Sustainability* **9**:560. DOI:10.3390/su9040560
368. Buijs, A.E., Mattijssen, T.J., Van der Jagt, A.P. et al. (2016). Active citizenship for urban green infrastructure: fostering the diversity and dynamics of citizen contributions through mosaic governance. *Curr. Opin. Environ. Sustain.* **22**:1–6. DOI:10.1016/j.cosust.2017.01.002
369. Anguelovski, I., Connolly, J.J.T., Pearsall, H. et al. (2019). Why green "climate gentrification" threatens poor and vulnerable populations. *Proc. Natl. Acad. Sci. USA* **116**:26139–26143. DOI:10.1073/pnas.1920490117
370. Shokry, G., Connolly, J.J. and Anguelovski, I. (2020). Understanding climate gentrification and shifting landscapes of protection and vulnerability in green resilient Philadelphia. *Urban Clim.* **31**:100539. DOI:10.1016/j.uclim.2019.100539
371. Kotsila, P., Anguelovski, I., Baró, F. et al. (2020). Nature-based solutions as discursive tools and contested practices in urban nature's neoliberalisation processes. *Environ. Plan* **E**:252–274. DOI:10.1177/2514848620901437
372. Wihlborg, M., Sörensen, J. and Alkan Olsson, J. (2019). Assessment of barriers and drivers for implementation of blue-green solutions in Swedish municipalities. *J. Environ. Manage.* **233**:706–718. DOI:10.1016/j.jenvman.2018.12.018
373. Blair, J. and Osmond, P. (2020). Employing Green Roofs to Support Endangered Plant Species: The Eastern Suburbs Banksia Scrub in Australia. *Open J. Ecol.* **10**:111–140. DOI:10.4236/oje.2020.103009
374. Ambrey, C.L. (2016). An investigation into the synergistic wellbeing benefits of greenspace and physical activity: Moving beyond the mean. *Urban For. Urban Green.* **19**:7–12. DOI:10.1016/j.ufug.2016.06.020
375. Calderón-Argelich, A., Anguelovski, I., Connolly, J.J.T. et al. (2023). Greening plans as (re)presentation of the city: Toward an inclusive and gender-sensitive approach to urban greenspaces. *Urban For. Urban Green.* **86**:127984. DOI:10.1016/j.ufug.2023.127984
376. Jones, J. and Russo, A. (2024). Exploring the role of public participation in delivering inclusive, quality, and resilient green infrastructure for climate adaptation in the UK. *Cities* **148**:104879. DOI:10.1016/j.cities.2024.104879
377. Barrie, H., Soebarto, V., Lange, J. et al. (2019). Using citizen science to explore neighbourhood influences on ageing well: Pilot project. *Healthcare* **7**:126. DOI:10.3390/healthcare7040126
378. Barrie, D.H., Soebarto, P.V., McCorry, D.F. et al. (2019). Green spaces, public spaces and ageing well: A citizen science pilot study. *Healthcare* **7**:126. DOI:10.3390/healthcare7040126
379. Ferguson, M., Roberts, H.E., McEachan, R.R.C. et al. (2018). Contrasting distributions of urban green infrastructure across social and ethno-racial groups. *Landsc. Urban Plann.* **175**:136–148. DOI:10.1016/j.landurbplan.2018.03.020
380. Herath, P. and Bai, X. (2024). Benefits and co-benefits of urban green infrastructure for sustainable cities: six current and emerging themes. *Sustain. Sci.* **19**:1039–1063. DOI:10.1007/s11625-024-01475-9
381. Cleary, A., Roiko, A., Burton, N.W. et al. (2019). Changes in perceptions of urban green space are related to changes in psychological well-being: Cross-sectional and longitudinal study of mid-aged urban residents. *Health Place* **59**:102201. DOI:10.1016/j.healthplace.2019.102201
382. Benoliel, M.A., Manso, M., Ferreira, P.D. et al. (2021). "Greening" and comfort conditions in transport infrastructure systems: Understanding users' preferences. *Build. Environ.* **195**:107759. DOI:10.1016/j.buildenv.2021.107759
383. Dorst, H., van der Jagt, A., Toxopeus, H. et al. (2022). What's behind the barriers? Uncovering structural conditions working against urban nature-based solutions. *Landsc. Urban Plann.* **220**:104335. DOI:10.1016/j.landurbplan.2021.104335
384. Pandey, G., Lyden, S., Franklin, E. et al. (2025). A systematic review of agrivoltaics on productivity, profitability, and environmental co-benefits. *Sustain. Prod. Consum.* **56**:13–36. DOI:10.1016/j.spc.2025.03.006
385. Coaffee, J. and Bosher, L. (2008). Integrating counter-terrorist resilience into sustainability. *Proc. Inst. Civ. Eng. Urban Des. Plan.* **161**:75–83. DOI:10.1680/udap.2008.161.2.75
386. He, Q., Wu, L., Lee, C.S. et al. (2025). Greener the safer? Effects of urban green space on community safety and perception of safety using satellite and street view imagery data. *J. Crim. Justice* **97**:102372. DOI:10.1016/j.jcrimjus.2025.102372
387. Fisher, B.S. and Nasar, J.L. (1992). Fear of crime in relation to three exterior site features: Prospect, refuge, and escape. *Environ. Behav.* **24**:35–65. DOI:10.1177/0013916592241002
388. Kabisch, N., Qureshi, S. and Haase, D. (2015). Human–environment interactions in urban green spaces—A systematic review of contemporary issues and prospects for future research. *Environ. Impact Assess. Rev.* **50**:25–34. DOI:10.1016/j.eiar.2014.08.007
389. UN-Habitat (2020). World Cities Report 2020: The Value of Sustainable Urbanization (United Nations Human Settlements Programme (UN-Habitat)). DOI:10.18356/27bc31a5-en
390. Rahm, J., Sternudd, C. and Johansson, M. (2021). "In the evening, I don't walk in the park": The interplay between street lighting and greenery in perceived safety. *Urban Des. Int.* **26**:42–52. DOI:10.1057/s41289-020-00134-6
391. Rigolon, A. and Christensen, J. (2019). Greening without gentrification. https://old.vibrantcitieslab.com/wp-content/uploads/2020/07/Greening-without-Gentrification-report-2019.pdf
392. Hedayati Marzbali, M., Abdullah, A., Razak, N.A. et al. (2012). Validating crime prevention through environmental design construct through checklist using structural equation modelling. *Int. J. Law Crime Justice* **40**:82–99. DOI:10.1016/j.ijlcj.2011.08.005
393. Piroozfar, P., Farr, E.R.P., Aboagye-Nimo, E. et al. (2019). Crime prevention in urban spaces through environmental design: A critical UK perspective. *Cities* **95**:102411. DOI:10.1016/j.cities.2019.102411
394. Nordh, H., Hartig, T., Hagerhall, C.M. et al. (2009). Components of small urban parks that predict the possibility for restoration. *Urban For. Urban Green.* **8**:225–235. DOI:10.1016/j.ufug.2009.06.003
395. Sreetheran, M.S.M. and Bosh, C.V.D. (2015). Fear of crime in urban parks-what the residents of Kuala Lumpur have to say? *Urban For. Urban Green.* **14**:702–713. DOI:10.1016/j.ufug.2015.05.012
396. Li, X., Zhang, C. and Li, W. (2015). Does the visibility of greenery increase perceived safety in urban areas? Evidence from the place pulse 1.0 dataset. *ISPRS Int. J. GeoInf.* **4**:1166–1183. DOI:10.3390/ijgi4031166
397. Pauleit, S., Ambrose-Oji, B., Andersson, E. et al. (2019). Advancing urban green infrastructure in Europe: Outcomes and reflections from the GREEN SURGE project. *Urban For. Urban Green.* **40**:4–16. DOI:10.1016/j.ufug.2018.10.006
398. Kumar, P., Druckman, A., Gallagher, J. et al. (2019). The nexus between air pollution, green infrastructure and human health. *Environ. Int.* **133**:105181. DOI:10.1016/j.envint.2019.105181
399. Haase, D., Frantzeskaki, N. and Elmqvist, T. (2014). Ecosystem services in urban landscapes: practical applications and governance implications. *Ambio* **43**:407–412. DOI:10.1007/s13280-014-0503-1
400. Bennetts, H., Soebarto, V., Oakley, S. et al. (2017). Feeling safe and comfortable in the urban environment. *J. Urban.* **10**:401–421. DOI:10.1080/17549175.2017.1310746
401. Bartesaghi-Koc, C., Osmond, P. and Peters, A. (2019). Mapping and classifying green infrastructure typologies for climate-related studies based on remote sensing data. *Urban For. Urban Green.* **37**:154–167. DOI:10.1016/j.ufug.2018.11.008
402. Belli, L., Cilfone, A., Davoli, L. et al. (2020). IoT-enabled smart sustainable cities: Challenges and approaches. *Smart Cities* **3**:1039–1071. DOI:10.3390/smartcities3030052
403. Peters, K., Elands, B. and Buijs, A. (2010). Social interactions in urban parks: Stimulating social cohesion? *Urban For. Urban Green.* **9**:93–100. DOI:10.1016/j.ufug.2009.11.003
404. Kondo, M., Hohl, B., Han, S. et al. (2016). Effects of greening and community reuse of vacant lots on crime. *Urban Stud.* **53**:3279–3295. DOI:10.1177/0042098015608058
405. Gobster, P.H., Nassauer, J.I., Daniel, T.C. et al. (2007). The shared landscape: what does aesthetics have to do with ecology? *Landsc. Ecol.* **22**:959–972. DOI:10.1007/s10980-007-9110-x
406. Nassauer, J.I. (1995). Messy ecosystems, orderly frames. *Landsc. J.* **14**:161–170. DOI:10.3368/lj.14.2.161
407. Li, J. and Nassauer, J.I. (2020). Cues to care: A systematic analytical review. *Landsc. Urban Plann.* **201**:103821. DOI:10.1016/j.landurbplan.2020.103821
408. Andersson, E., Barthel, S. and Ahrné, K. (2007). Measuring social–ecological dynamics behind the generation of ecosystem services. *Ecol. Appl.* **17**:1267–1278. DOI:10.1890/06-1116.1
409. Ahern, J. (2011). From fail-safe to safe-to-fail: Sustainability and resilience in the new urban world. *Landsc. Urban Plann.* **100**:341–343. DOI:10.1016/j.landurbplan.2011.02.021
410. Marshall, C.A.M., Wilkinson, M.T., Hadfield, P.M. et al. (2023). Urban wildflower meadow planting for biodiversity, climate and society: An evaluation at King's College, Cambridge. *Ecol. Solut. Evid.* **4**:e12243. DOI:10.1002/2688-8319.12243
411. Kabisch, N., Strohbach, M., Haase, D. et al. (2016). Urban green space availability in European cities. *Ecol. Indic.* **70**:586–596. DOI:10.1016/j.ecolind.2016.02.029
412. Vogt, J., Gillner, S., Hofmann, M. et al. (2017). Citree: A database supporting tree selection for urban areas in temperate climate. *Landsc. Urban Plann.* **157**:14–25. DOI:10.1016/j.landurbplan.2016.06.005
413. Nesbitt, L., Hotte, N., Barron, S. et al. (2017). The social and economic value of cultural ecosystem services provided by urban forests in North America: A review and suggestions for future research. *Urban For. Urban Green.* **25**:103–111. DOI:10.1016/j.ufug.2017.05.005
414. Betancourt, A.A.R. (2023). The Aesthetics of the Contemporary Urban Landscape and its Implications for Well-Being. In Proceedings of the International Conference of Contemporary Affairs in Architecture and Urbanism-ICCAUA, *6*. DOI:10.38027/iccaua2023en0157
415. Ignatieva, M., Stewart, G.H. and Meurk, C. (2011). Planning and design of ecological networks in urban areas. *Landscape Ecol. Eng.* **7**:17–25. DOI:10.1007/s11355-010-0143-y
416. Ahern, J. (2013). Urban landscape sustainability and resilience: the promise and challenges of integrating ecology with urban planning and design. *Landsc. Ecol.* **28**:1203–1212. DOI:10.1007/s10980-012-9799-z
417. Andersson, E., Haase, D., Anderson, P. et al. (2021). What are the traits of a social-ecological system: Towards a framework in support of urban sustainability. *npj Urban Sustain.* **1**:14. DOI:10.1038/s42949-020-00008-4
418. Ernstson, H., Van Der Leeuw, S.E., Redman, C.L. et al. (2010). Urban transitions: On urban resilience and human-dominated ecosystems. *Ambio* **39**:531–545. DOI:10.1007/s13280-010-0081-9

419. Nassauer, J.I. and Opdam, P. (2008). Design in science: extending the landscape ecology paradigm. *Landsc. Ecol.* **23**:633–644. DOI:10.1007/s10980-008-9226-7
420. Karolyi, G.A. and Tobin-de la, P.J. (2023). Biodiversity finance: A call for research into financing nature. *Financ. Manag.* **52**:231–251. DOI:10.1111/fima.12417
421. Statista (2021). Nature based solutions. https://www.statista.com/study/88123/nature-based-solutions-nbs/
422. Swiss Re Group (2020). A fifth of world's countries at risk. https://www.swissre.com/media/press-release/nr-20200923-biodiversity-and-ecosystems-services.html
423. Dasgupta, S.P. (2021). The economics of biodiversity: The Dasgupta review (abridged version). https://assets.publishing.service.gov.uk/media/602e92b2e90e07660f807b47/The_Economics_of_Biodiversity_The_Dasgupta_Review_Full_Report.pdf
424. Buchanan, B. (2016). Securitization: a financing vehicle for all seasons? *SSRN Journal* **138**:559–577. DOI:10.2139/ssrn.2914023
425. Mukunda, G. (2014). The price of Wall Street's power. *Harv. Bus. Rev.* **96**:70–78. https://hbr.org/2014/06/the-price-of-wall-streets-power
426. Buchanan, B., Silvola, H. and Vähämaa, E. (2025). Sustainability and private investors. *Eur. J. Finance* **31**:174–201. DOI:10.1080/1351847x.2024.2362282
427. Paulson Institute (2020). Financing nature: Closing the global biodiversity financing gap. https://www.paulsoninstitute.org/conservation/financing-nature-report/
428. Forum, W.E. (2020). Future of nature and business report. https://realsustainability.org/the-future-of-nature-and-business-world-economic-forum/
429. Aksoy, L., Buoye, A.J., Fors, M. et al. (2022). Environmental, Social and Governance (ESG) metrics do not serve services customers: A missing link between sustainability metrics and customer perceptions of social innovation. *J. Serv. Manag.* **33**:565–577. DOI:10.1108/josm-11-2021-0428
430. Schweinberger, M.C. (2024). Debt for Nature Swaps-Birth of a New Asset Class?. DOI:10.2139/ssrn.4843793
431. Chamon, M.M., Klok, E., Thakoor, M.V.V. et al. (2022). Debt-for-climate Swaps: Analysis, Design, and Implementation (International Monetary Fund), pp. 20–39. DOI:10.5089/9798400215872.001
432. Flammer, C. (2021). Corporate green bonds. *J. Financ. Econ.* **142**:499–516. DOI:10.1016/j.jfineco.2021.01.010
433. Flammer, C. (2020). Green bonds: effectiveness and implications for public policy. *Environ. Energy Policy Econ.* **1**:95–128. DOI:10.1086/706794
434. Field, A. (2022). How The Nature Conservancy's Blue Bond helped Belize restructure its debt and conserve its oceans. https://www.forbes.com/sites/annefield/2022/06/24/how-the-nature-conservancys-blue-bond-helped-belize-restructure-its-debt-and-conserve-its-oceans/
435. Hutchinson, M.C. and Lucey, B. (2024). A bibliometric and systemic literature review of biodiversity finance. *Financ. Res. Lett.* **64**:105377. DOI:10.1016/j.frl.2024.105377
436. International Chamber of Commerce (2021). Business guide to the Convention on Biological Diversity. https://iccwbo.org/news-publications/policies-reports/business-guide-to-the-united-nations-convention-on-biological-diversity/
437. Kandulu, J. and Soebarto, V. (2023). Estimating the Net Benefit of Street Trees in the Greater Adelaide Metropolitan, Australia. Environmental Institute (University of Adelaide). https://researchnow.flinders.edu.au/en/publications/estimating-the-net-benefit-of-street-trees-in-greater-adelaide-me
438. CABE (2009). Making the invisible visible: The real value of park assets. https://www.designcouncil.org.uk/fileadmin/uploads/dc/Documents/%252Athe-real-value-of-park-assets.pdf
439. Marissa Matsler, A. (2019). Making 'green'fit in a 'grey'accounting system: The institutional knowledge system challenges of valuing urban nature as infrastructural assets. *Environ. Sci. Policy* **99**:160–168. DOI:10.1016/j.envsci.2019.05.023
440. Kennedy, A., Gutterman, S., Pilgrim, B. et al. (2023). The climate change adaptation gap: An actuarial perspective. https://actuaries.org/app/uploads/2025/04/IAA_CRTF_Paper6_AdaptationGap.pdf
441. AASB 116 (2019). Property, Plant and Equipment. https://www.aasb.gov.au/admin/file/content105/c9/AASB116_08-15_COMPdec16_01-19.pdf
442. Van Dijk, A., Mount, R., Gibbons, P. et al. (2014). Environmental reporting and accounting in Australia: progress, prospects and research priorities. *Sci. Total Environ.* **473–474**:338–349. DOI:10.1016/j.scitotenv.2013.12.053
443. Langeveld, J.G., Cherqui, F., Tscheikner-Gratl, F. et al. (2022). Asset management for blue-green infrastructures: a scoping review. *Blue-Green Syst.* **4**:272–290. DOI:10.2166/bgs.2022.019
444. Kronenberg, J., Skuza, M. and Łaszkiewicz, E. (2023). To what extent do developers capitalise on urban green assets? *Urban For. Urban Green.* **87**:128063. DOI:10.1016/j.ufug.2023.128063
445. Barbier, E.B. (2011). Wetlands as natural assets. *Hydrol. Sci. J.* **56**:1360–1373. DOI:10.1080/02626667.2011.629787
446. Vardon, M., Burnett, P. and Dovers, S. (2016). The accounting push and the policy pull: balancing environment and economic decisions. *Ecol. Econ.* **124**:145–152. DOI:10.1016/j.ecolecon.2016.01.021
447. Roghani, B., Bahrami, M., Cherqui, F. et al. (2025). Exploring key characteristics of performance indicators for green infrastructure assessment. *Sci. Total Environ.* **969**:178819. DOI:10.1016/j.scitotenv.2025.178819
448. ISO 55000 (2024). Asset Management — Vocabulary, Overview and Principles (International Organization for Standardization (ISO))
449. Australia, L.S. (2025). Asset management for green infrastructure. https://www.ipwea.org/ipweacommunities/assetmanagement/am-green-infrastructure
450. Nations, U. (2024). System of Environmental-Economic Accounting - Ecosystem accounting. *Manuals and Guides*:110–176. DOI:10.5089/9789212591834.069
451. Mainelli, M. and Mills, S. (2019). Nature Smart Cities Business Model: An examination of the financing options available to local authorities to fund urban greening programmes. https://naturesmartcities.eu/public/library/Nature%20Smart%20Cities%20Business%20Model%20pdf.pdf
452. Government A (2025). Nature Repair Market. https://www.dcceew.gov.au/environment/environmental-markets/nature-repair-market?utm_source
453. Chelli, A., Brander, L. and Geneletti, D. (2025). Cost-Benefit analysis of urban nature-based solutions: A systematic review of approaches and scales with a focus on benefit valuation. *Ecosyst. Serv.* **71**:101684. DOI:10.1016/j.ecoser.2024.101684
454. Shin, E. and Kim, H. (2019). Benefit–cost analysis of green roof initiative projects: the case of Jung-gu, Seoul. *Sustainability* **11**:3319. DOI:10.3390/su11123319
455. Schoen, V., Caputo, S. and Blythe, C. (2020). Valuing physical and social output: A rapid assessment of a London community garden. *Sustainability* **12**:5452. DOI:10.3390/su12135452
456. Alves, A., Gersonius, B., Kapelan, Z. et al. (2019). Assessing the Co-Benefits of green-blue-grey infrastructure for sustainable urban flood risk management. *J. Environ. Manage.* **239**:244–254. DOI:10.1016/j.jenvman.2019.03.036
457. Wilbers, G.-J., de Bruin, K., Seifert-Dähnn, I. et al. (2022). Investing in urban blue–green infrastructure—Assessing the costs and benefits of stormwater management in a Peri-urban catchment in Oslo, Norway. *Sustainability* **14**:1934. DOI:10.3390/su14031934
458. Wild, T.C., Henneberry, J. and Gill, L. (2017). Comprehending the multiple 'values' of green infrastructure—Valuing nature-based solutions for urban water management from multiple perspectives. *Environ. Res.* **158**:179–187. DOI:10.1016/j.envres.2017.05.043
459. Manso, M., Teotónio, I., Silva, C.M. et al. (2021). Green roof and green wall benefits and costs: A review of the quantitative evidence. *Renew. Sustain. Energy Rev.* **135**:110111. DOI:10.1016/j.rser.2020.110111
460. Teotónio, I., Silva, C.M. and Cruz, C.O. (2021). Economics of green roofs and green walls: A literature review. *Sustain. Cities Soc.* **69**:102781. DOI:10.1016/j.scs.2021.102781
461. Vojinovic, Z., Keerakamolchai, W., Weesakul, S. et al. (2016). Combining ecosystem services with cost-benefit analysis for selection of green and grey infrastructure for flood protection in a cultural setting. *Environments* **4**:3. DOI:10.3390/environments4010003
462. Kim, D. and Song, S.-K. (2019). The multifunctional benefits of green infrastructure in community development: An analytical review based on 447 cases. *Sustainability* **11**:3917. DOI:10.3390/su11143917
463. Nowak, D.J., Crane, D.E. and Stevens, J.C. (2006). Air pollution removal by urban trees and shrubs in the United States. *Urban For. Urban Green.* **4**:115–123. DOI:10.1016/j.ufug.2006.01.007
464. Jones, L., Vieno, M., Fitch, A. et al. (2019). Urban natural capital accounts: Developing a novel approach to quantify air pollution removal by vegetation. *J. Environ. Econ. Policy* **8**:413–428. DOI:10.1080/21606544.2019.1597772
465. Simpson, C.H., Brousse, O., Taylor, T. et al. (2025). The mortality and associated economic burden of London's summer urban heat island effect: a modelling study. *Lancet Planet. Health* **9**:e219–e226. DOI:10.1016/s2542-5196(25)00025-7
466. Johnson, D. and Geisendorf, S. (2019). Are neighborhood-level SUDS worth it? An assessment of the economic value of sustainable urban drainage system scenarios using cost-benefit analyses. *Ecol. Econ.* **158**:194–205. DOI:10.1016/j.ecolecon.2018.12.024
467. Carter, T. and Keeler, A. (2008). Life-cycle cost–benefit analysis of extensive vegetated roof systems. *J. Environ. Manage.* **87**:350–363. DOI:10.1016/j.jenvman.2007.01.024
468. Nordman, E.E., Isely, E., Isely, P. et al. (2018). Benefit-cost analysis of stormwater green infrastructure practices for Grand Rapids, Michigan, USA. *J. Clean. Prod.* **200**:501–510. DOI:10.1016/j.jclepro.2018.07.152
469. Le Coent, P., Graveline, N., Altamirano, M.A. et al. (2021). Is-it worth investing in NBS aiming at reducing water risks? Insights from the economic assessment of three European case studies. *Nat.-Based Solut.* **1**:100002. DOI:10.1016/j.nbsj.2021.100002
470. Locatelli, L., Guerrero, M., Russo, B. et al. (2020). Socio-economic assessment of green infrastructure for climate change adaptation in the context of urban drainage planning. *Sustainability* **12**:3792. DOI:10.3390/su12093792
471. Löwe, R., Viti, M., Arnbjerg-Nielsen, K. et al. (2025). Amenity valuation of urban and peri-urban nature in high resolution on continental scale. *Nat.-Based Solut.* **7**:100214. DOI:10.1016/j.nbsj.2025.100214
472. Raymond, C.M., Frantzeskaki, N., Kabisch, N. et al. (2017). A framework for assessing and implementing the co-benefits of nature-based solutions in urban areas. *Environ. Sci. Policy* **77**:15–24. DOI:10.1016/j.envsci.2017.07.008
473. Heidari, B., Schmidt, A.R. and Minsker, B. (2022). Cost/benefit assessment of green infrastructure: Spatial scale effects on uncertainty and sensitivity. *J. Environ. Manage.* **302**:114009. DOI:10.1016/j.jenvman.2021.114009
474. Aevermann, T. and Schmude, J. (2015). Quantification and monetary valuation of urban ecosystem services in Munich, Germany. *Z. Wirtsch.* **59**:188–200. DOI:10.1515/zfw-2015-0304
475. Perini, K. and Rosasco, P. (2016). Is greening the building envelope economically sustainable? An analysis to evaluate the advantages of economy of scope of vertical greening systems and green roofs. *Urban For. Urban Green.* **20**:328–337. DOI:10.1016/j.ufug.2016.08.002
476. McPherson, E.G. (2003). A benefit-cost analysis of ten tree species in Modesto, California, USA. *J. Arboric.* **29**:1–8. DOI:10.48044/jauf.2003.001

477. Vandermeulen, V., Verspecht, A., Vermeire, B. et al. (2011). The use of economic valuation to create public support for green infrastructure investments in urban areas. *Landsc. Urban Plann.* **103**:198–206. DOI:10.1016/j.landurbplan.2011.07.010
478. Kabisch, N., Korn, H., Stadler, J. et al. (2017). Nature-based solutions to climate change adaptation in urban areas—Linkages between science, policy and practice. In Nature-Based Solutions to Climate Change Adaptation in Urban Areas (Springer Nature), pp. 1–11. DOI:10.1007/978-3-319-56091-5_1
479. Babí Almenar, J., Petucco, C., Sonnemann, G. et al. (2023). Modelling the net environmental and economic impacts of urban nature-based solutions by combining ecosystem services, system dynamics and life cycle thinking: An application to urban forests. *Ecosyst. Serv.* **60**:101506. DOI:10.1016/j.ecoser.2022.101506
480. O'Donnell, E.C., Lamond, J.E. and Thorne, C.R. (2017). Recognising barriers to implementation of Blue-Green Infrastructure: a Newcastle case study. *Urban Water J.* **14**:964–971. DOI:10.1080/1573062x.2017.1279190
481. Shazmin Shareena, A.A. and Nur Amira, A.Z. (2021). Green roof for sustainable urban flash flood control via cost benefit approach for local authority. *Urban For. Urban Green.* **57**:126876. DOI:10.1016/j.ufug.2020.126876
482. Soares, A.L., Rego, F.C., McPherson, E.G. et al. (2011). Benefits and costs of street trees in Lisbon, Portugal. *Urban For. Urban Green.* **10**:69–78. DOI:10.1016/j.ufug.2010.12.001
483. Johnson, D., See, L., Oswald, S.M. et al. (2021). A cost-benefit analysis of implementing urban heat island adaptation measures in small- and medium-sized cities in Austria. *Environ. Plan. B* **48**:2326–2345. DOI:10.1177/2399808320974689
484. Johnson, D., See, L., Oswald, S.M. et al. (2021). A cost–benefit analysis of implementing urban heat island adaptation measures in small-and medium-sized cities in Austria. *Environ. Plan. B* **48**:2326–2345. DOI:10.1177/2399808320974689
485. Connop, S., Vandergert, P., Eisenberg, B. et al. (2016). Renaturing cities using a regionally-focused biodiversity-led multifunctional benefits approach to urban green infrastructure. *Environ. Sci. Policy* **62**:99–111. DOI:10.1016/j.envsci.2016.01.013
486. Williams, C., Byrne, C., Evenden, S. et al. (2025). Urban green space provision: the case for policy-based solutions to support human health. *Med. J. Aust.* **222**:110–113. DOI:10.5694/mja2.52569
487. Tache, A.-V., Popescu, O.-C. and Petrişor, A.I. (2024). Planning Blue–Green Infrastructure for Facing Climate Change: The Case Study of Bucharest and Its Metropolitan Area. *Urban Sci.* **8**:250. DOI:10.3390/urbansci8040250
488. Dadashpoor, H. and Shahhossein, G. (2024). Defining urban sprawl: A systematic review of 130 definitions. *Habitat Int.* **146**:103039. DOI:10.1016/j.habitatint.2024.103039
489. Bressane, A., Loureiro, A.I.S., Medeiros, L.C.D.C. et al. (2024). Overcoming Barriers to Managing Urban Green Spaces in Metropolitan Areas: Prospects from a Case Study in an Emerging Economy. *Sustainability* **16**:7019. DOI:10.3390/su16167019
490. Verheij, J., Ay, D., Gerber, J.-D. et al. (2023). Ensuring public access to green spaces in urban densification: The role of planning and property rights. *Plan. Theory Pract.* **24**:342–365. DOI:10.1080/14649357.2023.2239215
491. Islam, M., Sarker, D., Hasan, J. et al. (2025). Public perceptions on urban open space and city livability in Barishal, Bangladesh. *Geol. Ecol. Landsc.* **9**:173–182. DOI:10.1080/24749508.2023.2179749
492. Simkin, R.D., Seto, K.C., McDonald, R.I. et al. (2022). Biodiversity impacts and conservation implications of urban land expansion projected to 2050. *Proc. Natl. Acad. Sci. USA* **119**:e2117297119. DOI:10.1073/pnas.2117297119
493. Stålhammar, S. and Raymond, C.M. (2024). Contested representations of benefits of urban nature in a densifying marginalised neighbourhood. *J. Environ. Plan. Manag.* **68**:2217–2241. DOI:10.1080/09640568.2024.2311822
494. van der Meulen, G.J.M., Bacchin, T.K. and van Dorst, M.J. (2023). The hydro-cultural dimension in water-sensitive urban design for Kozhikode, India. *J. Landsc. Archit.* **18**:22–33. DOI:10.1080/18626033.2023.2347142
495. ADI (2025). Green infrastructure in urban planning: Enhancing water management in cities. https://www.adi-international.org/green-infrastructure-in-urban-planning-enhancing-water-management-in-cities/
496. Visuals, A. (2025). Green spaces in urban planning: The importance of biodiversity. https://archovavisuals.com/green-spaces-in-urban-planning-of-biodiversity/
497. Xu, C., Haase, D., Su, M. et al. (2020). Assessment of landscape changes under different urban dynamics based on a multiple-scenario modeling approach. *Environ. Plan. B* **47**:1361–1379. DOI:10.1177/2399808320910161
498. Feitosa, R.C., Wilkinson, S.J., Oliveira, B. et al. (2021). Wind and greenery effects in attenuating heat stress: A case study. *J. Clean. Prod.* **291**. DOI:10.1016/j.jclepro.2021.125919
499. Li, H.W., Zhao, Y.L., Sutzl, B. et al. (2022). Impact of green walls on ventilation and heat removal from street canyons: Coupling of thermal and aerodynamic resistance. *Build. Environ.* **214**:108945. DOI:10.1016/j.buildenv.2022.108945
500. Iaria, J. and Susca, T. (2022). Analytic Hierarchy Processes (AHP) evaluation of green roof- and green wall- based UHI mitigation strategies via ENVI-met simulations. *Urban Clim.* **46**:101293. DOI:10.1016/j.uclim.2022.101293
501. Blanco, I., Schettini, E. and Vox, G. (2019). Predictive model of surface temperature difference between green facades and uncovered wall in Mediterranean climatic area. *Appl. Therm. Eng.* **163**:114406. DOI:10.1016/j.applthermaleng.2019.114406
502. Morabito, M., Crisci, A., Guerri, G. et al. (2021). Surface urban heat islands in Italian metropolitan cities: Tree cover and impervious surface influences. *Sci. Total Environ.* **751**:142334. DOI:10.1016/j.scitotenv.2020.142334
503. Kostadinovic, D., Jovanovic, M., Bakic, V. et al. (2022). Experimental investigation of summer thermal performance of the green roof system with mineral wool substrate. *Build. Environ.* **217**:109061. DOI:10.1016/j.buildenv.2022.109061
504. Lynn, B.H. and Lynn, I.M. (2020). The impact of cool and green roofs on summertime temperatures in the cities of Jerusalem and Tel Aviv. *Sci. Total Environ.* **743**:140568. DOI:10.1016/j.scitotenv.2020.140568
505. Tan, H., Kotamarthi, R., Wang, J. et al. (2023). Impact of different roofing mitigation strategies on near-surface temperature and energy consumption over the Chicago metropolitan area during a heatwave event. *Sci. Total Environ.* **860**:160508. DOI:10.1016/j.scitotenv.2022.160508
506. Fini, A., Frangi, P., Mori, J. et al. (2017). Nature based solutions to mitigate soil sealing in urban areas: Results from a 4-year study comparing permeable, porous, and impermeable pavements. *Environ. Res.* **156**:443–454. DOI:10.1016/j.envres.2017.03.051
507. Coutts, A.M., White, E.C., Tapper, N.J. et al. (2016). Temperature and human thermal comfort effects of street trees across three contrasting street canyon environments. *Theor. Appl. Climatol.* **124**:55–68. DOI:10.1007/s00704-015-1409-y
508. Cai, Y., Li, C., Ye, L. et al. (2022). Effect of the roadside tree canopy structure and the surrounding on the daytime urban air temperature in summer. *Agric. For. Meteorol.* **316**:108850. DOI:10.1016/j.agrformet.2022.108850
509. Schwaab, J., Meier, R., Mussetti, G. et al. (2021). The role of urban trees in reducing land surface temperatures in European cities. *Nat. Commun.* **12**:6763. DOI:10.1038/s41467-021-27052-3
510. Zonato, A., Martilli, A., Gutierrez, E. et al. (2021). Exploring the Effects of Rooftop Mitigation Strategies on Urban Temperatures and Energy Consumption. *JGR. Atmospheres* **126**:e2021JD035002. DOI:10.1029/2021JD035002
511. Sadeghi, M., Chaston, T., Hanigan, I. et al. (2022). The health benefits of greening strategies to cool urban environments – A heat health impact method. *Build. Environ.* **207**:108546. DOI:10.1016/j.buildenv.2021.108546
512. Haddad, S., Paolini, R., Ulpiani, G. et al. (2020). Holistic approach to assess co-benefits of local climate mitigation in a hot humid region of Australia. *Sci. Rep.* **10**:14216. DOI:10.1038/s41598-020-71079-1
513. Xi, C., Wang, D. and Cao, S.-J. (2023). Impacts of trees-grass area ratio on thermal environment, energy saving, and carbon benefits. *Urban Clim.* **47**:101393. DOI:10.1016/j.uclim.2022.101393
514. Su, W., Zhang, W. and Chang, Q. (2025). Tailored green and blue infrastructure for heat mitigation under renewal planning of urban blocks in Beijing. *Sci. Total Environ.* **967**:178759. DOI:10.1016/j.scitotenv.2025.178759
515. Blair, R.B. and Johnson, E.M. (2008). Suburban habitats and their role for birds in the urban–rural habitat network: points of local invasion and extinction? *Landsc. Ecol.* **23**:1157–1169. DOI:10.1007/s10980-008-9267-y
516. Cortinovis, C., Olsson, P., Boke-Olén, N. et al. (2022). Scaling up nature-based solutions for climate-change adaptation: Potential and benefits in three European cities. *Urban For. Urban Green.* **67**:127450. DOI:10.1016/j.ufug.2021.127450
517. Pogliani, L., Ronchi, S., Arcidiacono, A. et al. (2023). Regeneration in an ecological perspective. Urban and territorial equalisation for the provision of ecosystem services in the Metropolitan City of Milan. *Land Use Policy* **129**:106606. DOI:10.1016/j.landusepol.2023.106606
518. Pauleit, S., Zölch, T., Hansen, R. et al. (2017). Nature-based solutions and climate change–four shades of green. Nature-based solutions to climate change adaptation in urban areas: Linkages between science. *Policy and Practice*:29–49. DOI:10.1007/978-3-319-56091-5_3
519. Xi, C., Ren, C., Zhang, R. et al. (2023). Nature-based solution for urban traffic heat mitigation facing carbon neutrality: sustainable design of roadside green belts. *Appl. Energy* **343**:121197. DOI:10.1016/j.apenergy.2023.121197
520. Xi, C., Han, L., Wang, J. et al. (2023). How can greenery space mitigate urban heat island? An analysis of cooling effect, carbon sequestration, and nurturing cost at the street scale. *J. Clean. Prod.* **419**:138230. DOI:10.1016/j.jclepro.2023.138230
521. Xi, C., Ding, J., Wang, J. et al. (2022). Nature-based solution of greenery configuration design by comprehensive benefit evaluation of microclimate environment and carbon sequestration. *Energy Build.* **270**:112264. DOI:10.1016/j.enbuild.2022.112264
522. Kanniah, K.D. (2017). Quantifying green cover change for sustainable urban planning: A case of Kuala Lumpur, Malaysia. *Urban For. Urban Green.* **27**:287–304. DOI:10.1016/j.ufug.2017.08.016
523. Tabatabaee, S., Mahdiyar, A., Mohandes, S.R. et al. (2022). Towards the development of a comprehensive lifecycle risk assessment model for green roof implementation. *Sustain. Cities Soc.* **76**:103404. DOI:10.1016/j.scs.2021.103404
524. Rosa, D.W.B., Hot, C.V.P.S., Gomes, I.T. et al. (2024). Water quality benefits of implementing Green and Blue Infrastructure in a peri-urban catchment–Case study of a Brazilian metropolis. *J. Clean. Prod.* **478**:143943. DOI:10.1016/j.jclepro.2024.143943
525. Barbosa, A., Martín, B., Hermoso, V. et al. (2019). Cost-effective restoration and conservation planning in Green and Blue Infrastructure designs. A case study on the Intercontinental Biosphere Reserve of the Mediterranean: Andalusia (Spain)–Morocco. *Sci. Total Environ.* **652**:1463–1473. DOI:10.1016/j.scitotenv.2018.10.416
526. D'amato, D. and Korhonen, J. (2021). Integrating the green economy, circular economy and bioeconomy in a strategic sustainability framework. *Ecol. Econ.* **188**:107143. DOI:10.1016/j.ecolecon.2021.107143
527. Fuenfschilling, L., Frantzeskaki, N. and Coenen, L. (2019). Urban experimentation & sustainability transitions. *Eur. Plan. Stud.* **27**:219–228. DOI:10.1080/09654313.2018.1532977
528. Tapia, F. and Reith, A. (2025). Integrating service design principles in NbS implementation: Insights from Szombathely (Hungary). *City Environ. Interact.* **26**:100188. DOI:10.1016/j.cacint.2025.100188

529. Tapia, F., Ochoa-Peralta, D. and Reith, A. (2025). From design to action: Service design tools for enhancing collaboration in nature-based solutions implementation. *J. Environ. Manage.* **379**:124739. DOI:10.1016/j.jenvman.2025.124739
530. Ahmed, A. and Puppim de Oliveira, J.A. (2017). Integration of biodiversity in urban planning instruments in developing countries: the case of Kumasi Metropolitan Assembly, Ghana. *J. Environ. Plan. Manag.* **60**:1741–1764. DOI:10.1080/09640568.2016.1255183
531. Kramer, J., Silverton, S. and Späth, P. (2024). Urban governance arrangements for sustainability and justice—linking theory with experience. *Urban Transform.* **6**:6. DOI:10.1186/s42854-024-00064-4
532. Li, K., Shao, J., Lin, R. et al. (2025). The research on the evaluation framework and application of urban three-dimensional (aboveground and underground) regeneration sensitivity——Taking Kunshan Old City in China as an example. *Tunn. Undergr. Sp. Technol.* **159**:106428. DOI:10.1016/j.tust.2025.106428
533. Zhang, C., Xi, C., Feng, Z. et al. (2022). Passive design for green buildings by using green glass space and earth air tunnel. *Energy Build.* **273**:112367. DOI:10.1016/j.enbuild.2022.112367
534. Cortes, A., Tadeu, A., Santos, M.I. et al. (2021). Innovative module of expanded cork agglomerate for green vertical systems. *Build. Environ.* **188**:107461. DOI:10.1016/j.buildenv.2020.107461
535. Wang, H., Zhao, J., Lu, J. et al. (2025). Combined impacts of vertical greening and permeable pavement systems on street Canyons' microclimate in hot and humid regions in China. *Urban Clim.* **59**:102333. DOI:10.1016/j.uclim.2025.102333
536. Parada, F., Gabarrell, X., Rufí-Salís, M. et al. (2021). Optimizing irrigation in urban agriculture for tomato crops in rooftop greenhouses. *Sci. Total Environ.* **794**:148689. DOI:10.1016/j.scitotenv.2021.148689
537. Lai, S.H., Chin, R.J., Soo, E.Z.X. et al. (2024). Optimal Design of Subsurface Conveyance System Based Bio-Ecological Drainage System Simulation. *KSCE J. Civ. Eng.* **28**:2564–2570. DOI:10.1007/s12205-024-1475-8
538. Wu, L. and Convertino, M. (2025). Ecological corridor design for ecoclimatic regulation: Species as eco-engineers. *Ecol. Indic.* **171**:113149. DOI:10.1016/j.ecolind.2025.113149
539. Castellar, J.A.C., Popartan, L.A., Pucher, B. et al. (2024). What does it take to renature cities? An expert-based analysis of barriers and strategies for the implementation of nature-based solutions. *J. Environ. Manage.* **354**:120385. DOI:10.1016/j.jenvman.2024.120385
540. Jang, N. and Doyon, A. (2024). Increasing knowledge and trust to overcome barriers to green infrastructure implementation: a Vancouver case study. *Urban Water J.* **21**:802–811. DOI:10.1080/1573062x.2024.2351856
541. Okour, Y. and Shaweesh, H. (2024). Identifying the barriers to green infrastructure implementation in semi-arid urban areas using the DPSIR framework: A case study of Amman, Jordan. *City Environ. Interact.* **24**:100165. DOI:10.1016/j.cacint.2024.100165
542. Grunewald, K., Bastian, O., Louda, J. et al. (2021). Lessons learned from implementing the ecosystem services concept in urban planning. *Ecosyst. Serv.* **49**:101273. DOI:10.1016/j.ecoser.2021.101273
543. Borelli, S., Conigliaro, M., Quaglia, S. et al. (2018). Urban and Peri-urban agroforestry as multifunctional land use. In Agroforestry: Anecdotal to Modern Science (Springer), pp. 705–724. DOI:10.1007/978-981-10-7650-3_28
544. Johns, C.M. (2019). Understanding barriers to green infrastructure policy and stormwater management in the City of Toronto: a shift from grey to green or policy layering and conversion? *J. Environ. Plan. Manag.* **62**:1377–1401. DOI:10.1080/09640568.2018.1496072
545. Thorne, C.R., Lawson, E.C., Ozawa, C. et al. (2018). Overcoming uncertainty and barriers to adoption of Blue-Green Infrastructure for urban flood risk management. *J. Flood Risk Manag.* **11**:S960–S972. DOI:10.1111/jfr3.12218
546. Dhakal, K.P. and Chevalier, L.R. (2017). Managing urban stormwater for urban sustainability: Barriers and policy solutions for green infrastructure application. *J. Environ. Manage.* **203**:171–181. DOI:10.1016/j.jenvman.2017.07.065
547. Chaffin, B.C., Shuster, W.D., Garmestani, A.S. et al. (2016). A tale of two rain gardens: Barriers and bridges to adaptive management of urban stormwater in Cleveland, Ohio. *J. Environ. Manage.* **183**:431–441. DOI:10.1016/j.jenvman.2016.06.025
548. Brudermann, T. and Sangkakool, T. (2017). Green roofs in temperate climate cities in Europe–An analysis of key decision factors. *Urban For. Urban Green.* **21**:224–234. DOI:10.1016/j.ufug.2016.12.008
549. Mhajerani, N. (2021). Green infrastructure planning and siting. In Design Criteria for Green Infrastructure in the Right-of-Way (Engineering & Construction Services Design & Construction), pp. 11–27
550. Bhat, M.Y. and Soontha, L. (2025). Economic valuation of environment: An introduction to valuation methods. In Non-Market Valuation in South Asia (Routledge), pp. 21–46
551. AIPH (International Association of Horticultural Producers) (2025). Green City Case Study: Melbourne, Australia: Grey to Green. https://aiph.org/green-city-case-studies/melbourne-australia/
552. Bonilla-Jurado, D., Zumba, E., Lucio-Quintana, A. et al. (2024). Advancing university education: exploring the benefits of Education for Sustainable Development. *Sustainability* **16**:7847. DOI:10.3390/su16177847
553. Husic, D.W. (2024). Reframing sustainability initiatives in higher education. *Sustain. Earth Rev.* **7**:5. DOI:10.1186/s42055-024-00076-9
554. Abdallah, A.K., Ismail, L.S. and Alkaabi, A.M. (2025). Green Careers: Educating for the Future of Sustainability. In Legal Frameworks and Educational Strategies for Sustainable Development (IGI Global), pp. 337–366. DOI:10.4018/979-8-3693-2987-0.ch017
555. Thake, A.M. (2025). Transitioning to a green economy-the impact on the labor market and workforce skills. In Greening Our Economy for a Sustainable Future (Elsevier), pp. 163–175. DOI:10.1016/b978-0-443-23603-7.00013-3
556. Shuvo, F.K., Mazumdar, S. and Labib, S.M. (2021). Walkability and greenness do not walk together: investigating associations between greenness and walkability in a large metropolitan city context. *Int. J. Environ. Res. Public Health* **18**:4429. DOI:10.3390/ijerph18094429
557. Cervero, R. and Duncan, M. (2003). Walking, bicycling, and urban landscapes: evidence from the San Francisco Bay Area. *Am. J. Public Health* **93**:1478–1483. DOI:10.2105/ajph.93.9.1478
558. Frank, L.D., Sallis, J.F., Saelens, B.E. et al. (2010). The development of a walkability index: application to the Neighborhood Quality of Life Study. *Br. J. Sports Med.* **44**:924–933. DOI:10.1136/bjsm.2009.058701
559. Southworth, M. (2005). Designing the walkable city. *J. Urban Plan. Dev.* **131**:246–257. DOI:10.1061/(asce)0733-9488(2005)131:4(246)
560. Albala, P.L.R. and Mülfarth, R.C.K. (2024). Pedestrian pathways: an index based on the interrelations between walkability and environmental comfort. *Ambiente Constr.* **24**:e138386. DOI:10.1590/s1678-86212024000100777
561. Wedyan, M. and Saeidi-Rizi, F. (2025). Assessing the impact of walkability indicators on health outcomes using machine learning algorithms: A case study of Michigan. *Travel Behav. Soc.* **39**:100983. DOI:10.1016/j.tbs.2025.100983
562. Taylor, L. and Hochuli, D.F. (2015). Creating better cities: how biodiversity and ecosystem functioning enhance urban residents' wellbeing. *Urban Ecosyst.* **18**:747–762. DOI:10.1007/s11252-014-0427-3
563. Derek, M., Kulczyk, S., Grzyb, T. et al. (2025). 'This is my magical place here'. Linking cultural ecosystem services and landscape elements in urban green spaces. *Ecosyst. Serv.* **71**:101699. DOI:10.1016/j.ecoser.2025.101699
564. Shafray, E. and Kim, S. (2017). A study of walkable spaces with natural elements for urban regeneration: A focus on cases in Seoul, South Korea. *Sustainability* **9**:587. DOI:10.3390/su9040587
565. World Health Organization (WHO) (2016). Health economic assessment tools (HEAT) for walking and for cycling. https://www.who.int/europe/tools-and-toolkits/health-economic-assessment-tool-for-walking-and-cycling
566. Ewing, R. and Handy, S. (2009). Measuring the unmeasurable: Urban design qualities related to walkability. *J. Urban Des.* **14**:65–84. DOI:10.1080/13574800802451155
567. Fior, M., Galuzzi, P., Pasqui, G. et al. (2022). Safety, Green and Blue Networks, Active Mobility and Walkability. In Discovering Proximity: Generating New Urbanity—An Action Research for Milan (Springer), pp. 71–102. DOI:10.1007/978-3-031-08958-9_4
568. Meerow, S. and Newell, J.P. (2017). Spatial planning for multifunctional green infrastructure: Growing resilience in Detroit. *Landsc. Urban Plann.* **159**:62–75. DOI:10.1016/j.landurbplan.2016.10.005
569. Wolf, K.L., Lam, S.T., McKeen, J.K. et al. (2020). Urban trees and human health: A scoping review. *Int. J. Environ. Res. Public Health* **17**:4371. DOI:10.3390/ijerph17124371
570. Marquet, O., Floyd, M.F., James, P. et al. (2020). Associations between worksite walkability, greenness, and physical activity around work. *Environ. Behav.* **52**:139–163. DOI:10.1177/0013916518797165
571. Clark, C., Sbihi, H., Tamburic, L. et al. (2017). Association of long-term exposure to transportation noise and traffic-related air pollution with the incidence of diabetes: a prospective cohort study. *Environ. Health Perspect.* **125**:087025. DOI:10.1289/ehp1279
572. Petersen, C.J., Russel, D.J., Jensen, A. et al. (2024). Walkable maps and policy innovation for nature: a novel methodology for understanding policy learning. *Int. J. Qual. Methods* **23**:16094069241254006. DOI:10.1177/16094069241254006
573. de Oliveira Rolo, D.A.d.M., Gallardo, A.L.C.F., Ribeiro, A.P. et al. (2022). Local society perception on ecosystem services as an adaptation strategy in urban stream recovery programs in the city of São Paulo, Brazil. *Environ. Manage.* **69**:684–698. DOI:10.1007/s00267-021-01471-0
574. Petroni, M.L., Siqueira-Gay, J. and Gallardo, A.L.C.F. (2022). Understanding land use change impacts on ecosystem services within urban protected areas. *Landsc. Urban Plann.* **223**:104404. DOI:10.1016/j.landurbplan.2022.104404
575. Leslie, E., Coffee, N., Frank, L. et al. (2007). Walkability of local communities: using geographic information systems to objectively assess relevant environmental attributes. *Health Place* **13**:111–122. DOI:10.1016/j.healthplace.2005.11.001
576. Casteli Figueiredo Gallardo, A.L. and Bond, A. (2025). A Nature-based Solutions framework for embedding climate change mitigation and adaptation into urban land use plans through Strategic Environmental Assessment (SEA). *Environ. Manage.* **75**:256–271. DOI:10.1007/s00267-024-02073-2

The Innovation, Volume 7

Supplemental Information

Overlooked considerations in prescribing green and blue infrastructure solutions for urban environments

Prashant Kumar, Karina Corada Perez, Akash Biswal, Hao Sun, Anubhav Kumar Dwivedi, Sarkawt Hama, Soheila Khalili, Ajit Ahlawat, Maria de Fatima Andrade, Ronaldo Adriano Alves, Emannuelly A. Amaral dos Santos, Maria Athanassiadou, Camilo Bastos Ribeiro, Prabin Bhusal, Miguel Luiz Bucalem, Bonnie G. Buchanan, Leticia Figueiredo Candido, Shi-Jie Cao, Amarilis Lucia Casteli Figueiredo Gallardo, Ruidong Chang, Amanda K. Chaves Ribeiro, Brian Considine, Regina Maura de Miranda, Letícia Aparecida de Paiva, Priyanka de Souza, Marco A. Franco, Edmilson D. Freitas, H. Christopher Frey, Marco F. Funari, Bruno Furieri, John Gallagher, Leandro Luiz Giatti, Marcos Jeronimo Goroski Rambalducci, Christos H. Halios, Felicity Harris, Leonardo Hoinaski, Colin Horton, Yuhan Huang, Laurence Jones, Robyn Jones, John Kandulu, Madhusudan Katti, Giuliano Maselli Locosselli, Augusto Akio Lucchezi Miyahara, Jorge Alberto Martins, Leila Droprinchinski Martins, Mauricio Cruz Mantoani, Roberta Consentino Kronka Mülfarth, Yasmin Kaore Lago Kitagawa, Willian Lemker Andreão, Jackson Lemons, Giulia Mariano Machado, Shelagh K. Malham, Meredith P. Martin, Maria Clara V.M. Starling, Aonghus McNabola, Otavio Medeiros Sobrinho, Eugene Mohareb, Erick G. Sperandio Nascimento, Thiago Nogueira, Gwilym Owen, Rajan Parajuli, Hari Prasad Pandey, Rizzieri Pedruzzi, Pedro José Pérez Martínez, Janaina Antonino Pinto, Jorge Armando Piscoya Santibañez, Shila Pokhrel, Paula Lelis Rabelo Albala, Neyval C. Reis, Anderson P. Rudke, Devendra Saroj, Yiming Sui, Veronica Soebarto, Yonatal Tefera, Taciana Toledo de Almeida Albuquerque, Bruna Lima Veras Maia, Fang Wang, Jannis Wenk, Robson Will, Carmel Williams, Hannah Sloan Wood, Qingyun Wu, Chang Xi, Russell Yates, and Runming Yao

Supplementary Information (SI)

for

Overlooked Considerations in Prescribing Green and Blue Infrastructure Solutions for Urban Environments

Prashant Kumar,[1,2,16,19,*] Karina Corada Perez,[3] Akash Biswal,[1] Hao Sun,[1] Anubhav Kumar Dwivedi,[1] Sarkawt Hama,[1] Soheila Khalili,[1,2] Ajit Ahlawat,[4,5] Maria de Fatima Andrade,[6] Ronaldo Adriano Alves,[7] Emannuelly A. Amaral dos Santos,[8] Maria Athanassiadou,[9] Camilo Bastos Ribeiro,[10] Prabin Bhusal,[11,12] Miguel Luiz Bucalem,[13] Bonnie G. Buchanan,[14] Leticia Figueiredo Candido,[15] Shi-Jie Cao,[1,16] Amarilis Lucia Casteli Figueiredo Gallardo,[13,17] Ruidong Chang,[18] Amanda K. Chaves Ribeiro,[8] Brian Considine,[19,41] Regina Maura de Miranda,[20] Letícia Aparecida de Paiva,[21] Priyanka de Souza,[22,23] Marco A. Franco,[6] Edmilson D. Freitas,[6] H. Christopher Frey,[24] Marco F. Funari,[1,2] Bruno Furieri,[25] John Gallagher,[19] Leandro Luiz Giatti,[26] Marcos Jeronimo Goroski Rambalducci,[27] Christos H. Halios,[28] Felicity Haris,[29] Leonardo Hoinaski,[10] Colin Horton,[30] Yuhan Huang,[31] Laurence Jones,[32,33] Robyn Jones,[34] John Kandulu,[35] Madhusudan Katti,[11] Giuliano Maselli Locosselli,[15,36] Augusto Akio Lucchezi Miyahara,[15] Jorge Alberto Martins,[27] Leila Droprinchinski Martins,[27] Mauricio Cruz Mantoani,[6] Roberta Consentino Kronka Mülfarth,[37] Yasmin Kaore Lago Kitagawa,[38] Willian Lemker Andreão,[39] Jackson Lemons,[11] Giulia Mariano Machado,[26] Shelagh K Malham,[40] Meredith P. Martin,[11] Maria Clara V. M. Starling,[8] Aonghus McNabola,[19,41] Otavio Medeiros Sobrinho,[8] Eugene Mohareb,[28] Erick G. Sperandio Nascimento,[1,42,43] Thiago Nogueira,[26] Gwilym Owen,[44] Rajan Parajuli,[11] Hari Prasad Pandey,[45,46] Rizzieri Pedruzzi,[47] Pedro José Pérez Martínez,[21] Janaina Antonino Pinto,[48] Jorge Armando Piscoya Santibañez,[6] Shila Pokhrel,[11,46] Paula Lelis Rabelo Albala,[37,59] Neyval C. Reis,[25] Anderson P. Rudke,[8] Devendra Saroj,[1,2] Yiming Sui,[28] Veronica Soebarto,[18] Yonatal Tefera,[49] Taciana Toledo de Almeida Albuquerque,[8] Bruna Lima Veras Maia,[8] Fang Wang,[50,51,52] Jannis Wenk,[53,54] Robson Will,[10] Carmel Williams,[49] Hannah Sloan Wood,[55] Qingyun Wu,[31] Chang Xi,[16] Russell Yates,[56] Runming Yao,[27,57,58]

[1]Global Centre for Clean Air Research (GCARE), School of Engineering, Civil and Environmental Engineering, Faculty of Engineering and Physical Sciences, University of

[*]Correspondence: p.kumar@surrey.ac.uk (P. A.)

Surrey, Guildford GU2 7XH, United Kingdom
[2]Institute for Sustainability, University of Surrey, Guildford GU2 7XH, United Kingdom
[3]Sustainability Research Institute, University of East London, London E16 2RD, United Kingdom
[4]Department of Geoscience and Remote Sensing, Delft University of Technology (TU Delft), Delft 2628 CN, The Netherlands
[5]Leibniz Institute for Tropospheric Research, e.V. (TROPOS), Leipzig 04318, Germany
[6]Institute of Astronomy, Geophysics and Atmospheric Sciences, Department of Atmospheric Sciences, University of São Paulo, São Paulo 05508-090, Brazil
[7] Department of Geography, State University of Londrina, Londrina 86057-970, Brazil
[8]Department of Sanitary and Environmental Engineering, School of Engineering, Federal University of Minas Gerais, Belo Horizonte 31270-901, Brazil
[9]Urban Climate Applications, Met Office, Exeter EX1 3PB, United Kingdom
[10]Department of Sanitary and Environmental Engineering, Federal University of Santa Catarina, Florianópolis 88040-070, Brazil
[11]Department of Forestry and Environmental Resources, North Carolina State University, Raleigh, North Carolina 27695, United States
[12]Institute of Forestry, Tribhuvan University, Pokhara 33700, Nepal
[13]Polytechnic School, University of São Paulo, São Paulo 05508-010, Brazil
[14]Sustainable and Explainable Fintech (SAEF) Center, University of Surrey, Guildford GU2 7XH, United Kingdom
[15]Institute of Environmental Research from the State of São Paulo, São Paulo 01061-970, Brazil
[16]School of Architecture, Southeast University, 2 Sipailou, Nanjing 210096, China
[17]Smart and Sustainable Cities Program at the University Nove de Julho, São Paulo 01525-000, Brazil
[18]School of Architecture and Civil Engineering, The University of Adelaide, Adelaide SA 5005, Australia
[19]Department of Civil, Structural & Environmental Engineering, Trinity College Dublin, the University of Dublin, Dublin D02 PN40, Ireland
[20]School of Arts, Sciences and Humanities, University of São Paulo, São Paulo 03828-000 Brazil

[21]Faculty of Engineering, Architecture and Urbanism, University of Campinas, Campinas13083-970, Brazil
[22]Department of Urban and Regional Planning, University of Colorado Denver, Denver CO 80202, United States
[23]CU Population Center, University of Colorado Boulder, Boulder CO 80302, United States
[24]Department of Civil, Construction, and Environmental Engineering, North Carolina State University, Raleigh NC 27606, United States
[25]Department of Environmental Engineering, Federal University of Espírito Santo, Vitória 29075-910, ES, Brazil
[26]Department of Environmental Health, School of Public Health, University of São Paulo, São Paulo 01246904, Brazil
[27]Federal University of Technology, Parana 80230-910, Brazil
[28]School of Built Environment, University of Reading, Reading RG6 6DF, United Kingdom
[29]Portsmouth City Council, Portsmouth PO1 2AL, United Kingdom
[30]Rugby Borough Council, Rugby CV21 2RR, United Kingdom
[31]Centre for Green Technology, School of Civil and Environmental Engineering, University of Technology Sydney, NSW 2007, Australia
[32]UK Centre for Ecology & Hydrology, Deiniol Road, Bangor LL57 2UW, United Kingdom
[33]Liverpool Hope University, Department of Geography and Environmental Science, Liverpool L16 9JD, United Kingdom
[34]School of Psychology and Sport Science, Bangor University, Bangor LL57 2DG, United Kingdom
[35]College of Business, Government and Law, Flinders University, Bedford Park, South Australia 5042, Australia
[36]Center for Nuclear Energy in Agriculture, University of São Paulo, São Paulo 13416-000, Brazil
[37]Faculty of Architecture, Urbanism and Design, University of São Paulo, São Paulo 05508-080, Brazil
[38]Foundation for Scientific and Cultural Development, Federal University of Lavras, Lavras 37203-202, Brazil
[39]ArcelorMittal, Global Research and Development, Espirito Santo 29161-376, Brazil, Brazil

[40]School of Ocean Sciences, Bangor University, Menai Bridge, Anglesey LL59 5AU, United Kingdom
[41]School of Engineering, RMIT University, Melbourne VIC 3000, Australia
[42]Surrey Institute for People-Centred Artificial Intelligence, Faculty of Engineering and Physical Sciences, University of Surrey, Guildford GU2 7XH, United Kingdom
[43]Stricto Sensu Department, SENAI CIMATEC University, Salvador 41650-010, Brazil
[44]Resilience Unit, Cardiff Council, Cardiff CF10 4UW, United Kingdom
[45]University of Southern Queensland, Toowoomba, Queensland 4350, Australia
[46]Ministry of Forests and Environment, Government of Nepal, Kathmandu 44600, Nepal
[47]Department of Sanitary and Environmental Engineering, Rio de Janeiro State University. Rio de Janeiro 20550-013, Brazil
[48]Department of Infrastructure and Environment, Faculty of Civil Engineering, Architecture and Urbanism, State University of Campinas, São Paulo 13083-889, Brazil
[49]School of Public Health, The University of Adelaide, Adelaide SA 5005, Australia
[50]State Key Laboratory of Soil and Sustainable Agriculture, Institute of Soil Science, Chinese Academy of Sciences, Nanjing 210008, China
[51]University of Chinese Academy of Sciences, Beijing 100049, China
[52]Joint FAO/IAEA Centre of Nuclear Techniques in Food and Agriculture, International Atomic Energy Agency, Vienna 1400, Austria
[53]Federal Institute of Hydrology (BfG), Department G - Qualitative Hydrology, Am Mainzer Tor 1, Koblenz 56068, Germany
[54]Department of Chemical Engineering, University of Bath, BA2 7AY, United Kingdom
[55]Independent Consultant, Copenhagen 2200, Denmark
[56]Surrey County Council, Woodhatch, Reigate RH2 8EF, United Kingdom
[57]Joint International Research Laboratory of Green Buildings and Built Environments (Ministry of Education), Chongqing University, Chongqing 400045, China
[58]National Centre for International Research of Low-carbon and Green Buildings (Ministry of Science and Technology), Chongqing University, Chongqing 400045, China
[59]Faculty of Architecture and Urbanism, University of Brasília, Brasília 70904-900, Brazil

This document includes:

- Figures S1-S10
- Tables S1-S13
- Sections S1-S3

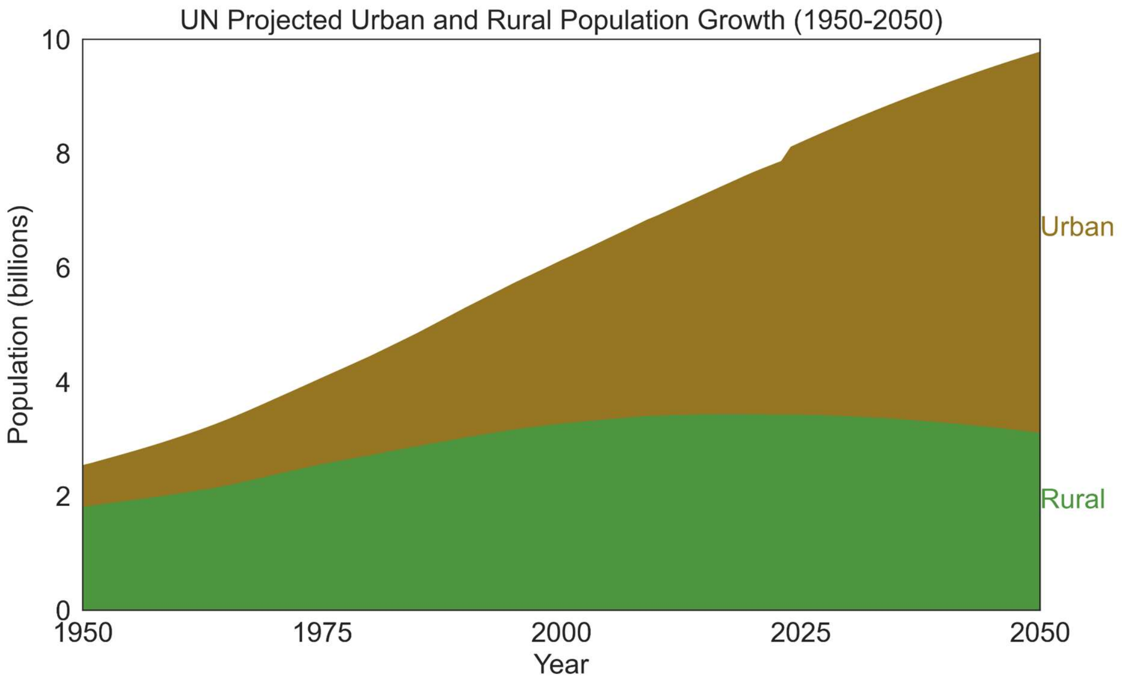

Figure S1. UN projected urban and rural population growth from 1950-2050 (Data Source: https://ourworldindata.org/urbanization).

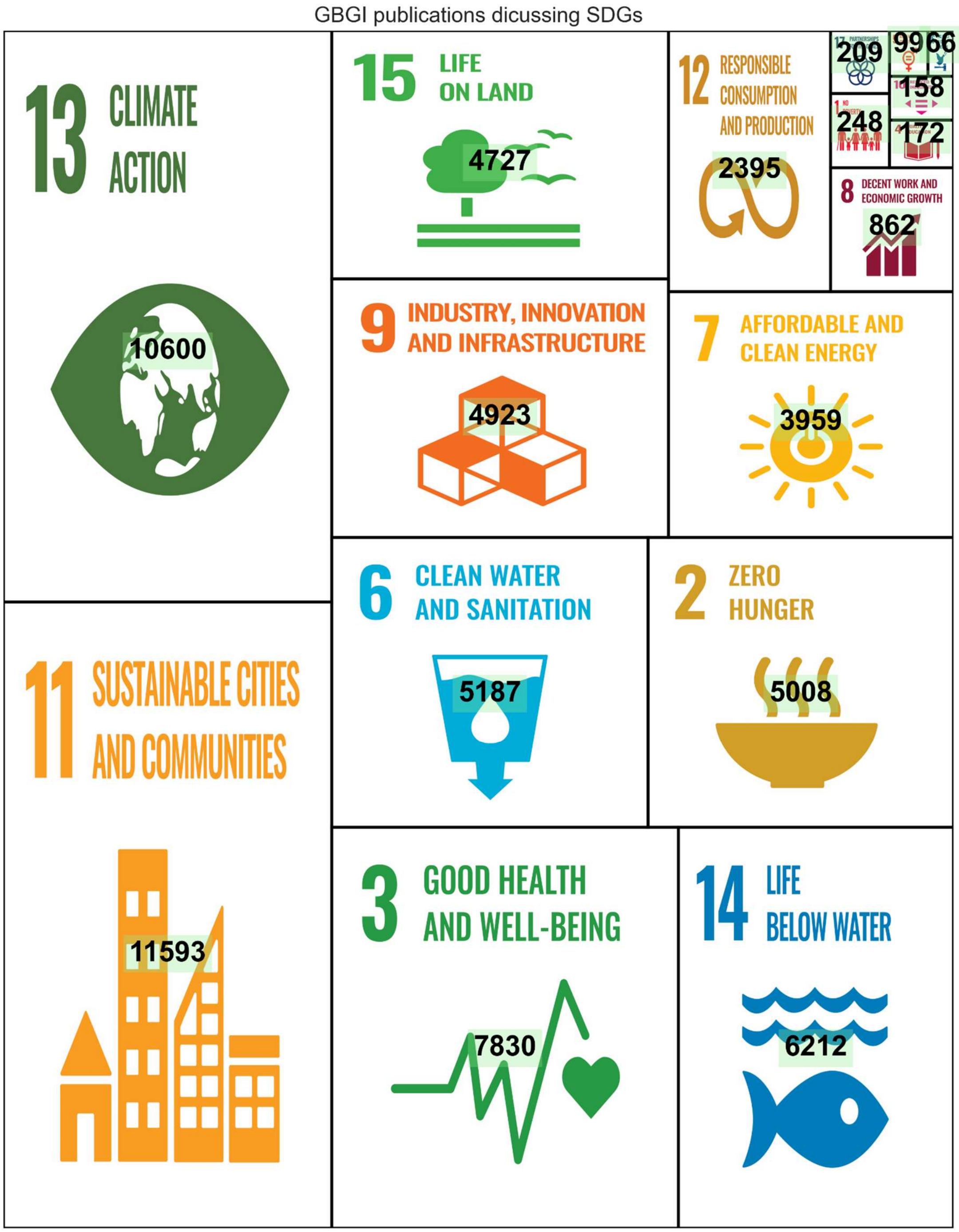

Figure S2. Treemap visualisation of publication records obtained after the Web of Science portal (Clarivate) using the search query: Green Infrastructure (All Fields) OR Nature-based Solutions (All Fields) AND "Air Pollution" (Keyword Plus®) AND "Climate Change" (Keyword Plus®) AND "Temperature" (Keyword Plus®) AND "Flooding" (Keyword Plus®). The data reflects the distribution of research focused on the intersection of urban environmental challenges and nature-based mitigation strategies. The size of box is an indication for the number of publications linked with the SDGs and the text inside the light green box shows the number of matching publications.

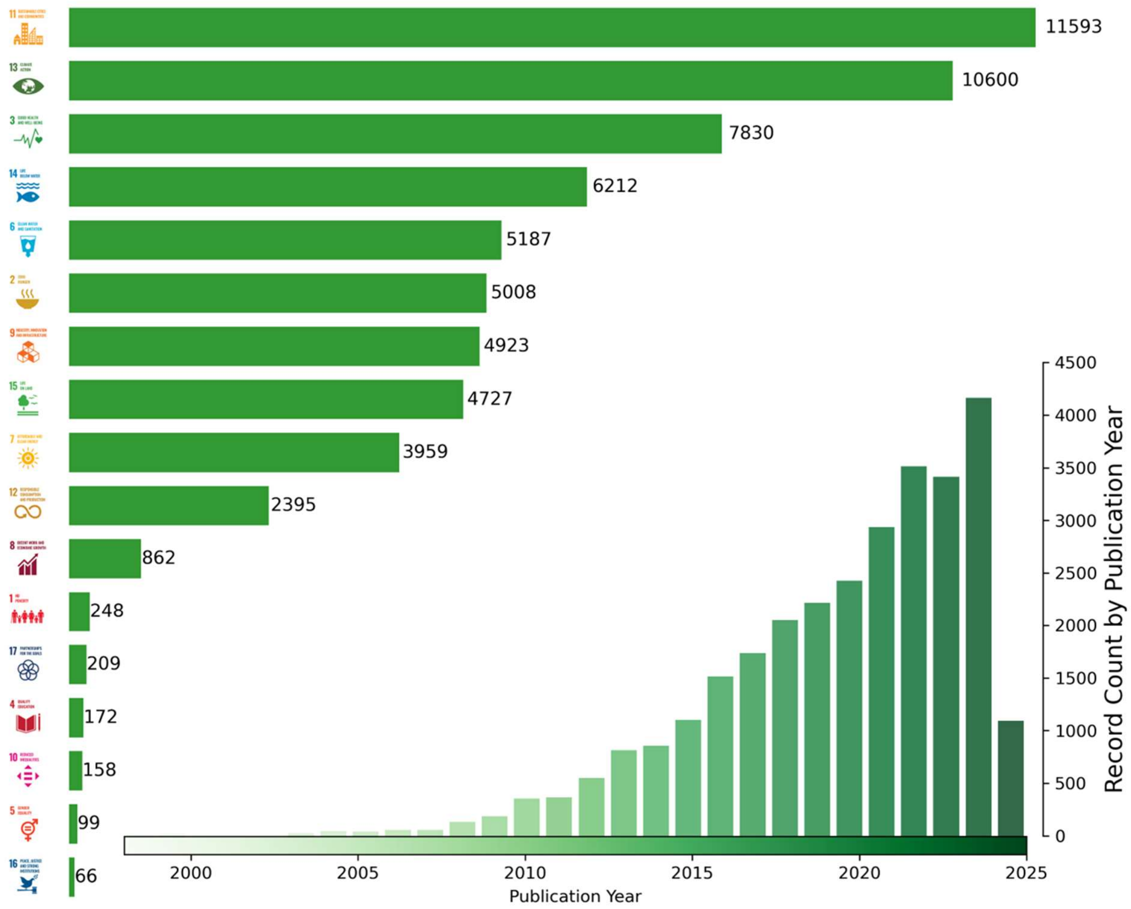

Figure S3. The horizontal bars represent the total number of publications associated with each SDG, and the vertical bar chart in the bottom shows the total volume of publications discussing the GBGI papers. The publication records obtained from the Web of Science portal (Clarivate) using the search query: Green Infrastructure (All Fields) OR Nature-based Solutions (All Fields) AND "Air Pollution" (Keyword Plus®) AND "Climate Change" (Keyword Plus®) AND "Temperature" (Keyword Plus®) AND "Flooding" (Keyword Plus®).

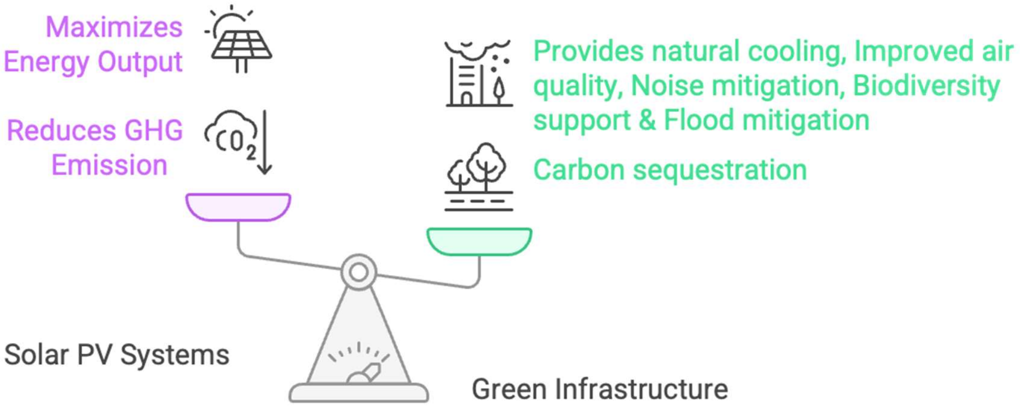

Figure S4. Balance model comparing solar PV Systems and GI. PV offers energy maximisation and GHG reduction, while GI provides natural cooling, improved air quality, noise mitigation, biodiversity support, and flood mitigation, in addition to carbon sequestration. GI delivers broader ecosystem services, though it may also compete with PV systems for available space.

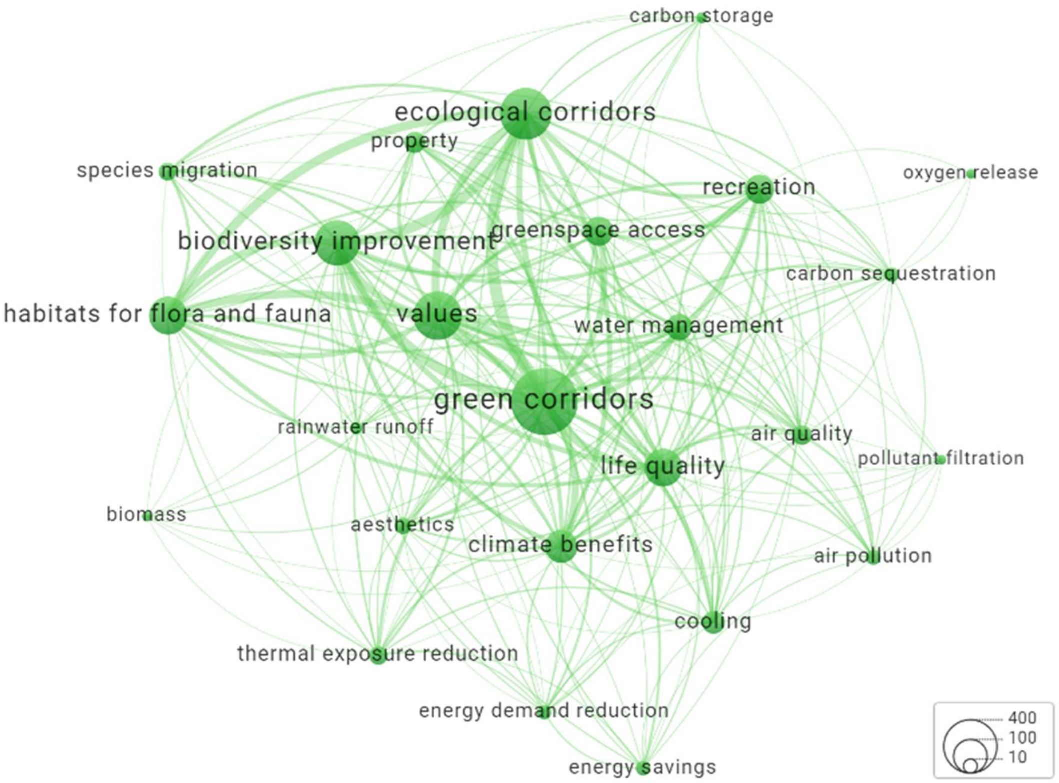

Figure S5. Results of a bibliometric analysis: thematic map obtained from 751 publications screened for the following keywords: climate benefits, thermal exposure reduction, cooling, biodiversity improvement, species migration, habitats for flora and fauna, greenspace access, energy savings, energy demand reduction, water management, rainwater runoff, air pollution, air quality, pollutant filtration, oxygen Release, biomass, carbon storage, carbon sequestration, recreation, aesthetics, life quality, property, values.

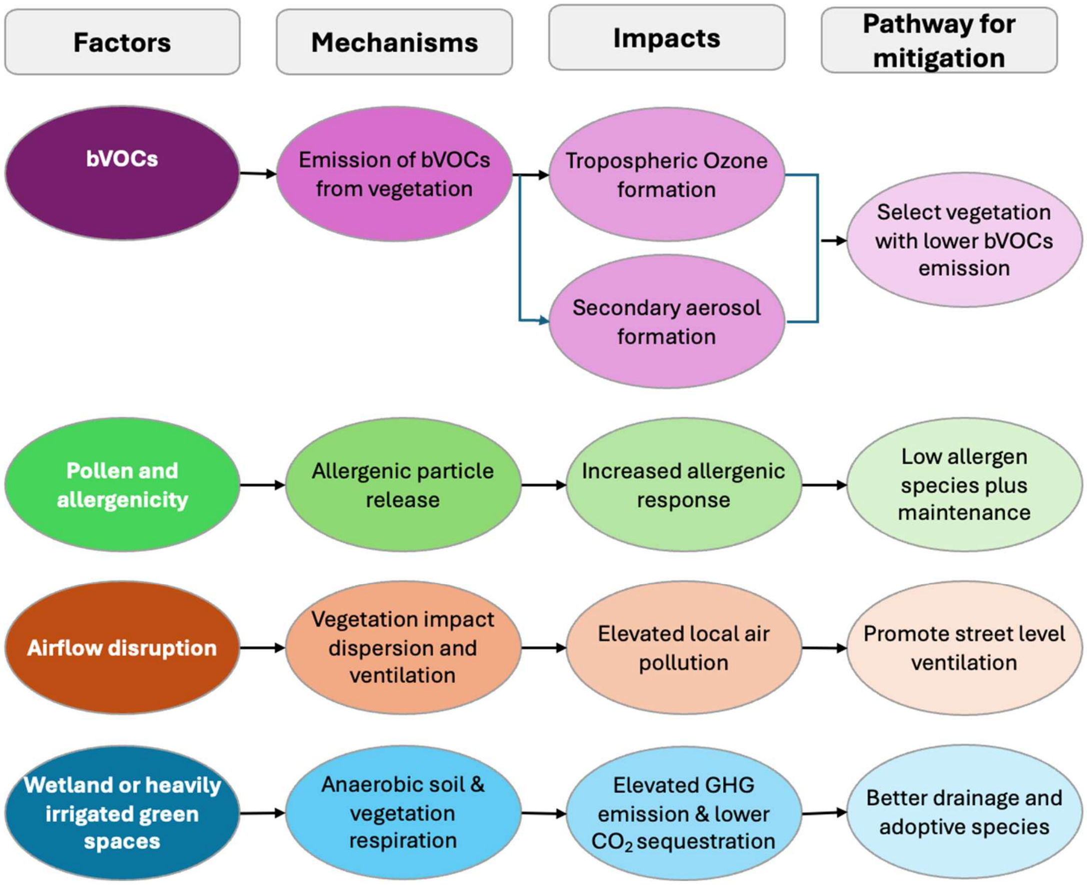

Figure S6. Conceptual diagram illustrating unintended consequences hindering the implementation of GBI. The image shows how factors like bVOC emissions, allergenic pollen, airflow disruption, and GHG release can lead to environmental and health impacts, along with strategies to mitigate each.

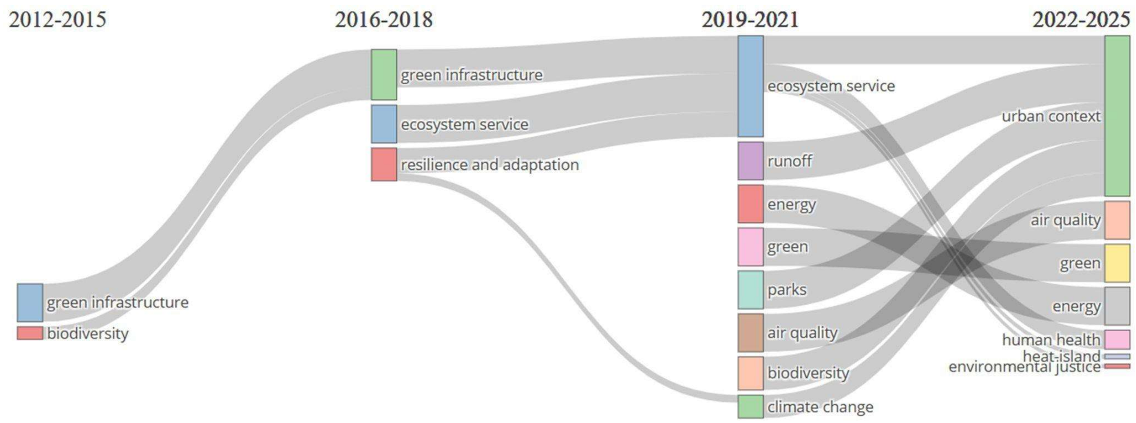

Figure S7. Thematic Evolution of GBI Research from 2012 to 2025. (Search string: "green infrastructure" OR "blue infrastructure" OR "green-blue infrastructure" OR "nature-based solutions" AND ("mitigate" OR "adapt" OR "address") AND ("environmental challenges" OR "environmental problems" OR "environmental issues" OR "urban resilience"). Database: Scopus and Web of Science).

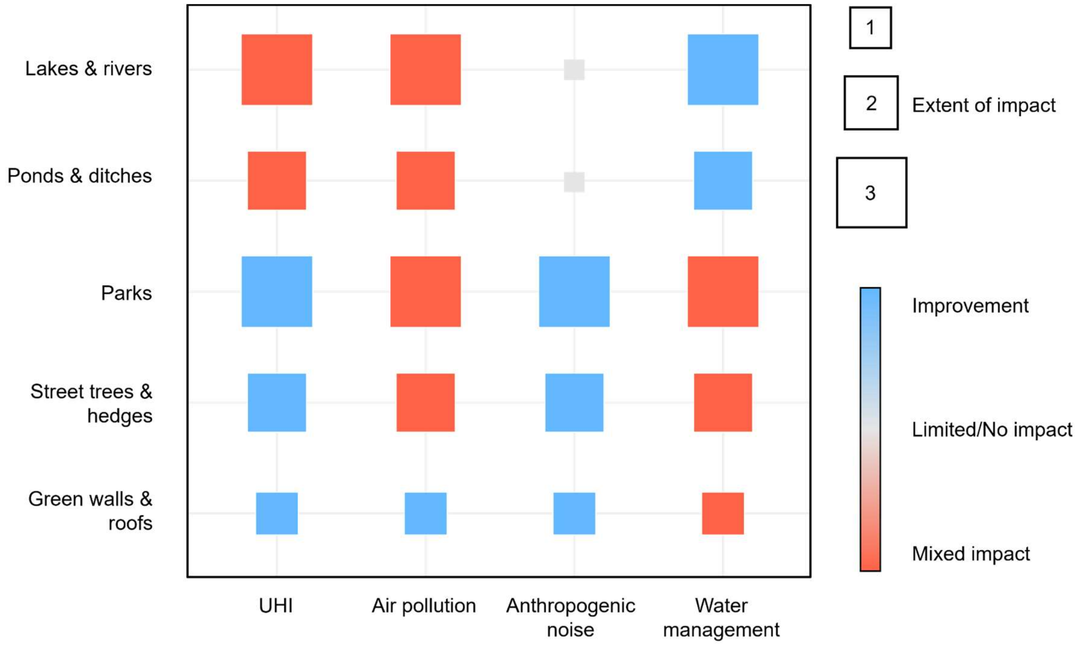

Figure S8. A visual matrix illustrating the diverse impacts of different GBI types on key urban challenges. Square colour and size indicate the type and relative magnitude of influence that each GBI type has on a given urban challenge, based on the authors' synthesis of literature. Detailed corresponding information can be found in Table S5.

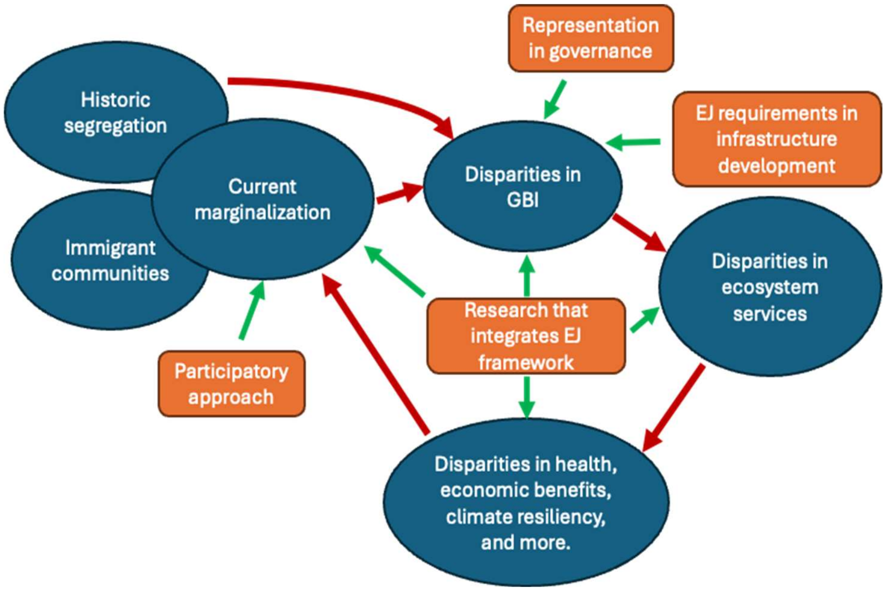

Figure S9. Framework linking socio-economic marginalisation with disparities in ecosystem services and well-being (blue circles and red arrows), with policy interventions points highlighted by orange box.

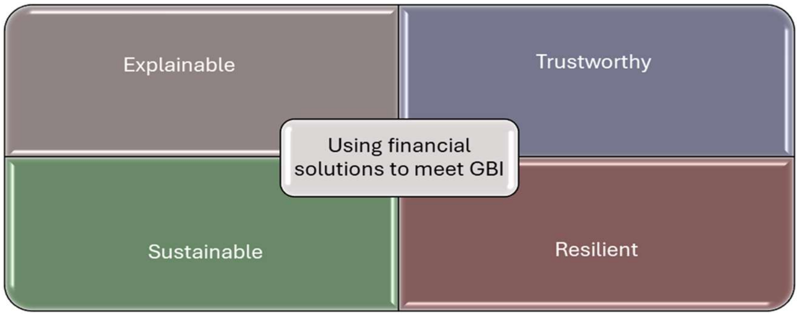

Figure S10. Building a strong financial ecosystem to support Green and Blue Infrastructure. The framework illustrates the four foundational pillars essential for mobilizing financial solutions that effectively support the implementation and scaling of GBI.

S1. Co-design process for barrier identification

The identification and selection of the barriers presented in this study were the result of a rigorous, multi-stage participatory process designed to leverage diverse expertise and build consensus. The three-step process involved: (1) Initial Identification via a large interdisciplinary RECLAIM Network Plus workshop, with over 60 participants, held in-person at the University of Surrey, Guildford, UK, on 30 January 2025 (RECLAIM 2025). (2) Independent Refinement by a multidisciplinary expert group in-person meeting of 20 participants at North Carolina State University (NCSU), Raleigh, NC, USA, on 26 March 2025. (3) Finalisation through an iteration involving all authors named in this manuscript to ensure a balanced, non-redundant list of 'overlooked' barriers.

The process began with a large, interdisciplinary RECLAIM Network Plus workshop (RECLAIM 2025), which generated an initial longlist of potential barriers through structured panel discussions and breakout sessions. This workshop included experts from councils, local government, policy makers, research funders, and collaborators from academic and non-academic sectors such as charity organisations and businesses. To refine this extensive long-list, a second, more focused workshop was convened with approximately 20 multidisciplinary experts at NCSU, the majority of whom had not been involved in the generation of the long-list, and provided a second independent expert opinion. This group - comprising engineers, social scientists, computer scientists, mathematicians, ecologists, urban planners, environmental policy, and climate scientists - collectively screened and prioritised the topics to agree upon a coherent, shorter list.

To ensure inclusivity and final consensus, this refined list was then circulated to the entire author team, including members who could not attend the previous workshops. This third stage was crucial for eliminating repetition, balancing the coverage of topics, identifying and matching their expertise, and verifying that each barrier met the core criterion of being 'overlooked' in current planning and policy.

This entire collaborative effort was co-ordinated by the lead author through one-to-one discussions, and a shared open-access working draft online manuscript outline. This platform allowed all contributors to openly comment, critique, and support suggestions in real-time, creating a transparent and iterative workflow that directly shaped the final selection and framing of the barriers presented herein.

S2. Overcoming siloisation in urban GBI studies

The bibliometric analysis was produced with 753 English language publications from the Scopus online database, using the keywords “Green Corridors” OR “Ecological Corridors”. Publications were then screened for the presence of the above predefined keywords. The screening involved searching across titles, abstracts, author keywords, and index keywords for specific terms associated with each of the predefined keywords. Variations in wording and conceptually equivalent expressions were also considered indicative of keyword presence and the resulting keyword list was then used to replace the original author keywords in the dataset. This standardised keyword dataset was subsequently imported into VOSviewer to generate a co-occurrence map of the relationships between “Green Corridors”, “Ecological Corridors” and the other thematic categories as shown in Figure S5.

A search within the results was conducted for keywords in the following eight ecosystem service categories: 1. climate benefits; thermal exposure reduction; cooling 2. biodiversity improvement; species migration; habitats for flora and fauna 3. greenspace access 4. energy savings; energy demand reduction 5. water management; rainwater runoff 6. air pollution; air quality; pollutant filtration; oxygen release 7. biomass; carbon storage; carbon sequestration 8. recreation; aesthetics; life quality; property; values. Keywords from four or more of the above categories appeared in 12 studies which were downloaded and examined. Seven studies were excluded, and the remaining five studies are presented in Table S2.

S3. Strategies to mitigate environmental challenges

To explore how scholarly research has addressed fragmented approaches to GBI implementation - particularly in relation to the mitigation, adaptation, and management of environmental challenges - a comprehensive literature search was conducted using the Scopus (n = 207) and Web of Science (n = 305) databases. The following search string was applied: ("green infrastructure" OR "blue infrastructure" OR "green-blue infrastructure" OR "nature-based solutions") AND ("mitigate" OR "adapt" OR "address") AND ("environmental challenges" OR "environmental problems" OR "environmental issues" OR "urban resilience"). After the removal of duplicate records, a total of 335 unique articles were kept for bibliometric and thematic analysis. Bibliometric analyses, including thematic mapping and thematic evolution, were performed using the Bibliometrix R package (Aria and Cuccurullo, 2017) by applying the Biblioshiny interface. Thematic maps were based on keyword co-occurrence networks, analysed through clustering algorithms and plotted according to their centrality and density. Thematic evolution was examined across four time slices corresponding to 3-year periods (2012–2015, 2016–2018, 2019–2021, and 2022–2025) to enable an analysis of the development and growth of research within this topic over time. Over the past decade, research on GBI has undergone a conceptual transformation (Figure S7). This visual representation maps how scholarly interest has progressed in response to environmental challenges.

Table S1. Deeper analysis of case studies, detailing GBGI type, challenges, trade-off, failures and references with corss-link to relevant paper sections for further context.

GBGI types used in case study	Challenges addressed/ faced	Trade-offs	Failure (partial or full)	Reference: author (year)	Link to manuscript section
Urban trees	Demonstrated carbon mitigation potential: urban trees offset ~17% of carbon emissions over 60 years	Long-term carbon benefits but limited integration with renewable generation options	Partial: effective over long timescales, but does not address immediate emission reduction needs	Grossi et al. (2023)	GBI conflicts with Net Zero
Urban trees	Trees reduce solar roof radiation by ~1% after 20 years of growth	Trade-off between tree canopy benefits vs. rooftop PV efficiency; some argue PV delivers greater carbon reduction than trees	Partial: tree benefits remain, but PV may outperform in emission reduction	Reitberger et al. (2025)	GBI conflicts with Net Zero
Green corridors	Multi-functionality (water storage/purification, local climate regulation, aesthetics) and biodiversity & energy flow (species connectivity, energy patterns via least-cost path and InVest)	Balancing ecological restoration and multifunctional ES delivery with infrastructure expansion and connectivity	Partial: improvements achieved in ES and connectivity, but gaps in ecological resilience and ecosystem functions remained	Wong et al., (2018); Tao et al., (2022)	Siloisation in urban GBI studies
Green corridors	Well-being & accessibility: studies highlighted recreation and life quality benefits using literature and online tools	Trade-off between limited quantitative ecological data and qualitative life-quality outcomes	Partial: broadened focus beyond biodiversity, but methods less robust	Larrea et al. (2024); Mancilla et al. (2024)	Siloisation in urban GBI studies

Green corridor	Biodiversity & energy flow: analysed species movement resistance and energy flow with InVest + least-cost path	Balancing urban development with ecological connectivity	Partial: useful for planning, but still siloed in selected themes	Isola et al. (2024)	Siloisation in urban GBI studies
Wetlands	Designed for nitrogen removal and water regulation but can increase CH_4 and CO_2 emissions	Wetlands designed to reduce nitrogen loads can have low carbon impacts and affect wildlife.	Partial: Poorly implemented wetland restoration programs can result in the loss or perceived loss of community use rights	Li et al. (2024); Yuan et al. (2025)	Unintended consequences
Urban trees	Noise reduction: increased tree crown volume and canopy linked to decreased noise levels	Focused on acoustic benefits, less attention to other ecosystem services	Partial: case-specific evidence, broader transferability uncertain	Zhao et al. (2021)	Conflict control among urban environmental challenges
Green roof	Seasonal impact of green roofs on outdoor fresh air intakes via HVAC filters around 14% reduction in ozone	Ozone removal benefit but higher microbial and VOC fluxes transfer from green roof to HVAC system	Partial: green roof has benefits for ozone removal but impact of PM ingress on HVAC filter loading not addressed	Ramasubramanian et al. (2021)	Urban ventilation
Green roof	Demonstrated ambient PM reductions - 31.8% after 1 year of growth	Enhances PM removal but the impact on a HVAC system fresh air intake is not clear	Partial: effective at PM removal on rooftop but limited to local scale	Kostadinović et al. (2023)	Urban ventilation
Schoolyard greening	Heatwave resilience: transforming schoolyards into urban cool islands, accessible to communities during extreme heat	Trade-off between limited space use and multifunctional adaptation benefits	Partial: success in pilot schools, broader city-wide impacts still scaling	Climate-ADAPT (2022)	Thermal resilience

Urban trees	Stress tolerance during severe drought: tree showed increased photosynthetic activity and growth	Focus on single species; broader applicability to other urban tree species uncertain	Partial: demonstrates strong resilience, but not generalisable across all GBI species	Locosselli et al. (2024)	Pathways to Resilient GBI via Plant Adaptation
Rooftop farming	Food-water-energy nexus: open-air roofs produced up to 7.44 kg vegetables/m² annually and reduced upstream energy & water footprints by a factor of 4.5	Trade-off between maximizing food production and rooftop space available for other GBGI or PV systems	Partial: strong potential, but scalability and long-term viability depend on local context	Yuan et al. (2025)	GBI trade-offs
Urban trees	Redlining and racial segregation created lasting disparities in canopy, impervious cover, air quality, heat, biodiversity, and health	Unequal GBGI distribution across racial groups	Partial: inequities persist post-redlining	Gibbons (2023); Benz & Burney (2021)	Environmental injustice and GBI decision making
Classical Chinese gardens	Demonstrates cultural-ecological integration; regulates microclimate	Strong cultural embedment but limited scalability to urban level	Partial: effective at garden scale, limited in urban upscaling	Xiong et al. (2020)	Cultural perspectives on GBI
Urban parks	Promoted stronger community attachment compared to fragmented/inaccessible spaces	Centralisation boosts social cohesion but risks unequal access for distant residents	Partial: effective for attached users, limited inclusivity across city	Zhu et al. (2017)	Social adoption hindering implementation of GBI
Small urban parks and greenways	Safety concerns over drug use and antisocial behaviour led to redesign	Improved surveillance may reduce natural character	Partial: redesign improved safety but altered original intent	Rigolon & Christensen (2019)	Safety and security barriers in GBI implementation
Urban meadows	Shift from ornamental lawns to multifunctional, climate-adaptive landscapes	Enhances biodiversity and resilience but challenges traditional aesthetic norms	Partial: ecological benefits achieved, but resistance from	Marshall et al. (2023)	Balancing climate adaptation and aesthetic goals in urban GBI

			conventional aesthetic expectations		
Green/blue bonds and debt-for-nature swaps	Difficulty securing finance; biodiversity funding gap persists	Credible certification ensures investor trust, but depends on global financial markets and risk pricing	Partial: successful in some countries (e.g., Seychelles, Belize, Ecuador) but large biodiversity finance gap persists	Flammer (2020, 2021); Schweinberger (2023)	Financial barriers to GBI implementation
Parks, urban vegetation, and wetlands	Quantified environmental value but excluded from formal accounting; often treated as liabilities	Developers profit from green proximity, but local councils lack revenue for upkeep; hybrid grey-green benefits undercounted	Partial: undervaluation limits financing options and institutional support	Matsler (2019); Kennedy et al. (2023)	Challenges in recognising GBI as assets
Green roofs	Faced high upfront construction costs, uncertain long-term return on investment, and maintenance challenges	Economically viable only with subsidies due to high construction and maintenance costs. Even with subsidies, many projects yield zero or negative net present values (NPV)	Partial failure in cases without sustained subsidies or incentive-based programs; risk of abandonment when costs outweigh benefits	Nordman et al. (2018)	Lack of comprehensive cost-benefit analysis (CBA)
Green roofs, walls, permeable surfaces,, pocket parks	Land scarcity and sprawl limit space; rising land values prioritise densification; peri-urban expansion fragments ecosystems	Space-efficient retrofits provide ES but need maintenance; peri-urban GBI often isolated	Partial: microscale effective but limited in scale and connectivity	Tache et al. (2024); Bressane et al. (2024)	Land scarcity and urban sprawl

Rooftop farms, permeable pavements, bio-retention, modular planters	Technical conflicts, poor multi-scale coordination, policy inertia, and weak maintenance planning	3D and multifunctional GBI enhance ES but face cost and governance trade-offs	Partial: pilots (e.g., Malmö) succeed but grey bias dominates	Wang et al. (2025); Kramer et al. (2024)	Urban design barriers
Parks, corridors, hybrid green-grey systems	Lack of clear policies; weak leadership, fragmented governance, competing grey priorities	Long-term benefits but low short-term political visibility; grey favoured	Partial: gradual shifts (e.g., Melbourne Grey-to-Green) but slow elsewhere	Abdallah et al. (2025)	Lack of clear GBI implementation policies
Green corridors, linear parks, stormwater-linked streetscapes	Poor GBI–walkability integration; car-centric planning, grey engineering, land costs	Walkability and ES gains need space reallocation and upfront cost	Partial: co-location common but true integration rare	Wedyan et al. (2025); Petersen et al. (2024)	Conflicts in promoting GBI and walkability

Table S2. Summary of key review papers on solar PV and green infrastructure integration (2010-2025). Literature addressing conflicts, synergies, impacts, and frameworks for balancing renewable energy with conservation of GI.

Author (year)	Key focus area of review	Main findings
Pandey et al. (2025a)	How agrivoltaic systems integrate agriculture and solar energy production to enhance land-use efficiency, profitability, and environmental sustainability.	Agrivoltaic systems enhance water efficiency (150-300%), improve land use (200%), and increase revenue (15x), despite higher installation costs (5-40%), offering competitive returns (payback <10 years) and revenue diversification, especially in arid regions.
Reitberger et al. (2025)	Reduction of solar radiation on the roof as a result of tree growth.	Tree growth over 20 years only reduced solar radiation on the roof by 1%.
Skandalos & Karamanis (2025)	An integrated approach to rooftop PV and green infrastructure under present and future European climate scenarios.	NbS solutions such as tree planting, green roofs, and green walls offer significant carbon sequestration benefits that complement those provided by solar PV installations. Additionally, tree shading contributes to annual reductions in cooling demand.
Asa'a et al. (2024)	Agrivoltaic systems that combine solar energy generation with crop cultivation for optimized land use.	Agrivoltaics increases land use efficiency, creates beneficial microclimates, and shows crop-specific shade responses with PV design optimization being critical for balancing energy and agricultural production.
Dunlap et al. (2024)	Impact of intensive solar development in California.	To maximise solar energy produced, intensive solar development can have negative impacts on nature and green spaces.
Oliveira et al. (2024)	Ecological impacts of floating photovoltaics (FPV) on aquatic organisms across different taxonomic groups, identifying research trends, knowledge gaps, and potential effects on aquatic biodiversity.	FPV generally reduces algal growth and primary production, with effects increasing at higher coverage. Impacts on aquatic organisms are complex, including altered community structures and physiological changes, but research remains limited despite rapid industry expansion.
Essak & Ghosh (2023)	Floating photovoltaics (FPV) as a growing renewable energy technology, analysing its benefits over ground-mounted systems, ecological impacts, applications across different water bodies, and global implementation potential.	FPV offers 3-12.5% higher efficiency than ground-mounted systems, reduces water evaporation, addresses land scarcity, and shows strong growth potential. However, impacts on water quality and ecology remain understudied and require further research.
Grossi et al. (2023)	Potential of trees to absorb carbon emissions in Montreal.	Trees can reduce carbon emissions of a building with BIPV by 17% even before accounting for the on-site electricity generation.

Liu et al. (2023)	Integration of solar energy technologies and NBS to achieve carbon neutrality.	Incorporating NBS is important to maximise resilient benefits. Solar energy technologies alone are not enough to achieve carbon neutrality.
Wang et al. (2023a)	Integration of photovoltaic systems with green roofs and facades to create sustainable, energy-efficient buildings with multiple environmental benefits.	The combination of building-integrated PV with GI provides mutual benefits, with vegetation cooling photovoltaic panels to improve efficiency (1-3%) while optimizing space usage, though performance depends on installation distance, plant species, and microclimate.
Zhang et al. (2023a)	Benefits and potential environmental impacts of implementing PV technologies.	Power generation with PV reduces pollution although also has potential negative impacts, e.g. toxic chemicals in PV materials production and can negatively impact vulnerable lands and ecological functions.
Kruitwagen et al. (2021)	A comprehensive global database of photovoltaic installations across commercial, industrial, and utility scales.	The data indicate that the majority of solar PV facilities are located on croplands, arid regions, and grasslands.
Dhar et al. (2020)	Environmental challenges linked to solar and wind energy development, along with mitigation strategies and potential reclamation practices.	Wind and solar energy installations can adversely affect land, native vegetation, and biodiversity; however, these impacts can be significantly reduced by incorporating reclamation into the planning phase and applying best management practices.
Zhong & Tong (2020)	Factors that affect the performance of PV systems and proposed a new spatial optimization method to maximise PV performance.	Different PV panel orientations and alignment scenarios can be optimised to maximise energy production.
Hernandez et al. (2014)	USSE development has direct and indirect effects on biodiversity, land-use change, soils, water resources, and human health.	Several USSE characteristics and development strategies have low environmental impacts and therefore opportunities exist to minimize impacts, including having proper inventory and monetizing the real value of natural capital and ES of land.
Anders et al. (2010)	Laws in the United States at the state level on access to direct sunlight.	Removing vegetation is allowed by law in the US if the vegetation comprises the performance of rooftop solar PV.

Table S3. Summary of papers examining four or more ecosystem service categories associated with Urban Green Corridors.

Author (Year)	Aim	Methods	Conclusion	ES Studied
Wong et al. (2018)	Examine the impact of a GC in multiple ES. Beijing, China.	Ecological Production Functions methodology assessed the impact of the GC before and after its construction.	During landscape design ecosystem functions need to be considered in order to avoid shortfalls. Multi-functionality can be obtained by pairing green and built infrastructure.	Regulation of the local climate, water storage and purification, aesthetics.
Tao et al. (2022)	Establish an evaluation framework aiming to ecological restoration and protection at multiple scales . Nanjing, China.	ESs quantified by InVEST. An Ordered Weighted Averaging model simulated areas that were selected for their ecological conservation importance. Circuit theory identified ecological sources, corridors, barriers and pinch points at the city and main urban scales.	Many GC and ecological barriers identified at the small and urban scales, indicating that studying large-scale ecological networks at city scale only cannot fully reflect the challenges involved.	Habitat improvement carbon storage, nitrogen retention, and water conservation.
Larrea et al. (2024); Mancilla et al. (2024)	Design of a GC that aims to ecosystem restoration, improvement of green spaces, connectivity and revaluing of the urban landscape. Cusco, Peru.	Non-experimental approach: literature analysis, landscape analysis with climate consultant, planimetry, 3D survey.	A case study of botanical gardens including biofilters, terraces and guinea pigs farms contributes to water quality, energy generation and habitat for flora and fauna.	Energy generation, recreational areas, water purification, flora and fauna habitat.
Isola et al. (2024)	A methodology to approach UGI, as a network of areas connected by urban GCs. Cagliari, Italy.	a. Spatial taxonomy of GI with Ess (InVEST model); b. GCs identified with Least-Cost Path analysis c. Linear Regression between the two.	Results allow the quantitative profile of GI that contributes as a structural reference for urban policies.	Recreation opportunities; water retainment capacity; carbon sequestration and storage; habitat quality; cooling capacity.

Table S4. Summary of studies outlining unintended consequences of GBI implementation. The table provides an overview of selected studies that examine unintended consequences associated with the implementation of GBI. Each entry includes the reference, a brief description of the study and the specific unintended consequence, GHG, bVOC, pollen emission, allergenicity and airflow disruption.

**Author (Year)**	**Description**	**Unintended consequence addressed**
Nowak and Crane (2002)	The article quantifies carbon storage but warns that poorly managed vegetation may also emit CO_2, reducing net sequestration gains.	GHG
Pinto et al. (2010)	Review of O_3 effects on bVOC emissions, highlighting positive feedback that enhance SOA formation.	bVOC
Cariñanos and Casares-Porcel (2011)	This study addresses how specific urban planting strategies can unintentionally increase allergy incidence due to the use of highly allergenic species.	Pollen and allergenicity
Pugh et al. (2012)	This study demonstrates that certain green GI arrangements can trap air pollutants instead of dispersing them, worsening local air quality.	Airflow disruption
Calfapietra et al. (2013)	Examines how biogenic VOC emissions from urban trees contribute to ozone formation, emphasizing differences among species and associated feedback mechanisms.	bVOC
Nowak et al. (2013)	Assessed the life-cycle carbon impact of urban trees.	GHG
Janhäll (2015)	The review discusses how tall or improperly placed vegetation can reduce air dilution and lead to pollutant buildup in urban microenvironments.	Airflow disruption
Chen et al. (2016)	This study evaluated different species to improve air quality at an urban level.	Airflow disruption
Livesley et al. (2016)	This study shows that urban green areas with high vegetation density may enhance nocturnal CO_2 accumulation, especially in areas with limited airflow.	GHG
Säumel et al. (2016)	This study shows the maintenance activities such as pruning, irrigation, and waste management can generate CO_2 emissions that partially offset sequestration benefits.	GHG
Velasco et al. (2016)	The study found that the capacity of urban greenery in (sub)tropical areas to sequester CO_2 is relatively constrained. In Mexico City it acted as a small sink, while in Singapore it was a source.	GHG
Abhijith et al. (2017)	This review highlights how certain GI designs can unintentionally increase pollutant concentrations by disrupting airflow in street canyons.	Airflow disruption
Sierra-Heredia et al. (2018)	The study explores the distribution and health risks of Canadian aeroallergens, while assessing how climate dynamics, air pollution, and urban vegetation changes contribute.	Pollen and allergenicity
Eisenman et al. (2019)	This study shows that urban tree planting programs often fail to adequately consider the allergenicity of	Pollen and allergenicity

	selected species.	
Sawers (2019)	Policy-oriented review highlighting the importance of species selection to minimize bVOC contributions to air pollution.	bVOC
Barwise and Kumar (2020)	This review emphasizes how poor selection of plant species for vegetative barriers may fail to reduce or even exacerbate air pollution levels.	Airflow disruption, pollen and allergenicity, GHG, bVOC
Salvador et al. (2020)	Field research in a subtropical forest reveals that bVOC oxidation products play a major role in ozone and secondary organic aerosol (SOA) formation.	bVOC
Gu et al. (2021)	Urban greening within LA may raise bVOCs, offsetting air quality gains. Using low-VOC plants is advised.	bVOC
Sousa-Silva et al. (2021)	This study finds that an insufficient understanding of tree pollen allergenicity can lead to planting choices that unintentionally increase health risks.	Pollen and allergenicity
Browning et al. (2022)	This study shows the positive health outcomes from GBGI exposure but noted that allergenic pollen release represents a significant challenge that can diminish these benefits for portions of the population.	Pollen and allergenicity
Cao et al. (2022)	Long-term modelling in China indicating biomass-driven increases in bVOC emissions intensify summertime O_3 and SOA.	bVOC
Fellini et al. (2022)	Wind tunnel tests show that dense tree canopies may reduce air ventilation, unintentionally trapping pollutants at pedestrian level.	Airflow disruption
Zhang and Steiner, (2022)	The study provides evidence that climate change will alter both the timing and volume of airborne pollen emissions by century's end.	GHG, Pollen and allergenicity
Stawoska et al. (2023)	The study found that birch pollen from polluted areas had higher Bet v1 allergen levels and altered protein structures, potentially increasing allergenicity, despite minimal effects on plant physiology.	Pollen and allergenicity
Li et al. (2024a)	This bibliometric review reveals that constructed wetlands, if poorly designed, may act as unintended sources of methane and carbon dioxide.	GHG
Maison et al. (2024)	Street-level models in Paris show higher pollution and exposure than regional models, especially for NO_2 and BC.	Airflow disruption
Yuan et al. (2025)	This review shows how plants in wetlands influence greenhouse gas emissions, depending on species, diversity, and harvesting.	GHG

Table S5. Summary of key papers addressing the synergistic relationship between GBI and urban ventilation from 2010-2025. The table outlines the effect GBI and urban ventilation (UV) has on urban heat island (UHI), air quality (AQ), building energy performance (BEP), humidity (H), natural ventilation (NV), HVAC systems and thermal comfort (TC).

**Author (Year)**	**Description**	**UV**	**GI**	**AQ**	**HVAC**	**UHI**	**GBI**	**NV**	**H**	**BEP**	**TC**
Amorim et al. (2013)	Evaluation of the impact GI has on pollution dispersal emitted by road traffic in an urban setting using numerical models.	√	√	√							
Asere and Blumberga (2020)	Analysis of how indoor air quality, building efficiency, and HVAC energy use are interrelated.				√					√	
Asim et al. (2022)	Review of the parameters that inform the sustainable performance of HVAC systems.			√						√	√
Badach et al. (2022)	GIS and numerical modeling used to assess how structural changes influence ventilation pathways.	√		√			√				
Beier et al. (2022)	Microclimate modelling of the influence GBI has on UHI.					√	√				
Belda et al. (2021)	Numerical model examining real world urban case study, and the influence GI has upon particulate concentrations, surface and air temperatures.	√	√	√		√					
Buccolieri et al. (2010)	Modelling urban ventilation to test the ‘breathability’ of cities.	√									
Considine et al. (2022)	Development of rooftop air filtration technology to reduce particulate load under diverse climates.	√		√	√						
Considine et al. (2023)	Street canyon model analysing the concentration rate of vehicular particulate emissions entering the outdoor fresh air intake of a HVAC system located at rooftop level.	√									
Irga et al. (2024)	Integrating green walls into tunnel ventilation stacks to reduce the potential increase in ambient particulate emissions.	√	√	√							
Kostadinović et al. (2023)	On-site study of green roof performance in passive air pollutant control.	√									
Liu et al. (2021)	Review of green roof effects on hydrology, thermal regulation, and air quality.		√	√		√					

Morgan et al. (2017)	Deployment of passive air filtration to reduce ambient particulate matter entering ventilation systems.		√		√						
Palusci & Cecere, (2022)	Developed a workflow and framework for improving urban ventilation in existing urban areas to mitigate UHI and air pollution.	√									
Park et al. (2024)	Analysis of GI as a mitigation measure for UHI in a humid region.	√	√			√			√		
Ramasubramanian et al. (2019)	Field study investigating the reduction of O_3 present in the outdoor fresh air ventilation intake at the rooftop level where a green roof is present.	√				√					
Ramasubramanian et al. (2021)	Field study comparing the concentrations of bVOC, microbial composition and O_3 present on the filters of a HVAC system for a green and white roof.		√	√	√						
Salmond et al. (2013)	Field study of the influence GI has on the vertical and horizontal gradients of pollutants in a street canyon.			√	√						
Shi et al. (2022)	Investigated how ventilation corridors and GBI influence UHI across diverse urban climates.	√				√	√				
Tang et al. (2023)	Developed theoretical references for urban and GBI planning based on wind-driven ventilation and pollutant dispersion in street canyons.	√									
Vasudevan et al. (2024)	Adjustable wind deflectors used to promote pollution removal from urban street canyons.	√		√							
Wahba et al. (2018)	Retrofitting GI to a building's roof and walls to enhance the HVAC system's energy performance.		√	√	√					√	
Xiong et al. (2024)	Proposed a natural ventilation model for energy-efficient buildings, integrating multiple environmental variables to assess thermal comfort and indoor air quality.			√				√		√	
Zhang et al. (2020)	GHG emissions associated with air condition systems at a city scale.	√		√	√					√	
Zhang et al. (2023b)	Parametric investigation into how GI planted upstream of a building impacts the natural cross ventilation rates.				√			√			
Zhao et al. (2024)	Machine learning model to predict local temperatures and wind velocities with the addition of new buildings and HVAC heat rejection in high density urban areas.	√									

Table S6. Potential conflicts to achieving synergistic control of urban challenges through GBI.

GBI types	Potential disservices	Case studies and meta-analysis reviews
Large BI (e.g., lakes and rivers)	Nighttime UHI intensification (e.g., due to increased humidity and heat release from water masses)	(Chen et al., 2024; Fricke et al., 2024; Hu and Li, 2020; Theeuwes et al., 2013; Triyuly et al., 2021)
	Greenhouse gas emissions from sediment accumulation (e.g., N_2O and CH_4 emissions)	(Woszczyk and Schubert, 2021; Yin et al., 2025; Zhang et al., 2021)
Small BI (e.g., ponds and ditches)	Nighttime UHI intensification (e.g., due to increased humidity and heat release from water sources)	(Yao et al., 2023; Fricke et al., 2024)
	Greenhouse gas emissions from sediment accumulation (e.g., CH_4 and N_2O emissions)	(Bauduin et al., 2024; Holgerson and Raymond, 2016; Rosentreter et al., 2021; van Bergen et al., 2019)
Large GI (e.g., Parks)	Biogenic emissions (bVOCs and pollen from vegetation, contributing to elevated O_3 and PM concentrations)	(Ahn et al., 2022; Bao et al., 2023; Churkina et al., 2017; Ma et al., 2022; Maison et al., 2024)
Street trees and hedges	Obstruction of pollutant dispersion (e.g., dense tree canopies reducing street-level ventilation in urban canyons)	(Buccolieri et al., 2018; Li et al., 2023; Salmond et al., 2013; Gromke and Ruck, 2007; Guo et al., 2023; Řezníček et al., 2025)
All types of GI	Over-Irrigation Leading to Water Scarcity (e.g., excessive water consumption by turfgrass during drought seasons)	(Cheung et al., 2022; Litvak et al., 2017; Pincetl et al., 2019; Saher and Ott, 2025; Van Mechelen et al., 2015; Wilfong et al., 2025)

Table S7. Overview of data collection methods (measurements, questionnaires, and simulations) and objectives pursued by GBI implementation (cooling/heating effect assessment and thermal comfort evaluation).

Author (Year)	Data collection method					Objective	
	Measurements			Subjective questionnaires	Simulation	Cooling/ heating effect	Thermal comfort
	Environmental parameters	Physiological parameters	Remote sensing				
Dashti et al. (2024)	√			√		√	√
Fei et al. (2023a)	√			√		√	√
Fei et al. (2024)	√				√	√	
Li et al. (2020)	√				√	√	√
Pritipadmaja et al. (2023)			√			√	
Manavvi and Milosevic (2025)	√					√	√
Jiang et al. (2020)					√	√	
Tan et al. (2021)			√			√	
Fei et al. (2022)	√			√	√	√	√
Ming et al. (2025)	√				√	√	
Cao et al. (2022)	√				√	√	
Behzad & Guilandoust, (2024)					√	√	√
Islam et al. (2024)			√			√	
Yang et al. (2020)			√			√	
Fei et al. (2023b)	√			√		√	√
Sun et al. (2020)			√			√	
Sun et al. (2021)					√	√	√
Wang et al. (2024)	√			√		√	√
Huang et al. (2024b)			√			√	
Balany et al. (2022)			√			√	
Lehnert et al. (2021)	√				√	√	√
Huang et al. (2024a)	√	√		√		√	√

Table S8. Summary of vegetation responses to climate change in cities. The table presents an overview of four main responses of plants to stress and how they take place in cities according to recent case-studies and reviews.

Responses to climate change	Reported pathways	Author (Year)
Phenotypic plasticity	● Phenotypic plasticity confers resilience to plant species growing in cities.<br>● Possible plasticity to enhance the knowledge of tree sensitivity and adaptive capacity to climate change.<br>● Leaf-level characteristics exhibited higher variability across different species and urban locations.<br>● Heat and drought tolerant species adjust assimilation rate to maintain growth rate.<br>● Air pollution modulates morphological and physiological traits, affecting photosynthesis differently depending on the plant species.<br>● Artificial light at night negatively impacts the photosynthetic parameters and the pigment content.	Das et al. (2024); Esperon-Rodriguez et al. (2020); Géron et al. (2024); Hanley et al. (2021); Locosselli et al. (2024); Martínez-Villa et al. (2024); Matsumoto et al. (2022); Papadopoulou et al. (2023); Sotillo et al. (2024); Wang et al. (2023); Wei et al. (2023)
Adaptation	● Urban environments may impose selective pressures that drive adaptation in short-lived plant species.<br>● Phenotypic plasticity may enhance the adaptive potential of specific plant type in cities.<br>● Genetic differentiation among urban and rural populations of the same species indicates adaptive responses to urban selective pressures.<br>● Phenotypic plasticity can increase the adaptive capacity of plant species in urban settings.	de Barros Ruas et al. (2022); Fukano et al. (2020); Fukano et al. (2023a); Fukano et al. (2023b), Lambert et al. (2021); Silva Luz et al. (2024); Sotillo et al. (2024); Woudstra et al. (2024)
Assisted migration	● Changes in environmental conditions due to urbanization processes support the use of natural features from nearby ecosystems to enhance the resilience of GBI in urban areas.	Miyahara et al. (2022)
Extinction	● Environmental changes due to urbanization processes can make it difficult to native species survive and reproduce, leading to local extinctions.<br>● GBI management, eutrophication, aridity, and landscape fragmentation are key local drivers of extinction for species with low phenotypic plasticity.	Hahs et al. (2009); Sotillo et al. (2024)

122 **Table S9.** Summary of key environmental injustice and GBI decision making challenges and research findings North and South Global countries.
123 The table provides an overview of selected studies that focus on green gentrification and racial inequalities in the implementation of GBI

Place studied	Key focus	What was covered	Author (Year)
Australia	Relationship between the development of urban green areas, the price increase, and the formation of gentrification.	Housing mobility and gentrification dynamics.	Caprioli et al. (2023)
Brazil–Curitiba and São Paulo	Remote sensing is used to examine the temperature variation, identifying heat islands.	Green infrastructures and neighbourhood income level.	Ribeiro et al. (2023)
China	Employs an array of social-environmental benefits to evaluate GI’s contributions to human well-being.	Mitigation of the UHI effect, recreational functions, enhanced landscape connectivity, and efficient stormwater management. Well-being and socio-economic indexes.	Xiong et al. (2024)
Colombia	Colombian GBI implementation experience	Recognize the significant influence of grassroots activism and civil society in defending urban commons, and recommend a critical review of green-blue infrastructure and river rehabilitation projects in Latin America.	Pradilla & Hack (2024)
Europe (3 cities) and the US (4 cities)	Explores the role that green gentrification plays in exacerbating racial tensions within historically marginalized urban communities, benefiting via enhancements like parks, gardens, waterfront restoration, and urban greenways.	Examines the complex relationship between historical environmental and racial injustices and current racial green inequities produced by the green city agenda.	Lewartowska (2024)
Italy–Rome and Poland–Gdansk	NbS can enhance air quality and improve societal well-being, but it is often not implemented equally, resulting in environmental injustice patterns.	The study supports concerns for fair NbS distribution, pointing to the potential exacerbation of socioeconomic divides, which heighten the exposure of disadvantaged communities to climate impacts.	Azadgar et al. (2025)
Portugal–Porto	Explores green infrastructure and its effects on urban heat island in Porto, Portugal, by monitoring the distribution of air spatial temperature and humidity.	Evaluation of vulnerability to heat-related health risks using heat risk index, integrating land surface temperature, land cover, and demographic data through remote sensing.	Lopes et al. (2025)
US	Conducted coding and analysis of 122 GBI formal	Intersectional urban challenges of social injustice and inequity,	Grabowski et al.

	plans from 20 US cities to explore how equity and justice are addressed within the areas of visions, processes, and distributions.	climate change, aging and expensive infrastructure, and socio-economic change.	(2023)
US–Boston	The study of green climate-resilient infrastructure–driven planning highlights a nuanced experience of gentrification and displacement affecting community belonging.	Underrecognized placemaking practices and alternative approaches to tackling socio-climate vulnerability both shape the dynamics of climate justice and injustice.	Shokry et al. (2025)
US–New York	Climate-driven hazards considering disproportionately affect low-income communities and communities of colour.	Co-production between government stakeholders and researchers to evaluate future situations over NYC for 2100 in response to climate hazards.	Dutta et al. (2025)

Table S10. Summary of key cultural challenges and research insights in GBI. This table presents a synthesis of cultural challenges identified in the planning, implementation, and adoption of GBI, drawn from diverse case studies and literature reviews. It highlights key findings from the selected studies along with actionable recommendations.

Cultural challenge	Case study/review	Key findings	Recommendations	Author (Year)
Under-recognition of cultural benefits	Review of 14,344 papers on CES using CiteSpace	CES underrepresented vs. ecological functions; limited focus on heritage, identity, education	Integrate CES via clear frameworks and wider spatial scales	Li et al. (2024b)
	Review of 153 interdisciplinary studies	Biophysical goals dominate; cultural identity and heritage overlooked	Use interdisciplinary and Indigenous-informed planning	McNabb et al. (2024a)
	Gorky Park, Russia	CES present but heritage and education uses underrepresented	Add cultural programming, educational and heritage-sensitive elements	Dushkova et al. (2025)
	Review (U.S, China, Canada)	Non-monetary values like place and learning are often missed	Incorporate CES such as sense of place and education into GBI	Elmqvist et al. (2015)
Limited community and cultural engagement	Semarang, Indonesia	Top-down BGI ignores local culture; weak awareness and uptake	Apply inclusive, bottom-up planning	Drosou et al. (2019)
	Wigram Basin, New Zealand	Weak perception of socio-cultural value due to limited engagement.	Include cultural experts, use co-design, plan explicitly for cultural value.	McNabb et al. (2024b)
	Helsinki, Finland	Technocratic planning sparked opposition due to overlooked values	Use early value mapping to include local cultural dimensions	Kati & Jari (2016)
	Narrative review (U.S. GI practices)	Lack of genuine participation excludes underrepresented groups and reinforces power imbalances	Start with participatory planning to empower communities and reflect local cultural values	Lovell & Taylor (2013)
	Conceptual review and framework proposal	Existing BGI frameworks overlook community values, practices, and engagement	Create tailored social resilience frameworks with place-based values and co-designed indicators	Campbell et al. (2024)
Lack of	Tumbes Basin, Peru	Indigenous agro-ecological GBI ignored in	Decolonize GI via Indigenous systems and	Tomateo (2021)

integration of Indigenous knowledge		formal planning	flexible typologies	
Cultural heritage and traditional landscapes	Jiangnan Furniture Museum, China	Optimized courtyard design improves microclimate and reduces seasonal energy loads	Combine vernacular spatial logic with passive strategies for cultural and environmental performance	Xu et al. (2018)
	Suzhou gardens (China)	Traditional gardens regulate climate and preserve identity	Apply water–vegetation–architecture logic in modern parks	Jiadai & Ibrahim, (2025)
	Antwerp, Belgium	Vertical greening (VG) applied to protected buildings raises concerns over material damage and lack of guidance	Provide context-specific strategies and heritage- compatible VG guidelines	Kale et al. (2023)
	Roman Theatre of Piane di Falerone, Italy	Removing vegetation solely based on preservation risks can worsen thermal comfort and reduce site usability	Balance vegetation removal with thermal comfort using tools like the Vegetation Hazard Impact Index (VHI) and microclimate modelling	Fabbri et al. (2025)
	Malaysian heritage cities (e.g., Sekeping, Penang & Kuala Lumpur)	Green reuse supports identity and performance, but GBI lacks heritage-specific criteria	Create a "Green Adaptive Reuse Index" linking GBI with heritage conservation	Alauddin et al. (2022)
'Green gentrification' and cultural displacement	Washington, DC	Long-term residents see GI as tied to displacement	Early community engagement, listening sessions, trust-building	Gearin et al. (2023)
	Six-city comparison (Barcelona, Halle, Lodz, New York, Oslo, Stockholm)	Overlooking justice leads to displacement, especially among marginalized groups	Apply EJ filters and participatory processes to address institutional and perceptual barriers	Kronenberg et al. (2021)
Culturally specific landscape preferences	Taj Mahal (India) & Ryoan-ji Garden (Japan)	Religious beliefs shape distinct spatial aesthetics rooted in Islamic and Zen traditions	GBI design should respect cultural interpretations of nature and avoid universalized models	Minnema (2019)
	Berlin, Germany	Green space meets quantity but not immigrant/elderly needs	Design based on culturally diverse usage and programming	Kabisch and Haase (2014)

Table S11. Summary of security barriers and solutions in GBI implementation across scales.

Scale	Safety/Security Concern	Description	Mitigation Strategy	Author (Year)
Street-level	Surveillance Blind Spots	Vegetated areas obstruct visibility, enabling illicit activities	Design with sightlines, low vegetation, smart lighting and cameras	Marzbali et al. (2012); He et al. (2025)
Household-level	Entry Point Obstruction	Vegetated fences/hedges obscure view of entry points	Transparent fencing, motion lighting, neighbourhood watch	Piroozfar et al. (2019); Marzbali et al. (2012)
Pocket Parks	Unregulated Gathering Spaces	Small parks may attract loitering or anti-social behaviour	Open layouts, visibility from homes, community programming	Kabisch et al. (2015); Nordh et al. (2009)
Informal Areas	Crime-Associated Stigma	GBI avoided for fear it may harbour crime in underserved areas	Participatory design, community policing	Pauleit et al. (2019); Kondo et al. (2016)
Transit Greens	Safety during Low-Use Periods	Isolated greenways/transit paths increase night-time vulnerability	Active lighting, regular patrols, clear escape routes	Bennetts et al. (2017)
All Scales	Over-Securitization	Excessive monitoring limits accessibility and inclusion	Balanced governance, inclusive and transparent planning	Wolch et al. (2014)

Table S12. Design trade-offs and integration strategies between climate adaptation and aesthetics in GBI.

Design Element	Climate Adaptation Function	Aesthetic Preference	Design Challenge	Creative Integration Strategy	Author (Year)
**Vegetation Type**	Native and drought-tolerant species enhance resilience and biodiversity	Ornamental and manicured plants are often preferred visually	Native species are perceived as messy or unmanaged	Combine native plants with structured layouts, signage, and seasonal color	Gillner et al. (2015); Lindemann-Matthies and Brieger, (2016); Livesley et al. (2016); Sjöman et al. (2012); Talal et al. (2021)
**Spatial Structure**	Heterogeneous layered landscapes buffer flood and reduce heat	Open, symmetrical spaces perceived as tidy and safe	Complex or irregular layout may be viewed as chaotic or unsafe	Frame ecological complexity with structured elements like paths or borders	Gillner et al. (2015); Ignatieva et al. (2017); Livesley et al. (2016)
**Water Feature**	Bioswales, retention ponds, and wetlands absorb runoff and improve water quality	Decorative fountains and still water bodies are preferred	Functional water features may appear unattractive or muddy	Integrate stormwater infrastructure with artistic and accessible design	Li and Nassauer, (2020); Meerow & Newell, (2017)
**Maintenance Approach**	Low-input systems mimic natural processes and reduce costs	High-maintenance landscapes signal care and order	Reduced upkeep can lead to perceptions of neglect	Promote "messy but cared for" aesthetic with signs and community involvement	Haase et al. (2014); Ignatieva et al. (2017); Li and Nassauer, (2020)
**Public Perception**	Resilient designing enhances long-term ecological health and equity	Residents often favour short-term beauty or tradition	Lack of awareness can reduce support for adaptive landscapes	Engage communities in co-designing, educating, and storytelling	Frantzeskaki et al. (2019); Hoyle et al. (2017); Raymond et al. (2017)
**Planning and Policy**	Integrated planning aligns spatial design with climate goals	Beautifying and greening goals are often fragmented	Ecological functions remain underfunded or undervalued	Support cross-sector collaboration and multifunctional policy tools	Kabisch et al. (2016); Langemeyer and Baró, (2021)

Table S13. Mapping of the twelve recommendations in Section 8 to the 21 identified barriers across environmental, social, economic, and governance domains. Each recommendation is linked to the specific barrier(s) and manuscript section(s) it addresses, enhancing clarity and strengthening the connection between challenges and proposed solutions.

Recommendation (as in manuscript)	Barrier(s) addressed	Section(s)
1. Strengthen interdisciplinary collaboration	Siloisation; Conflict control among urban challenges	3.2; 3.4
2. Prioritise science-driven species selection and adaptive design	Unintended consequences; Plant adaptation challenges; Biodiversity undervaluation	3.3; 3.7
3. Harness microclimate modelling and AI for ventilation-sensitive design	Urban ventilation; Trade-offs in microclimate and air quality	3.5; 3.8
4. Integrate thermal adaptation and vulnerability information	Thermal resilience; Environmental justice	3.6; 4.1
5. Integrate social dimensions into research frameworks	Cultural disconnection; Adoption challenges; Aesthetic controversies	4.2; 4.3; 4.5
6. Promote cross-disciplinary collaboration with communities	Social adoption; Safety concerns	4.3; 4.4
7. Develop context-sensitive policies	Safety concerns; Aesthetic controversies; Land and& space constraints	4.4; 4.5; 6.1
8. Strengthen financial ecosystem with ESG metrics	Economic undervaluation; Cost–benefit limitations	5.1; 5.2
9. Scale innovative financing mechanisms	Investment barriers; Regulatory gaps	5.3; 6.4
10. Align financial regulation and incentives	Integration challenges with other urban systems; Economic undervaluation; Investment barriers	5.1; 5.2; 5.3
11. Prioritise micro-scale, 3D GBI	Land and& space constraints; Trade-offs in land use	6.1
12. Embed GBI in urban planning through adaptive governance	Policy fragmentation; Integration challenges; Regulatory gaps	6.2; 6.3; 6.4

References

Abdallah A. K., Ismail L. S. and Alkaabi A. M. (2025). Green Careers: Educating for the Future of Sustainability. In Legal Frameworks and Educational Strategies for Sustainable Development, (IGI Global), 337-366.

Abhijith, K. V., Kumar, P., Gallagher, J., McNabola, A., Baldauf, R., Pilla, F., Broderick, B., Di Sabatino, S., & Pulvirenti, B. (2017). Air pollution abatement performances of green infrastructure in open road and built-up street canyon environments–A review. Atmospheric Environment, 162, 71–86.

Ahn, J.-W., Dinh, T.-V., Park, S.-Y., Choi, I.-Y., Park, C.-R., & Son, Y.-S. (2022). Characteristics of biogenic volatile organic compounds emitted from major species of street trees and urban forests. Atmospheric Pollution Research, 13(7), 101470.

AIPH (2025). Green City Case Study: Melbourne, Australia: Grey to Green. International Association of Horticultural Producers. Available online: https://aiph.org/green-city-case-studies/melbourne-australia/ (Accessed on August 28, 2025).

Alauddin, K., Mohd Nusa, F. N., Baharuddin, M. N., Abdul Rashid, M. S., & Remeli, R. (2022). The application of green adaptive reuse of historical buildings in UNESCO cities. Planning Malaysia, 20.

Amorim J.H., Rodrigues, V., Tavares, R., Valente, J., & Borrego, C. (2013). CFD modelling of the aerodynamic effect of trees on urban air pollution dispersion. Science of The Total Environment 461-462, 541-551.

Anders, S., Day, T., & Kuduk, C. A. (2010). "Hey, Your Tree Is Shading My Solar Panels": California's Solar Shade Control Act. Journal of Sustainable Real Estate, 2, 361-381.

Asa'a, S., Reher, T., Rongé, J., Diels, J., Poortmans, J., Radhakrishnan, H. S., van der Heide, A., Van de Poel, B., & Daenen, M. (2024). A multidisciplinary view on agrivoltaics: Future of energy and agriculture. Renewable and Sustainable Energy Reviews, 200, 114515.

Asere, L., Blumberga, A. (2020). Does energy efficiency-indoor air quality dilemma have an impact on the gross domestic product? J. Environ. Manage. 262, 110270.

Asim, N., Badiei, M., Mohammad, M., Razali, H., Rajabi, A., Chin Haw, L., & Jameelah Ghazali, M. (2022). Sustainability of Heating, Ventilation and Air-Conditioning (HVAC) Systems in Buildings—An Overview. International Journal of Environmental Research and Public Health, 19, 1016.

Azadgar, A., Luciani, G., & Nyka, L. (2025). Spatial allocation of nature-based solutions in the form of public green infrastructure in relation to the socio-economic district profile–a GIS-based comparative study of Gdańsk and Rome. Land Use Policy, 150.

Badach, J., Szczepański, J., Bonenberg, W., Gębicki, J., & Nyka, L. (2022). Developing the Urban Blue-Green Infrastructure as a Tool for Urban Air Quality Management. Sustainability, 14, 9688.

Balany, F., Muttil, N., Muthukumaran, S., Wong, M. S., & Ng, A. W. (2022). Studying the effect of blue-green infrastructure on microclimate and human thermal comfort in Melbourne's central business district. Sustainability, 14, 9057.

Bao, X., Zhou, W., Xu, L., & Zheng, Z. (2023). A meta-analysis on plant volatile organic compound emissions of different plant species and responses to environmental stress. Environmental Pollution, 318, 120886.

Barwise, Y., & Kumar, P. (2020). Designing vegetation barriers for urban air pollution abatement: A practical review for appropriate plant species selection. Npj Climate and Atmospheric Science, 3, 12.

Bauduin, T., Gypens, N., & Borges, A. V. (2024). Seasonal and spatial variations of greenhouse gas (CO2, CH4 and N2O) emissions from urban ponds in Brussels. Water research, 253, 121257.

Behzad, Z., & Guilandoust, A. (2024). Enhancing outdoor thermal comfort in a historic site in a hot dry climate (Case study: Naghsh-e-Jahan Square, Isfahan). Sustainable Cities and Society, 102, 105209.

Beier, M., Gerstendörfer, J., Mendzigall, K., Pavlik, D., Trute, P., von Tils, R. (2022). Climate Impact and Model Approaches of Blue-Green Infrastructure Measures for Neighborhood Planning. Sustainability 14, 6861.

Belda, M., Resler, J., Geletič, J., Krč, P., Maronga, B., Sühring, M., Kurppa, M., Kanani-Sühring, F., Fuka, V., Eben, K., Benešová, N., Auvinen, M. (2021). Sensitivity analysis of the PALM model system 6.0 in the urban environment. Geosci. Model Dev. 14, 4443–4464.

Bennetts, H., Soebarto, V., Oakley, S., & Babie, P. (2017). Feeling safe and comfortable in the urban environment. Journal of Urbanism: International Research on Placemaking and Urban Sustainability, 10, 401-421.

Benz S. A., & Burney, J. A. (2021). Widespread Race and Class Disparities in Surface Urban Heat Extremes Across the United States. Earth's Future 9:1–14.

Bressane A., Loureiro A. I. S., Medeiros L. C. d. C., et al. (2024). Overcoming Barriers to Managing Urban Green Spaces in Metropolitan Areas: Prospects from a Case Study in an Emerging Economy. Sustainability 16:7019

Browning, M. H., Rigolon, A., & McAnirlin, O. (2022). Where greenspace matters most: A systematic review of urbanicity, greenspace, and physical health. Landscape and Urban Planning, 217, 104233.

Buccolieri, R., Jeanjean, A. P., Gatto, E., & Leigh, R. J. (2018). The impact of trees on street ventilation, NOx and PM2. 5 concentrations across heights in Marylebone Rd street canyon, central London. Sustainable Cities and Society, 41, 227-241.

Buccolieri, R., Sandberg, M., & Di Sabatino, S. (2010). City breathability and its link to pollutant concentration distribution within urban-like geometries. Atmospheric Environment, 44, 1894-1903.

Calfapietra, C., Fares, S., Manes, F., Morani, A., Sgrigna, G., & Loreto, F. (2013). Role of Biogenic Volatile Organic Compounds (BVOC) emitted by urban trees on ozone concentration in cities: A review. Environmental pollution, 183, 71-80.

Campbell, A., Chanse, V., & Schindler, M. (2024). Developing a conceptual framework for characterizing and measuring social resilience in blue-green infrastructure (BGI). Sustainability, 16, 3847.

Cao, S., Wang, Y., Ni, Z., & Xia, B. (2022). Effects of blue-green infrastructures on the microclimate in an urban residential area under hot weather. Frontiers in Sustainable Cities, 4, 824779.

Caprioli, C., Bottero, M., & De Angelis, E. (2023). Combining an agent-based model, hedonic pricing and multicriteria analysis to model green gentrification dynamics. Computers, Environment and Urban Systems, 102.

Cariñanos, P., & Casares-Porcel, M. (2011). Urban Choigreen zones and related pollen allergy: A review. Some guidelines for designing spaces with low allergy impact. Landscape and urban planning, 101, 205-214.

Chen, L., Liu, C., Zou, R., Yang, M., & Zhang, Z. (2016). Experimental examination of effectiveness of vegetation as bio-filter of particulate matters in the urban environment. Environmental Pollution, 208, 198-208.

Chen, X., Wang, H., & Yang, J. (2024). Effect of green blue spaces on the urban thermal environment: A field study in Hong Kong. Urban Climate, 55, 101912.

Cheung, P. K., Nice, K. A., & Livesley, S. J. (2022). Irrigating urban green space for cooling benefits: The mechanisms and management considerations. Environmental Research: Climate, 1(1), 015001.

Churkina, G., Kuik, F., Bonn, B., Lauer, A., Grote, R., Tomiak, K., & Butler, T. M. (2017). Effect of VOC Emissions from Vegetation on Air Quality in Berlin during a Heatwave. Environmental Science & Technology, 51(11), 6120–6130.

Climate-ADAPT (2022). Paris Oasis Schoolyard Programme, France. European Environment

Cohen-Shacham, E., Walters, G., Janzen, C., & Maginnis, S. (2016). Nature-based solutions to address global societal challenges. IUCN: Gland, Switzerland, 97(2016), 2036.

Considine, B., Gallagher, J., Kumar, P., & McNabola, A. (2023). The impact of street level particulate emissions on the energy performance of roof level building ventilation systems. Journal of Wind Engineering and Industrial Aerodynamics, 233, 105310.

Considine, B., McNabola, A., Kumar, P., & Gallagher, J. (2022). A numerical analysis of particulate matter control technology integrated with HVAC system inlet design and implications on energy consumption. Building and Environment, 211, 108726.

Das, S., Ossola, A., & Beaumont, L. J. (2024). Records of urban occurrences expand estimates of the climate niches in tree species. Global Ecology and Biogeography, 33, e13809.
Dashti, A., Mohammadsharifi, N., Shokuhi, M., & Matzarakis, A. (2024). A comprehensive study on wintertime outdoor thermal comfort of blue-green infrastructure in an arid climate: A case of Isfahan, Iran. Sustainable Cities and Society, 113, 105658.
de Barros Ruas, R., Costa, L. M. S., & Bered, F. (2022). Urbanization driving changes in plant species and communities–A global view. Global Ecology and Conservation, 38, e02243.
Dhar, A., Naeth, M.A., Jennings, P.D., Gamal El-Din, M. (2020). Perspectives on environmental impacts and a land reclamation strategy for solar and wind energy systems. Science of The Total Environment, 718, 134602.
Drosou, N., Soetanto, R., Hermawan, F., Chmutina, K., Bosher, L., & Hatmoko, J. U. D. (2019). Key Factors Influencing Wider Adoption of Blue–Green Infrastructure in Developing Cities. Water, 11, 1234.
Dunlap, A.A., Sovacool, B.K., & Novakovic, B. (2024). "Our town is dying:" Exploring utility-scale and rooftop solar energy injustices in Southeastern California. Geoforum 156, 104120.
Dushkova, D., Taherkhani, M., Konstantinova, A., Vasenev, V. I., & Dovletyarova, E. A. (2025). Understanding Factors Affecting the Use of Urban Parks Through the Lens of Ecosystem Services and Blue–Green Infrastructure: The Case of Gorky Park, Moscow, Russia. Land, 14, 237.
Dutta, M., Herreros-Cantis, P., McPhearson, T., Mustafa, A., Palmer, M. I., Tosca, M., Ventrella, J., & Cook, E. M. (2025). New York City 2100: Environmental justice implications of future scenarios for addressing extreme heat. Landscape and Urban Planning, 254.
Eisenman, T. S., Jariwala, S. P., & Lovasi, G. S. (2019). Urban trees and asthma: a call for epidemiological research. The Lancet Respiratory Medicine, 7, e19-e20.
Elmqvist, T., Setälä, H., Handel, S., Van Der Ploeg, S., Aronson, J., Blignaut, J., Gómez-Baggethun, E., Nowak, D., Kronenberg, J., & De Groot, R. (2015). Benefits of restoring ecosystem services in urban areas. Current Opinion in Environmental Sustainability, 14, 101–108.
Esperon-Rodriguez, M., Rymer, P. D., Power, S. A., Challis, A., Marchin, R. M., & Tjoelker, M. G. (2020). Functional adaptations and trait plasticity of urban trees along a climatic gradient. Urban Forestry & Urban Greening, 54, 126771.
Essak, L., & Ghosh, A. (2022). Floating photovoltaics: A review. Clean Technologies, 4, 752-769.
Fabbri, K., Catalano, M., & Ugolini, A. (2025). Vegetation in Archaeological Areas: Risks, Opportunities, and Guidelines to Preserve or Remove: An Italian Case Study. Sustainability, 17, 2712.
Fei, F., Wang, L., Wang, Y., Yao, W., Fukuda, H., Xiao, Y., Tian, L. & Ji, T. (2023a). A new method for evaluating the synergistic effect of urban water body and vegetation in the summer outdoor thermal environment. Journal of Cleaner Production, 414, 137680.
Fei, F., Wang, Y., Wang, L., Fukuda, H., Yao, W., Zhou, Y. & Dong, X. (2023b). Mechanisms of urban blue-green infrastructure on winter microclimate using artificial neural network. Energy and Buildings, 293, 113188.
Fei, F., Wang, Y., Yao, W., Gao, W. & Wang, L. (2022). Coupling mechanism of water and greenery on summer thermal environment of waterfront space in China's cold regions. Building and Environment, 214, 108912.

Fei, F., Xiao, Y., Wang, L., Wang, Y., Fukuda, H., Yao, W., Yu, H., & Dong, Q. (2024). A novel approach for quantifying the influence intensity of urban water and greenery resources on microclimate for efficient utilization. Sustainable Cities and Society, 112, 105597.

Fellini, S., Marro, M., Del Ponte, A. V., Barulli, M., Soulhac, L., Ridolfi, L., & Salizzoni, P. (2022). High resolution wind-tunnel investigation about the effect of street trees on pollutant concentration and street canyon ventilation. Building and Environment, 226, 109763.

Flammer C. (2020). Green bonds: effectiveness and implications for public policy. Environmental and Energy Policy and the Economy 1:95-128.

Flammer C. (2021). Corporate green bonds. Journal of Financial Economics 142:499-516.

Frantzeskaki, N., McPhearson, T., Collier, M. J., Kendal, D., Bulkeley, H., Dumitru, A., Walsh, C., Noble, K., Van Wyk, E., Ordóñez, C., Oke, C., & Pintér, L. (2019). Nature-based solutions for urban climate change adaptation: linking science, policy, and practice communities for evidence-based decision-making. BioScience, 69, 455-466.

Fricke, L., Legg, R., & Kabisch, N. (2024). Impact of blue spaces on urban microclimate in different climate zones, daytimes and seasons–a systematic review. Urban Forestry & Urban Greening, 128528.

Fukano, Y., Guo, W., Uchida, K., & Tachiki, Y. (2020). Contemporary adaptive divergence of plant competitive traits in urban and rural populations and its implication for weed management. Journal of Ecology, 108, 2521-2530.

Fukano, Y., Uchida, K., & Tachiki, Y. (2023a). Urban-rural gradients: how landscape changes drive adaptive evolution of plant competitive traits. Evolutionary Ecology, 37, 215-232.

Fukano, Y., Yamori, W., Misu, H., Sato, M. P., Shirasawa, K., Tachiki, Y., & Uchida, K. (2023b). From green to red: Urban heat stress drives leaf color evolution. Science Advances, 9, 42.

Gearin, E., Dunson, K., & Hampton, M. (2023). Greened out: mitigating the impacts of eco-gentrification through community dialogue. Architecture_MPS, 25, 1-14.

Géron, C., Lembrechts, J. J., Fameree, M., Taddei, V., Nijs, I., & Monty, A. (2024). Phenotypic plasticity as the main driver of alien plant trait variation in urban versus rural microclimate for the model species Veronica persica. Oecologia, 205, 643-654.

Gibbons J. (2023). Examining the long-term influence of New Deal era redlining on contemporary gentrification. Urban Studies 60:2816-2834.

Gillner, S., Vogt, J., Tharang, A., Dettmann, S., & Roloff, A. (2015). Role of street trees in mitigating effects of heat and drought at highly sealed urban sites. Landscape and Urban Planning, 143, 33-42.

Grabowski, Z. J., McPhearson, T., & Pickett, S. T. A. (2023). Transforming US urban green infrastructure planning to address equity. Landscape and Urban Planning, 229. https://doi.org/10.1016/j.landurbplan.2022.104591

Gromke, C., & Ruck, B. (2007). Influence of trees on the dispersion of pollutants in an urban street canyon experimental investigation of the flow and concentration field. Atmospheric Environment, 41, 3287-3302.

Grossi, F., Ge, H., Zmeureanu, R., & Baba, F. (2023). Feasibility of planting trees around buildings as a nature-based solution of carbon sequestration An LCA approach using two case studies. Buildings, 13, 41.

Gu, S., Guenther, A., & Faiola, C. (2021). Effects of anthropogenic and biogenic volatile organic compounds on Los Angeles air quality. Environmental Science & Technology, 55, 12191-12201.

Guo, Y., Xiao, Q., Ling, C., Teng, M., Wang, P., Xiao, Z., Wu, C. (2023). The right tree for the right street canyons: An approach of tree species selection for mitigating air pollution. Building and Environment 245, 110886.

Haase, D., Larondelle, N., Andersson, E., Artmann, M., Borgström, S., Breuste, J., Gomez-Baggethun, E., Gren, A., Hamstead, Z., Hansen, R., Kabisch, N., Kremer, P., Langemeyer, J., Lorance Rall, E., McPhearson, T., Pauleit, S., Qureshi, S., Schwarz, N., Voigt, A., Wurster D., & Elmqvist. T. (2014). A quantitative review of urban ecosystem service assessments: Concepts, models, and implementation. Ambio, 43, 413–433.

Hahs, A. K., McDonnell, M. J., McCarthy, M. A., Vesk, P. A., Corlett, R. T., Norton, B. A., Clemants, S. E., Duncan, R. P., Thompson, K., Schwartz, M. W., & Williams, N. S. (2009). A global synthesis of plant extinction rates in urban areas. Ecology Letters, 12, 1165-1173.

Hanley, P. A., Arndt, S. K., Livesley, S. J., & Szota, C. (2021). Relating the climate envelopes of urban tree species to their drought and thermal tolerance. Science of The Total Environment, 753, 142012.

He, Q., Wu, L., Lee, C. S., Zhu, C., Bai, W., Guo, W., & Ye, X. (2025). Greener the safer? Effects of urban green space on community safety and perception of safety using satellite and street view imagery data. Journal of Criminal Justice, 97, 102372.

Hernandez, R.R., Easter, S.B., Murphy-Mariscal, M.L., Maestre, F.T., Tavassoli, M., Allen, E.B., Barrows, C.W., Belnap, J., Ochoa-Hueso, R., Ravia, S., & Allen, M.F. (2014). Environmental impacts of utility-scale solar energy. Renewable and Sustainable Energy Review, 29, 766–779.

Holgerson, M. A., & Raymond, P. A. (2016). Large contribution to inland water CO_2 and CH_4 emissions from very small ponds. Nature Geoscience, 9(3), 222–226.

Hoyle, H., Hitchmough, J., & Jorgensen, A. (2017). Attractive, climate-adapted and sustainable? Public perception of non-native planting in the designed urban landscape. Landscape and Urban Planning, 164, 49-63.

Hu, L., & Li, Q. (2020). Greenspace, bluespace, and their interactive influence on urban thermal environments. Environmental Research Letters, 15, 034041.

Huang, B., Zhao, Y., Yang, J., Wang, W., Guo, T., Luo, X., & Du, M. (2024a). Thermal Comfort and Restorative Benefits of Waterfront Green Spaces for College Students in Hot and Humid Regions. Sustainability, 16, 8924.

Huang, S., Xiao, X., Tian, T., & Che, Y. (2024b). Seasonal influences on preferences for urban blue-green spaces: Integrating land surface temperature into the assessment of cultural ecosystem service value. Sustainable Cities and Society, 102, 105237.

Ignatieva, M., Eriksson, F., Eriksson, T., Berg, P., & Hedblom, M. (2017). The lawn as a social and cultural phenomenon in Sweden. Urban Forestry & Urban Greening, 21, 213–223.

Irga, P., Bailes, S., Matheson, S., & Torpy, F. (2024). Incorporation of Green Infrastructure on Road Tunnel Ventilation Stacks: Potential Ambient Air Quality Improvement, Journal of Living Architecture, 10, 1-15.

Islam, M. R., Talukdar, S., Rihan, M., & Rahman, A. (2024). Evaluating cooling effect of blue-green infrastructure on urban thermal environment in a metropolitan city: Using geospatial and machine learning techniques. Sustainable Cities and Society, 113, 105666.

Isola, F., Lai, S., Leone, F., Zoppi, C. (2024). Urban Green Infrastructure and Ecosystem Service Supply: A Study Concerning the Functional Urban Area of Cagliari, Italy. Sustainability, 16(19), 8628.

Janhäll, S. (2015). Review on urban vegetation and particle air pollution – Deposition and dispersion. Atmospheric Environment, 105, 130–137.

Jiadai, T., & Ibrahim, W. Y. B. W. (2025). Visualization microclimate scenarios of Classical Chinese Garden in Suzhou, China. Journal of Infrastructure, Policy and Development, 9(1), 10772.

Jiang, Y., Jiang, S., & Shi, T. (2020). Comparative study on the cooling effects of green space patterns in waterfront build-up blocks: An experience from Shanghai. International journal of environmental research and public health, 17, 8684.

Kabisch, N., & Haase, D. (2014). Green justice or just green? Provision of urban green spaces in Berlin, Germany. Landscape and Urban Planning, 122, 129–139.

Kabisch, N., Qureshi, S., & Haase, D. (2015). Human–environment interactions in urban green spaces A systematic review. Environmental Impact Assessment Review, 50, 25–34.

Kabisch, N., Strohbach, M., Haase, D., & Kronenberg, J. (2016). Urban green space availability in European cities. Ecological Indicators, 70, 586–596.

Kale, E., De Groeve, M., Pinnel, L., Erkan, Y., Hacigüzeller, P., Orr, S. A., & De Kock, T. (2023). Mapping Vertical Greening on Urban Built Heritage Exposed to Environmental Stressors–A Case Study in Antwerp, Belgium. Sustainability, 15, 12987.

Kati, V., & Jari, N. (2016). Bottom-up thinking Identifying socio-cultural values of ecosystem services in local blue–green infrastructure planning in Helsinki, Finland. Land use policy, 50, 537-547.

Kennedy A., Gutterman, S., Pilgrim, B., Ranjan, A., Serre, D., Wason, S., & Climate Risk Task Force (2023). The climate change adaptation gap: An actuarial perspective.

Kondo, M., Hohl, B., Han, S., & Branas, C. (2016). Effects of greening and community reuse of vacant lots on crime. Urban studies, 53, 3279-3295.

Kostadinović, D., Jovanović, M., Bakić, V., Stepanić, N. (2023). Mitigation of urban particulate pollution using lightweight green roof system. Energy Build. 293, 113203.

Kramer J., Silverton S. and Späth P. (2024). Urban governance arrangements for sustainability and justice–linking theory with experience. Urban Transformations 6:6.

Kronenberg, J., Andersson, E., Barton, D. N., Borgström, S. T., Langemeyer, J., Björklund, T., Haase, D., Kennedy, C., Koprowska, K., Łaszkiewicz, E., McPhearson, T., Stange, E. E., & Wolff, M. (2021). The thorny path toward greening: Unintended consequences, trade-offs, and constraints in green and blue infrastructure planning, implementation, and management. Ecology and Society, 26, 36.

Kruitwagen, L., Story, K.T., Friedrich, J., Byers, L., Skillman, S., & Hepburn, C. (2021). A global inventory of photovoltaic solar energy generating units. Nature, 598, 604-611.

Lambert, M. R., Brans, K. I., Des Roches, S., Donihue, C. M., & Diamond, S. E. (2021). Adaptive evolution in cities: progress and misconceptions. Trends in Ecology & Evolution, 36, 239-257.

Langemeyer, J., & Baró, F. (2021). Nature-based solutions as nodes of green-blue infrastructure networks: A cross-scale, co-creation approach. Nature-Based Solutions, 1, 100006.

Larrea, V., Pelaez, F., Esenarro, D. (2024). Design of a Green Corridor and the Revitalization of the Huatanay River, City of Cuzco, Peru 2024. Urban Science, 8, 185 2024.

Lehnert, M., Tokar, V., Jurek, M., & Geletič, J. (2021). Summer thermal comfort in Czech cities: measured effects of blue and green features in city centres. International Journal of Biometeorology, 65, 1277-1289.

Lewartowska, E., Anguelovski, I., Oscilowicz, E., Triguero-Mas, M., Cole, H., Shokry, G., Pérez-del-Pulgar, C., & Connolly, J. J. T. (2024). RACIAL INEQUITY IN GREEN INFRASTRUCTURE AND GENTRIFICATION: Challenging Compounded

Environmental Racisms in the Green City. International Journal of Urban and Regional Research, 48(2), 294–322.
Li Z., Kong L., Hu L., et al. (2024). Greenhouse gas emissions from constructed wetlands: A bibliometric analysis and mini-review. Science of the Total Environment 906:167582.
Li, J., & Nassauer, J. I. (2020). Cues to care: A systematic analytical review. Landscape and Urban Planning, 201, 103821.
Li, J., & Nassauer, J. I. (2021). Technology in support of nature-based solutions requires understanding everyday experiences. Ecology and Society, 26,35.
Li, J., Xu, H., Ren, M., Duan, J., You, W., & Zhou, Y. (2024b). Knowledge Mapping of Cultural Ecosystem Services Applied on Blue-Green Infrastructure A Scientometric Review with CiteSpace. Forests, 15, 1736.
Li, Z., Kong, L., Hu, L., Wei, J., Zhang, X., Guo, W., & Shi, W. (2024a). Greenhouse gas emissions from constructed wetlands: A bibliometric analysis and mini-review. Science of the Total Environment, 906, 167582.
Li, Z., Zhang, H., Juan, Y. H., Lee, Y. T., Wen, C. Y., & Yang, A. S. (2023). Effects of urban tree planting on thermal comfort and air quality in the street canyon in a subtropical climate. Sustainable Cities and Society, 91, 104334.
Lindemann-Matthies, P., & Brieger, H. (2016). Does urban gardening increase aesthetic quality of urban areas? A case study from Germany. Urban forestry & urban greening, 17, 33-41.
Litvak, E., Manago, K. F., Hogue, T. S., & Pataki, D. E. (2017). Evapotranspiration of urban landscapes in Los Angeles, California at the municipal scale. Water Resources Research, 53, 4236-4252.
Liu, H., Kong, F., Yin, H., Middel, A., Zheng, X., Huang, J., Xu, H., Wang, D., Wen, Z. (2021). Impacts of green roofs on water, temperature, and air quality: A bibliometric review. Build. Environ. 196, 107794.
Liu, Y., Skandalos, N., Braslina, L., Kapsalis, V., & Karamanis, D. (2023). Integrating Solar Energy and Nature-Based Solutions for Climate-Neutral Urban Environments. Solar, 3, 382–415
Livesley, S. J., McPherson, E. G., & Calfapietra, C. (2016). The Urban Forest and Ecosystem Services: Impacts on Urban Water, Heat, and Pollution Cycles at the Tree, Street, and City Scale. Journal of Environmental Quality, 45, 119–124.
Locosselli, G. M., Cintra, B. B. L., Ferreira, L. S., Da Silva-Luz, C. L., Miyahara, A. A. L., Brienen, R. J., Gloor, E., Boom, A., Grandis, A., & Buckeridge, M. S. (2024). Stress-tolerant trees for resilient cities: Tree-ring analysis reveals species suitable for a future climate. Urban Climate, 55, 101964.
Lopes, H. S., Vidal, D. G., Cherif, N., Silva, L., & Remoaldo, P. C. (2025). Green infrastructure and its influence on urban heat island, heat risk, and air pollution: A case study of Porto (Portugal). Journal of Environmental Management, 376.
Lovell, S. T., & Taylor, J. R. (2013). Supplying urban ecosystem services through multifunctional green infrastructure in the United States. Landscape Ecology, 28, 1447–1463.
Ma, M., Gao, Y., Ding, A., Su, H., Liao, H., Wang, S., Wang, X., Zhao, B., Zhang, S., Fu, P., Guenther, A.B., Wang, M., Li, S., Chu, B., Yao, X., Gao, H., 2022. Development and Assessment of a High-Resolution Biogenic Emission Inventory from Urban Green Spaces in China. Environ. Sci. Technol. 56, 175–184.
Maison, A., Lugon, L., Park, S.-J., Baudic, A., Cantrell, C., Couvidat, F., d'Anna, B., Karl, T., Kuenen, J. J. P., Lannuque, V., Marais, E. A., Mellouki, A., Petetin, H., Sartelet, K., Tournadre, B., Valari, M., Zaveri, R. A., & Gros, V. (2024). Significant impact of urban

tree biogenic emissions on air quality estimated by a bottom-up inventory and chemistry transport modeling. Atmospheric Chemistry and Physics, 24, 6011–6046.

Manavvi, S., & Milosevic, D. (2025). Chasing cool: Unveiling the influence of green-blue features on outdoor thermal environment in Roorkee (India). Building and Environment, 267, 112238.

Mancilla, D., Robledo, S., Esenarro, D., Raymundo, V., Vega, V. (2024). Green Corridors and Social Connectivity with a Sustainable Approach in the City of Cuzco in Peru. Urban Science, 8, 79.

Marshall C. A., Wilkinson M. T., Hadfield P. M., et al. (2023). Urban wildflower meadow planting for biodiversity, climate and society: An evaluation at King's College, Cambridge. Ecological Solutions and Evidence 4:e12243.

Martínez-Villa, J. A., Paquette, A., Feeley, K. J., Morales-Morales, P. A., Messier, C., & Durán, S. M. (2024). Changes in morphological and physiological traits of urban trees in response to elevated temperatures within an Urban Heat Island. Tree physiology, 44, 145.

Marzbali, M. H., Abdullah, A., Razak, N. A., & Tilaki, M. J. M. (2012). Validating crime prevention through environmental design construct through checklist using structural equation modelling. International Journal of Law, Crime and Justice, 40, 82-99.

Matsler A. M. (2019). Making 'green'fit in a 'grey'accounting system: The institutional knowledge system challenges of valuing urban nature as infrastructural assets. Environmental Science & Policy 99:160-168.

Matsumoto, M., Kiyomizu, T., Yamagishi, S., Kinoshita, T., Kumpitsch, L., Kume, A., Hanba, Y.T. (2022). Responses of photosynthesis and long-term water use efficiency to ambient air pollution in urban roadside trees. Urban Ecosystems, 25, 1029-1042.

McNabb, T., Charters, F. J., Challies, E., & Dionisio, R. (2024a). Unlocking urban blue-green infrastructure: an interdisciplinary literature review analysing co-benefits and synergies between bio-physical and socio-cultural outcomes. Blue-Green Systems, 6, 217-231.

McNabb, T., Charters, F., Dionisio, R., & Challies, E. (2024b). Design and implementation of blue-green infrastructure for socio-cultural benefits at community scales: The case of Wigram Basin in Ōtautahi Christchurch. Nature-Based Solutions, 6, 100192.

Meerow, S., & Newell, J. P. (2017). Spatial planning for multifunctional green infrastructure: Growing resilience in Detroit. Landscape and Urban Planning, 159, 62–75.

Ming, T., Hu, Y., Shi, T., Li, Y., Hu, S., Yang, D., Lv, B., Peng, C., & Chen, Y. (2025). Effect of Blue–Green Infrastructure in mitigating microenvironmental heat islands: Field- and simulation-based insights. Atmosphere, 16, 134.

Minnema, L. (2019). Cross-Cultural Comparisons between the Mughal Tomb Garden of Taj Mahal in Agra (India) and the Dry Landscape Garden of the Ryoan-Ji Zen Monastery in Kyoto (Japan). Worldviews: Global Religions, Culture, and Ecology, 23, 197–229.

Miyahara, A. A. L., Wild, T., Sandre, A. A., Pellegrino, P. R. M., da Silva Filho, C. A., Buckeridge, M. S., & Locosselli, G. M. (2022). Developing and classifying urban biomes as a basis for nature-based solutions. Urban Climate, 45, 101251.

Morgan, D. T., Daly, T., Gallagher, J., & McNabola, A. (2017). Reducing energy consumption and increasing filter life in HVAC systems using an aspiration efficiency reducer: Long-term performance assessment at full-scale. Journal of Building Engineering, 12, 267-274.

Nordh, H. Hartig, T., Hagerhall, C.M., & fry, G.(2009). Components of small urban parks that predict the possibility for restoration. Urban Forestry & Urban Greening, 8, 225–235.Piroozfar, P., Farr, E. R., Aboagye-Nimo, E., & Osei-Berchie, J. (2019). Crime prevention in urban spaces through environmental design: A critical UK perspective. Cities, 95, 102411.

Nordman E. E., Isely E., Isely P., et al. (2018). Benefit-cost analysis of stormwater green infrastructure practices for Grand Rapids, Michigan, USA. Journal of Cleaner Production 200:501-510.

Nowak, D. J., & Crane, D. E. (2002). Carbon storage and sequestration by urban trees in the USA. Environmental Pollution, 116, 381–389.

Nowak, D.J., Greenfield, E.J., Hoehn, R.E., Lapoint, E. (2013). Carbon storage and sequestration by trees in urban and community areas of the United States. Environmental Pollution, 178, 229–236.

Oliveira, P. M. B., Almeida, R. M., & Cardoso, S. J. (2024). Effects of floating photovoltaics on aquatic organisms: a review. Hydrobiologia, 1-16.

Palusci, O. & Cecere, C. (2022). Urban Ventilation in the Compact City: A Critical Review and a Multidisciplinary Methodology for Improving Sustainability and Resilience in Urban Areas. Sustainability, 14, 3948.

Pandey, G., Lyden, S., Franklin, E., Millar, B., & Harrison, M. T. (2025a). A systematic review of agrivoltaics on productivity, profitability, and environmental co-benefits. Sustainable Production and Consumption, 56, 13-36.

Papadopoulou, S., Stefi, A. L., Christodoulakis, N. S., Gkikas, D., & Rhizopoulou, S. (2023). Structural and Physiological Traits of Compound Leaves of Ceratonia siliqua Trees Grown in Urban and Suburban Ambient Conditions. Plants, 12, 514.

Park, B. J., Lee, D. K., Yun, S. H., Kim, E. S., Lee, J. H., & Kim, S. H. (2024). Assessing the impact of green infrastructure on thermal comfort in relation to humidity: A case study in Korea. Urban Forestry & Urban Greening, 95, 128305.

Pauleit, S., Ambrose-Oji, B., Andersson, E., Anton, B., Buijs, A., Haase, D., Elands, B., Hansen, R., Kowarik, I., Kronenberg, J., Mattijssen, T., Stahl Olafsson, A., Rall, E., Van Der Jagt, A. P. N., & Konijnendijk Van Den Bosch, C. (2019). Advancing urban green infrastructure in Europe: Outcomes and reflections from the GREEN SURGE project. Urban Forestry & Urban Greening, 40, 4–16.

Petersen C. J., Russel D. J., Jensen A., et al. (2024). Walkable maps and policy innovation for nature: a novel methodology for understanding policy learning. International Journal of Qualitative Methods 23:16094069241254006.

Pincetl, S., Gillespie, T.W., Pataki, D.E., Porse, E., Jia, S., Kidera, E., Nobles, N., Rodriguez, J., & Choi, D. (2019). Evaluating the effects of turf-replacement programs in Los Angeles. Landscape and Urban Planning, 185, 210–221.

Pinto, D. M., Blande, J. D., Souza, S. R., Nerg, A. M., & Holopainen, J. K. (2010). Plant volatile organic compounds (VOCs) in ozone (O3) polluted atmospheres: the ecological effects. J Chem Ecol, 36, 22-34.

Piroozfar, P., Farr, E. R., Aboagye-Nimo, E., & Osei-Berchie, J. (2019). Crime prevention in urban spaces through environmental design: A critical UK perspective. Cities, 95, 102411.

Pradilla, G., & Hack, J. (2024). An urban rivers renaissance? Stream restoration and green–blue infrastructure in Latin America – Insights from urban planning in Colombia. Urban Ecosystems. https://doi.org/10.1007/s11252-024-01571-9

Pritipadmaja, Garg, R. D. & Sharma, A. K. (2023). Assessing the cooling effect of blue-green spaces: implications for Urban Heat Island mitigation. Water, 15, 2983.

Pugh, T. A. M., MacKenzie, A. R., Whyatt, J. D., & Hewitt, C. N. (2012). Effectiveness of green infrastructure for improvement of air quality in urban street canyons. Environmental Science & Technology, 46, 7692–7699.

Ramasubramanian, P., Luhung, I., Lim, S. B. Y., Schuster, S. C., Starry, O., and Gall, E. T. (2021). Impact of Green and White Roofs on Air Handler Filters and Indoor Ventilation Air. Build. Environ., 197, p. 107860.

Ramasubramanian, P., Starry, O., Rosenstiel, T., Gall, E.T. (2019). Pilot study on the impact of green roofs on ozone levels near building ventilation air supply. Build. Environ. 151, 43–53. https://doi.org/10.1016/j.buildenv.2019.01.023

Raymond, C. M., Frantzeskaki, N., Kabisch, N., Berry, P., Breil, M., Nita, M. R., Geneletti, D., & Calfapietra, C. (2017). A framework for assessing and implementing the co-benefits of nature-based solutions in urban areas. Environmental Science & Policy, 77, 15–24.

RECLAIM (2025). Third RECLAIM Network Plus Conference. https://www.youtube.com/watch?v=ekK0gCXmV5s&list=PL4xPfJG-Mbo4LvhL3rtVptHETgLU6w0GX&index=69.

Reitberger, R., Kooniyara, V.P., Parhizgar, L., & Roetzer, T. (2025). Tree growth simulation in Geographic Information Systems: Coupling CityTree and ArcGIS for solar radiation analysis. Sustainable Cities and Society, 120, 106128.

Řezníček, H., Geletič, J., Belda, M., Beneš, L., Bureš, M., Eben, K., Fuka, V., Krč, P., Michálek, P., Patiño, W., Radović, J., Sühring, M., Vlček, O., Resler, J., 2025. Analysis of the complex role of trees in street canyons using a large-eddy simulation model. Quarterly Journal of the Royal Meteorological Society n/a, e4954.

Ribeiro, A. P., Bollmann, H. A., de Oliveira, A., Rakauskas, F., Cortese, T. T. P., Rodrigues, M. S. C., Quaresma, C. C., & Ferreira, M. L. (2023). The role of tree landscape to reduce effects of urban heat islands: a study in two Brazilian cities. Trees - Structure and Function, 37(1), 17–30.

Rigolon A. and Christensen J. (2019). Greening without gentrification. Parks and recreation. Available online: Parks-Related-Anti-Displacement-Strategies-report-with-appendix.pdf (accessed on 09 May 2025).

Rosentreter, J.A., Borges, A.V., Deemer, B.R., Holgerson, M.A., Liu, S., Song, C., Melack, J., Raymond, P.A., Duarte, C.M., Allen, G.H., Olefeldt, D., Poulter, B., Battin, T.I., & Eyre, B.D. (2021). Half of global methane emissions come from highly variable aquatic ecosystem sources. Nature Geoscience, 14, 225–230

Saher, R., Ott, T. (2025). Assessing the irrigation water requirement and irrigation water use at a house scale in Las Vegas Valley. Agricultural Water Management, 308, 109278.

Salmond, J.A., Williams, D.E., Laing, G., Kingham, S., Dirks, K., Longley, I., Henshaw, G.S. (2013). The influence of vegetation on the horizontal and vertical distribution of pollutants in a street canyon. Science of The Total Environment, 443, 287–298.

Salvador, C. M., Chou, C. C. K., Ho, T.-T., Tsai, C.-Y., Tsao, T.-M., Tsai, M.-J., & Su, T.-C. (2020). Contribution of Terpenes to Ozone Formation and Secondary Organic Aerosols in a Subtropical Forest Impacted by Urban Pollution. Atmosphere, 11, 1232.

Säumel, I., Weber, F., & Kowarik, I. (2016). Toward livable and healthy urban streets: Roadside vegetation provides ecosystem services where people live and move. Environmental Science & Policy, 62, 24-33.

Sawers, B. (2019). Controlling Biogenic Volatile Organic Compounds for Air Quality. Indiana Law Journal, 94, 79-90.

Shi, Z., Yang, J., Zhang, Y., Xiao, X., Xia, C.J. (2022). Urban ventilation corridors and spatiotemporal divergence patterns of urban heat island intensity: a local climate zone perspective. Environmental Science and POllution Research, 29, 74394–74406.

Shokry, G., Anguelovski, I., & Connolly, J. J. T. (2023). (Mis-)belonging to the climate-resilient city: Making place in multi-risk communities of racialized urban America. Journal of Urban Affairs.

Sierra-Heredia, C., North, M., Brook, J., Daly, C., Ellis, A. K., Henderson, D., Henderson, S. B., Lavigne, É., & Takaro, T. K. (2018). Aeroallergens in Canada: Distribution, public health impacts, and opportunities for prevention. International Journal of Environmental Research and Public Health, 15, 1577.

Silva Luz, C. L. D., Reale, R., Candido, L. F., Zappi, D., & Locosselli, G. M. (2024). Using Morphological Characters to Support Decision-Making in Nature-Based Solutions: A Shortcut to Promote Urban Plant Biodiversity. Urban Science, 8, 233.

Sjöman, H., Östberg, J., & Bühler, O. (2012). Diversity and distribution of the urban tree population in ten major Nordic cities. Urban Forestry & Urban Greening, 11, 31–39.

Skandalos, N., & Karamanis, D. (2025). Decarbonizing operational emissions in urban neighborhoods with the integration of rooftop photovoltaics and green infrastructure under current and future climate conditions. Energy & Buildings, 329, 115306.

Sotillo, A., Hardion, L., Chanez, E., Fujiki, K., & Muratet, A. (2024). Plant responses to urban gradients: Extinction, plasticity, adaptation. Journal of Ecology, 112, 2861-2875.

Sousa-Silva, R., Smargiassi, A., Kneeshaw, D., Dupras, J., Zinszer, K., & Paquette, A. (2021). Strong variations in urban allergenicity riskscapes due to poor knowledge of tree pollen allergenic potential. Scientific Reports, 11, 10196.

Stawoska, I., Myszkowska, D., Oliwa, J., Skoczowski, A., Wesełucha-Birczyńska, A., Saja-Garbarz, D., & Ziemianin, M. (2023). Air pollution in the places of Betula pendula growth and development changes the physicochemical properties and the main allergen content of its pollen. Plos one, 18, e0279826.

Sun, Hao, Carlos Jimenez-Bescos, Murtaza Mohammadi, Fangliang Zhong, and John Kaiser Calautit. (2021). Numerical investigation of the influence of vegetation on the aero-thermal performance of buildings with courtyards in hot climates. Energies 14, 5388.

Sun, X., Tan, X., Chen, K., Song, S., Zhu, X., & Hou, D. (2020). Quantifying landscape-metrics impacts on urban green-spaces and water-bodies cooling effect: The study of Nanjing, China. Urban Forestry & Urban Greening, 55, 126838.

Tache A.-V., Popescu O.-C. and Petrişor A.-I. (2024). Planning Blue–Green Infrastructure for Facing Climate Change: The Case Study of Bucharest and Its Metropolitan Area. Urban Science 8:250.

Talal, M. L., Santelmann, M. V., & Tilt, J. H. (2021). Urban park visitor preferences for vegetation – An on-site qualitative research study. Plants, People, Planet, 3, 375–388.

Tan, X., Sun, X., Huang, C., Yuan, Y., & Hou, D. (2021). Comparison of cooling effect between green space and water body. Sustainable Cities and Society, 67, 102711.

Tang, Y.-F., Wen, Y.-B., Chen, H., Tan, Z.-C., Yao, Y.-H., & Zhao, F.-Y. (2023). Airflow Mitigation and Pollutant Purification in an Idealized Urban Street Canyon with Wind Driven Natural Ventilation: Cooperating and Opposing Effects of Roadside Tree Plantings and Non-uniform Building Heights. Sustainable Cities and Society, 92, 104483.

Tao, Q., Gao, G., Xi, H., Wanf, F., Cheng, X., Ou, W., Tao, Y. (2022). An integrated evaluation framework for multiscale ecological protection and restoration based on multi-scenario trade-offs of ecosystem services: Case study of Nanjing City, China.Ecological Indicators, 140, 108962.

Theeuwes, N. E., Solcerová, A., & Steeneveld, G. J. (2013). Modeling the influence of open water surfaces on the summertime temperature and thermal comfort in the city. Journal of Geophysical Research: Atmospheres, 118(16), 8881–8896.

Tomateo, C. (2021). Indigenous land systems and emerging of Green Infrastructure planning in the Peruvian coastal desert: Tensions and opportunities. Journal of Environmental Policy & Planning, 23, 683–700.

Triyuly, W., Triyadi, S., & Wonorahardjo, S. (2021). Synergising the thermal behaviour of water bodies within thermal environment of wetland settlements. International Journal of Energy and Environmental Engineering, 12(1), 55–68.

van Bergen, T.J.H.M., Barros, N., Mendonça, R., Aben, R.C.H., Althuizen, I.H.J., Huszar, V., Lamers, L.P.M., Lürling, M., Roland, F., Kosten, S. (2019). Seasonal and diel variation in greenhouse gas emissions from an urban pond and its major drivers. Limnology and Oceanography, 64, 2129–2139.

Van Mechelen, C., Dutoit, T., Hermy, M. (2015). Adapting green roof irrigation practices for a sustainable future: A review. Sustainable Cities and Society, 19, 74–90.

Vasudevan, M., Pilla, F., & McNabola, A. (2024). Assessment of pollution removal mechanisms in steep-asymmetric city-type environments using wind deflectors. Energy & Built Environment (in press).

Velasco, E., Roth, M., Norford, L., Molina, L.T. (2016). Does urban vegetation enhance carbon sequestration? Landscape and Urban Planning, 148, 99–107.

Wahba, S.M., Kamel, B.A., Nassar, K.M., Abdelsalam, A.S. (2018). Effectiveness of Green Roofs and Green Walls on Energy Consumption and Indoor Comfort in Arid Climates. Civ. Eng. J. 4, 2284–2295.

Wang H. X., Zhao, J. H., Lu, J., Ge, J., Lv, G. Q., Luo, X. Y., Lin, H. Q., Gu, M. Y. (2025). Combined impacts of vertical greening and permeable pavement systems on street Canyons' microclimate in hot and humid regions in China. Urban Climate 59:102333.

Wang, W., He, J. & Wang, X. (2024). Quantitatively comparing the morphological influences on the cool island effect in urban waterfront blue-green spaces across six cities near 30° N. Urban Climate, 56, 102076.

Wang, W., Yang, H., & Xiang, C. (2023a). Green roofs and facades with integrated photovoltaic system for zero energy eco-friendly building–A review. Sustainable Energy Technologies and Assessments, 60, 103426.

Wang, Y., Li, B., Bao, P., Wang, R., Min, A., & Xiong, P. (2023). A Case Study of Leaf Wettability Variability and the Relations with Leaf Traits and Surface Water Storage for Urban Landscape Plants. Water, 15, 2152.

Wedyan M. and Saeidi-Rizi F. (2025). Assessing the impact of walkability indicators on health outcomes using machine learning algorithms: A case study of Michigan. Travel Behaviour and Society 39:100983.

Wei, Y., Li, Z., Zhang, J., & Hu, D. (2023). Effects of artificial light at night and drought on the photosynthesis and physiological traits of two urban plants. Frontiers in Plant Science, 14, 1263795.

Wilfong, M., Litvak, E., Grijseels, N.H., Hamilton, K., Kucera, D., Welsh, L., Endter-Wada, J., Jenerette, G.D., Pataki, D.E. (2025). Irrigation rates and turfgrass evapotranspiration in cities with contrasting water availability. Journal of the American Water Resources Association, 61, e13236.

Wolch, J.R., Byrne, J. & Newell, J.P. (2014). Urban green space, public health, and environmental justice: The challenge of making cities 'just green enough'. Landscape and Urban Planning, 125, 234-244.

Wong C.P., Jiang B. Kinzig A.P., & Ouyangi Z. (2018). Quantifying multiple ecosystem services for adaptive management of green infrastructure. Ecosphere, 9, e02495.

Woszczyk, M., & Schubert, C. J. (2021). Greenhouse gas emissions from Baltic coastal lakes. Science of The Total Environment, 755, 143500.

Woudstra, Y., Kraaiveld, R., Jorritsma, A., Vijverberg, K., Ivanovic, S., Erkens, R., Huber, H., Gravendeel, B., & Verhoeven, K. J. (2024). Some like it hot: adaptation to the urban heat island in common dandelion. Evolution Letters, 8, 881-892.

Xiong Y., Zhang J., Xu X., et al. (2020). Strategies for improving the microclimate and thermal comfort of a classical Chinese garden in the hot-summer and cold-winter zone. Energy and Buildings 215:109914.

Xiong, G., He, R., Wang, G., Hong, J., & Jin, Y. (2024). Environmental Inequalities in Ecosystem Services Benefits of Green Infrastructure: A Case Study from China. Forests, 15(1).

Xiong, J., Li, B., Short, C. A., Kumar, P., & Pain, C. (2024). Comprehensive evaluation of natural ventilation potential of buildings in urban areas under the influence of multiple environment-related factors. Journal of Building Engineering, 89, 109218.

Xu, X., Luo, F., Wang, W., Hong, T., & Fu, X. (2018). Performance-Based Evaluation of Courtyard Design in China's Cold-Winter Hot-Summer Climate Regions. Sustainability, 10, 3950.

Yang, G., Yu, Z., Jørgensen, G., & Vejre, H. (2020). How can urban blue-green space be planned for climate adaption in high-latitude cities? A seasonal perspective. Sustainable Cities and Society, 53, 101932.

Yang, G., Yu, Z., Jørgensen, G., & Vejre, H. (2020). How can urban blue-green space be planned for climate adaption in high-latitude cities? A seasonal perspective. Sustainable Cities and Society, 53, 101932.

Yao, L., Sailor, D., Yang, X., Xu, G., Zhao, L. (2023). Are water bodies effective for urban heat mitigation? Evidence from field studies of urban lakes in two humid subtropical cities, Building and Environment, 245, 110860.

Yin, J., Chen, X., Xie, W., & Wen, L. (2025). Urban lakes as significant sources of greenhouse gas (CO2, CH4, and N2O) emissions: Insights from field measurements and statistical analyses. Environmental Monitoring and Assessment, 197(5), 1–18.

Yuan Q., Meng F., Li W., et al. (2025). Tradeoff optimization of urban roof systems oriented to food-water-energy nexus. Applied Energy 380:124987.

Yuan, Q., Lian, J., Yang, F., Shen, M., Wang, Y., Kong, Q., Chen, B., Cai, X., Tao, H., & Wu, H. (2025). A systematic review on greenhouse gas emissions from constructed wetlands: Focusing on effects of planting strategies and emission reduction measures. Journal of Water Process Engineering, 69, 106696.

Zhang, H., Yu, Z., Zhu, C., Yang, R., Yan, B., & Jiang, G. (2023a). Green or not? Environmental challenges from photovoltaic technology. Environmental Pollution, 320, 121066.

Zhang, N., Wang, G., Gallagher, J., Song, Q., Tam, V. W. Y., & Duan, H. (2020). A dynamic analysis of the global warming potential associated with air conditioning at a city scale: an empirical study in Shenzhen, China, Environmental Impact Assessment Review, 81, 106354

Zhang, W., Li, H., Xiao, Q., & Li, X. (2021). Urban rivers are hotspots of riverine greenhouse gas (N2O, CH4, CO2) emissions in the mixed-landscape chaohu lake basin. Water Research, 189, 116624.

Zhang, X., Buddhika, J. W. G., Wang, J., Weerasuriya, A. U., & Tse, K. T. (2023b). Numerical investigation of effects of trees on cross-ventilation of an isolated building. Journal of Building Engineering, 73, 106808.

Zhang, Y., & Steiner, A. L. (2022). Projected climate-driven changes in pollen emission season length and magnitude over the continental United States. Nature communications, 13, 1234.
Zhao, Z., Li, H., Wang, S. (2024). Machine learning-based surrogate models for fast impact assessment of a new building on urban local microclimate at design stage. Build. Environ. 266, 112142.
Zhao N., Prieur J.-F., Liu Y., et al. (2021). Tree characteristics and environmental noise in complex urban settings–A case study from Montreal, Canada. Environmental Research 202:111887.
Zhong, Q., & Tong, D. (2020). Spatial layout optimization for solar photovoltaic (PV) panel installation. Renewable energy, 150, 1-11.
Zhu Y., Ding J., Zhu Q., et al. (2017). The impact of green open space on community attachment—A case study of three communities in Beijing. Sustainability 9:560.
Schweinberger M. C. (2023). Debt for Nature Swaps-Birth of a New Asset Class? Available at SSRN 4843793.
